# Late-stage *gem*-difluoroallylation of phenol in bioactive molecules and peptides with 3,3-difluoroallyl sulfonium salts†

**DOI:** 10.1039/d3sc06302j

**Published:** 2024-01-15

**Authors:** Minqi Zhou, Jin-Xiu Ren, Xiao-Tian Feng, Hai-Yang Zhao, Xia-Ping Fu, Qiao-Qiao Min, Xingang Zhang

**Affiliations:** a College of Chemistry and Henan Institute of Advanced Technology, Zhengzhou University Zhengzhou 450001 China; b Key Laboratory of Fluorine and Nitrogen Chemistry and Advanced Materials (Chinese Academy of Sciences), Shanghai Institute of Organic Chemistry, University of Chinese Academy of Sciences, Chinese Academy of Sciences 345 Lingling Road Shanghai 200032 China xgzhang@mail.sioc.ac.cn

## Abstract

An efficient method for the late-stage selective *O*-fluoroalkylation of tyrosine residues with a stable yet highly reactive fluoroalkylating reagent, 3,3-difluoroallyl sulfonium salts (DFASs), has been developed. The reaction proceeds in a mild basic aqueous buffer (pH = 11.6) with high efficiency, high biocompatibility, and excellent regio- and chemoselectivity. Various oligopeptides and phenol-containing bioactive molecules, including carbohydrates and nucleosides, could be selectively *O*-fluoroalkylated. The added vinyl and other functional groups from DFASs can be valuable linkers for successive modification, significantly expanding the chemical space for further bioconjugation. The synthetic utility of this protocol has been demonstrated by the fluorescently labeled anti-cancer drug and the synthesis of O-link type 1,4,7,10-tetraazacyclododecane-*N*,*N*′,*N*,*N*′-tetraacetic acid-tyrosine^3^-octreotate (DOTA-TATE), showing the prospect of the method in medicinal chemistry and chemical biology.

## Abstract 2

An efficient method for the late-stage selective *O*-fluoroalkylation of tyrosine residues with 3,3-difluoroallyl sulfonium salts (DFASs) has been developed. The added functional groups from DFASs can serve as linkers for successive modifications.

## Graphical abstract

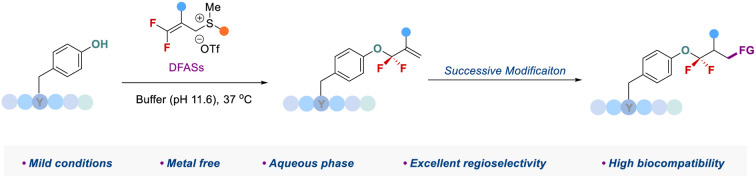

## Introduction

The high demand for discovering new drug leads and the increasing interest in chemical biology have triggered extensive efforts on the site-specific late-stage modification of bioactive molecules, peptides, and proteins.^1^ In this context, the development of site-selective late-stage fluoroalkylation (LSF) reactions has emerged as an intriguing research topic, mainly due to the unique properties of fluorine atom(s) that often significantly change the physicochemical and biological properties of organic molecules.^2^ In particular, the site-specific introduction of fluorine functionalities into peptides has become one of the powerful tactics to modulate their acidity, basicity, hydrophobicity, geometry conformation, and bioavailability.^3^ Moreover, because of the absence of fluorine atoms in native biomolecules, fluorine functional groups can also serve as a probe to study the protein-ligand interaction and the instant conformational changes *via*^19^F NMR.^4^ Consequently, elegant progress has been made in the LSF of peptides over the past decade.

**Fig. 1:**
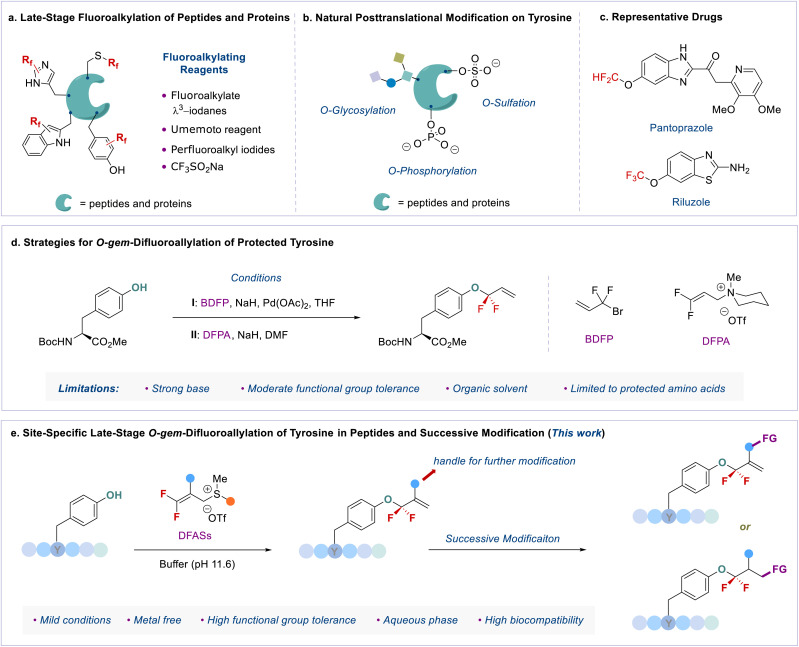
Site-specific late-stage fluoroalkylation of peptides and proteins and representative drugs bearing the fluoroalkyl aryl ether motif.

Compared to traditional solid phase peptide synthesis (SPPS),^5^ this LSF strategy features synthetic convenience and simplicity without the tedious synthesis of fluorinated amino acids. However, most developed methods focus on the *S*-fluoroalkylation of highly nucleophilic cysteine.^6^ Taking advantage of fluoroalkyl radicals, the direct C–H bond fluoroalkylations of amino acid residues bearing an electron-rich aromatic ring, such as indole, phenol, and imidazole moieties,^4*b*,7^ have also been developed. Despite the significance of these achievements, the development of new and efficient methods for LSF of peptides remains in high demand, because of the following crucial issues: (1) site-specificity: the developed methods are limited to cysteine (γ-S),^6^ tryptophan (indole-C),^7^ tyrosine (phenol-C),^7*b*^ and histidine (imidazole-C)^7*c*^ fluoroalkylations (Fig. 1a); (2) fluorine space: usually, perfluoroalkyl groups, such as the trifluoromethyl (CF_3_) group, are used to modify peptides,^4*a*,*b*,7*a*,*b*,8^ thereby regulating the exploration of the unique fluorine effect of different types of fluorine functionalities in peptidomimetics; (3) lack of efficient fluoroalkylating reagents: the developed methods heavily rely on the fluoroalkylated hypervalent iodine reagent (*e.g.*, Togni reagent),^4*b*,8^ Umemoto reagent,^7*b*^ Langlois' reagent (CF_3_SO_2_Na),^4*a*,7*a*^ or perfluoroalkyl iodides^9^ (Fig. 1a).

We envisioned that the site-specific *O*-fluoroalkylation of tyrosine residues would be a promising alternative to modifying peptides and proteins because (1) natural *O*-modification of tyrosine, such as phosphorylation,^10^ glycosylation,^11^ and sulfation,^12^ is usually involved in many vital bioprocesses (Fig. 1b). The site-specific *O*-fluoroalkylation of a tyrosine residue would add a new tool to the modification of biomolecules toolbox; (2) the fluoroalkylation of tyrosine residues on peptides can increase their hydrophobicity and metabolic stability, thus improving the membrane permeability and bioavailability of peptides;^13^ (3) compared to *O*-alkylation, *O*-fluoroalkylation of phenol causes a reduced in-plane conformational preference due to the hyperconjugative 
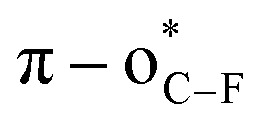
 interaction.^14^ This unique conformational property has been applied to modern drug design and development.^15^ For instance, the fluoroalkyl aryl ether motif has been used in different drugs (Fig. 1c), such as pantoprazole®, a marketed proton-pump inhibitor used in the treatment of gastroesophageal reflux disease (GERD),^16^ and riluzole, a neuroprotective drug that blocks glutamatergic neurotransmission in the CNS.^17^ However, most developed methods for the fluoroalkylation of phenols are limited to trifluoro- and difluoro-methylation of simple substrates. As such, developing a new method that can site-selectively form an *O*-CF_2_R bond with phenolic complexes in the aqueous phase and enlarge the chemical space for modification of peptides and bioactive molecules is of great interest.

Here, we report an efficient method for late-stage *gem*-difluoroallylation of phenol in bioactive molecules and peptides with a bench-stable yet highly active fluoroalkylating reagent, 3,3-difluoroallyl sulfonium salts (DFASs) (Fig. 1e). The reaction proceeds in a mild basic aqueous buffer (pH = 11.6) without a metal catalyst, showing high phenolic site-specificity and high biocompatibility with different amino acid residues and carbohydrates. The added vinyl and other functional groups from DFASs provide versatile handles for successive modification to construct useful bioconjugates, rendering the approach valuable for applications in medicinal chemistry and chemical biology.

## Results and discussion

A critical factor in site-specific *O*-fluoroalkylation of tyrosine residue with enlarged chemical space that not only enables the exploration of the unique fluorine effect in peptidomimetics, but also offers a new opportunity for further bioconjugation, is identifying a suitable fluoroalkylating reagent. Very recently, we developed a highly active fluoroalkylating reagent, 3,3-difluoroallyl sulfonium salt (DFAS), that can efficiently construct the C–CF_2_R bond *via* copper catalysis.^18^ We envisioned that DFAS would be an attractive choice for site-specific *O*-fluoroalkylation of tyrosine, as the added carbon–carbon double bond provides a versatile handle for successive modification. Furthermore, the difluoromethylene (CF_2_) group possesses unique properties that have important applications in medicinal chemistry.^2,19^ The *O*-selective introduction of the CF_2_ group onto tyrosine may provide a new opportunity to explore the CF_2_ effect in chemical biology. Although the *gem*-difluoroallylation of protected tyrosine with 3-bromo-3,3-difluoropropene (BDFP)^14^ and 3,3-difluoropropen-1-yl ammonium salts (DFPASs)^20^ has been reported, the low reactivity of these *gem*-difluoroallylating reagents requires a strong base, such as NaH, to promote the reaction (Fig. 1d). While NaH is moisture sensitive and can cause the racemization and hydrolysis of peptides,^21^ it thus fails to modify peptides and proteins under biocompatible conditions, such as aqueous phase and biocompatibility with different amino acid residues.

To test our assumption, protected tyrosine 1a was chosen as a model substrate (Table 1). No reaction occurred without base (entries 1–3). The addition of 1.0 equiv. of Na_2_CO_3_ to the solution of 1a (1.0 equiv.) and 2a (1.0 equiv.) in DMSO at 37 °C could provide a mixture of regioisomers 3a and 4a with poor *α*-regioselectivity (*α*/*γ* = 1 : 1.6), in which the desired *gem*-difluoroallylated product 3a was obtained in 7% yield (entry 4). Although a low yield was provided, this result encouraged us to test aqueous phase conditions. A series of common basic buffers frequently used in chemical biology were examined in combination with DMSO as a cosolvent (entries 5–7). CBS buffer shows a beneficial effect on the reaction efficiency and *α*-regioselectivity (entries 7–11), and a 95% isolated yield of 3a (*α*/*γ* > 20 : 1) was obtained when CBS solution (pH = 11.62, 0.1 M, aqueous Na_2_CO_3_) was used (entry 11). Other organic co-solvents were also examined. DMF, dichloromethane (DCM), acetonitrile, and acetone afforded 3a in comparable yields (entries 12–15). However, methanol decreased the yield of 3a to 65% (entry 16). We also compared the reactivities of DFAS 2a with those of other *gem*-difluoroallylating reagents. *gem*-Difluoroallyl ammonium salt DFPA afforded 3a in only 25% yield under the same reaction conditions (entry 17), and no product was observed with *gem*-difluoroallyl bromide BDFP (entry 18), thus featuring the highest reactivity of 2a. To demonstrate the unique fluorine effect of 2a, the reaction of allyl sulfonium salt 2a′ was conducted, providing the corresponding allyl product 3a′ in only 15% yield after prolonging the reaction time to 24 h (entry 19). Kinetic studies showed that the formation of allyl product 3a using 2a is much faster than using 2a′ (Fig. S1 and S2†). Compound 3a could be obtained in 90% yield at 5 min (Table S3†), while only 12% yield of 3a′ was produced with 2a′ at 30 min (Table S4†). We ascribed this beneficial effect to the strong electron-withdrawing effect of fluorine that activates the C–S bond in 2a.

**Table 1:** Optimization of the reaction conditions^a^

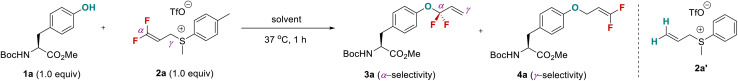							
Entry	Solvent	3a and 4a		Entry	Solvent	3a and 4a	
		3a/4a yield^b^ (%)	*α*/*γ*			3a/4a yield^b^ (%)	*α*/*γ*
1	DCM	0	—	11	CBS (pH = 11.62, 0.1 M)/DMSO (1 : 1, v/v)	>99 (95)/—	>20 : 1
2	DMSO	0	—	12	CBS (pH = 11.62, 0.1 M)/DMF (1 : 1, v/v)	95/—	>20 : 1
3	DMF	0	—	13	CBS (pH = 11.62, 0.1 M)/DCM (1 : 1, v/v)	96/—	>20 : 1
4	DMSO with 1.0 equiv. Na_2_CO_3_	7/11	1 : 1.6	14	CBS (pH = 11.6, 0.1 M)/MeCN (1 : 1, v/v)	94/—	>20 : 1
5	PBS (pH = 7.6, 0.1 M)/DMSO (1 : 1, v/v)	36/4	9 : 1	15	CBS (pH = 11.62, 0.1 M)/acetone (1 : 1, v/v)	95/—	>20 : 1
6	Tris (pH = 8.9, 0.1 M)/DMSO (1 : 1, v/v)	5.5/3.5	1.6 : 1	16	CBS (pH = 11.62, 0.1 M)/MeOH (1 : 1, v/v)	61/—	>20 : 1
7	CBS (pH = 8.30, 0.1 M)/DMSO (1 : 1, v/v)	37/—	>20 : 1	17^c^	CBS (pH = 11.62, 0.1 M)/DMSO (1 : 1, v/v)	25/—	>20 : 1
8	CBS (pH = 9.40, 0.1 M)/DMSO (1 : 1, v/v)	63/—	>20 : 1	18^d^	CBS (pH = 11.62, 0.1 M)/DMSO (1 : 1, v/v)	0	—
9	CBS (pH = 9.72, 0.1 M)/DMSO (1 : 1, v/v)	71/—	>20 : 1	19^e^	CBS (pH = 11.62, 0.1 M)/DMSO (1 : 1, v/v)	15/—	—
10	CBS (pH = 10.08, 0.1 M)/DMSO (1 : 1, v/v)	78/—	>20 : 1				

a Reaction conditions (unless otherwise specified): 1a (0.2 mmol, 1.0 equiv.), 2a (1.0 equiv.), solvent (4 mL) 37 °C, 1 h.

b Determined by ^19^F NMR using fluorobenzene as an internal standard; the number given in parentheses is the isolated yield.

c DFPA was used instead of 2a.

d BDFP was used instead of 2a.

e Allyl sulfonium salt 2a′ was used instead of 2a, and the yield is for 3a′.

**Scheme 1:**
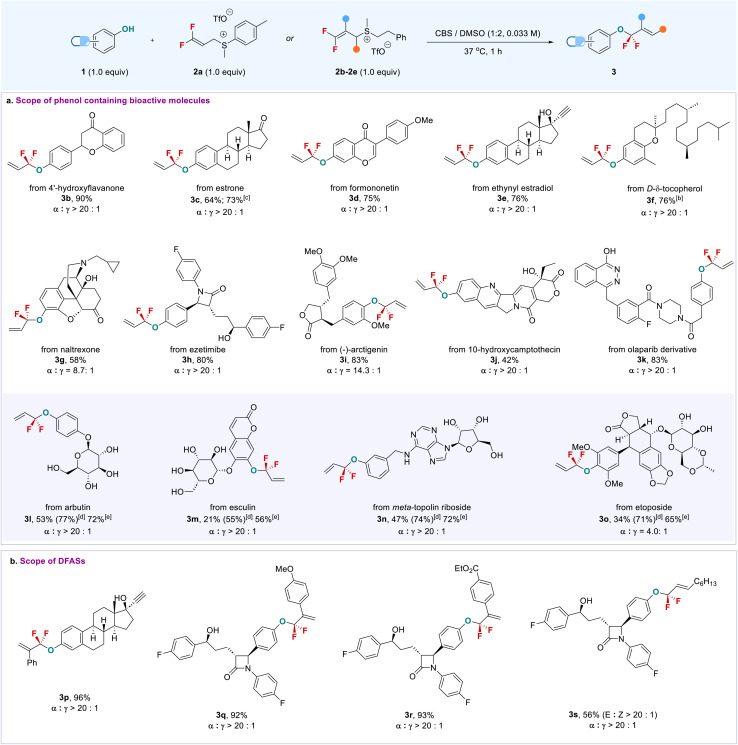
Late-stage *O-gem*-difluoroallylation of complex phenol-containing bioactive molecules. ^*a*^Reaction conditions: 1 (0.2 mmol, 1.0 equiv.), 2 (0.2 mmol, 1.0 equiv.), CBS (2 mL), DMSO (4 mL), 37 °C. All the ratios (*α*/γ) were determined by ^19^F NMR before working up. ^*b*^DCM instead of DMSO was used. ^*c*^Gram-scale reaction. ^*d*^Yield was determined by ^19^F NMR using fluorobenzene as an internal standard. ^*e*^Two steps overall yield: *O-gem*-difluoroallylation, followed by acetylation.

With the viable reaction conditions, we started to examine the phenol-containing bioactive molecules, in which a mixture of DMSO/CBS (2/1) was used due to the low solubility of some substrates in the aqueous phase (Scheme 1). Generally, this late-stage *O-gem*-difluoroallylation process shows excellent regio- (*α*/*γ* > 20 : 1) and chemoselectivity and high functional group tolerance, especially for enolizable carbonyl and chiral centers that are prone to racemization under basic conditions and were compatible with the current buffer solution. As shown in Scheme 1a, 4′-hydroxyflavanone (3b) and estrone (3c) bearing enolizable ketone underwent the phenolic *gem*-difluoroallylation smoothly without observation of the side products formed from the nucleophilic attack of ketone enolate on the 2a*via* an S_N_2′ pathway. This finding is in sharp contrast to the previous strongly basic conditions that were not only inapplicable to the aqueous phase,^14,20^ but also would lead to different fluorinated products due to enolizable ketone. Formononetin (3d) and d-δ-tocopherol (3f) were also applied to the reaction; even the terminal alkyne and free alcohol-containing ethynyl estradiol (3e) did not interfere with the reaction efficiency. Notably, drugs containing multi-chiral carbon centers, which would be racemized or decomposed under strongly basic conditions, were also competent coupling partners. For instance, long-lasting opioid antagonist naltrexone^22^ (3g) and cholesterol absorption inhibitor ezetimibe^23^ (3h) furnished their corresponding products smoothly. Even anti-cancer drugs, (−)-arctigenin (3i) and 10-hydroxycamptothecin (3j), were suitable substrates, thus offering new opportunities to modify their structures for bioconjugation or to discover new bioactivities. Importantly, olaparib (3k), a PARP inhibitor used for cancer treatment, showed high phenolic chemoselectivity,^24^ though it contains a phthalazin-1-ol motif that may also undergo *gem*-difluoroallylation with 2a.

**Scheme 2:**
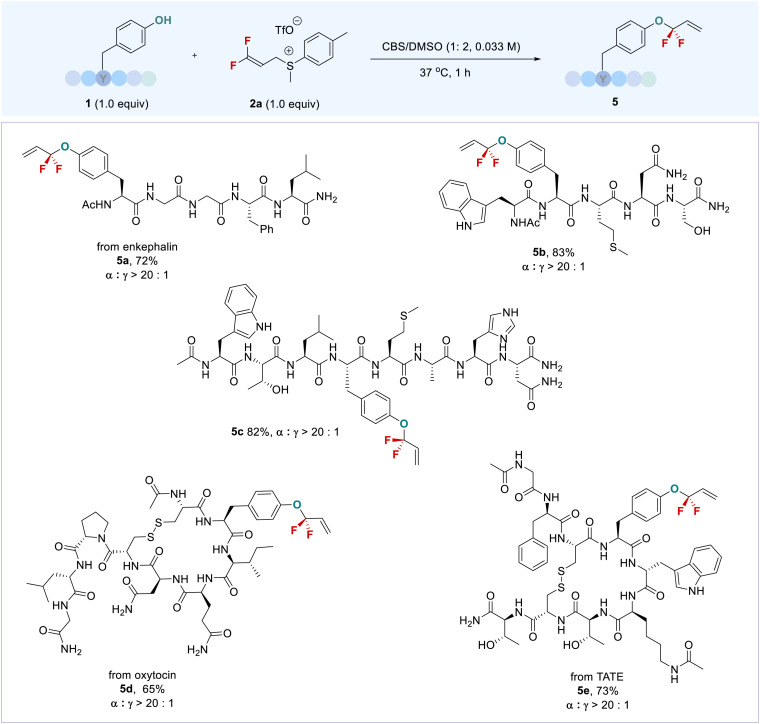
Late-stage *O-gem*-difluoroallylation of tyrosine residues in peptides with DFAS 2a. ^*a*^Reaction conditions: 1 (0.05 mmol, 1.0 equiv.), 2 (0.05 mmol, 1.0 equiv.), CBS (2 mL), DMSO (4 mL), 37 °C.

The high functional group tolerance and chemoselectivity of this approach can also be demonstrated by the late-stage phenolic *O-gem*-difluoroallylation of carbohydrate-containing bioactive molecules. Arbutin (3l) and esculin (3m) bearing an unprotected sugar ring exclusively provided phenolic *gem*-difluoroallylation products with high efficiency; even *meta*-topolin riboside (3n) was amenable to the reaction without influence by the presence of an adenine moiety, thereby providing a new tool to modify the carbohydrates and nucleic acids. Because of the high hydrophilicity of these fluorinated carbohydrate derivatives, their isolated yields were not good, though high ^19^F NMR yields were observed. This issue could be addressed by a sequential procedure: direct phenolic *gem*-difluoroallylation, followed by the acetylation of the carbohydrates, providing protected products with high efficiency. Notably, the anti-cancer drug etoposide exhibited high reactivity (3o). The steric effect that arose from its two *ortho*-substituted methoxy groups did not affect the allylation yield, but the *α*-regioselectivity of the product was decreased (*α*/*γ* = 4.0 : 1). The higher regioselectivity could be obtained after purification of product 3o (*α*/*γ* = 14.3 : 1, see the ESI†). In addition to 2a, a variety of DFASs 2b–2e bearing aryl or alkyl substituents were examined, providing the corresponding products 3p–3s with high efficiency and excellent regioselectivity (*α*/*γ* > 20 : 1) (Scheme 1b). In contrast, vinyl bromide or chloride-containing DFAS 2g–h were not applicable to the reaction due to the formation of some uncertain by-products (Table S2,† entries 5 and 6). The reaction can also be scaled up, as exemplified by the gram-scale synthesis of 3c, with an even higher yield (73%) obtained without loss of regioselectivity. Encouraged by the successful late-stage *O-gem*-difluoroallylation of phenol-containing bioactive molecules, we next turned our attention to the modification of tyrosine in peptides with DFAS 2a (Scheme 2). The representative N-terminal protected linear peptides (5a–5c), including enkephalin^25^ (5a), underwent the *O-gem*-difluoroallylation of tyrosine smoothly. This process exhibited bioorthogonal activity towards unprotected nucleophilic residues, such as tryptophan, histidine, methionine, threonine, *etc.* Furthermore, the bioactive and clinical cyclopeptides, oxytocin (5d) and tyrosine^3^-octreotate (TATE, 5e) with a labile disulfide bond, were well *O*-fluoroalkylated on the tyrosine residue. As the first biochemically described and synthesized cyclic nonapeptide hormone, oxytocin has been called the best-understood neuropeptide.^26^ It has been demonstrated that the subtle modification of oxytocin could lead to significant changes in its activity.^27^ Although research on the modification of oxytocin has lasted for decades,^28^ the well-known fluorine effect is missing in this context due to the lack of a valid fluorination method. To date, the only fluorination research on oxytocin is limited to PET application by using ^18^F-fluoroethylamidation on [Gly-OH^9^] oxytocin through a tedious protection/deprotection procedure.^29^ The successful *O*-fluoroalkylation of oxytocin with DFAS offers an opportunity to explore the fluorine effect on oxytocin, thereby providing the possibility to discover new oxytocin-based bioactive molecules. TATE is a somatostatin agonist used for peptide receptor radionuclide therapy (PRRT) by ligating with a radionuclide chelator, tetraxetan.^30^ Nevertheless, almost all the ligation occurs at the N-terminal of TATE, we could now provide an alternative link position, and the introduction of a fluorine functionality on tyrosine3 may improve the half-live of somatostatin agonist analogues,^31^ thus leading to enhanced activity. We also examined the reaction of DFAS 2a with thiophenols; however, a reversed regioselectivity (*γ*/*α* > 20 : 1) with *gem*-difluoroalkene as the major product was observed (for details, see ESI†3w and 3x). Unfortunately, the cysteine, lysine, and N-terminal residues were inapplicable to the reaction under the current conditions, which will be addressed by developing new fluoroalkylating reagents.

**Scheme 3:**
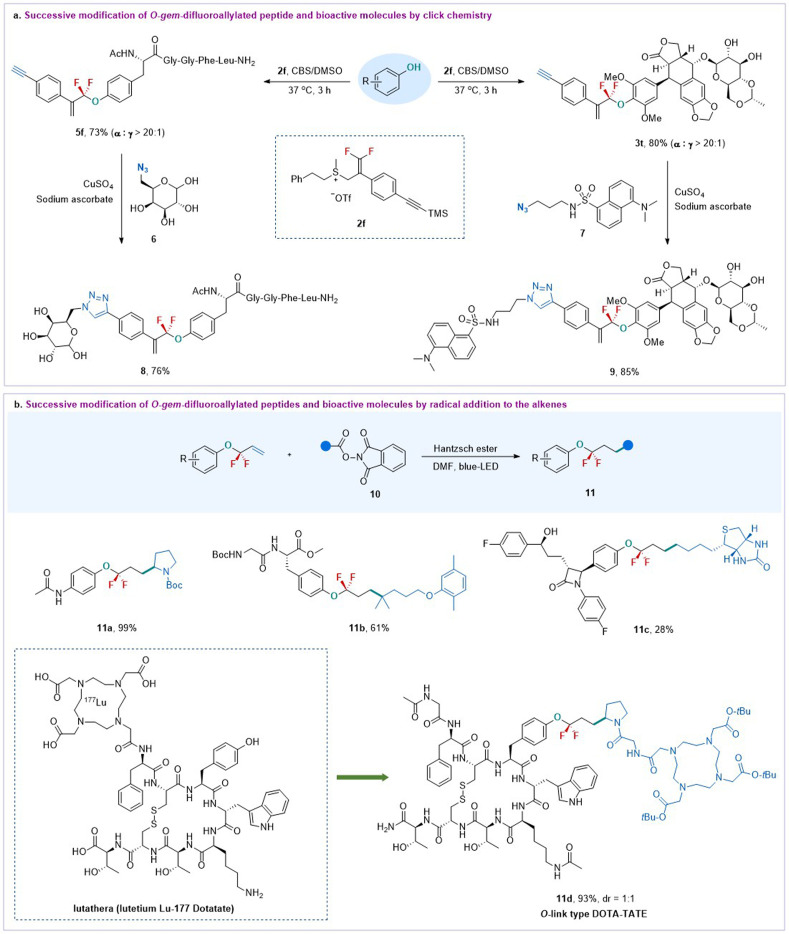
Successive modification of peptides and bioactive molecules from *gem*-difluoroallylated compounds.

Most importantly, the added vinyl and other functional groups from the DFASs can serve as valuable linkers for successive modification (Scheme 3), offering new opportunities for further bioconjugation. For instance, the reaction of enkephalin with an alkynyl group containing DFAS 2f provided *O-gem*-difluoroallylated peptide 5f efficiently, which successively underwent click chemistry^32^ with 6-azido-6-deoxy-d-glucose 6 to afford 8 with high efficiency (Scheme 3a), thereby providing a new route to glycopeptides of great interest in medicinal chemistry and chemical biology. This successive procedure can also be applied to complex bioactive molecules, as exemplified by forming 3t between anti-cancer drug etoposide and 2f, followed by CuAAc chemistry^32^ with azide-containing fluorescent 7. The resulting fluorescently labeled etoposide analogue 8 may have potential applications in cell imaging. It should be mentioned that the reaction of 2f with etoposide provided much higher *α*-regioselectivity than that of 2a, indicating that the steric effect may play a critical role in the regioselectivity. Furthermore, the *gem*-difluoroallyl compounds are versatile synthons for diverse transformations, such as olefin metathesis, dihydroxylation, oxidation, *etc.*^18^ Here, we found that the resulting *gem*-difluoroallylated products could serve as good coupling partners for the radical addition reaction. As shown in Scheme 3b, the reaction of alkyl redox esters, including proline and biotin derivatives, with the resulting *O-gem*-difluoroallylated products in the presence of Hantzsch ester could provide a series of bioconjugates 11a–11c under irradiation of blue light. However, their corresponding nonfluorinated *O*-allylated compounds exhibited much lower reactivity, producing the nonfluorinated product in poor yield (see ESI Section 6.3†), thereby highlighting the unique properties of the CF_2_ group. Notably, this tactic can be efficiently applied to the synthesis of O-link type DOTA-TATE 11d instead of a conventional N-link DOTA-TATE,^30^ offering a new opportunity to evaluate their bioactivities for cancer therapeutics.

## Conclusions

In conclusion, we have developed an efficient *O*-fluoroalkylation method for late-stage phenolic modification of bioactive molecules and peptides. The critical factor for the success of this protocol is the use of the stable yet highly reactive fluoroalkylating reagent, DFASs. The reaction proceeds smoothly in a mild basic aqueous buffer, featuring high efficiency, high functional group tolerance, and excellent regio- and chemo-selectivity; especially, the successive modification strategy significantly expands the chemical space for further functionalizations. A series of carbohydrates, nucleosides, and nucleophilic amino acid residues, including tryptophan, histidine, methionine, and threonine, exhibit bioorthogonal reactivity to the reaction, showing the prospect of the approach in chemical biology. Notably, the unique properties of the CF_2_ group not only render DFASs much more reactive than their non-fluorinated counterparts, but also enable the *gem*-difluoroallyl group to be an active radical receptor for radical addition, adding a new tool to the modification of the biomolecules toolbox. We anticipate that this method will be attractive to medicinal chemistry and chemical biology researchers who wish to modify the biomolecules site-specifically. Perhaps most importantly, this late-stage phenolic difluoroalkylation offers promising opportunities to explore the unique fluorine effect of the CF_2_ group in medicinal chemistry and chemical biology, leading to the discovery of new bioactive molecules.

## Data availability

All experimental data, procedures for data analysis, and pertinent data sets are provided in the ESI.†

## Author contributions

X. Z. conceived and designed the experiments. X. Z. directed the project. M. Z. performed the experiments. J.-X. R., X.-T. F., H.-Y. Z., and Q.-Q. M. prepared some starting materials. M. Z. and X.-P. F. analyzed the data. X. Z. wrote the paper. All authors discussed the results and commented on the manuscript.

## Conflicts of interest

There are no conflicts to declare.

## Supplementary Material

SC-015-D3SC06302J-s001

## References

[cit1] Bhutani P., Joshi G., Raja N., Bachhav N., Rajanna P. K., Bhutani H., Paul A. T., Kumar R. (2021). J. Med. Chem..

[cit2] O'Hagan D. (2008). Chem. Soc. Rev..

[cit3] Remete A. M., Nonn M., Fustero S., Fülöp F., Kiss L. (2018). Tetrahedron.

[cit4] (e) Fishwick C. , Ko-Ferrigno P., and Rabbitts T., Molecular Medicine and Medicinal Chemistry, in Fluorine in Pharmaceutical and Medicinal Chemistry, ed. V. Gouverneur and K. Müller, vol. 6, Imperial College Press, 2012

[cit5] Sanchez C. A., Gadais C., Chaume G., Girard S., Chelain E., Brigaud T. (2021). Org. Lett..

[cit6] Zhou M., Feng Z., Zhang X. (2023). Chem. Commun..

[cit7] Imiołek M., Karunanithy G., Ng W.-L., Baldwin A. J., Gouverneur V., Davis B. G. (2018). J. Am. Chem. Soc..

[cit8] Capone S., Kieltsch I., Flögel O., Lelais G., Togni A., Seebach D. (2008). Helv. Chim. Acta.

[cit9] Wright T. H., Bower B. J., Chalker J. M., Bernardes G. J. L., Wiewiora R., Ng W.-L., Raj R., Faulkner S., Vallée M. R. J., Phanumartwiwath A., Coleman O. D., Thézénas M.-L., Khan M., Galan S. R. G., Lercher L., Schombs M. W., Gerstberger S., Palm-Espling M. E., Baldwin A. J., Kessler B. M., Claridge T. D. W., Mohammed S., Davis B. G. (2016). Science.

[cit10] Coussens L. M., Werb Z. (2002). Nature.

[cit11] Wolfert M. A., Boons G.-J. (2013). Nat. Chem. Biol..

[cit12] Yang Y.-S., Wang C.-C., Chen B.-H., Hou Y.-H., Hung K.-S., Mao Y.-C. (2015). Molecules.

[cit13] Witt K. A., Gillespie T. J., Huber J. D., Egleton R. D., Davis T. P. (2001). Peptides.

[cit14] Cogswell T. J., Dahlén A., Knerr L. (2019). Chem.–Eur. J..

[cit15] (b) Ferstandiy L. , Fluorinated Anesthetics in Chemistry of Organic Fluorine Compounds, Part II, ACS Monograph 187, ACS, Washington, DC, 1995, pp. 1133–1137

[cit16] Dabrowski A., Štabuc B., Lazebnik L. (2018). Gastroenterol. Rev..

[cit17] Doble A. (1996). Neurology.

[cit18] Feng X.-T., Ren J.-X., Gao X., Min Q.-Q., Zhang X. (2022). Angew. Chem., Int. Ed..

[cit19] Müller K., Faeh C., Diederich F. (2007). Science.

[cit20] Ye F., Ge Y., Spannenberg A., Neumann H., Xu L.-W., Beller M. (2021). Nat. Commun..

[cit21] El-Faham A., Albericio F. (2011). Chem. Rev..

[cit22] Lee B., Elston D. M. (2019). J. Am. Acad. Dermatol..

[cit23] Gu J., Zhu N., Li H.-F., Zhang C.-J., Gong Y.-Z., Liao D.-F., Qin L. (2022). Front. Pharmacol.

[cit24] Dawicki-McKenna J. M., Langelier M. F., DeNizio J. E., Riccio A. A., Cao C. D., Karch K. R., McCauley M., Steffen J. D., Black B. E., Pascal J. M. (2015). Mol. Cell.

[cit25] Takahashi A. , Handbook of Hormones Comparative Endocrinology for Basic and Clinical Research, Subchapter 9A – Enkephalin Academic Press, 2 edn, 2021, vol. 1, pp. 91–94

[cit26] Jurek B., Neumann I. D. (2018). Physiol. Rev..

[cit27] Muttenthaler M., Andersson Å., Vetter I., Menon R., Busnelli M., Ragnarsson L., Bergmayr C., Arrowsmith S., Deuis J. R., Chiu H. S., Palpant N. J., O'Brien M., Smith T. J., Wray S., Neumann I. D., p C. W., Lewis R. J., Alewood P. F. (2017). Sci. Signaling.

[cit28] Wiśniewski K. , Design of Oxytocin Analogs, in Cyclic Peptide Design. Methods in Molecular Biology, ed. G. Goetz, Humana, New York, NY. 2019, vol. 2001, pp. 235–271 10.1007/978-1-4939-9504-2_11 31134574

[cit29] Jelinski M., Hamacher K., Coenen H. H. (2002). J. Labelled Compd. Radiopharm..

[cit30] Reubi J. C., Schär J.-C., Waser B., Wenger S., Heppeler A., Schmitt J. S., Mäcke H. R. (2000). Eur. J. Nucl. Med..

[cit31] Basu S., Parghane R. V., Kamaldeep, Chakrabarty S. (2020). Semin. Nucl. Med..

[cit32] Meldal M., Tornøe C. W. (2008). Chem. Rev..

