# Supplementary material for: Late-stage gem-difluoroallylation of phenol in bioactive molecules and peptides with 3,3-difluoroallyl sulfonium salts
Source: Chem Sci. 2024 Jan 15;15(8):2937–45. doi: 10.1039/d3sc06302j (PMC10882445; doi:10.1039/d3sc06302j)

Electronic Supplementary Material (ESI) for Chemical Science.
This journal is © The Royal Society of Chemistry 2024

Supporting Information

Late-Stage *gem*-Difluoroallylation of Phenol in Bioactive Molecules and Peptides with 3,3-Difluoroallyl Sulfonium Salts

Minqi Zhou[1], Jin-Xiu Ren[2], Xiao-Tian Feng[1], Hai-Yang Zhao[2], Xia-Ping Fu[2], Qiao-Qiao Min[2] & Xingang Zhang[1,2]*

[1] College of Chemistry and Henan Institute of Advanced Technology, Zhengzhou University, Zhengzhou 450001, China.

[2] Key Laboratory of Fluorine and Nitrogen Chemistry and Advanced Materials (Chinese Academy of Sciences), Shanghai Institute of Organic Chemistry, University of Chinese Academy of Sciences, Chinese Academy of Sciences, 345 Lingling Road, Shanghai 200032, China.

xgzhang@mail.sioc.ac.cn

Table of Contents

1. Materials and Methods…………………………………………………………………....S2

2. Preparation of 3,3-Difluoroallyl Sulfonium Salts (DFASs) **2**…....………………………………..S3

3. Optimizations of the Reaction Conditions for the *gem*-Difluoroallylation of Protected Tyrosine **1a** with DFAS **2a** S5

4. General Procedures for the *gem*-Difluoroallylation of Phenols **1** with DFASs **2**..............................S9

5. Characterization Data for *gem*-Difluoroallylated Compounds **3** and **5**..S9

6. Successive Modification of Peptides and Bioactive Molecules from Compounds **3** and **5**................S27

7. References...S34

8. Copies of ^{1}H, ^{19}F and ^{13}C NMR Spectra of Compounds **S-3** and **2f**...S35

9. Copies of ^{1}H, ^{19}F, and ^{13}C NMR Spectra of Compounds **3-12**..S41

1. Materials and Methods

General information: ^{1}H NMR and ^{13}C NMR spectra were recorded on Bruker AM400, AM500 or AM600 spectrometers and were calibrated using residual undeuterated solvent ($CHCl_3$ at 7.26 ppm ^{1}H NMR, 77.00 ppm ^{13}C NMR; DMSO-d_6 at 2.50 ppm ^{1}H NMR, 39.52 ppm ^{13}C NMR; CD_3OD at 3.31 ppm ^{1}H NMR, 49.00 ppm ^{13}C NMR). ^{19}F NMR spectra were recorded on a Bruker AM400, AM500 or AM600 spectrometer ($CFCl_3$ was used as the external standard, and the low field is positive). Chemical shifts (δ) are reported in ppm, and coupling constants (*J*) are in Hertz (Hz). The following abbreviations were used to explain multiplicities: s = singlet, d = doublet, t = triplet, q = quartet, m = multiplet, br = broad. The NMR yield was determined by ^{19}F NMR using fluorobenzene as an internal standard before working up the reaction. The electrospray ionization mass spectrometry (ESI-MS) and the subsequent tandem mass spectrometry (ESI-MS/MS) experiments were performed using a Thermo TSQ Quantum AccessTM triple-quadrupole mass spectrometer (Thermo-Fisher Scientific, Waltham, MA, USA).

Materials: Unless otherwise noted, reagents were used as received from commercial sources and used without further purification. Peptides were customized from GenScript and TACHEM. All solvents were not superdry. DFASs were prepared according to literature[1]. 12 W blue LED strips (GreeThink 12V-5050-60; 1 m×12.5 mm×4.4 mm) was purchased from Taobao.com.

2. Preparation of 3,3-Difluoroallyl Sulfonium Salts (DFASs) 2

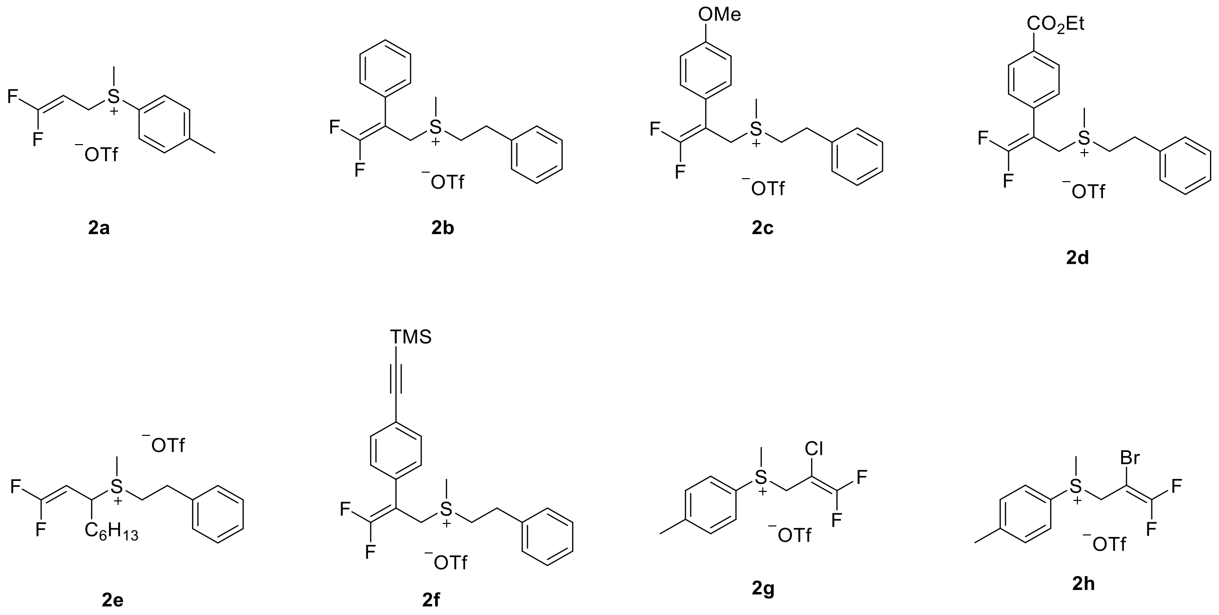

Figure S1. Structures of DFASs **2**

Note: DFASs **2a-2h** are prepared according to the literature[1], and **2a-2e**, and **2g-2h** are known compounds.

Preparation of DFAS 2f.

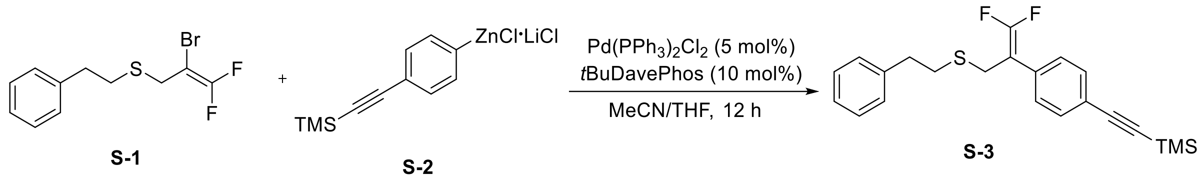

Procedure: To a 100 mL Schlenk tube equipped with a magnetic stir bar were added $Pd(PPh_3)_2Cl_2$ (5 mol%) and *t*-BuDavePhos (10 mol%). The tube was evacuated and backfilled with Ar (3 times). Aryl zinc reagent **S-2** (1.5 equiv, in THF), **S-1** (5.0 mmol, 1.0 equiv), and MeCN (20.0 mL) were added. The resulting reaction mixture was stirred at room temperature for 12 hours. The reaction mixture was quenched with saturated aqueous NH_4Cl solution. The organic layer was separated. The aqueous layer was extracted with ethyl acetate twice. The combined organic layers were washed with brine, dried over anhydrous sodium sulfate, filtered, and concentrated. The residue was purified by reverse-phase flash column chromatography (CH_3CN: H_2O = 9: 1) to afford **S-3** (0.88 g, 46% yield) as a yellow oil. ^1H NMR (400 MHz, $CDCl_3$) δ 7.48 (d, J = 7.6 Hz, 2 H), 7.38 – 7.29 (m, 4 H), 7.27 – 7.21 (m, 1 H), 7.18 (d, J = 7.2 Hz, 2 H), 3.53 (s, 2 H), 2.88 (t, J = 6.8 Hz, 2 H), 2.74 (t, J = 6.8 Hz, 2 H), 0.27 (s, 9 H). ^{19}F NMR (376 MHz, $CDCl_3$) δ -87.4 (d, J = 33.8 Hz, 1 F), -87.6 (d, J = 33.8 Hz, 1 F). ^{13}C NMR (126 MHz, $CDCl_3$) δ

154.5 (dd, J = 295.5 Hz, 291.1 Hz), 140.2, 132.0, 131.8, 131.5, 128.49, 128.46, 126.4, 122.5, 104.6, 95.0, 90.3 (dd, J = 20.3 Hz, 14.0 Hz), 36.0, 33.1, 29.5, -0.1. MS (DART): m/z (%) 387 $(M+H)^+$. HRMS (DART): Calcd. for $C_{22}H_{25}F_2SSi$: 387.1409 $(M+H)^+$; Found: 387.1404 $(M+H)^+$.

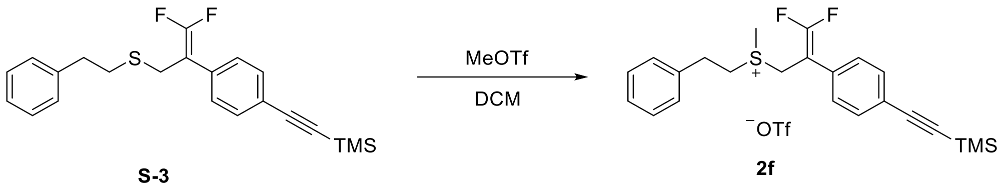

S-3 **2f**

Procedure: To a 100 mL round bottom flask equipped with a magnetic stir bar was added **S-3** (2.3 mmol, 1.0 equiv, 0.5 M in DCM). MeOTf (2.4 mmol, 1.05 equiv) was added dropwise at room temperature. The reaction mixture was stirred at room temperature overnight. Part of the solvent was removed. Ethyl ether was added to the mixture until a large amount of solid was precipitated. The solid was filtered and washed with ethyl ether three times to afford **2f** (0.77g, 61% yield) as a white solid (m.p. 87-90 °C). ^{1}H NMR (400 MHz, $CDCl_3$) δ 7.50 (d, J = 8.4 Hz, 2 H), 7.35 – 7.24 (m, 5 H), 7.18 (d, J = 7.2 Hz, 2 H), 4.64 (d, J = 14.4 Hz, 1 H), 4.42 (d, J = 14.4 Hz, 1 H), 3.76 (t, J = 6.8 Hz, 2 H), 3.14 – 3.02 (m, 2 H), 2.71 (s, 3 H), 0.25 (s, 9 H). ^{19}F NMR (376 MHz, $CDCl_3$) δ -78.4 (d, J = 13.2 Hz, 1 F), -78.5 (s, 3 F), -78.6 (d, J = 13.2 Hz, 1 F). ^{13}C NMR (126 MHz, $CDCl_3$) δ 156.2 (t, J = 299.5 Hz), 135.6, 132.9, 129.4, 128.6, 128.01, 127.99 (t, J = 3.3 Hz), 124.3, 119.2 (t, J = 320.5 Hz), 103.7, 96.6, 84.2 (t, J = 20.3 Hz), 43.7, 40.8 (d, J = 4.4 Hz), 30.8, 22.5, -0.2. MS (ESI): m/z (%) 401 $(M-OTf)^+$. HRMS (ESI): Calcd. for $C_{23}H_{27}F_2SSi$: 401.1565 $(M-OTf)^+$; Found: 401.1560 $(M-OTf)^+$.

3. Optimizations of the Reaction Conditions for the *gem*-Difluoroallylation of Protected Tyrosine 1a with DFAS 2a.

Table S1. Optimization of the Solvents[a]

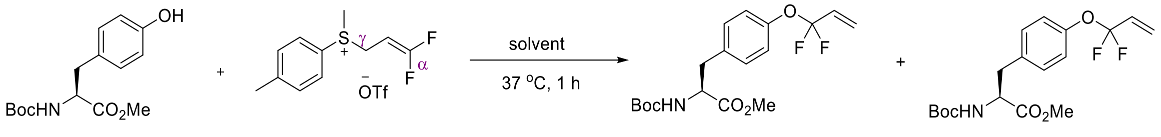

Entry	Solvent	**3a** and **4a**	
		**3a**/ **4a**, yield (%)[b]	α/γ
1	DCM	nd	/
2	DMSO	nd	/
3	DMF	nd	/
4	DMSO + Na_2CO_3 (1.0 equiv)	7/11	1:1.6
5	PBS (pH = 7.6, 0.1 M) / DMSO (1:1, v/v)	36/4	9:1
6	Tris (pH = 8.9, 0.1 M) / DMSO (1:1, v/v)	5.5/3.5	1.6:1
7	CBS (pH = 8.30, 0.1 M) / DMSO (1:1, v/v)	37/--	>20:1
8	CBS (pH = 9.40, 0.1 M) / DMSO (1:1, v/v)	63/--	>20:1
9	CBS (pH = 9.72, 0.1 M) / DMSO (1:1, v/v)	71/--	>20:1
10	CBS (pH = 10.08, 0.1 M) / DMSO (1:1, v/v)	78/--	>20:1
11	CBS (pH = 11.62, 0.1 M) / DMSO (1:1, v/v)	>99 (95)/--	>20:1
12	CBS (pH = 11.62, 0.1 M) / DMF (1:1, v/v)	95/--	>20:1
13	CBS (pH = 11.62, 0.1 M) / DCM (1:1, v/v)	96/--	>20:1
14	CBS (pH = 11.62, 0.1 M) / MeCN (1:1, v/v)	94/--	>20:1
15	CBS (pH = 11.62 , 0.1 M) / acetone (1:1, v/v)	95/--	>20:1
16	CBS (pH = 11.62, 0.1 M) / MeOH (1:1, v/v)	61/--	>20:1

[a]Reaction conditions (unless otherwise specified): **1a** (0.2 mmol, 1.0 equiv), **2a** (0.2 mmol, 1.0 equiv), solvent (4 mL), 37 °C, 1 h. [b]Determined by ^{19}F NMR using fluorobenzene as an internal standard; the number given in parentheses is the isolated yield; nd, not detected.

Table S2. Control Experiments[a]

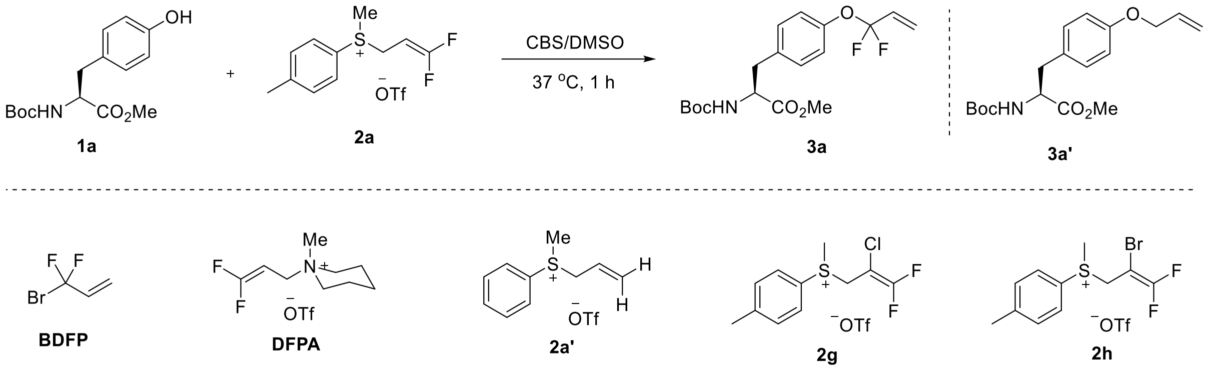

Entry	Conditions	**3a**, Yield (%)[b]
1	Standard conditions	>99
2	**BDFP** instead of **2a**	nd
3	**DFPA** instead of **2a**	25
4	**2a'** instead of **2a**	15[c]
5	**2g** instead of **2a**	25[d] (formation of many uncertain by-products)
6	**2h** instead of **2a**	trace[d]

[a]Reaction conditions (unless otherwise specified): **1a** (0.2 mmol, 1.0 equiv), **2a** (1.0 equiv), CBS (2 mL), DMSO (4 mL), 37 °C, 1 h. [b]Determined by ^{1}H NMR using 1,3,5-trimethoxybenzene as an internal standard; nd, not determined. [c]The yield is for **3a′**. [d]The yield is for the corresponding product.

Table S3. Kinetic Studies of the *gem*-Difluoroallylation of 1a with 2a.

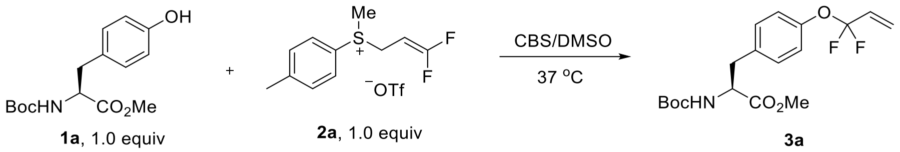

Entry	Reaction time (min)	Yield (%)[b]
1	1	78
2	5	90
3	15	97
4	30	98
5	60	100

[a]Reaction conditions (unless otherwise specified): **1a** (0.2 mmol, 1.0 equiv), **2a** (1.0 equiv), CBS (2 mL), DMSO (4 mL), 37 °C. [b]Determined by ^{19}F NMR using fluorobenzene as an internal standard.

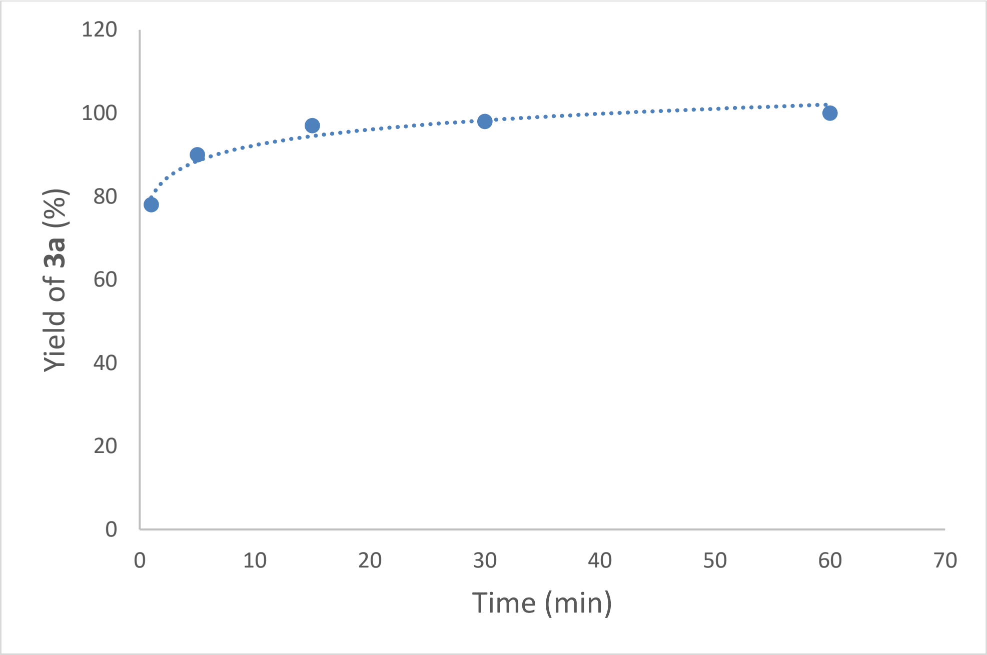

Figure S1. Kinetic Studies of the *gem*-Difluoroallylation of 1a with 2a.

Table S4. Kinetic Studies of the Allylation of 1a with 2a'.

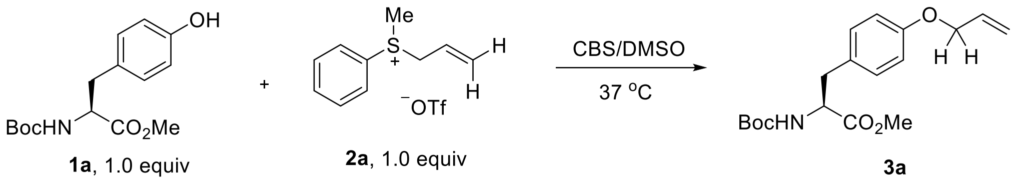

Entry	Reaction time (h)	Yield (%)[b]
1	0.5	12
2	1	15
3	3	16
4	6	14
5	12	17
6	18	18
7	24	15

[a]Reaction conditions (unless otherwise specified): **1a** (0.2 mmol, 1.0 equiv), **2a** (1.0 equiv), CBS (2 mL), DMSO (4 mL), 37 °C. [b]Determined by ^{1}H NMR using 1,3,5-trimethoxybenzene as an internal standard.

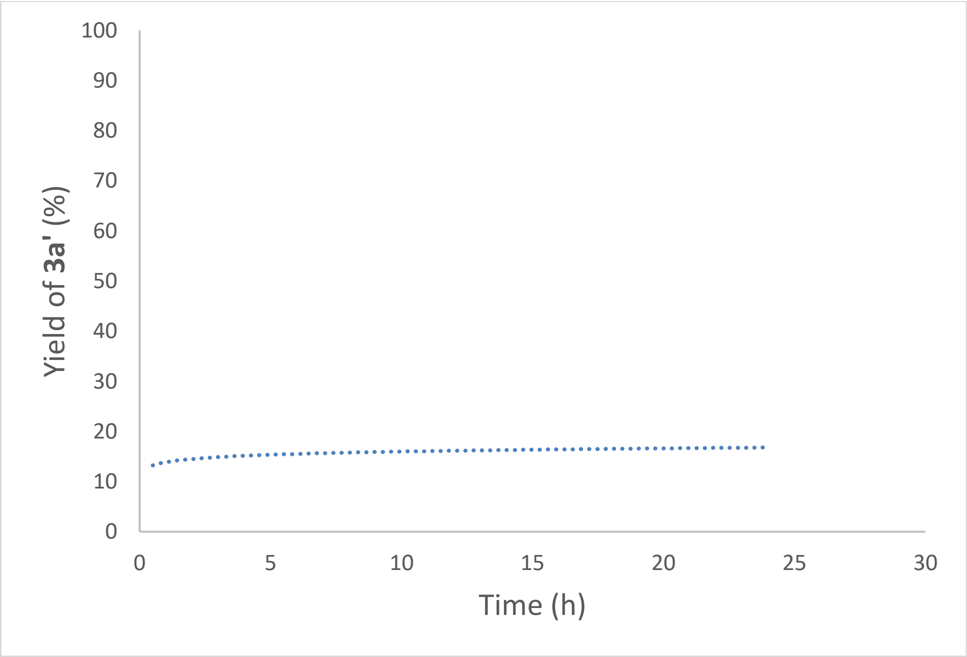

Figure S2. Kinetic Studies of *the* Allylation of 1a with 2a'.

4. General Procedures for the *gem*-Difluoroallylation of Phenols 1 with DFASs 2.

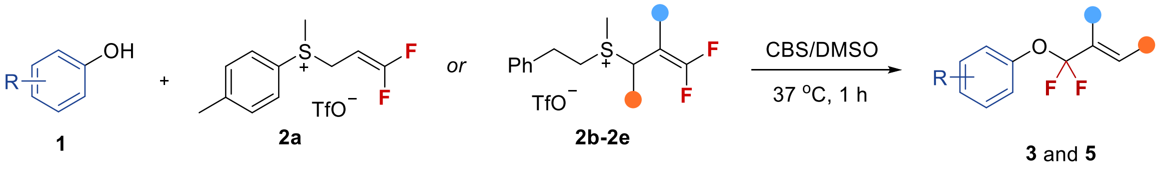

A 25 mL vial equipped with a stirring bar, was added phenol **1** (0.2 mmol, 1.0 equiv) and DFAS **2** (0.2 mmol, 1.0 equiv) under air. DMSO (4.0 mL) and CBS buffer (2.0 mL) were added subsequently. The vial was stirred at 37 °C. After stirring for 1 h, the reaction was cooled to room temperature, and fluorobenzene (1.0 equiv) was added. The yield was determined by ^{19}F NMR before working up. The reaction mixture was then diluted with ethyl acetate and H_2O. The resulting mixture was extracted with ethyl acetate, and the combined organic layers were washed with brine, dried over Na_2SO_4, filtered through a pad of Celite®, and concentrated. The residue was purified with silica gel chromatography to provide the desired product.

5. Characterization Data for *gem*-Difluoroallylated Compounds 3 and 5.

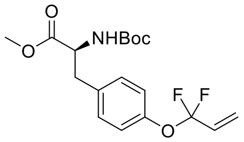

Methyl (*S*)-2-((*tert*-butoxycarbonyl)amino)-3-(4-((1,1-difluoroallyl)oxy)phenyl)propanoate (3a). [Known compound[2]]. Compound **3a** (70.3 mg, 95% yield, α/γ > 20:1) as a colorless oil was purified with silica gel chromatography (Petroleum ether: Ethyl acetate = 8: 1). ^{1}H NMR (400 MHz, $CDCl_3$) δ 7.15 – 7.07 (m, 4 H), 6.10 – 5.98 (m, 1 H), 5.90 (dm, *J* = 17.2 Hz, 1 H), 5.58 (d, *J* = 10.8 Hz, 1 H), 5.01 (d, *J* = 7.6 Hz, 1 H), 4.62 – 4.53 (m, 1 H), 3.70 (s, 3 H), 3.14 – 2.97 (m, 2 H), 1.41 (s, 9 H). ^{19}F NMR (376 MHz, $CDCl_3$) δ -68.8 (d, *J* = 6.4 Hz, 2 F). ^{13}C NMR (126 MHz, $CDCl_3$) δ 172.2, 155.0, 149.3, 133.3, 130.2, 129.3 (t, *J* = 34.0 Hz), 121.8, 121.7 (t, *J* = 6.3 Hz), 120.6 (t, *J* = 260.8 Hz), 80.0, 54.3, 52.2, 37.7, 28.2.

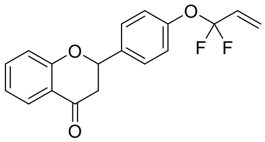

2-(4-((1,1-Difluoroallyl)oxy)phenyl)chroman-4-one (3b). Compound **3b** (57.2 mg, 90% yield, α/γ > 20:1) as a white solid (m.p. 76-78 ºC) was purified with silica gel chromatography (Petroleum ether: Ethyl acetate = 6: 1). ^{1}H NMR (400 MHz, $CDCl_3$) δ 7.94 (dd, *J* = 7.6 Hz, 1.2 Hz, 1 H), 7.55 – 7.45 (m, 3 H), 7.28 (d, *J* = 8.4 Hz, 2 H), 7.10 – 7.03 (m, 2 H), 6.13 – 6.01 (m, 1 H), 5.94 (d, *J* = 17.2 Hz, 1 H), 5.62 (d, *J* = 10.4 Hz, 1 H), 5.48 (dd, *J* = 13.6 Hz, 2.8 Hz, 1 H), 3.08 (dd, *J* = 16.8 Hz, 13.6 Hz, 1 H), 2.89 (dd, *J* = 16.8 Hz, 2.8 Hz, 1 H). ^{19}F NMR (376 MHz, $CDCl_3$) δ -68.8 (d, *J* = 6.8 Hz, 2 F). ^{13}C NMR (126 MHz, $CDCl_3$) δ 191.8, 161.4, 150.5, 136.3, 135.9, 129.2 (t, *J* = 33.3 Hz), 127.3, 127.1, 122.2, 122.0 (t, *J* = 6.4 Hz), 121.7, 120.9, 120.7 (t, *J* = 260.7

Hz), 118.1, 79.0, 44.6. MS (EI): m/z (%) 316 (M^+, 100), 299, 239, 223, 196, 183, 147, 120, 92, 77. HRMS (EI): Calcd. for $C_{18}H_{14}O_3F_2$: 316.0906 $(M)^+$; Found: 316.0904 $(M)^+$.

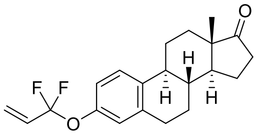

(8*R*,9*S*,13*S*,14*S*)-3-((1,1-Difluoroallyl)oxy)-13-methyl-6,7,8,9,11,12,13,14,15,16-decahydro-17*H*-cyclopenta[*a*]phenanthren-17-one (3c). Compound **3c** (44.1 mg, 64% yield, α/γ > 20:1) as a yellow solid (m.p. 144-147 ºC) was purified with silica gel chromatography (Petroleum ether: Ethyl acetate = 7: 1). ^{1}H NMR (400 MHz, $CDCl_3$) δ 7.25 (d, *J* = 8.4 Hz, 1 H), 6.98 (d, *J* = 8.4 Hz, 1 H), 6.94 (s, 1 H), 6.11 – 5.99 (m, 1 H), 5.91 (d, *J* = 17.2 Hz, 1 H), 5.58 (d, *J* = 10.4 Hz, 1 H), 2.94 – 2.88 (m, 2 H), 2.51 (dd, *J* = 19.2 Hz, 9.2 Hz, 1 H), 2.45 – 2.37 (m, 1 H), 2.32 – 2.23 (m, 1 H), 2.21 – 1.93 (m, 4 H), 1.69 – 1.39 (m, 6 H), 0.91 (s, 3 H). ^{19}F NMR (376 MHz, $CDCl_3$) δ -68.5 (d, *J* = 6.0 Hz, 2 F). ^{13}C NMR (101 MHz, $CDCl_3$) δ 220.7, 148.1, 137.9, 137.0, 129.5 (t, *J* = 34.2 Hz), 126.2, 121.9, 121.6 (t, *J* = 6.6 Hz), 120.6 (t, *J* = 260.0 Hz), 119.1, 50.4, 47.9, 44.1, 38.0, 35.8, 31.5, 29.4, 26.3, 25.7, 21.5, 13.8. MS (DART): m/z (%) 347 $(M+H)^+$. HRMS (DART): Calcd. for $C_{21}H_{25}O_2F_2$: 347.1817 $(M+H)^+$; Found: 347.1814 $(M+H)^+$.

Gram-Scale Synthesis of 3c

To a 250 mL vial equipped with a stirring bar were added Estrone **1c** (5 mmol, 1.0 equiv) and DFAS **2a** (5.25 mmol, 1.05 equiv) under air. DMSO (100 mL) and CBS buffer (0.1 M, 50 mL) were added subsequently. The vial was stirred at 37 ºC. After stirring for 1 h, the reaction was cooled to room temperature, and fluorobenzene (1.0 equiv) was added. The yield was determined by ^{19}F NMR before working up. The reaction mixture was then diluted with ethyl acetate and H_2O. The resulting mixture was extracted with ethyl acetate, and the combined organic layers were washed with brine, dried over Na_2SO_4, filtered through a pad of Celite®, and concentrated. The residue was purified with silica gel chromatography (Petroleum ether: Ethyl acetate = 10: 1) to provide the desired product **3c** (1.25 g, 73%).

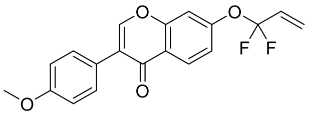

7-((1,1-Difluoroallyl)oxy)-3-(4-methoxyphenyl)-4H-chromen-4-one (3d). Compound **3d** (51.7 mg, 75% yield, α/γ > 20:1) as a white solid (m.p. 116-117 ºC) was purified with silica gel chromatography (Petroleum ether: Ethyl acetate = 5: 1). ^{1}H NMR (400 MHz, $CDCl_3$) δ 8.29 (d, *J* = 8.8 Hz, 1 H), 7.97 (s, 1 H), 7.50 (d, *J* = 8.4 Hz, 2 H), 7.33 (s, 1 H), 7.25 (d, *J* = 8.8 Hz, 1 H), 6.97 (d, *J* = 8.4 Hz, 2 H), 6.15 – 6.03 (m, 1 H), 5.99 (d, *J* = 17.6 Hz, 1 H), 5.67 (d, *J* = 10.4 Hz, 1 H), 3.84 (s, 3 H). ^{19}F NMR (376 MHz, $CDCl_3$) δ -69.4 (d, *J* = 6.0 Hz, 2

F). ^{13}C NMR (101 MHz, $CDCl_3$) δ 175.6, 159.6, 156.6, 154.1, 152.5, 130.0, 128.6 (t, J = 33.0 Hz), 127.8, 125.0, 123.7, 122.5 (t, J = 6.5 Hz), 121.7, 120.8 (t, J = 263.3 Hz), 118.7, 113.9, 109.6, 55.3. MS (DART): m/z (%) 345 $(M+H)^+$. HRMS (DART): Calcd. for $C_{19}H_{15}O_4F_2$: 345.0933 $(M+H)^+$; Found: 345.0929 $(M+H)^+$.

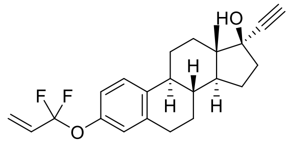

(8*R*,9*S*,13*S*,14*S*,17*R*)-3-((1,1-Difluoroallyl)oxy)-17-ethynyl-13-methyl-7,8,9,11,12,13,14,15,16,17-decahydro-6*H*-cyclopenta[*a*]phenanthren-17-ol (3e). Compound **3e** (56.5 mg, 76% yield, α/γ > 20:1) as a white solid (m.p. 90-96 ºC) was purified with silica gel chromatography (Petroleum ether: Ethyl acetate = 12: 1). ^1H NMR (400 MHz, $CDCl_3$) δ 7.27 (d, J = 8.0 Hz, 1 H), 6.99 (d, J = 8.0 Hz, 1 H), 6.94 (s, 1 H), 6.13 – 6.00 (m, 1 H), 5.93 (d, J = 17.2 Hz, 1 H), 5.59 (d, J = 10.8 Hz, 1 H), 2.91 – 2.84 (m, 2 H), 2.65 – 2.61 (m, 1 H), 2.43 – 2.31 (m, 2 H), 2.31 – 2.22 (m, 1 H), 2.10 – 2.00 (m, 2 H), 1.99 – 1.87 (m, 2 H), 1.87 – 1.67 (m, 3 H), 1.59 – 1.35 (m, 4 H), 0.90 (s, 3 H). ^{19}F NMR (376 MHz, $CDCl_3$) δ -68.5 (d, J = 6.0 Hz, 2 F). ^{13}C NMR (101 MHz, $CDCl_3$) δ 147.9, 138.0, 137.5, 129.5 (t, J = 33.9 Hz), 126.2, 121.9, 121.5 (t, J = 6.5 Hz), 120.6 (t, J = 262.2 Hz), 119.0, 87.4, 79.8, 74.1, 49.4, 47.0, 43.6, 39.0, 38.9, 32.7, 29.5, 27.0, 26.2, 22.8, 12.6. MS (DART): m/z (%) 373 $(M+H)^+$. HRMS (DART): Calcd. for $C_{23}H_{27}O_2F_2$: 373.1974 $(M+H)^+$; Found: 373.1971 $(M+H)^+$.

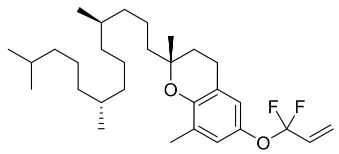

(*R*)-6-((1,1-Difluoroallyl)oxy)-2,8-dimethyl-2-((4*R*,8*R*)-4,8,12-trimethyltridecyl)chromane (3f). DCM instead of DMSO was used. Compound **3f** (72.8 mg, 76% yield, α/γ > 20:1) as a colorless oil was purified with silica gel chromatography (Petroleum ether: Ethyl acetate = 100: 1). ^1H NMR (400 MHz, $CDCl_3$) δ 6.82 (s, 1 H), 6.76 (s, 1 H), 6.11 – 5.99 (m, 1 H), 5.90 (d, J = 17.2 Hz, 1 H), 5.56 (d, J = 10.8 Hz, 1 H), 2.81 – 2.67 (m, 2 H), 2.16 (s, 3 H), 1.86 – 1.70 (m, 2 H), 1.62 – 1.50 (m, 3 H), 1.46 – 1.21 (m, 13 H), 1.19 – 1.01 (m, 8 H), 0.91 – 0.84 (m, 12 H). ^{19}F NMR (376 MHz, $CDCl_3$) δ -68.4 (d, J = 5.3 Hz, 2 F). ^{13}C NMR (126 MHz, $CDCl_3$) δ 149.7, 141.8, 129.7 (t, J = 34.0 Hz), 127.1, 122.2, 121.3 (t, J = 6.2 Hz), 120.8, 120.6 (t, J = 258.2 Hz), 120.1, 76.1, 40.1, 39.4, 37.44, 37.40, 37.3, 32.8, 32.7, 31.0, 28.0, 24.8, 24.4, 24.2, 22.7, 22.6, 22.4, 20.9, 19.74, 19.65, 16.1. MS (DART): m/z (%) 478 $(M)^+$. HRMS (DART): Calcd. for $C_{30}H_{48}O_2F_2$: 478.3617 $(M)^+$; Found: 478.3611 $(M)^+$.

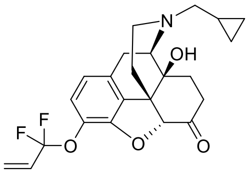

(4*R*,4a*S*,7a*R*,12b*S*)-3-(cyclopropylmethyl)-9-((1,1-difluoroallyl)oxy)-4a-hydroxy-2,3,4,4a,5,6-hexahydro-1*H*-4,12-methanobenzofuro[3,2-*e*]isoquinolin-7(7a*H*)-one (3g). Compound **3g** (48.1 mg, 58% yield, α/γ = 8.7:1) as a yellow oil was purified with silica gel chromatography (Petroleum ether: Ethyl acetate = 1: 1). ^{1}H NMR (400 MHz, $CDCl_3$) δ 6.98 (d, *J* = 8.4 Hz, 1 H), 6.64 (d, *J* = 8.4 Hz, 1 H), 6.17 – 6.05 (m, 1 H), 5.90 (dm, *J* = 17.2 Hz, 1 H), 5.56 (d, *J* = 10.8 Hz, 1 H), 4.69 (s, 1 H), 3.19 (d, *J* = 6.0 Hz, 1 H), 3.07 (d, *J* = 18.4 Hz, 1 H), 3.00 (dd, *J* = 14.4 Hz, 4.4 Hz, 1 H), 2.70 (dd, *J* = 12.0 Hz, 4.4 Hz, 1 H), 2.60 (dd, *J* = 18.4 Hz, 6.0 Hz, 1 H), 2.47 – 2.37 (m, 4 H), 2.30 (dt, *J* = 14.4 Hz, 2.8 Hz, 1 H), 2.11 (td, *J* = 12.0 Hz, 3.6 Hz, 1 H), 1.88 (ddd, *J* = 13.6 Hz, 4.4 Hz, 2.8 Hz, 1 H), 1.61 (td, *J* = 14.0 Hz, 3.2 Hz, 1 H), 1.54 (dd, *J* = 12.8 Hz, 2.4 Hz, 1 H), 0.91 – 0.80 (m, 1 H), 0.58 – 0.52 (m, 2 H), 1.17 – 1.11 (m, 2 H). ^{19}F NMR (376 MHz, $CDCl_3$) δ -68.8 (dd, *J* = 148.5 Hz, 6.8 Hz, 1 F), -69.3 (dd, *J* = 148.9 Hz, 7.5 Hz, 1 F), -84.1 (dm, *J* = 32.7 Hz, 1 F, *γ*-isomer), -85.5 – -85.6 (m, *γ*-isomer). ^{13}C NMR (126 MHz, $CDCl_3$) δ 207.6, 148.7, 131.9, 130.5, 130.1, 128.9 (t, *J* = 33.3 Hz), 124.4, 122.1 (t, *J* = 6.4 Hz), 121.0 (t, *J* = 260.9 Hz), 119.2, 90.6, 69.9, 61.8, 59.1, 50.6, 43.3, 36.0, 31.2, 30.7, 22.8, 9.3, 3.9, 3.8. MS (ESI): m/z (%) 418 $(M+H)^+$. HRMS (ESI): Calcd. for $C_{23}H_{26}O_4NF_2$: 418.1824 $(M+H)^+$; Found: 418.1824 $(M+H)^+$.

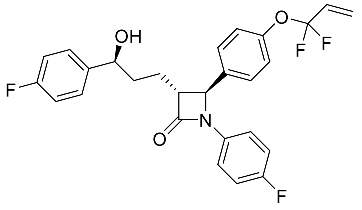

(3*R*,4*S*)-4-(4-((1,1-Difluoroallyl)oxy)phenyl)-1-(4-fluorophenyl)-3-((*S*)-3-(4-fluorophenyl)-3-hydroxypropyl)azetidin-2-one (3h). Compound **3h** (70.5 mg, 73% yield, α/γ > 20:1) as a colorless oil was purified with silica gel chromatography (Petroleum ether: Ethyl acetate = 2: 1). ^{1}H NMR (400 MHz, $CDCl_3$) δ 7.32 – 7.25 (m, 4 H), 7.24 – 7.18 (m, 4 H), 7.00 (d, *J* = 8.8 Hz, 2 H), 6.94 (t, *J* = 8.8 Hz, 2 H), 6.11 – 5.99 (m, 1 H), 5.93 (d, *J* = 17.2 Hz, 1 H), 5.61 (d, *J* = 10.4 Hz, 1 H), 4.73 – 4.68 (m, 1 H), 4.62 (d, *J* = 2.0 Hz, 1 H), 3.12 – 3.03 (m, 1 H), 2.13 (br, 1 H), 2.05 – 1.84 (m, 4 H). ^{19}F NMR (376 MHz, $CDCl_3$) δ -68.9 (d, *J* = 6.0 Hz, 2 F), -114.9 (m, 1 F), -117.8 (m, 1 F). ^{13}C NMR (101 MHz, $CDCl_3$) δ 167.4, 162.1 (d, *J* = 246.9 Hz), 159.0 (d, *J* = 244.7 Hz), 150.4, 140.0 (d, *J* = 3.0 Hz), 134.6, 133.6 (d, *J* = 2.6 Hz), 129.1 (t, *J* = 33.5 Hz), 127.4 (d, *J* = 8.2 Hz), 126.9, 122.5, 122.0 (t, *J* = 6.5 Hz), 120.6 (t, *J* = 261.5 Hz), 118.4 (d, *J* = 7.6 Hz), 115.9 (d, *J* = 22.8 Hz), 115.3 (d, *J* = 21.5 Hz), 73.0, 60.8, 60.3, 36.5, 25.0. MS (DART): m/z (%) 486 $(M+H)^+$. HRMS (DART): Calcd. for $C_{27}H_{24}O_3NF_4$: 486.1687 $(M+H)^+$; Found: 486.1683 $(M+H)^+$.

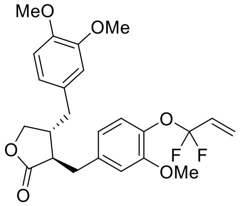

(3*R*,4*R*)-3-(4-((1,1-Difluoroallyl)oxy)-3-methoxybenzyl)-4-(3,4-dimethoxybenzyl)dihydrofuran-2(3*H*)-one (3i). Compound **3i** (74.7 mg, 83% yield, α/γ = 14.3:1) as a white solid (m.p. 115-118 ºC) was purified with silica gel chromatography (Petroleum ether: Ethyl acetate = 1: 1). ^{1}H NMR (400 MHz, $CDCl_3$) δ 7.16 (d, *J* = 8.0 Hz, 1 H), 6.76 – 6.72 (m, 2 H), 6.65 (dd, *J* = 8.0 Hz, 2.0 Hz, 1 H), 6.52 (dd, *J* = 8.0 Hz, 2.0 Hz, 1 H), 6.48 (d, *J* = 2.0 Hz, 1 H), 6.12 – 6.00 (m, 1 H), 5.91 (dm, *J* = 17.2 Hz, 1 H), 5.56 (d, *J* = 10.8 Hz, 1 H), 4.16 (dd, *J* = 9.2 Hz, 7.2 Hz, 1 H), 3.92 – 3.86 (m, 1 H), 3.85 (s, 3 H), 3.82 (s, 3 H), 3.79 (s, 3 H), 3.02 – 2.90 (m, 2 H), 2.66 – 2.44 (m, 4 H). ^{19}F NMR (376 MHz, $CDCl_3$) δ -68.8 (d, *J* = 6.8 Hz, 2 F). ^{13}C NMR (126 MHz, $CDCl_3$) δ 178.4, 152.2, 148.9, 147.7, 137.8, 136.3, 130.2, 129.0 (t, *J* = 33.9 Hz), 123.5, 121.8 (t, *J* = 6.2 Hz), 121.1, 120.8 (t, *J* = 259.8 Hz), 120.4, 113.4, 111.7, 111.2, 71.1, 55.8, 55.73, 55.66, 46.2, 41.0, 37.9, 34.5. MS (DART): m/z (%) 466 $(M+NH_4)^+$. HRMS (DART): Calcd. for $C_{24}H_{27}O_6F_2$: 449.1770 $(M+H)^+$; Found: 449.1766 $(M+H)^+$.

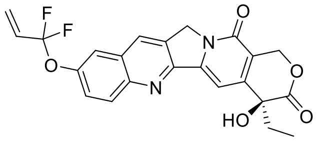

(*S*)-10-((1,1-Difluoroallyl)oxy)-4-ethyl-4-hydroxy-1,12-dihydro-14*H*-pyrano[3',4':6,7]indolizino[1,2-*b*]quinoline-3,14(4*H*)-dione (3j). Compound **3j** (37.0 mg, 42% yield, α/γ > 20:1) as a yellow solid (decomposed at 190 ºC) was purified with silica gel chromatography (Dichloromethane: Methanol = 25: 1). ^{1}H NMR (400 MHz, DMSO-d_6) δ 8.67 (s, 1 H), 8.17 (d, *J* = 9.2 Hz, 1 H), 8.03 – 7.92 (m, 1 H), 7.69 (d, *J* = 7.6 Hz, 1 H), 7.31 (s, 1 H), 6.54 (s, 1 H), 6.39 – 6.20 (m, 1 H), 6.01 (d, *J* = 17.2 Hz, 1 H), 5.79 (d, *J* = 10.8 Hz, 1 H), 5.41 (s, 2 H), 5.24 (s, 2 H), 1.93 – 1.79 (m, 2 H), 0.89 (t, *J* = 7.2 Hz, 3 H). ^{19}F NMR (376 MHz, DMSO-d_6) δ -67.4 (d, *J* = 5.3 Hz, 2 F). ^{13}C NMR (101 MHz, DMSO-d_6) δ 172.4, 156.7, 152.6, 150.0, 148.0, 145.7, 145.2, 131.3, 130.9, 130.5, 128.7 (t, *J* = 32.8 Hz), 128.3, 125.4, 123.6 (t, *J* = 6.4 Hz), 121.1 (t, *J* = 260.3 Hz), 119.2, 118.7, 96.8, 72.4, 65.3, 50.2, 30.3, 7.8. MS (DART): m/z (%) 441 $(M+H)^+$. HRMS (DART): Calcd. for $C_{23}H_{19}O_5N_2F_2$: 441.1257 $(M+H)^+$; Found: 441.1254 $(M+H)^+$.

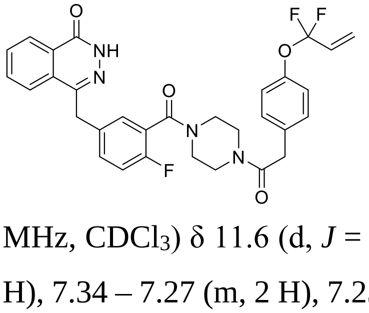

4-(3-(4-(2-(4-((1,1-difluoroallyl)oxy)phenyl)acetyl)piperazine-1-carbonyl)-4-fluorobenzyl)phthalazin-1(2*H*)-one (3k). Compound **3k** (95.5 mg, 83% yield, α/γ > 20:1) as a colorless oil was purified with silica gel chromatography (Dichloromethane: Methanol = 15: 1). ^{1}H NMR (500 MHz, $CDCl_3$) δ 11.6 (d, *J* = 22.0 Hz, 1 H), 8.47 – 8.42 (m, 1 H), 7.77 – 7.71 (m, 2 H), 7.71 – 7.67 (m, 1 H), 7.34 – 7.27 (m, 2 H), 7.23 – 7.19 (m, 2 H), 7.18 – 7.09 (m, 3 H), 7.04 – 6.95 (m, 1 H), 6.07 – 5.96 (m,

1 H), 5.89 (d, J = 17.5 Hz, 1 H), 5.57 (d, J = 11.0 Hz, 1 H), 4.30 – 4.23 (m, 2 H), 3.77 – 3.66 (m, 3 H), 3.62 – 3.49 (m, 3 H), 3.40 (m, 1 H), 3.25 (br, 1 H), 3.11 (br, 1 H). ^{19}F NMR (471 MHz, $CDCl_3$) δ -68.6 – -68.7 (m, 2 F), -117.8 (m, 1 F). ^{13}C NMR (126 MHz, $CDCl_3$) δ 169.4 (d, J = 20.5 Hz), 165.0 (d, J = 25.8 Hz), 160.9, 157.8 (d, J = 6.8 Hz), 155.8 (d, J = 7.1 Hz), 149.1 (d, J = 6.7 Hz), 145.4 (d, J = 4.8 Hz), 134.4, 133.5, 131.7, 131.7 – 131.5 (m), 131.5, 130.2, 129.5, 129.39, 129.36, 129.2 (d, J = 3.7 Hz), 129.1 – 129.0 (m), 128.8, 128.1, 127.0, 124.9, 123.4 (dd, J = 17.9 Hz, 7.3 Hz), 122.1, 121.9, 121.8 (t, J = 6.6 Hz), 120.5 (t, J = 259.9 Hz), 116.0 (dd, J = 21.8 Hz, 12.0 Hz), 46.6 (d, J = 20.9 Hz), 45.8 (d, J = 63.3 Hz), 41.8, 41.5 (d, J = 50.3 Hz), 40.7, 40.0 (d, J = 24.1 Hz), 37.5 (d, J = 9.6 Hz). MS (ESI): m/z (%) 577 $(M+H)^+$. HRMS (ESI): Calcd. for $C_{31}H_{28}O_4N_4F_3$: 577.2057 $(M+H)^+$; Found: 577.2050 $(M+H)^+$.

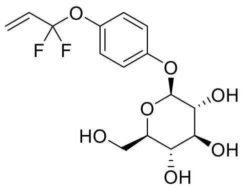

(2*S*,3*R*,4*S*,5*S*,6*R*)-2-(4-((1,1-difluoroallyl)oxy)phenoxy)-6-(hydroxymethyl)tetrahydro-2*H*-pyran-3,4,5-triol (3l). Compound **3l** (37.0 mg, 53% yield, 77% determined by ^{19}F NMR, α/γ > 20:1) as a yellow oil was purified with reverse-phase flash column chromatography (CH_3CN: H_2O = 7: 3). ^{1}H NMR (400 MHz, DMSO-d_6) δ 7.14 (d, J = 8.8 Hz, 2 H), 7.05 (d, J = 8.8 Hz, 2 H), 6.28 – 6.15 (m, 1 H), 5.89 (d, J = 17.2 Hz, 1 H), 5.71 (d, J = 10.8 Hz, 1 H), 5.32 (br, 1 H), 5.08 (br, 1 H), 5.03 (br, 1 H), 4.83 (d, J = 6.8 Hz, 1 H), 4.57 (br, 1 H), 3.69 (d, J = 11.2 Hz, 1 H), 3.50 – 3.42 (m, 1 H), 3.31 – 3.20 (m, 3 H), 3.20 – 3.12 (m, 1 H). ^{19}F NMR (376 MHz, DMSO-d_6) δ -67.3 (d, J = 6.4 Hz, 2 F). ^{13}C NMR (126 MHz, DMSO-d_6) δ 155.3, 143.8, 129.1 (t, J = 33.6 Hz), 123.1, 123.0 (t, J = 6.6 Hz), 120.8 (t, J = 257.2 Hz), 117.1, 100.7, 77.1, 76.6, 73.3, 69.7, 60.7. MS (DART): m/z (%) 366 $(M+NH_4)^+$. HRMS (DART): Calcd. for $C_{15}H_{22}O_7NF_2$: 366.1359 $(M+NH_4)^+$; Found: 366.1355 $(M+NH_4)^+$.

Representative Procedure for the Preparation of Protected Carbohydrates.

After the corresponding phenol-containing carbohydrate reacted with DFAS **2a** for 1 h, the reaction mixture was then diluted with ethyl acetate and H_2O. The resulting mixture was extracted with ethyl acetate, the combined organic layers were washed with brine, dried over Na_2SO_4, filtered through a pad of Celite® and concentrated. The resulting crude *gem*-difluoroallylated product was reacted with Ac_2O (5 mmol, 25 equiv) and pyridine (5 mmol, 25 equiv) for 6 h at room temperature. The reation mixture was concentrated under vacuum. The residue was purified with silica gel chromatography to provide the desired product **3l'-3o'**.

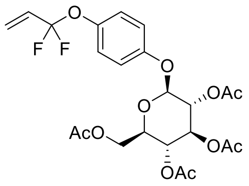

(2*R*,3*R*,4*S*,5*R*,6*S*)-2-(Acetoxymethyl)-6-(4-((1,1-difluoroallyl)oxy)phenoxy)tetrahydro-2*H*-pyran-3,4,5-triyl triacetate (3l′). Compound **3l′** (75.5 mg, 72% yield, α/γ > 20:1) as a white solid (m.p. 99-101 ºC) was purified with silica gel chromatography (Petroleum ether: Ethyl acetate = 1: 1). ^{1}H NMR (400 MHz, $CDCl_3$) δ 7.12 (d, *J* = 8.8 Hz, 2 H), 6.96 (d, *J* = 8.8 Hz, 2 H), 6.10 – 5.97 (m, 1 H), 5.90 (d, *J* = 17.2 Hz, 1 H), 5.58 (d, *J* = 10.8 Hz, 1 H), 5.32 – 5.22 (m, 2 H), 5.16 (t, *J* = 9.6 Hz, 1 H), 5.04 (d, *J* = 7.6 Hz, 1 H), 4.28 (dd, *J* = 12.4 Hz, 5.2 Hz, 1 H), 4.16 (dd, *J* = 12.4 Hz, 2.0 Hz, 1 H), 3.87 – 3.81 (m, 1 H), 2.07 (s, 3 H), 2.06 (s, 3 H), 2.04 (s, 3 H), 2.03 (s, 3 H). ^{19}F NMR (376 MHz, $CDCl_3$) δ -69.1 (d, *J* = 6.4 Hz, 2 F). ^{13}C NMR (101 MHz, $CDCl_3$) δ 170.5, 170.2, 169.4, 169.3, 154.3, 145.7, 129.2 (t, *J* = 33.8 Hz), 123.2, 121.8 (t, *J* = 6.8 Hz), 120.5 (t, *J* = 260.3 Hz), 117.8, 99.3, 72.6, 72.0, 71.1, 68.2, 61.9, 20.64, 20.61, 20.58, 20.56. MS (DART): m/z (%) 534 $(M+NH_4)^+$. HRMS (DART): Calcd. for $C_{23}H_{30}O_{11}NF_2$: 534.1781 $(M+NH_4)^+$; Found: 534.1770 $(M+NH_4)^+$.

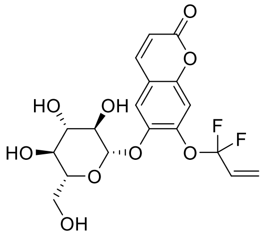

7-((1,1-difluoroallyl)oxy)-6-(((2*S*,3*R*,4*S*,5*S*,6*R*)-3,4,5-trihydroxy-6-(hydroxymethyl)tetrahydro-2*H*-pyran-2-yl)oxy)-2*H*-chromen-2-one (3m). Compound **3m** (17.9 mg, 21% yield, 55% yield determined by ^{19}F NMR, α/γ > 20:1) as a yellow oil was purified with reverse-phase flash column chromatography (CH_3CN: H_2O = 3: 7). ^{1}H NMR (400 MHz, CD_3OD) δ 7.93 (d, *J* = 9.6 Hz, 1 H), 7.54 (s, 1 H), 7.28 (s, 1 H), 6.41 (d, *J* = 9.6 Hz, 1 H), 6.28 – 6.16 (m, 1 H), 5.99 (dm, *J* = 17.2 Hz, 1 H), 5.66 (d, *J* = 10.8 Hz, 1 H), 4.99 (d, *J* = 7.2 Hz, 1 H), 3.92 (dd, *J* = 12.0 Hz, 2.0 Hz, 1 H), 3.70 (dd, *J* = 12.0 Hz, 6.0 Hz, 1 H), 3.54 – 3.45 (m, 3 H), 3.42 – 3.36 (m, 1 H). ^{19}F NMR (376 MHz, CD_3OD) δ -69.8 (d, *J* = 6.4 Hz, 2 F). ^{13}C NMR (126 MHz, DMSO-d_6) δ 159.8, 148.1, 146.3, 143.7, 141.6, 128.4, (t, *J* = 32.8 Hz), 124.1 (t, *J* = 6.6 Hz), 121.3 (t, *J* = 260.2 Hz), 116.8, 116.2, 115.3, 110.9, 100.9, 77.2, 76.9, 73.2, 69.6, 60.6. MS (DART): m/z (%) 417 $(M+H)^+$. HRMS (DART): Calcd. for $C_{18}H_{19}O_9F_2$: 417.0992 $(M+H)^+$; Found: 417.0988 $(M+H)^+$.

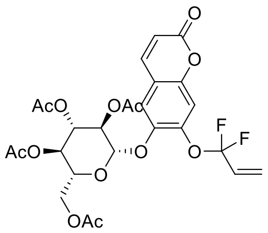

(2*R*,3*R*,4*S*,5*R*,6*S*)-2-(Acetoxymethyl)-6-((7-((1,1-difluoroallyl)oxy)-2-oxo-2*H*-chromen-6-yl)oxy)tetrahydro-2*H*-pyran-3,4,5-triyl triacetate (3m′). Compound **3m′** (65.0 mg, 56% yield, α/γ > 20:1) as a white solid (m.p. 128-131 ºC) was purified with silica gel chromatography (Petroleum ether: Ethyl acetate = 1: 1). ^{1}H NMR (400 MHz, $CDCl_3$) δ 7.61 (d, *J* = 9.6 Hz, 1 H), 7.27 (s, 1 H), 7.24

(s, 1 H), 6.39 (d, J = 9.6 Hz, 1 H), 6.11 – 5.98 (m, 1 H), 5.93 (d, J = 17.2 Hz, 1 H), 5.63 (d, J = 10.4 Hz, 1 H), 5.33 – 5.23 (m, 2 H), 5.20 – 5.12 (m, 1 H), 5.04 (d, J = 6.8 Hz, 1 H), 4.26 (dd, J = 12.4 Hz, 4.8 Hz, 1 H), 4.22 – 4.15 (m, 1 H), 3.89 – 3.83 (m, 1 H), 2.05 (s, 3 H), 2.02 (s, 3 H), 2.01 (m, 6 H). ^{19}F NMR (376 MHz, $CDCl_3$) δ -68.5 (dd, J = 149.3 Hz, 4.9 Hz, 1 F), -70.0 (dd, J = 149.3 Hz, 7.5 Hz, 1 F). ^{13}C NMR (126 MHz, $CDCl_3$) δ 170.3, 170.1, 169.3, 169.1, 160.0, 149.9, 145.5, 143.1, 142.3, 128.1 (t, J = 32.5 Hz), 123.1 (t, J = 6.3 Hz), 120.9 (t, J = 266.0 Hz), 116.7, 116.4, 116.3, 112.0, 99.8, 72.4, 72.1, 70.8, 68.1, 61.7, 20.6, 20.5 (3C). MS (DART): m/z (%) 585 $(M+H)^+$. HRMS (DART): Calcd. for $C_{26}H_{27}O_{13}F_2$: 585.1414 $(M+H)^+$; Found: 585.1406 $(M+H)^+$.

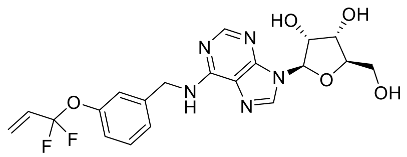

(2*R*,3*R*,4*S*,5*R*)-2-(6-((3-((1,1-difluoroallyl)oxy)benzyl)amino)-9*H*-purin-9-yl)-5-(hydroxymethyl)tetrahydrofuran-3,4-diol (3n). Compound **3n** (42.4 mg, 47% yield, 74% determined by ^{19}F NMR, α/γ > 20:1) as a colorless oil was purified with reverse-phase flash column chromatography (CH_3CN: H_2O = 5: 5). ^{1}H NMR (400 MHz, $CDCl_3$) δ 8.10 (s, 1 H), 7.75 (s, 1 H), 7.20 (t, J = 7.6 Hz, 1 H), 7.15 (s, 1 H), 7.11 (d, J = 7.6 Hz, 1 H), 7.06 (d, J = 7.6 Hz, 1 H), 6.86 – 6.75 (m, 1 H), 6.04 – 5.91 (m, 1 H), 5.84 (dm, J = 17.2 Hz, 1 H), 5.74 (d, J = 6.4 Hz, 1 H), 5.51 (d, J = 10.4 Hz, 1 H), 4.92 – 4.71 (m, 3 H), 4.65 – 4.50 (m, 1 H), 4.37 – 4.30 (m, 1 H), 4.13 (s, 1 H), 3.79 (d, J = 12.4 Hz, 1 H), 3.59 (d, J = 12.0 Hz, 1 H). ^{19}F NMR (376 MHz, $CDCl_3$) δ -68.5 (d, J = 6.0 Hz, 2 F). ^{13}C NMR (126 MHz, $CDCl_3$) δ 154.5, 152.4, 150.4, 147.2, 140.2, 139.7, 129.6, 129.1 (t, J = 33.5 Hz), 124.6, 121.9 (t, J = 6.4 Hz), 121.0, 120.9, 120.6 (t, J = 260.4 Hz), 120.4, 90.7, 87.3, 73.9, 72.2, 62.8, 43.9. MS (DART): m/z (%) 450 $(M+H)^+$. HRMS (DART): Calcd. for $C_{20}H_{22}O_5N_5F_2$: 450.1584 $(M+H)^+$; Found: 450.1577 $(M+H)^+$.

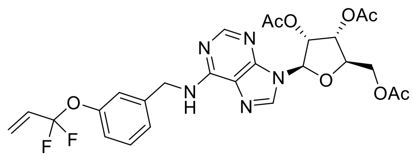

(2*R*,3*R*,4*R*,5*R*)-2-(acetoxymethyl)-5-(6-((3-((1,1-difluoroallyl)oxy)benzyl)amino)-9*H*-purin-9-yl)tetrahydrofuran-3,4-diyl diacetate (3n′). Compound **3n′** (83.1 mg, 72% yield, α/γ > 20:1) as a yellow oil was purified with silica gel chromatography (Petroleum ether: Ethyl acetate = 1: 3). ^{1}H NMR (400 MHz, $CDCl_3$) δ 8.38 (s, 1 H), 7.85 (s, 1 H), 7.29 (t, J = 8.0 Hz, 1H), 7.24 – 7.18 (m, 2 H), 7.11 (d, J = 8.4 Hz, 1 H), 6.53 (br, 1 H), 6.16 (d, J = 5.2 Hz, 1 H), 6.07 – 5.96 (m, 1 H), 5.94 – 5.84 (m, 2 H), 5.69 – 5.64 (m, 1 H), 5.56 (d, J = 10.4 Hz, 1 H), 4.86 (br, 2 H), 4.46 – 4.40 (m, 2 H), 4.39 – 4.32 (m, 1 H), 2.13 (s, 3 H), 2.09 (s, 3 H), 2.06 (s, 3 H). ^{19}F NMR (376 MHz, $CDCl_3$) δ -68.6 (d, J = 6.4 Hz, 2 F). ^{13}C NMR (151 MHz, $CDCl_3$) δ 170.3, 169.6, 169.3, 154.6, 153.4, 150.5, 140.2, 138.2, 129.6, 129.3 (t, J

= 33.5 Hz), 124.6, 121.8 (t, *J* = 6.6 Hz), 121.0, 120.7, 120.6 (t, *J* = 259.4 Hz), 120.1, 86.1, 80.2, 73.1, 70.6, 63.1, 43.9, 20.7, 20.5, 20.3. MS (DART): m/z (%) 576 $(M+H)^+$. HRMS (DART): Calcd. for $C_{26}H_{28}O_8N_5F_2$: 576.1900 $(M+H)^+$; Found: 576.1886 $(M+H)^+$.

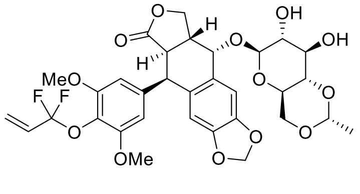

(5*R*,5a*R*,8a*R*,9*S*)-5-(4-((1,1-difluoroallyl)oxy)-3,5-dimethoxyphenyl)-9-(((2*R*,4a*R*,6*R*,7*R*,8*R*,8a*S*)-7,8-dihydroxy-2-methylhexahydropyrano[3,2-d][1,3]dioxin-6-yl)oxy)-5,8,8a,9-tetrahydrofuro[3',4':6,7]naphtho[2,3-d][1,3]dioxol-6(5a*H*)-one (3o). Compound **3o** (44.8 mg, 34% yield, 71% determined by ^{19}F NMR, α/γ = 4.0:1) as a white solid was purified with reverse-phase flash column chromatography (CH_3CN: H_2O = 6: 4). ^1H NMR (600 MHz, DMSO-d_6) δ 7.28 (s, *γ*-isomer), 7.03 (s, 1 H), 7.02 (s, *γ*-isomer), 6.70 (s, *γ*-isomer), 6.57 (s, *γ*-isomer), 6.55 (s, 1 H), 6.33 (s, 2 H), 6.28 (s, *γ*-isomer), 6.13 – 6.03 (m, 1 H), 6.03 – 6.00 (m, 2 H), 5.80 (dm, *J* = 16.8 Hz, 1 H), 5.58 (d, *J* = 10.8 Hz, 1 H), 5.10 (d, *J* = 5.4 Hz, *γ*-isomer), 4.98 (d, *J* = 3.6 Hz, 1 H), 4.91 (d, *J* = 4.8 Hz, 1 H), 4.86 (d, *J* = 4.8 Hz, 1 H), 4.76 – 4.72 (m, 1 H), 4.61 – 4.56 (m, 2 H), 4.34 – 4.26 (m, 2 H), 4.17 (dd, *J* = 9.6 Hz, 3.6 Hz, *γ*-isomer), 4.09 (dd, *J* = 10.2 Hz, 4.8 Hz, 1 H), 3.95 (dd, *J* = 9.6 Hz, 4.2 Hz, *γ*-isomer), 3.66 (s, 6 H), 3.52 (t, *J* = 9.6 Hz, 1 H), 3.47 (t, *J* = 10.2 Hz, *γ*-isomer), 3.42 – 3.33 (m, 2 H), 3.28 – 3.23 (m, 1 H), 3.20 (d, *J* = 9.0 Hz, 1 H), 3.16 – 3.10 (m, 1 H), 2.94 – 2.86 (m, 1 H), 1.27 (d, *J* = 4.8 Hz, 3 H), 1.24 (d, *J* = 5.4 Hz, *γ*-isomer). ^{19}F NMR (565 MHz, DMSO-d_6) δ -65.9 (dd, *J* = 148.6 Hz, 7.3 Hz, 1 F), -66.1 (dd, *J* = 148.6 Hz, 6.8 Hz, 1 F), -86.0 (d, *J* = 37.9 Hz, *γ*-isomer), -87.5 (dd, *J* = 37.9 Hz, 26.0 Hz, *γ*-isomer). ^{13}C NMR (151 MHz, DMSO-d_6) δ 173.9, 153.2 (*γ*-isomer), 152.4, 152.0 (*γ*-isomer), 147.53, 147.47 (*γ*-isomer), 146.1, 140.0 (*γ*-isomer), 138.9, 131.7, 128.9 (t, *J* = 33.7 Hz), 128.7, 126.6 (*γ*-isomer), 121.3 (t, *J* = 6.2 Hz), 120.9 (t, *J* = 258.5 Hz), 109.6, 109.3, 108.4 (*γ*-isomer), 107.9, 106.6 (*γ*-isomer), 105.3, 102.2 (*γ*-isomer), 101.3, 100.9, 100.5 (*γ*-isomer), 98.3, 98.2 (*γ*-isomer), 79.9, 79.8 (*γ*-isomer), 78.7, 74.3, 72.70 (*γ*-isomer), 72.67, 71.63 (*γ*-isomer), 71.58, 67.3, 67.2 (*γ*-isomer), 67.11, 67.08 (*γ*-isomer), 65.55, 65.46 (*γ*-isomer), 55.9 (*γ*-isomer), 55.8, 42.9, 42.3 (*γ*-isomer), 38.0 (*γ*-isomer), 37.2, 19.8. MS (DART): m/z (%) 682 $(M+NH_4)^+$. HRMS (DART): Calcd. for $C_{32}H_{38}O_{13}NF_2$: 682.2306 $(M+NH_4)^+$; Found: 682.2301 $(M+NH_4)^+$.

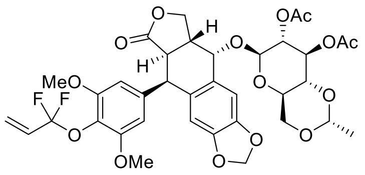

(2*R*,4a*R*,6*R*,7*R*,8*S*,8a*R*)-6-(((5*S*,5a*R*,8a*R*,9*R*)-9-(4-((1,1-Difluoroallyl)oxy)-3,5-dimethoxyphenyl)-8-oxo-5,5a,6,8,8a,9-hexahydrofuro[3',4':6,7]naphtho[2,3-*d*][1,3]dioxol-5-yl)oxy)-2-methylhexahydropyrano[3,2-*d*][1,3]dioxine-7,8-diyl diacetate (3o′). Compound **3o′** (97.7 mg, 65% yield, α/γ = 4.0:1) as a colorless oil was purified with silica gel chromatography (Petroleum ether: Ethyl acetate = 1: 1). ^{1}H NMR (400 MHz, $CDCl_3$) δ 6.76 (s, 1 H), 6.52 (s, 1 H), 6.24 (s, 2 H), 6.09 – 5.94 (m, 3 H), 5.86 (d, *J* = 17.6 Hz, 1 H), 5.49 (d, *J* = 10.8 Hz, 1 H), 5.19 (t, *J* = 9.6 Hz, 1 H), 4.89 (t, *J* = 8.0 Hz, 1 H), 4.83 (d, *J* = 3.2 Hz, 1 H), 4.79 (d, *J* = 8.0 Hz, 1 H), 4.67 (q, *J* = 4.8 Hz, 1 H), 4.59 – 4.54 (m, 1 H), 4.41 – 4.33 (m, 1 H), 4.24 – 4.14 (m, 2 H), 3.67 (s, 6 H), 3.56 (t, *J* = 10.4 Hz, 1 H), 3.45 (t, *J* = 9.6 Hz, 1 H), 3.40 – 3.35 (m, 1 H), 3.18 – 3.12 (m, 1 H), 2.89 – 2.77 (m, 1 H), 2.03 (s, 3 H), 1.81 (s, 3 H), 1.32 (d, *J* = 4.8 Hz, 3 H). ^{19}F NMR (376 MHz, $CDCl_3$) δ -68.4 (s, 2 F). ^{13}C NMR (126 MHz, $CDCl_3$) δ 174.4, 170.2, 169.3, 153.3, 148.7, 147.0, 137.9, 132.2, 129.1 (t, *J* = 33.9 Hz), 128.1, 127.3, 121.4 (t, *J* = 6.2 Hz), 121.2 (t, *J* = 260.8 Hz), 110.7, 108.9, 107.8, 101.6, 100.3, 99.7, 77.6, 74.5, 72.0, 71.4, 67.8, 67.6, 66.3, 56.2, 43.8, 40.9, 37.4, 20.7, 20.3, 20.1. MS (DART): m/z (%) 766 $(M+NH_4)^+$. HRMS (DART): Calcd. for $C_{36}H_{42}O_{15}NF_2$: 766.2517 $(M+NH_4)^+$; Found: 766.2508 $(M+NH_4)^+$.

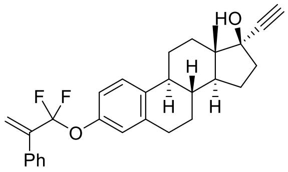

(8*R*,9*S*,13*S*,14*S*,17*R*)-3-((1,1-Difluoro-2-phenylallyl)oxy)-17-ethynyl-13-methyl-7,8,9,11,12,13,14,15,16,17-decahydro-6*H*-cyclopenta[*a*]phenanthren-17-ol (3p). Compound **3p** (86.1 mg, 96% yield, α/γ > 20:1) as a colorless oil was purified with silica gel chromatography (Petroleum ether: Ethyl acetate = 10: 1). ^{1}H NMR (400 MHz, $CDCl_3$) δ 7.62 – 7.57 (m, 2 H), 7.44 – 7.36 (m, 3 H), 7.28 (d, *J* = 8.4 Hz, 1 H), 7.02 (d, *J* = 8.4 Hz, 1 H), 6.95 (s, 1 H), 6.02 (s, 1 H), 5.74 (s, 1 H), 2.91 – 2.84 (m, 2 H), 2.63 (s, 1 H), 2.44 – 2.33 (m, 2 H), 2.32 – 2.23 (m, 1 H), 2.12 – 2.02 (m, 2 H), 2.00 – 1.88 (m, 2 H), 1.88 – 1.69 (m, 3 H), 1.60 – 1.35 (m, 4 H), 0.92 (s, 3 H). ^{19}F NMR (376 MHz, $CDCl_3$) δ -67.4 (s, 2 F). ^{13}C NMR (126 MHz, $CDCl_3$) δ 148.0, 141.7 (t, *J* = 29.7 Hz), 138.0, 137.5, 135.3, 128.4, 128.3, 127.7, 126.2, 121.9, 121.5 (t, *J* = 264.0 Hz), 119.4 (t, *J* = 5.4 Hz), 119.0, 87.4, 79.8, 74.1, 49.4, 47.0, 43.6, 39.0, 38.9, 32.7, 29.5, 27.0, 26.2, 22.8, 12.6. MS (DART): m/z (%) 466 $(M+NH_4)^+$. HRMS (DART): Calcd. for $C_{29}H_{34}O_2NF_2$: 466.2552 $(M+NH_4)^+$; Found: 466.2548 $(M+NH_4)^+$.

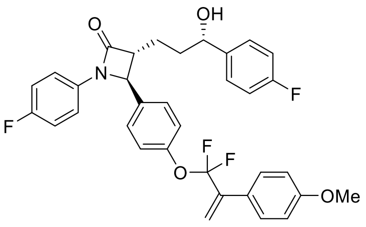

(3*R*,4*S*)-4-(4-((1,1-difluoro-2-(4-methoxyphenyl)allyl)oxy)phenyl)-1-(4-fluorophenyl)-3-((*S*)-3-(4-fluorophenyl)-3-hydroxypropyl)azetidin-2-one (3q). Compound **3q** (108.9 mg, 92% yield, α/γ > 20:1) as a colorless oil was purified with silica gel chromatography (Petroleum ether: Ethyl acetate = 2: 1). ^{1}H NMR (400 MHz, $CDCl_3$) δ 7.47 (d, *J* = 8.8 Hz, 2 H), 7.31 – 7.25 (m, 4 H), 7.24 – 7.18 (m, 4 H), 7.00 (t, *J* = 8.8 Hz, 2 H), 6.96 – 6.87 (m, 4 H), 5.92 (s, 1 H), 5.66 (s, 1 H), 4.69 (t, *J* = 6.0 Hz, 1 H), 4.62 (d, *J* = 2.4 Hz, 1 H), 3.81 (s, 3 H), 3.10 – 3.04 (m, 1 H), 2.77 (br, 1 H), 2.02 – 1.84 (m, 4 H). ^{19}F NMR (376 MHz, $CDCl_3$) δ -67.6 (s, 2 F), -114.9 (m, 1 F), -117.7 (m, 1 F). ^{13}C NMR (126 MHz, $CDCl_3$) δ 167.4, 162.1 (d, *J* = 245.7 Hz), 159.8, 159.0 (d, *J* = 244.2 Hz), 150.5, 140.6 (t, *J* = 29.0 Hz), 140.0 (d, *J* = 2.9 Hz), 134.6, 133.6 (d, *J* = 2.8 Hz), 127.4, 127.3 (d, *J* = 8.1 Hz), 126.8, 122.5, 121.7 (t, *J* = 265.1 Hz), 118.3 – 118.2 (m), 118.3 (d, *J* = 7.4 Hz), 115.8 (d, *J* = 22.4 Hz), 115.3 (d, *J* = 21.2 Hz), 113.7, 72.9, 60.7, 60.3, 55.2, 36.5, 24.9. MS (ESI): m/z (%) 592 $(M+H)^+$. HRMS (ESI): Calcd. for $C_{34}H_{30}O_4NF_4$: 592.2105 $(M+H)^+$; Found: 592.2098 $(M+H)^+$.

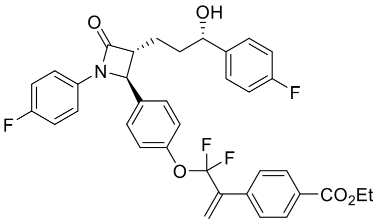

Ethyl 4-(3,3-difluoro-3-(4-((2*S*,3*R*)-1-(4-fluorophenyl)-3-((*S*)-3-(4-fluorophenyl)-3-hydroxypropyl)-4-oxoazetidin-2-yl)phenoxy)prop-1-en-2-yl)benzoate (3r). Compound **3r** (59.0 mg, 93% yield, α/γ > 20:1) as a colorless oil was purified with silica gel chromatography (Petroleum ether: Ethyl acetate = 3: 1). ^{1}H NMR (400 MHz, $CDCl_3$) δ 8.03 (d, *J* = 8.4 Hz, 2 H), 7.59 (d, *J* = 8.4 Hz, 2 H), 7.32 – 7.25 (m, 4 H), 7.23 – 7.16 (m, 4 H), 7.00 (t, *J* = 8.4 Hz, 2 H), 6.93 (t, *J* = 8.4 Hz, 2 H), 6.08 (s, 1 H), 5.80 (s, 1 H), 4.70 (t, *J* = 6.0 Hz, 1 H), 4.62 (d, *J* = 2.0 Hz, 1 H), 4.38 (q, *J* = 7.2 Hz, 2 H), 3.10 – 3.04 (m, 1 H), 2.15 (br, 1 H), 2.07 – 1.82 (m, 4 H), 1.40 (t, *J* = 7.2 Hz, 3 H). ^{19}F NMR (376 MHz, $CDCl_3$) δ -67.4 (s, 2 F), -114.9 (m, 1 F), -117.7 (m, 1 F). ^{13}C NMR (126 MHz, $CDCl_3$) δ 167.3, 166.2, 162.1 (d, *J* = 246.1 Hz), 159.0 (d, *J* = 244.2 Hz), 150.2, 140.7 (t, *J* = 29.6 Hz), 140.0 (d, *J* = 3.0 Hz), 139.4, 134.8, 133.6 (d, *J* = 2.5 Hz), 130.4, 129.5, 127.6, 127.3 (d, *J* = 7.7 Hz), 126.9, 122.5, 121.3 (t, *J* = 264.7 Hz), 121.1 (t, *J* = 5.2 Hz), 118.3 (d, *J* = 7.8 Hz), 115.9 (d, *J* = 22.7 Hz), 115.3 (d, *J* = 21.4 Hz), 73.0, 61.1, 60.7, 60.3, 36.5, 25.0, 14.3. MS (ESI): m/z (%) 634 $(M+H)^+$. HRMS (ESI): Calcd. for $C_{36}H_{32}O_5NF_4$: 634.2211 $(M+H)^+$; Found: 634.2202 $(M+H)^+$.

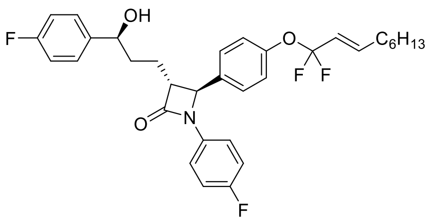

(3*R*,4*S*)-4-(4-(((*E*)-1,1-difluoronon-2-en-1-yl)oxy)phenyl)-1-(4-fluorophenyl)-3-((*S*)-3-(4-fluorophenyl)-3-hydroxypropyl)azetidin-2-one (3s). Compound **3s** (63.8 mg, 56% yield, *E* : *Z* > 20:1, α/γ > 20:1) as a colorless oil was purified with silica gel chromatography (Petroleum ether: Ethyl acetate = 3: 1). ^{1}H NMR (400 MHz, $CDCl_3$) δ 7.31 – 7.26 (m, 4 H), 7.24 – 7.17 (m, 4 H), 7.01 (t, *J* = 8.4 Hz, 2 H), 6.94 (t, *J* = 8.4 Hz, 2 H), 6.43 – 6.34 (m, 1 H), 5.74 – 5.65 (m, 1 H), 4.71 (t, *J* = 5.6 Hz, 1 H), 4.62 (d, *J* = 2.4 Hz, 1 H), 3.11 – 3.05 (m, 1 H), 2.18 – 2.10 (m, 2 H), 2.05 – 1.82 (m, 5 H), 1.48 – 1.39 (m, 2 H), 1.36 – 1.23 (m, 6 H), 0.89 (t, *J* = 6.8 Hz, 3 H). ^{19}F NMR (376 MHz, $CDCl_3$) δ -66.4 (d, *J* = 5.3 Hz, 2 F), -114.8 (m, 1 F), -117.8 (m, 1 F). ^{13}C NMR (151 MHz, $CDCl_3$) δ 167.4, 162.2 (d, *J* = 245.7 Hz), 159.1 (d, *J* = 244.2 Hz), 150.7, 140.0 (d, *J* = 2.4 Hz), 139.3 (t, *J* = 5.7 Hz), 134.4, 133.7, 127.4 (d, *J* = 8.2 Hz), 126.8, 122.5, 121.24 (t, *J* = 32.9 Hz), 121.18 (t, *J* = 260.2 Hz), 118.4 (d, *J* = 8.2 Hz), 115.9 (d, *J* = 23.1 Hz), 115.4 (d, *J* = 21.1 Hz), 73.1, 60.8, 60.4, 36.5, 31.6, 31.5, 28.7, 28.1, 25.0, 22.5, 14.0. MS (ESI): m/z (%) 592 $(M+Na)^+$. HRMS (ESI): Calcd. for $C_{33}H_{35}O_3NF_4Na$: 592.2445 $(M+Na)^+$; Found: 592.2453 $(M+Na)^+$.

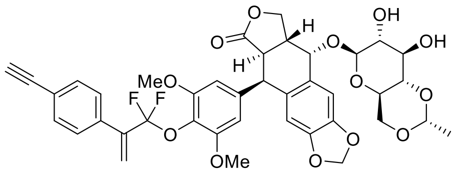

(5*R*,5a*R*,8a*R*,9*S*)-9-(((2*R*,4a*R*,6*R*,7*R*,8*R*,8a*S*)-7,8-dihydroxy-2-methylhexahydropyrano[3,2-*d*][1,3]dioxin-6-yl)oxy)-5-(4-((2-(4-ethynylphenyl)-1,1-difluoroallyl)oxy)-3,5-dimethoxyphenyl)-5,8,8a,9-tetrahydrofuro[3',4':6,7]naphtho[2,3-*d*][1,3]dioxol-6(5a*H*)-one (3t). Compound **3t** (61.2 mg, 80% yield, α/γ > 20:1) as a white solid (m.p. 133-140 °C) was purified with silica gel chromatography (Methanol: Dichloromethane = 1: 15). ^{1}H NMR (400 MHz, $CDCl_3$) δ 7.66 (d, *J* = 8.0 Hz, 2 H), 7.48 (d, *J* = 8.0 Hz, 2 H), 6.80 (s, 1 H), 6.45 (s, 2 H), 6.41 (s, 1 H), 6.09 (s, 1 H), 5.98 (s, 1 H), 5.96 (s, 1 H), 5.76 (s, 1 H), 4.93 (d, *J* = 3.2 Hz, 1 H), 4.72 (q, *J* = 5.2 Hz, 1 H), 4.57 – 4.45 (m, 2 H), 4.26 (d, *J* = 4.4 Hz, 1 H), 4.16 (d, *J* = 10.4 Hz, 4.4 Hz, 1 H), 3.93 (d, *J* = 7.6 Hz, 1 H), 3.77 (s, 6 H), 3.63 – 3.53 (m, 2 H), 3.44 (t, *J* = 8.0 Hz, 1 H), 3.33 (t, *J* = 9.2 Hz, 1 H), 3.22 – 3.12 (m, 2 H), 3.12 (s, 1 H), 3.02 – 2.90 (m, 2 H), 1.73 – 1.60 (m, 1 H), 1.36 (d, *J* = 5.2 Hz, 3 H). ^{19}F NMR (376 MHz, $CDCl_3$) δ -67.5 (d, *J* = 145.5 Hz, 1 F), -68.0 (d, *J* = 145.5 Hz, 1 F). ^{13}C NMR (126 MHz, $CDCl_3$) δ 180.1, 154.6, 148.7, 146.4, 142.4, 141.0 (t, *J* = 30.1 Hz), 135.8, 132.5, 131.7, 127.8, 127.0, 126.0, 121.9 (t, *J* = 266.1 Hz), 120.0 (t, *J* = 5.4 Hz), 109.7, 108.5, 105.6, 101.4, 99.7, 98.8, 83.4, 79.7, 77.9, 77.2, 75.3, 74.4, 72.9, 69.5, 68.1, 66.2, 56.3, 44.4, 44.0, 39.4, 20.3. MS (ESI): m/z (%) 787 $(M+Na)^+$. HRMS (ESI): Calcd. for $C_{40}H_{38}O_{13}F_2Na$: 787.2173 $(M+Na)^+$; Found: 787.2183 $(M+Na)^+$.

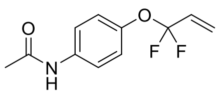

***N*-(4-((1,1-Difluoroallyl)oxy)phenyl)acetamide (3u).** 10 mmol-scale synthesis. To a 250 mL round bottom flask equipped with a stirring bar were added *N*-(4-hydroxyphenyl)acetamide (1.66 g, 11 mmol, 1.1 equiv) and DFAS **2a** (3.64 g, 10 mmol, 1.0 equiv) under air. DMSO (100 mL) and Na_2CO_3 aqueous solution (0.1 M, 50 mL) were added subsequently. The reaction mixture was stirred at 37 °C. After stirring for 1 h, the reaction mixture was then diluted with ethyl acetate and H_2O. The resulting mixture was extracted with ethyl acetate, and the combined organic layers were washed with brine, dried over Na_2SO_4, filtered through a pad of Celite®, and concentrated. Compound **3u** (1.88 g, 83% yield) as a light yellow solid (m.p. 80-83 °C) was purified with silica gel chromatography (Petroleum ether: Ethyl acetate = 2: 1). ^{1}H NMR (400 MHz, $CDCl_3$) δ 8.12 (s, 1 H), 7.47 (d, *J* = 8.8 Hz, 2 H), 7.11 (d, *J* = 8.8 Hz, 2 H), 6.08 – 5.96 (m, 1 H), 5.89 (d, *J* = 17.2 Hz, 1 H), 5.57 (d, *J* = 10.8 Hz, 1 H), 2.12 (s, 3 H). ^{19}F NMR (376 MHz, $CDCl_3$) δ -68.8 (d, *J* = 6.4 Hz, 2 F). ^{13}C NMR (126 MHz, $CDCl_3$) δ 168.8, 146.3, 135.5, 129.2 (t, *J* = 33.8 Hz), 122.4, 121.8 (t, *J* = 6.6 Hz), 121.0, 120.6 (t, *J* = 259.8 Hz), 24.3. MS (ESI): m/z (%) 228 $(M+H)^+$. HRMS (ESI): Calcd. for $C_{11}H_{12}O_2NF_2$: 228.0831 $(M+H)^+$; Found: 228.0831 $(M+H)^+$.

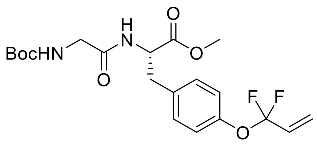

Methyl 2-(2-((*tert*-butoxycarbonyl)amino)acetamido)-3-(4-((1,1-difluoroallyl)oxy)phenyl)propanoate (3v). Compound **3v** (70.3 mg, 82% yield) as a colorless oil was purified with silica gel chromatography (Petroleum ether: Ethyl acetate = 1: 1). ^{1}H NMR (400 MHz, $CDCl_3$) δ 7.12 – 7.04 (m, 4 H), 6.83 – 6.74 (br, 1 H), 6.08 – 5.95 (m, 1 H), 5.88 (d, *J* = 17.2 Hz, 1 H), 5.56 (d, *J* = 10.4 Hz, 1 H), 5.29 (t, *J* = 5.2 Hz, 1 H), 4.84 (dd, *J* = 13.2 Hz, *J* = 6.0 Hz, 1 H), 3.86 – 3.69 (m, 2 H), 3.67 (s, 3 H), 3.14 – 3.01 (m, 2 H), 1.41 (s, 9 H). ^{19}F NMR (376 MHz, $CDCl_3$) δ -68.8 (d, *J* = 5.6 Hz, 2 F). ^{13}C NMR (126 MHz, $CDCl_3$) δ 171.6, 169.3, 156.0, 149.3, 132.9, 130.1, 129.3 (t, *J* = 33.9 Hz), 121.9, 121.7 (t, *J* = 6.3 Hz), 120.5 (t, *J* = 259.8 Hz), 80.2, 53.0, 52.3, 44.1, 37.1, 28.2. MS (ESI): m/z (%) 429 $(M+H)^+$. HRMS (ESI): Calcd. for $C_{20}H_{27}O_6N_2F_2$: 429.1832 $(M+H)^+$; Found: 429.1829 $(M+H)^+$.

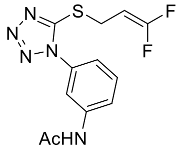

***N*-(3-(5-((3,3-Difluoroallyl)thio)-1*H*-tetrazol-1-yl)phenyl)acetamide (3w).** Compound **3w** (54.6 mg, 88% yield, γ/α > 20:1) as a yellow oil was purified with silica gel chromatography (Petroleum ether: Ethyl acetate = 1: 1). ^{1}H NMR (400 MHz, $CDCl_3$) δ 8.79 (s, 1 H), 8.03 (s, 1 H), 7.68 (d, *J* = 8.4 Hz, 1 H), 7.45 (t, *J* = 8.4 Hz, 1 H), 7.25 (d, *J* = 8.4 Hz, 1 H), 4.67 (dt, *J* = 23.6 Hz, 8.4 Hz, 1 H), 3.95 (d, *J* = 8.4 Hz, 2 H), 2.24 (s, 3 H). ^{19}F NMR (376 MHz, $CDCl_3$)

δ -83.7 (d, J = 30.8 Hz, 1 F), -84.7 (dd, J = 30.8 Hz, 24.1 Hz, 1 F). ^{13}C NMR (126 MHz, $CDCl_3$) δ 169.5, 157.5 (t, J = 292.1 Hz), 153.7, 139.8, 133.6, 130.2, 121.0, 118.4, 114.7, 75.0 (dd, J = 27.6 Hz, 18.3 Hz), 26.5 (d, J = 7.2 Hz), 24.4. MS (ESI): m/z (%) 312 $(M+H)^+$. HRMS (ESI): Calcd. for $C_{12}H_{12}ON_5F_2S$: 312.0725 $(M+H)^+$; Found: 312.0717 $(M+H)^+$.

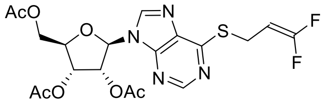

(2*R*,3*R*,4*R*,5*R*)-2-(Acetoxymethyl)-5-(6-((3,3-difluoroallyl)thio)-9*H*-purin-9-yl)tetrahydrofuran-3,4-diyl diacetate (3x). Compound **3x** was obtained from 6-thioinosine with difluoroallylation and acetylation in succession. Compound **3x** (83.7 mg, 86% yield, γ/α > 20:1) as a colorless oil was purified with silica gel chromatography (Petroleum ether: Ethyl acetate = 1: 1). ^{1}H NMR (500 MHz, $CDCl_3$) δ 8.69 (s, 1 H), 8.11 (s, 1 H), 6.18 (d, J = 5.0 Hz, 1 H), 5.93 (t, J = 5.0 Hz, 1 H), 5.63 (t, J = 5.0 Hz, 1 H), 4.60 (dt, J = 24.0 Hz, 8.0 Hz, 1 H), 4.45 – 4.38 (m, 2 H), 4.36 – 4.31 (m, 1 H), 3.95 (d, J = 8.0 Hz, 2 H), 2.11 (s, 3 H), 2.08 (s, 3 H), 2.04 (s, 3 H). ^{19}F NMR (376 MHz, $CDCl_3$) δ -86.0 (d, J = 36.1 Hz, 1 F), -86.7 (dd, J = 36.1 Hz, 24.1 Hz, 1 F). ^{13}C NMR (126 MHz, $CDCl_3$) δ 170.2, 169.5, 169.2, 160.5, 157.1 (t, J = 289.3 Hz), 152.0, 148.1, 141.2, 131.7, 86.4, 80.3, 76.1 (dd, J = 26.3 Hz, 18.4 Hz), 72.9, 70.4, 62.9, 21.9 (d, J = 6.7 Hz), 20.6, 20.4, 20.2. MS (ESI): m/z (%) 487 $(M+H)^+$. HRMS (ESI): Calcd. for $C_{19}H_{21}O_7N_4F_2S$: 487.1094 $(M+H)^+$; Found: 487.1095 $(M+H)^+$.

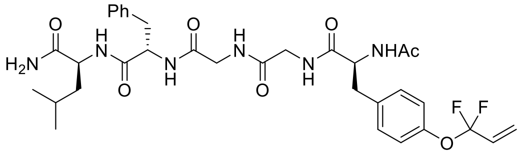

(*S*)-2-((2*S*,11*S*)-2-Benzyl-11-(4-((1,1-difluoroallyl)oxy)benzyl)-4,7,10,13-tetraoxo-3,6,9,12-tetraazatetradecanamido)-4-methylpentanamide (5a). 0.1 mmol-scale synthesis. To a 25 mL vial equipped with a stirring bar were added peptide (0.1 mmol, 1.0 equiv) and DFAS **2a** (0.15 mmol, 1.5 equiv) under air. DMSO (4 mL) and CBS buffer (2 mL) were added subsequently. The mixture was stirred at 37 °C. After stirring for 1 h, the reaction was cooled to room temperature and then diluted with H_2O. The resulting white solid was filtered to give crude product **5a**, which was washed by H_2O and petroleum ether to provide pure compound **5a** (68.7 mg, 72% yield, α/γ > 20:1). ^{1}H NMR (400 MHz, CD_3OD) δ 7.33 – 7.18 (m, 7 H), 7.12 (d, J = 8.0 Hz, 2 H), 6.17 – 6.03 (m, 1 H), 5.88 (dm, J = 17.2 Hz, 1 H), 5.62 (d, J = 10.8 Hz, 1 H), 4.60 (dd, J = 8.8 Hz, J = 5.6 Hz, 1 H), 4.53 (dd, J = 8.8 Hz, J = 6.0 Hz, 1 H), 4.38 – 4.30 (m, 1 H), 3.91 – 3.82 (m, 2 H), 3.80 – 3.68 (m, 2 H), 3.23 – 3.11 (m, 2 H), 3.05 – 2.90 (m, 2 H), 1.94 (s, 3 H), 1.68 – 1.54 (m, 3 H), 0.93 (d, J = 5.6 Hz, 3 H), 0.89 (d, J = 5.6 Hz, 3 H). ^{19}F NMR (376 MHz, CD_3OD) δ -69.9 (d, J = 6.1 Hz, 2 F). ^{13}C NMR (126 MHz, CD_3OD) δ 177.3, 174.5, 173.8, 173.4, 172.3, 171.9, 150.5, 138.4, 136.0, 131.3, 130.8 (t, J = 33.9 Hz), 130.4, 129.6,

127.9, 122.9, 122.4 (t, J = 6.6 Hz), 122.1 (t, J = 258.7 Hz), 56.7, 56.6, 53.0, 43.9, 43.6, 41.7, 38.3, 37.7, 25.8, 23.6, 22.5, 21.7. MS (ESI): m/z (%) 695 $(M+Na)^+$. HRMS (ESI): Calcd. for $C_{33}H_{42}O_7N_6F_2Na$: 695.2975 $(M+Na)^+$; Found: 695.2961 $(M+Na)^+$.

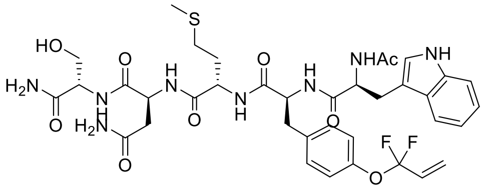

(*S*)-2-((*S*)-2-((*S*)-2-((*S*)-2-acetamido-3-(1*H*-indol-3-yl)propanamido)-3-(4-((1,1-difluoroallyl)oxy)phenyl)propanamido)-4-(methylthio)butanamido)-*N*¹-((*S*)-1-amino-3-hydroxy-1-oxopropan-2-yl)succinamide (5b). 0.1 mmol-scale synthesis. To a 25 mL vial equipped with a stirring bar was added pentapeptide (0.1 mmol, 1.0 equiv) and DFAS **2a** (0.15 mmol, 1.5 equiv) under air. DMSO (4 mL) and CBS buffer (2 mL) were added subsequently. The mixture was stirred at 37 ^oC. After stirring for 1 h, the reaction was cooled to room temperature and then diluted with H_2O. The resulting white solid was filtered to give crude product **5b**, which was washed by H_2O and petroleum ether to provide pure compound **5b** (67.6 mg, 83% yield, $\alpha/\gamma > 20:1$). ^1H NMR (400 MHz, DMSO-d_6) δ 10.70 (s, 1 H), 8.19 (d, J = 6.8 Hz, 1 H), 8.13 – 8.05 (m, 2 H), 7.97 (d, J = 8.0 Hz, 1 H), 7.79 (d, J = 7.2 Hz, 1 H), 7.52 (d, J = 7.6 Hz, 1 H), 7.43 (s, 1 H), 7.31 – 7.25 (m, 2 H), 7.23 (d, J = 7.6 Hz, 2 H), 7.11 – 6.89 (m, 7 H), 6.23 – 6.10 (m, 1 H), 5.85 (d, J = 17.2 Hz, 1 H), 5.65 (d, J = 10.8 Hz, 1 H), 4.76 (m, 1 H), 4.57 – 4.39 (m, 3 H), 4.38 – 4.30 (m, 1 H), 4.12 – 4.05 (m, 1 H), 3.65 – 3.57 (m, 1 H), 3.57 – 3.50 (m, 1 H), 3.07 – 2.92 (m, 2 H), 2.85 – 2.72 (m, 2 H), 2.61 – 2.53 (m, 1 H), 2.45 – 2.37 (m, 3 H), 1.99 (s, 3 H), 1.94 – 1.84 (m, 1 H), 1.82 – 1.73 (m, 1 H), 1.69 (s, 3 H). ^{19}F NMR (376 MHz, DMSO-d_6) δ -66.9 (d, J = 6.0 Hz, 2 F). ^{13}C NMR (126 MHz, DMSO-d_6) δ 172.0, 171.86, 171.85, 171.00, 170.96, 170.7, 169.3, 148.1, 136.1, 135.7, 130.6, 129.1 (t, J = 33.6 Hz), 127.3, 123.4, 123.0 (t, J = 6.3 Hz), 121.3, 120.9, 120.8 (t, J = 258.2 Hz), 118.4, 118.2, 111.3, 110.3, 61.5, 55.3, 53.7, 53.4, 52.0, 49.8, 36.9, 36.3, 32.0, 29.4, 27.5, 22.5, 14.6. MS (ESI): m/z (%) 817 $(M+H)^+$. HRMS (ESI): Calcd. for $C_{37}H_{47}O_9N_8F_2S$: 817.3149 $(M+H)^+$; Found: 817.3160 $(M+H)^+$.

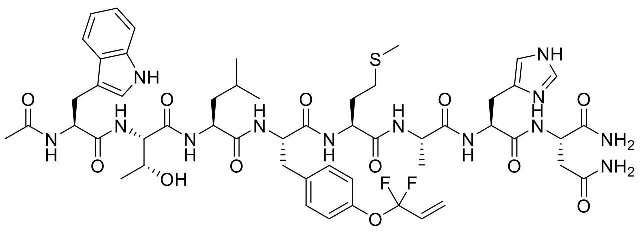

(*S*)-2-((2*S*,5*S*,8*S*,11*S*,14*S*,17*S*,20*S*)-2-((1*H*-imidazol-4-yl)methyl)-20-((1*H*-indol-3-yl)methyl)-11-(4-((1,1-difluoroallyl)oxy)benzyl)-17-((*R*)-1-hydroxyethyl)-14-isobutyl-5-methyl-8-(2-(methylthio)ethyl)-4,7,10,13,16,19,22-heptaoxo-3,6,9,12,15,18,21-heptaazatricosanamido)succinamide (5c). 0.05 mmol-scale synthesis. To a 25 mL

vial equipped with a stirring bar were added peptide (0.05 mmol, 1.0 equiv) and DFAS **2a** (0.075 mmol, 1.5 equiv) under air. DMSO (4 mL) and CBS buffer (2 mL) were added subsequently. The mixture was stirred at 37 °C. After stirring for 1 h, the reaction was cooled to room temperature and then diluted with H_2O. The resulting white solid was filtered to give crude product **5c**, which was washed by H_2O and petroleum ether to provide pure compound **5c** (47.2 mg, 82% yield, α/γ > 20:1). ^{1}H NMR (600 MHz, DMSO-d_6) δ 10.7 (s, 1 H), 8.10 (d, *J* = 7.2 Hz, 1 H), 8.08 – 8.04 (m, 2 H), 7.99 – 7.94 (m, 2 H), 7.92 (d, *J* = 7.8 Hz, 1 H), 7.79 – 7.74 (m, 2 H), 7.54 (d, *J* = 7.8 Hz, 1 H), 7.49 (s, 1 H), 7.29 – 7.25 (m, 2 H), 7.22 (d, *J* = 8.4 Hz, 2 H), 7.11 (d, *J* = 1.8 Hz, 1 H), 7.05 – 6.98 (m, 4 H), 6.91 (t, *J* = 7.8 Hz, 1 H), 6.81 (s, 2 H), 6.20 – 6.12 (m, 1 H), 5.85 (dm, *J* = 17.4 Hz, 1 H), 5.66 (d, *J* = 11.4 Hz, 1 H), 4.95 (br, 1 H), 4.57 – 4.52 (m, 1 H), 4.49 – 4.44 (m, 1 H), 4.43 – 4.38 (m, 1 H), 4.35 – 4.26 (m, 2 H), 4.24 – 4.14 (m, 3 H), 3.98 – 3.92 (m, 1 H), 3.08 (dd, *J* = 15.0 Hz, 4.2 Hz, 1 H), 3.00 (dd, *J* = 13.8 Hz, 3.6 Hz, 1 H), 2.93 – 2.86 (m, 2 H), 2.82 (dd, *J* = 14.4 Hz, 6.0 Hz, 1 H), 2.79 – 2.74 (m, 1 H), 2.47 – 2.45 (m, 4 H), 2.42 – 2.35 (m, 3 H), 1.97 (s, 3 H), 1.92 – 1.83 (m, 1 H), 1.74 (s, 3 H), 1.55 – 1.48 (m, 1 H), 1.37 – 1.29 (m, 2 H), 1.17 (d, *J* = 7.2 Hz, 3 H), 0.93 (d, *J* = 6.6 Hz, 3 H), 0.80 (d, *J* = 6.6 Hz, 3 H), 0.76 (d, *J* = 6.6 Hz, 3 H). ^{19}F NMR (376 MHz, DMSO-d_6) δ -66.9 (d, *J* = 6.0 Hz, 2 F). ^{13}C NMR (151 MHz, DMSO-d_6) δ 173.2, 172.2, 172.0, 171.9, 170.82, 170.78, 170.3, 169.9, 169.7, 148.1, 136.1, 135.4, 134.8, 130.4, 129.1 (t, *J* = 33.4 Hz), 127.4, 123.6, 122.9 (t, *J* = 6.3 Hz), 121.3, 120.9, 120.8 (t, *J* = 258.1 Hz), 118.4, 118.2, 111.3, 110.2, 66.4, 58.2, 53.8, 53.6, 53.0, 51.9, 51.3, 49.7, 48.5, 40.5, 40.1, 36.8, 36.3, 31.9, 29.5, 27.3, 24.1, 23.0, 22.5, 21.6, 19.5, 17.8, 14.6. MS (ESI): m/z (%) 1152 $(M+H)^+$. HRMS (ESI): Calcd. for $C_{53}H_{72}O_{12}N_{13}F_2S$: 1152.5107 $(M+H)^+$; Found: 1152.5110 $(M+H)^+$.

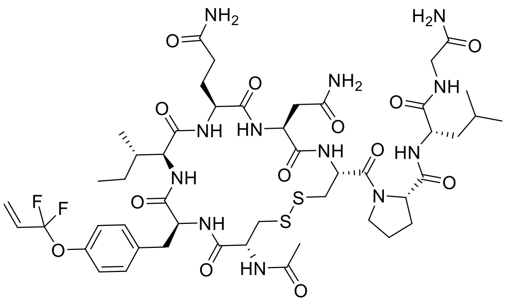

(*S*)-1-((4*R*,7*S*,10*S*,13*S*,16*S*,19*R*)-19-Acetamido-7-(2-amino-2-oxoethyl)-10-(3-amino-3-oxopropyl)-13-((*S*)-*sec*-butyl)-16-(4-((1,1-difluoroallyl)oxy)benzyl)-6,9,12,15,18-pentaoxo-1,2-dithia-5,8,11,14,17-pentaazacycloicosane-4-carbonyl)-*N*-((*S*)-1-((2-amino-2-oxoethyl)amino)-4-methyl-1-oxopentan-2-yl)pyrrolidine-2-carboxamide (5d). 0.05 mmol-scale synthesis. To a 25 mL vial equipped with a stirring bar was added peptide (0.05 mmol, 1.0 equiv) and DFAS **2a** (0.075 mmol, 1.5 equiv) under air. DMSO (4 mL) and CBS buffer (2 mL) were added subsequently. The mixture was stirred at 37 °C. After stirring for 1 h, the reaction was cooled to room temperature and then diluted with H_2O. The resulting white solid was filtered to give crude product **5d**,

which was washed by H_2O and petroleum ether to provide pure compound **5d** (36.5 mg, 65% yield, α/γ > 20:1). ^{1}H NMR (400 MHz, CD_3OD) δ 7.40 (d, *J* = 8.0 Hz, 2 H), 7.11 (d, *J* = 8.0 Hz, 2 H), 6.18 – 6.06 (m, 1 H), 5.89 (d, *J* = 17.6 Hz, 1 H), 5.63 (d, *J* = 10.8 Hz, 1 H), 5.27 (t, *J* = 7.2 Hz, 1 H), 4.89 – 4.79 (m, 3 H), 4.71 (dd, *J* = 8.0 Hz, 4.8 Hz, 1 H), 4.47 – 4.42 (m, 1 H), 4.29 (t, *J* = 7.6 Hz, 1 H), 4.10 (dd, *J* = 8.4 Hz, 4.8 Hz, 1 H), 3.91 (d, *J* = 9.6 Hz, 1 H), 3.88 (s, 1 H), 3.83 – 3.76 (m, 1 H), 3.75 – 3.65 (m, 1 H), 3.44 (dd, *J* = 13.6 Hz, 4.4 Hz, 1 H), 3.40 – 3.33 (m, 1 H), 3.17 (dd, *J* = 13.6 Hz, 6.8 Hz, 1 H), 3.07 – 2.87 (m, 3 H), 2.80 – 2.72 (m, 2 H), 2.44 – 2.32 (m, 2 H), 2.31 – 2.21 (m, 1 H), 2.20 – 2.09 (m, 2 H), 2.08 – 1.96 (m, 3 H), 1.94 – 1.86 (m, 5 H), 1.75 – 1.57 (m, 4 H), 1.31 – 1.19 (m, 1 H), 1.04 – 0.94 (m, 9 H), 0.92 (d, *J* = 6.0 Hz, 3 H). ^{19}F NMR (376 MHz, CD_3OD) δ -69.8 (d, *J* = 6.3 Hz, 2 F). ^{13}C NMR (126 MHz, CD_3OD) δ 178.0, 175.2, 175.0, 174.7, 174.4, 173.6, 172.6, 172.2, 171.5, 170.6, 150.4, 136.3, 131.8, 130.8 (t, *J* = 34.0 Hz), 122.6, 122.3 (t, *J* = 6.2 Hz), 122.1 (t, *J* = 258.3 Hz), 62.7, 62.4, 56.2, 54.9, 54.2, 53.8, 53.1, 52.3, 43.5, 43.3, 41.1, 40.8, 40.4, 38.5, 37.0, 36.9, 32.8, 30.4, 27.1, 27.0, 26.0, 23.5, 22.4, 21.8, 16.1, 11.6. MS (ESI): m/z (%) 1125 $(M+H)^+$. HRMS (ESI): Calcd. for $C_{48}H_{71}O_{13}N_{12}F_2S_2$: 1125.4668 $(M+H)^+$; Found: 1125.4658 $(M+H)^+$.

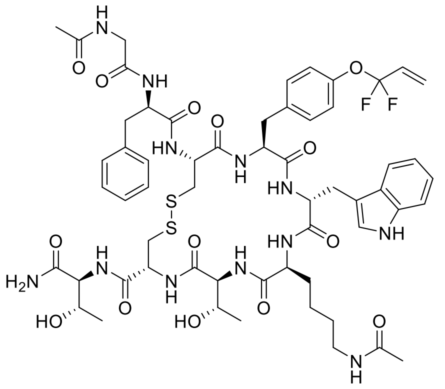

(4*R*,7*S*,10*S*,13*R*,16*S*,19*R*)-13-((1*H*-indol-3-yl)methyl)-19-((*R*)-2-(2-acetamidoacetamido)-3-phenylpropanamido)-10-(4-acetamidobutyl)-*N*-((2*S*,3*S*)-1-amino-3-hydroxy-1-oxobutan-2-yl)-16-(4-((1,1-difluoroallyl)oxy)benzyl)-7-((*S*)-1-hydroxyethyl)-6,9,12,15,18-pentaoxo-1,2-dithia-5,8,11,14,17-pentaazacycloicosane-4-carboxamide (5e). 0.05 mmol-scale synthesis. To a 25 mL vial equipped with a stirring bar, was added peptide (0.05 mmol, 1.0 equiv) and DFAS **2a** (0.075 mmol, 1.5 equiv) under air. DMSO (4 mL) and CBS buffer (2 mL) were added subsequently. The vial was heated to 37 ^{o}C. After stirring for 1 h, the reaction was cooled to room temperature and then diluted with H_2O. The resulting white solid was filtered to give crude product **5e**, which was washed by H_2O and petroleum ether to provide pure compound **5e** (46.1 mg, 73% yield). ^{1}H NMR (600 MHz, DMSO-d_6) δ 10.81 (s, 1 H), 8.94 (d, *J* = 9.0 Hz, 1 H), 8.79 (d, *J* = 5.4 Hz, 1 H), 8.52 (d, *J* = 6.6 Hz, 1 H), 8.47 (d, *J* = 9.0 Hz, 1 H), 8.36 (d, *J* = 8.4 Hz, 1 H), 8.24 (d, *J* = 8.4 Hz, 1 H), 8.01 (d, *J* = 8.4 Hz, 1 H), 7.87 (t, *J* = 5.4 Hz, 1 H), 7.73 (t, *J* = 5.4 Hz, 1 H), 7.53 (d, *J* = 9.0 Hz, 1 H), 7.49 – 7.43 (m, 2 H), 7.35 – 7.29 (m, 4 H), 7.25 (t, *J* = 7.2 Hz, 2 H), 7.18 (t, *J* = 7.2 Hz, 1 H), 7.11 – 7.04 (m, 4 H), 7.03 – 6.96 (m, 3 H), 6.24 – 6.16 (m, 1 H), 5.89 (d, *J* =

17.4 Hz, 1 H), 5.69 (d, J = 10.8 Hz, 1 H), 5.36 – 5.27 (m, 2 H), 5.20 (d, J = 4.8 Hz, 1 H), 4.81 – 4.73 (m, 2 H), 4.66 – 4.60 (m, 1 H), 4.54 – 4.48 (m, 1 H), 4.28 – 4.20 (m, 2 H), 4.12 – 4.06 (m, 1 H), 4.03 – 3.91 (m, 2 H), 3.66 (dd, J = 16.8 Hz, 6.0 Hz, 1 H), 3.50 (dd, J = 16.8 Hz, 5.4 Hz, 1 H), 3.15 (d, J = 10.8 Hz, 1 H), 3.01 (dd, J = 13.2 Hz, 9.0 Hz, 1 H), 2.93 – 2.77 (m, 9 H), 2.70 (dd, J = 13.8 Hz, 6.0 Hz, 1 H), 1.80 (s, 3 H), 1.78 (s, 3 H), 1.73 – 1.65 (m, 1 H), 1.32 – 1.23 (m, 1 H), 1.23 – 1.13 (m, 2 H), 1.10 (d, J = 6.6 Hz, 3 H), 1.05 (d, J = 6.0 Hz, 3 H), 0.83 – 0.71 (m, 2 H). ^{19}F NMR (565 MHz, DMSO-d$_6$) δ -66.9 (d, J = 6.4 Hz, 2 F). ^{13}C NMR (151 MHz, DMSO-d$_6$) δ 173.0, 172.5, 172.4, 171.7, 171.0, 170.9, 170.0, 169.9, 169.49, 169.47, 169.1, 148.6, 138.4, 136.6, 135.1, 130.7, 129.8, 129.6 (t, J = 34.1 Hz), 128.4, 127.4, 126.7, 124.3, 123.4 (t, J = 6.5 Hz), 121.8, 121.4, 121.2 (t, J = 257.9 Hz), 118.7, 118.5, 111.8, 109.4, 67.7, 67.6, 58.7, 58.5, 55.7, 54.2, 53.2, 52.8, 52.4, 45.5, 44.8, 42.1, 40.9, 40.5, 39.3, 38.7, 38.5, 31.2, 29.2, 26.6, 23.1, 22.9, 20.5, 19.8. MS (ESI): m/z (%) 1265 (M+H)$^+$. HRMS (ESI): Calcd. for $C_{58}H_{75}O_{14}N_{12}F_2S_2$: 1265.4930 (M+H)$^+$; Found: 1265.4933 (M+H)$^+$.

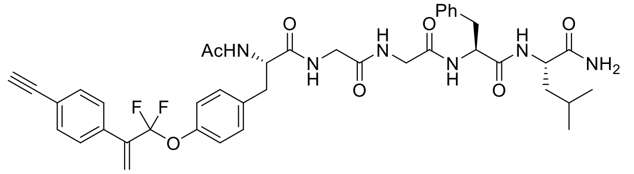

(*S*)-2-((2*S*,11*S*)-2-benzyl-11-(4-((2-(4-ethynylphenyl)-1,1-difluoroallyl)oxy)benzyl)-4,7,10,13-tetraoxo-3,6,9,12-tetraazatetradecanamido)-4-methylpentanamide (5f). The reaction mixture was cooled to room temperature and then diluted with H_2O. The resulting white solid was filtered to give crude product **5f**, which was washed by H_2O and petroleum ether to give pure **5f** (56.3 mg, 73% yield, α/γ > 20:1). ^{1}H NMR (500 MHz, DMSO-d$_6$) δ 8.33 (t, J = 6.0 Hz, 1 H), 8.17 (d, J = 8.0 Hz, 1 H), 8.09 (d, J = 8.0 Hz, 1 H), 8.05 (t, J = 6.0 Hz, 1 H), 7.99 (d, J = 8.5 Hz, 1 H), 7.60 – 7.57 (m, 2 H), 7.55 – 7.52 (m, 2 H), 7.29 – 7.26 (m, 2 H), 7.26 – 7.23 (m, 4 H), 7.20 – 7.15 (m, 1 H), 7.14 – 7.10 (m, 3 H), 6.99 (s, 1 H), 6.04 (s, 1 H), 6.01 (s, 1 H), 4.53 – 4.46 (m, 2 H), 4.29 (s, 1 H), 4.22 – 4.16 (m, 1 H), 3.77 – 3.65 (m, 3 H), 3.61 (dd, J = 16.5 Hz, 5.5 Hz, 1 H), 3.06 – 3.00 (m, 2 H), 2.81 – 2.71 (m, 2 H), 1.76 (s, 3 H), 1.60 – 1.52 (m, 1 H), 1.49 – 1.44 (m, 2 H), 0.87 (d, J = 7.0 Hz, 3 H), 0.82 (d, J = 6.0 Hz, 3 H). ^{19}F NMR (471 MHz, DMSO-d$_6$) δ -65.8 (s, 2 F). ^{13}C NMR (126 MHz, DMSO-d$_6$) δ 174.1, 171.8, 170.9, 169.6, 169.2, 168.8, 148.0, 139.4 (t, J = 29.9 Hz), 137.8, 136.2, 134.7, 132.0, 130.5, 129.3, 128.2, 127.6, 126.4, 123.5, 121.5 (m), 121.41, 121.38 (t, J = 262.8 Hz), 83.1, 82.1, 54.2, 54.1, 51.1, 42.2, 42.0, 40.9, 37.5, 36.8, 24.3, 23.1, 22.5, 21.7. MS (ESI): m/z (%) 773 (M+H)$^+$. HRMS (ESI): Calcd. for $C_{41}H_{47}O_7N_6F_2$: 773.3469 (M+H)$^+$; Found: 773.3471 (M+H)$^+$.

6. Successive Modification of Peptides and Bioactive Molecules from Compounds 3 and 5

6.1 Click Reactions

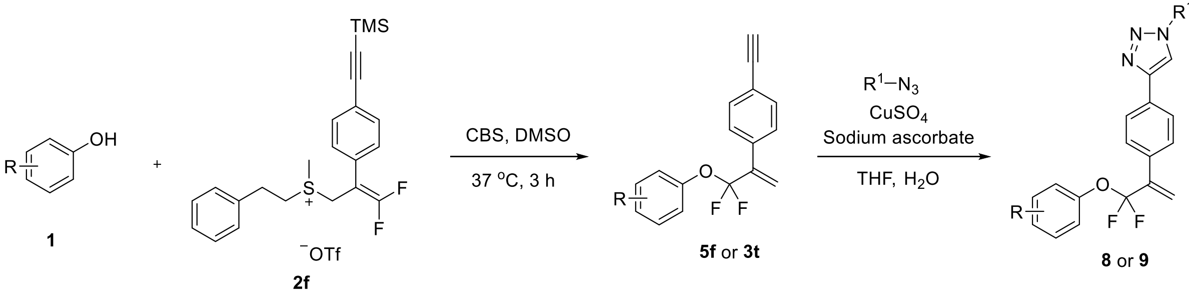

Synthesis of Compound 5f or 3t: To a 25 mL vial equipped with a stirring bar were added phenol **1** (0.1 mmol, 1.0 equiv) and DFAS **2f** (0.1 mmol, 1.0 equiv) under air. DMSO (4 mL) and CBS buffer (2 mL) were added subsequently. The mixture was stirred at 37 °C. After stirring for 3 h, the reaction was cooled to room temperature, and the corresponding product **5f** or **3t** was purified by the following method.

Synthesis of Compound 8 or 9: To a 25 mL vial equipped with a stirring bar were added $CuSO_4$ (0.05 mmol, 1.0 equiv), sodium ascorbate (0.05 mmol, 1.0 equiv), the corresponding azide compound **6** or **7** (0.075 mmol, 1.5 equiv), and *gem*-difluoroallylated phenol **5f** or **3t** (0.05 mmol, 1.0 equiv) under air. THF (2 mL) and H_2O (2 mL) were added subsequently. The mixture was stirred for 6 h, and the corresponding product **8** or **9** was purified by the following method.

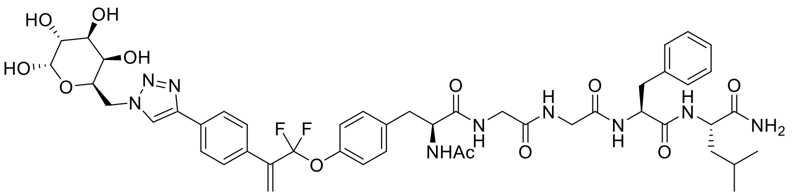

(*S*)-2-((2*S*,11*S*)-2-benzyl-11-(4-((1,1-difluoro-2-(4-(1-(((2*R*,3*R*,4*S*,5*R*,6*S*)-3,4,5,6-tetrahydroxytetrahydro-2*H*-pyran-2-yl)methyl)-1*H*-1,2,3-triazol-4-yl)phenyl)allyl)oxy)benzyl)-4,7,10,13-tetraoxo-3,6,9,12-tetraazatetradecanamido)-4-methylpentanamide (8). Compound **8** (37.1 mg, 76% yield) was purified by washing with H_2O and petroleum ether. ^1H NMR (400 MHz, CD_3OD) δ 8.11 (d, *J* = 7.2 Hz, 1 H), 7.58 (d, *J* = 8.0 Hz, 2 H), 7.35 (d, *J* = 8.0 Hz, 2 H), 7.03 – 6.90 (m, 8 H), 6.84 (d, *J* = 8.4 Hz, 2 H), 5.73 (s, 1 H), 5.58 (s, 1 H), 4.42 (dd, *J* = 14.8 Hz, 6.4 Hz, 2 H), 4.37 – 4.13 (m, 4 H), 4.10 – 4.04 (m, 1 H), 3.80 – 3.73 (m, 1 H), 3.67 – 3.51 (m, 5 H), 3.49 – 3.41 (m, 2 H), 3.29 – 3.24 (m, 1 H), 3.05 (s, 2 H), 2.95 – 2.85 (m, 2 H), 2.78 – 2.63 (m,

2 H), 1.67 (s, 3 H), 1.42 – 1.28 (m, 3 H), 0.65 (d, *J* = 4.8 Hz, 3 H), 0.61 (d, *J* = 4.8 Hz, 3 H). ^{19}F NMR (376 MHz, CD_3OD) δ -68.1 (s, 2 F). ^{13}C NMR (126 MHz, CD_3OD) δ 177.4, 174.5, 173.8, 173.5, 172.3, 171.9, 150.5, 142.6 (t, *J* = 29.7 Hz), 138.3, 136.2 (d, *J* = 3.3 Hz), 136.1, 132.0 (d, *J* = 5.2 Hz), 131.4, 130.4, 129.6, 129.3, 127.9, 126.5, 123.7 (d, *J* = 19.7 Hz), 123.0 (t, *J* = 262.7 Hz), 120.5, 98.7, 94.3, 74.7 (d, *J* = 14.7 Hz), 73.4, 71.5, 70.8 (d, *J* = 19.2 Hz), 70.2 (d, *J* = 19.2 Hz), 56.6 (d, *J* = 10.6 Hz), 53.1, 52.7 (d, *J* = 15.8 Hz), 49.8, 43.9, 43.6, 41.7, 38.3, 37.8, 25.8, 23.6, 22.5, 21.7. MS (ESI): m/z (%) 978 $(M+H)^+$. HRMS (ESI): Calcd. for $C_{47}H_{58}O_{12}N_9F_2$: 978.4168 $(M+H)^+$; Found: 978.4163 $(M+H)^+$.

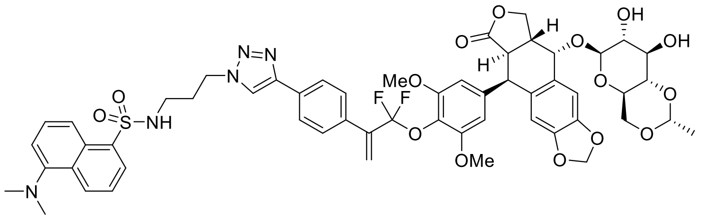

***N*-(3-(4-(4-(3-(4-((5*R*,5a*R*,8a*R*,9*S*)-9-(((2*R*,4a*R*,6*R*,7*R*,8*R*,8a*S*)-7,8-dihydroxy-2-methylhexahydropyrano[3,2-*d*][1,3]dioxin-6-yl)oxy)-6-oxo-5,5a,6,8,8a,9-hexahydrofuro[3',4':6,7]naphtho[2,3-*d*][1,3]dioxol-5-yl)-2,6-dimethoxyphenoxy)-3,3-difluoroprop-1-en-2-yl)phenyl)-1*H*-1,2,3-triazol-1-yl)propyl)-5-(dimethylamino)naphthalene-1-sulfonamide (9).** Compound **9** (46.7 mg, 85% yield) as a yellow solid (m.p. 159-164 ^{o}C) was purified with silica gel chromatography (Methanol: Dichloromethane = 1: 35). ^{1}H NMR (500 MHz, $CDCl_3$) δ 8.55 (d, *J* = 8.0 Hz, 1 H), 8.28 (d, *J* = 8.5 Hz, 1 H), 8.20 (d, *J* = 7.0 Hz, 1 H), 7.80 – 7.69 (m, 5 H), 7.55 (t, *J* = 8.0 Hz, 1 H), 7.49 (t, *J* = 8.0 Hz, 1 H), 7.19 (d, *J* = 7.5 Hz, 1 H), 6.80 (s, 1 H), 6.44 (s, 2 H), 6.40 (s, 1 H), 6.07 (s, 1 H), 5.96 (s, 1 H), 5.95 (s, 1 H), 5.78 (s, 1 H), 5.34 (br, 1 H), 4.93 (d, *J* = 3.0 Hz, 1 H), 4.71 (q, *J* = 5.0 Hz, 1 H), 4.54 (d, *J* = 9.0 Hz, 1 H), 4.51 – 4.46 (m, 1 H), 4.41 (t, *J* = 6.0 Hz, 2 H), 4.28 (d, *J* = 4.5 Hz, 1 H), 4.15 (dd, *J* = 10.0 Hz, 5.0 Hz, 1 H), 3.96 (d, *J* = 7.5 Hz, 1 H), 3.75 (s, 6 H), 3.62 – 3.54 (m, 2 H), 3.44 (t, *J* = 8.0 Hz, 1 H), 3.32 (t, *J* = 9.0 Hz, 1 H), 3.21 (dd, *J* = 9.5 Hz, 5.0 Hz, 1 H), 3.17 – 3.11 (m, 1 H), 3.02 – 2.96 (m, 1 H), 2.91 – 2.85 (m, 8 H), 2.09 – 2.03 (m, 2 H), 1.34 (d, *J* = 5.0 Hz, 3 H), 1.26 – 1.24 (m, 2 H). ^{19}F NMR (376 MHz, $CDCl_3$) δ -67.1 (m, 2 F). ^{13}C NMR (126 MHz, $CDCl_3$) δ 180.2, 154.5, 148.6, 146.4, 142.2, 141.1 (t, *J* = 30.1 Hz), 135.1, 134.2, 132.5, 130.7, 130.2, 129.8, 129.7, 129.4, 128.6, 128.3, 127.1, 126.1, 125.2, 123.3, 122.1 (t, *J* = 266.0 Hz), 120.6, 119.4, 115.4, 109.6, 108.5, 105.6, 101.4, 99.6, 99.0, 79.7, 75.3, 74.4, 73.0, 69.5, 68.1, 66.2, 56.3, 46.9, 45.4, 44.3, 44.1, 39.8, 39.4, 30.2, 29.7, 20.3. MS (ESI): m/z (%) 1098 $(M+H)^+$. HRMS (ESI): Calcd. for $C_{55}H_{58}O_{15}N_5F_2S$: 1098.3613 $(M+H)^+$; Found: 1098.3601 $(M+H)^+$.

6.2 Radical Addition to the Alkenes

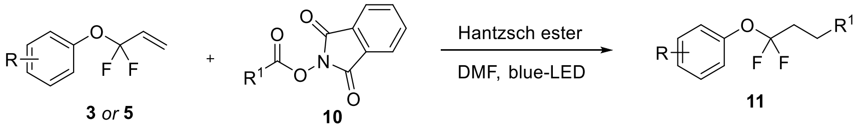

Procedure: To a 25 mL of Schlenk tube were added Hantzsch ester (0.2 mmol, 2.0 equiv), redox ester **10**[3] (0.2 mmol, 2.0 equiv), compound **3** or **5** (0.1 mmol, 1.0 equiv) under air. The reaction mixture was then evacuated and backfilled with Ar (3 times). DMF (2 mL) was added. The reaction mixture was stirred for 12 h under irradiation of blue LED (12W, 460-465 nm). The reaction mixture was diluted with ethyl acetate and H_2O. The organic layers were washed with brine three times, dried over Na_2SO_4, filtered, and concentrated. The residue was purified with silica gel chromatography to give the product **11**.

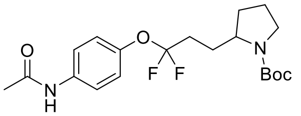

***tert*-Butyl 2-(3-(4-acetamidophenoxy)-3,3-difluoropropyl)pyrrolidine-1-carboxylate (11a).** Compound **11a** (41.5 mg, 99% yield) as a yellow oil was purified with silica gel chromatography (Petroleum ether: Ethyl acetate = 2: 1). ^{1}H NMR (400 MHz, DMSO-d6) δ 10.00 (s, 1 H), 7.59 (d, *J* = 8.8 Hz, 2 H), 7.11 (d, *J* = 8.8 Hz, 2 H), 3.78 – 3.69 (m, 1 H), 3.32 – 3.16 (m, 2 H), 2.23 – 2.06 (m, 2 H), 2.03 (s, 3 H), 1.98 – 1.86 (m, 2 H), 1.84 – 1.69 (m, 2 H), 1.68 – 1.56 (m, 2 H), 1.39 (s, 9 H). ^{19}F NMR (565 MHz, DMSO-d6, 80 °C) δ -68.5 (t, *J* = 11.3 Hz, 2 F). ^{13}C NMR (151 MHz, DMSO-d6, 80 °C) δ 167.7, 153.4, 144.8, 136.6, 124.8 (t, *J* = 264.2 Hz), 121.3, 119.9, 77.9, 55.6, 45.8, 31.6 (t, *J* = 29.5 Hz), 29.6, 27.8, 26.7, 23.3, 22.5. MS (ESI): m/z (%) 421 $(M+Na)^+$. HRMS (ESI): Calcd. for $C_{20}H_{28}O_4N_2F_2Na$: 421.1909 $(M+Na)^+$; Found: 421.1906 $(M+Na)^+$.

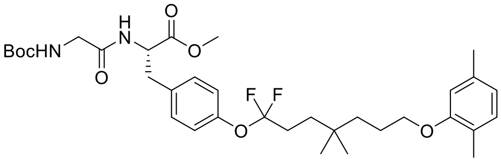

Methyl (*S*)-2-(2-((*tert*-butoxycarbonyl)amino)acetamido)-3-(4-((7-(2,5-dimethylphenoxy)-1,1-difluoro-4,4-dimethylheptyl)oxy)phenyl)propanoate (11b). Compound **11b** (38.6 mg, 61% yield) as a yellow oil was purified with silica gel chromatography (Petroleum ether: Ethyl acetate = 2: 1). ^{1}H NMR (400 MHz, $CDCl_3$) δ 7.13 – 7.03 (m, 4 H), 7.01 (d, *J* = 7.2 Hz, 1 H), 6.70 – 6.59 (m, 3 H), 5.20 – 5.08 (m, 1 H), 4.91 – 4.81 (m, 1 H), 3.93 (t, *J* = 6.0 Hz, 2 H), 3.88 – 3.79 (m, 1 H), 3.76 (d, *J* = 5.6 Hz, 1 H), 3.70 (s, 3 H), 3.16 – 3.04 (m, 2 H), 2.31 (s, 3 H), 2.19 (s, 3 H), 2.16 – 2.03 (m, 2 H), 1.84 – 1.71 (m, 2 H), 1.62 – 1.53 (m, 2 H), 1.44 (s, 9 H), 1.47 – 1.36 (m, 2 H), 0.95 (s, 6 H). ^{19}F NMR (376 MHz, $CDCl_3$) δ -70.9 (t, *J* = 10.5 Hz, 2 F). ^{13}C NMR (126 MHz, $CDCl_3$) δ 171.6, 169.2, 157.0,

156.0, 149.6, 136.4, 132.6, 130.2, 130.1, 125.5 (t, *J* = 266.6 Hz), 123.5, 121.7, 120.6, 111.9, 80.3, 68.3, 53.0, 52.4, 44.2, 37.7, 37.1, 33.5, 31.8, 31.0 (t, *J* = 28.6 Hz), 28.2, 26.9, 24.1, 21.4, 15.8. MS (ESI): m/z (%) 657 $(M+Na)^+$. HRMS (ESI): Calcd. for $C_{34}H_{48}O_7N_2F_2Na$: 657.3322 $(M+Na)^+$; Found: 657.3327 $(M+Na)^+$.

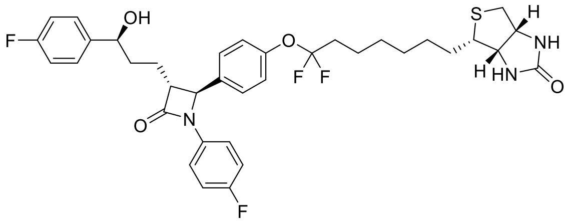

(3a*S*,4*S*,6a*R*)-4-(7,7-Difluoro-7-(4-((2*S*,3*R*)-1-(4-fluorophenyl)-3-((*S*)-3-(4-fluorophenyl)-3-hydroxypropyl)-4-oxoazetidin-2-yl)phenoxy)heptyl)tetrahydro-1*H*-thieno[3,4-*d*]imidazol-2(3*H*)-one (11c). Compound **11c** (19.1 mg, 28% yield, 78% purity) as a colorless oil was purified with purified silica gel chromatography (Methanol: Dichloromethane = 1: 15). Analytical sample was purified by reverse-phase preparative HPLC (SHIMADZU, Shim-pack GIS, 5 um C18, 20*250 mm, methol:water = 7:3). ^{1}H NMR (400 MHz, $CDCl_3$) δ 7.31 – 7.25 (m, 4 H), 7.24 – 7.19 (m, 2 H), 7.17 (d, *J* = 8.0 Hz, 2 H), 7.04 – 6.97 (m, 2 H), 6.96 – 6.89 (m, 2 H), 5.11 (br, 1 H), 4.70 (t, *J* = 6.4 Hz, 1 H), 4.63 – 4.59 (m, 1 H), 4.54 – 4.47 (m, 1 H), 4.33 – 4.27 (m, 1 H), 3.20 – 3.12 (m, 1 H), 3.11 – 3.05 (m, 1 H), 2.92 (dd, *J* = 12.8 Hz, 4.4 Hz, 1 H), 2.73 (d, *J* = 12.8 Hz, 1 H), 2.18 – 1.84 (m, 8 H), 1.70 – 1.57 (m, 4 H), 1.47 – 1.33 (m, 6 H). ^{19}F NMR (376 MHz, $CDCl_3$) δ -70.6 (t, *J* = 11.3 Hz, 2 F), -115.0 (m, 1 F), -117.9 (m, 1 F). ^{13}C NMR (126 MHz, CD_3OD) δ 169.5, 166.2, 163.5 (d, *J* = 244.2 Hz), 160.5 (d, *J* = 242.9 Hz), 152.0, 142.2 (d, *J* = 2.5 Hz), 136.4, 135.1, 128.8 (d, *J* = 8.4 Hz), 128.4, 126.6 (t, *J* = 266.0 Hz), 123.5, 119.9 (d, *J* = 7.8 Hz), 116.8 (d, *J* = 23.6 Hz), 115.9 (d, *J* = 21.5 Hz), 73.7, 63.5, 61.7, 61.6, 61.3, 57.2, 41.0, 37.4, 36.6 (t, *J* = 29.4 Hz), 30.3, 30.2, 29.8, 29.7, 26.1, 23.6. MS (ESI): m/z (%) 686 $(M+H)^+$. HRMS (ESI): Calcd. for $C_{36}H_{39}O_4N_3F_4SNa$: 708.2490 $(M+Na)^+$; Found: 708.2486 $(M+Na)^+$.

Synthesis of *O*-Link Type DOTA-TATE

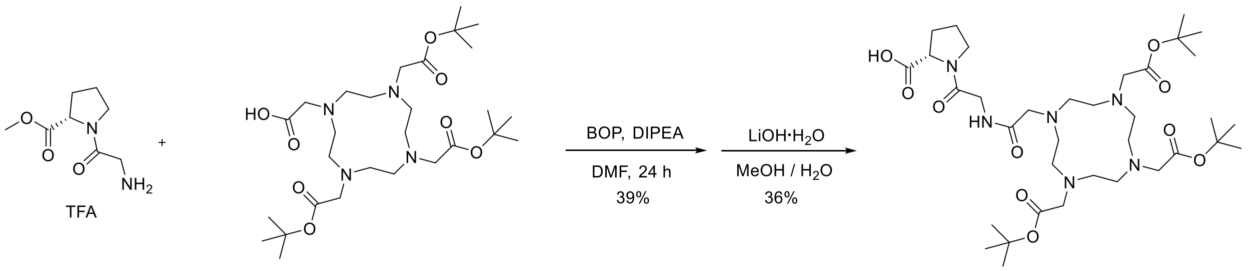

12

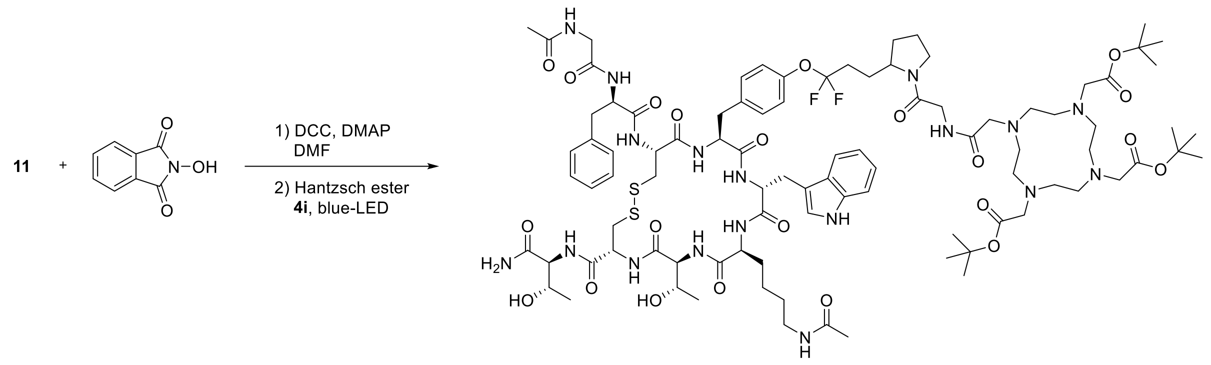

11d

To a 100 mL round bottle equipped with a magnetic stir bar were added TFA-Gly-Pro-OMe (1.8 g, 6 mmol, 1.2 equiv), BOP (2.65 g, 6 mmol, 1.2 equiv), 2-(4,7,10-tris(2-(*tert*-butoxy)-2-oxoethyl)-1,4,7,10-tetraazacyclododecan-1-yl)acetic acid (2.86 g, 5 mmol, 1.0 equiv) and DMF (20 mL). Subsequently, DIPEA (1.94 g, 15 mmol, 3.0 equiv) was added. After the reaction was stirred at room temperature for 24 h, H_2O and EA were added. The resulting mixture was then extracted by EA 2 times. Then, the organic layer was washed with brine three times. The combined organic phase was dried over Na_2SO_4, filtered, and concentrated. The DOTA-Gly-Pro-OMe (1.43 g, 39% yield) as a yellow oil was purified with silica gel chromatography (Methanol: Dichloromethane = 1: 100).

To a 100 mL round bottle equipped with a magnetic stir bar were added DOTA-Gly-Pro-OMe (1.4 g, 1.9 mmol, 1.0 equiv) and a solution of $LiOH{\cdot}H_2O$ (239.2 mg, 5.7 mmol, 3.0 equiv) in MeOH (16 mL) and H_2O (4 mL). After stirring at room temperature for 12 h, the reaction mixture was quenched with saturated NH_4Cl solution. The resulting mixture was then extracted by EA 3 times. The combined organic phase was dried over Na_2SO_4, filtered, and concentrated. Compound **12** (490.9 mg, 36% yield) as a white solid (m.p. 127-130 ºC) was purified with silica gel chromatography (Methanol: Dichloromethane = 1: 10). ^{1}H

NMR (400 MHz, DMSO-d6) δ 8.49 – 8.32 (m, 1 H), 4.26 – 4.05 (m, 2 H), 3.81 – 3.22 (m, 17 H), 3.03 – 2.76 (m, 6 H), 2.18 – 2.04 (m, 4 H), 1.98 – 1.84 (m, 4 H), 1.47 – 1.34 (m, 27 H). ^{13}C NMR (151 MHz, DMSO-d6, 80 ºC) δ 172.7, 172.4, 172.1 – 171.3 (m), 166.2, 80.9, 80.7, 69.5, 58.3, 56.0, 55.3, 51.0 – 48.9 (m), 45.2, 40.9, 28.2, 27.4, 27.3, 23.9. MS (ESI): m/z (%) 727 $(M+H)^+$. HRMS (ESI): Calcd. for $C_{35}H_{63}O_{10}N_6$: 727.4600 $(M+H)^+$; Found: 727.4586 $(M+H)^+$.

To a 25 mL Schlenk tube were added **12** (0.05 mmol), *N*-hydroxyphthalimide (0.05 mmol), DCC (0.05 mmol), DMAP (0.005 mmol) under air, followed by DMF (0.5 mL). The reaction mixture was stirred for 6 h to give the redox esters solution. To another Schlenk tube were added Hantzsch ester (0.05 mmol) and compounds **5e** (0.01 mmol) under air. The reaction mixture was then evacuated and backfilled with Ar (3 times). The redox ester solution was added to the reaction. The resulting reaction mixture was stirred for 12 h under irradiation of blue LED (12W, 460-465 nm). The reaction mixture was diluted with ethyl acetate and H_2O. The water layers were washed with ethyl acetate three times, compound **11d** (16.2 mg, 83% yield) as a brown solid was obtained after vacuum freeze-drying. ^{19}F NMR (376 MHz, $CDCl_3$) δ -69.2 (s, 1 F), -71-1 (s, 1 F). MS (ESI): m/z (%) 974 $(M+2H)^{2+}$. HRMS (ESI): Calcd. for $C_{92}H_{138}O_{22}N_{18}F_2S_2$: 974.4816 $(M+2H)^{2+}$; Found: 974.4809 $(M+2H)^{2+}$.

6.3 Control experiment with the nonfluorinated compound.

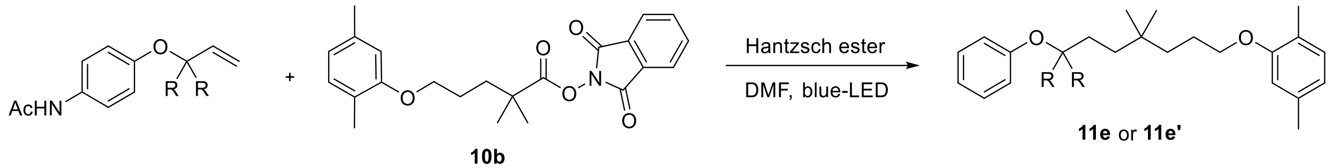

Entry	Substruct	Yield
1	R = F	**11e**, 99%
2	R = H	**11e'**, 10%

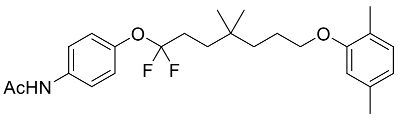

***N*-(4-((7-(2,5-dimethylphenoxy)-1,1-difluoro-4,4-dimethylheptyl)oxy)phenyl)acetamide (11e).** Compound **11e** (43.3 mg, 99% yield) as a yellow solid (m.p. 83-85 ºC) was purified with silica gel

chromatography (Petroleum ether: Ethyl acetate = 2: 1). ^{1}H NMR (500 MHz, $CDCl_3$) δ 7.93 (s, 1 H), 7.51 (d, *J* = 8.5 Hz, 2 H), 7.16 (d, *J* = 8.5 Hz, 2 H), 7.06 (d, *J* = 7.5 Hz, 1 H), 6.71 (d, *J* = 7.5 Hz, 1 H), 6.68 (s, 1 H), 3.97 (t, *J* = 6.5 Hz, 2 H), 2.36 (s, 3 H), 2.24 (s, 3 H), 2.14 (s, 3 H), 2.21 – 2.09 (m, 2 H), 1.86 – 1.78 (m, 2 H), 1.66 – 1.60 (m, 2 H), 1.48 – 1.42 (m, 2 H), 0.99 (s, 6 H). ^{19}F NMR (376 MHz, $CDCl_3$) δ -70.6 (t, *J* = 10.9 Hz, 2 F). ^{13}C NMR (126 MHz, $CDCl_3$) δ 168.7, 157.0, 146.6, 136.4, 135.2, 130.2, 125.5 (t, *J* = 266.0 Hz), 123.5, 122.3, 120.9, 120.6, 111.9, 68.2, 37.7, 33.5, 31.8, 30.9 (t, *J* = 28.6 Hz), 26.9, 24.2, 24.1, 21.3, 15.7. MS (ESI): m/z (%) 434 $(M+H)^+$. HRMS (ESI): Calcd. for $C_{25}H_{34}O_3NF_2$: 434.2501 $(M+H)^+$; Found: 434.2502 $(M+H)^+$.

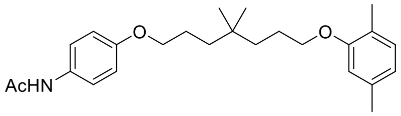

***N*-(4-((7-(2,5-dimethylphenoxy)-4,4-dimethylheptyl)oxy)phenyl)acetamide (11e').** Compound **11e'** (8.1 mg, 10% yield) as a colorless oil was purified with silica gel chromatography (Petroleum ether: Ethyl acetate = 2: 1). ^{1}H NMR (400 MHz, $CDCl_3$) δ 7.37 (d, *J* = 8.8 Hz, 2 H), 7.13 (s, 1 H), 7.00 (d, *J* = 7.6 Hz, 1 H), 6.84 (d, *J* = 8.8 Hz, 2 H), 6.65 (d, *J* = 7.6 Hz, 1 H), 6.62 (s, 1 H), 3.91 (t, *J* = 6.0 Hz, 4 H), 2.31 (s, 3 H), 2.18 (s, 3 H), 2.15 (s, 3 H), 1.80 – 1.69 (m, 4 H), 1.42 – 1.33 (m, 4 H), 0.93 (s, 6 H). ^{13}C NMR (126 MHz, $CDCl_3$) δ 168.2, 157.1, 156.0, 136.4, 130.7, 130.2, 123.5, 121.9, 120.5, 114.8, 111.9, 69.0, 68.5, 37.9, 37.7, 32.3, 27.1, 24.3, 24.2, 24.1, 21.4, 15.8. MS (ESI): m/z (%) 398 $(M+H)^+$. HRMS (ESI): Calcd. for $C_{25}H_{36}O_3N$: 398.2690 $(M+H)^+$; Found: 398.2684 $(M+H)^+$.

7. References

1. X.-T. Feng, J.-X. Ren, X. Gao, Q.-Q. Min, X. Zhang, *Angew. Chem., Int. Ed.* **2022**, *61*, e202210103; *Angew. Chem.* **2022**, *134*, e202210103.
2. T. J. Cogswell, A. Dahlén, L. Knerr, *Chem. Eur. J.* **2019**, *25*, 1184-1187.
3. H. Song, R. Cheng, Q.-Q. Min, X. Zhang, *Org. Lett.* **2020**, *22*, 7747−7751.

8. Copies of ^{1}H, ^{19}F and ^{13}C NMR Spectra of Compounds S-3 and 2f.

^{1}H NMR spectrum of **S-3** (400 MHz, $CDCl_3$)

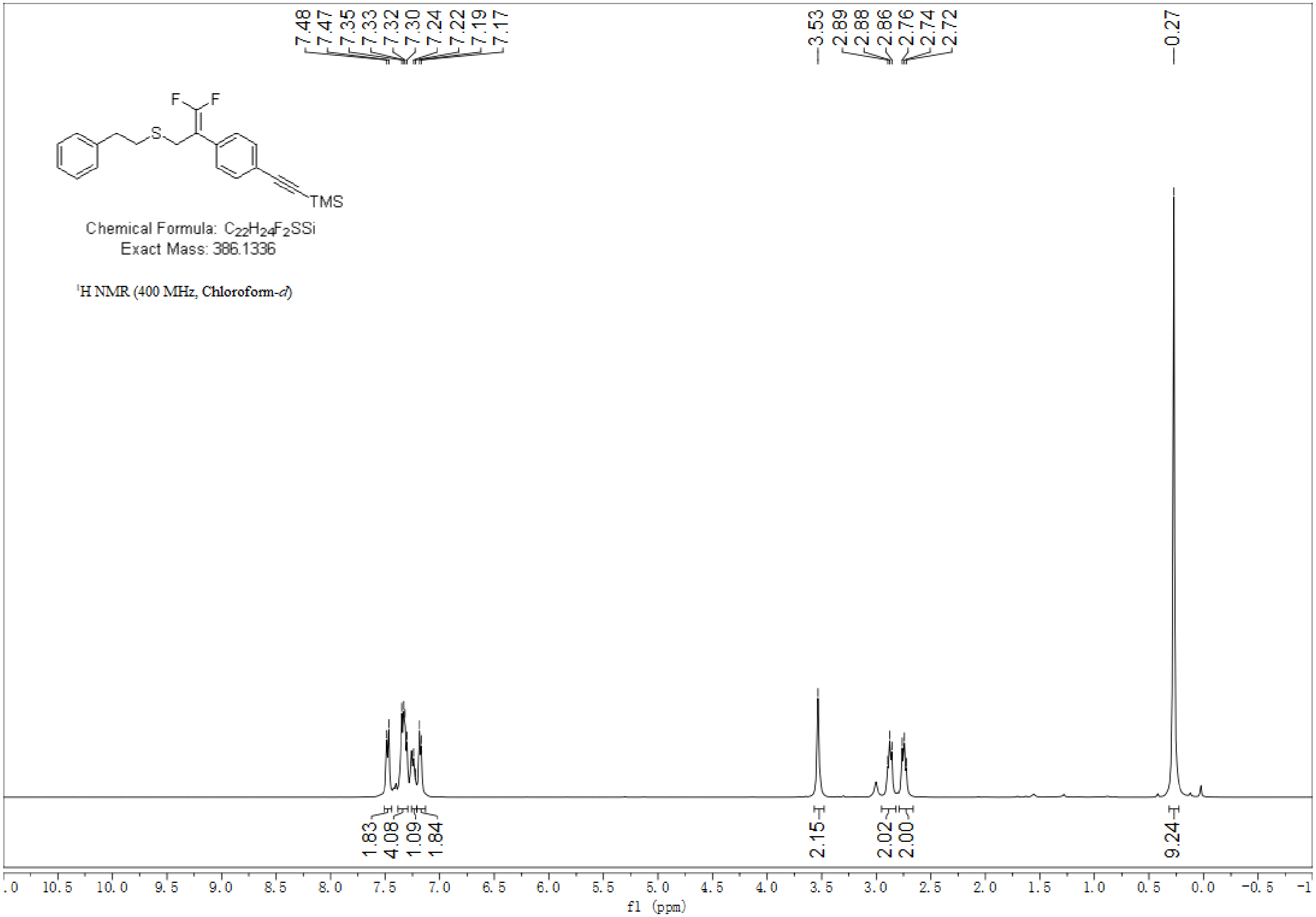

^{19}F NMR spectrum of **S-3** (376 MHz, $CDCl_3$)

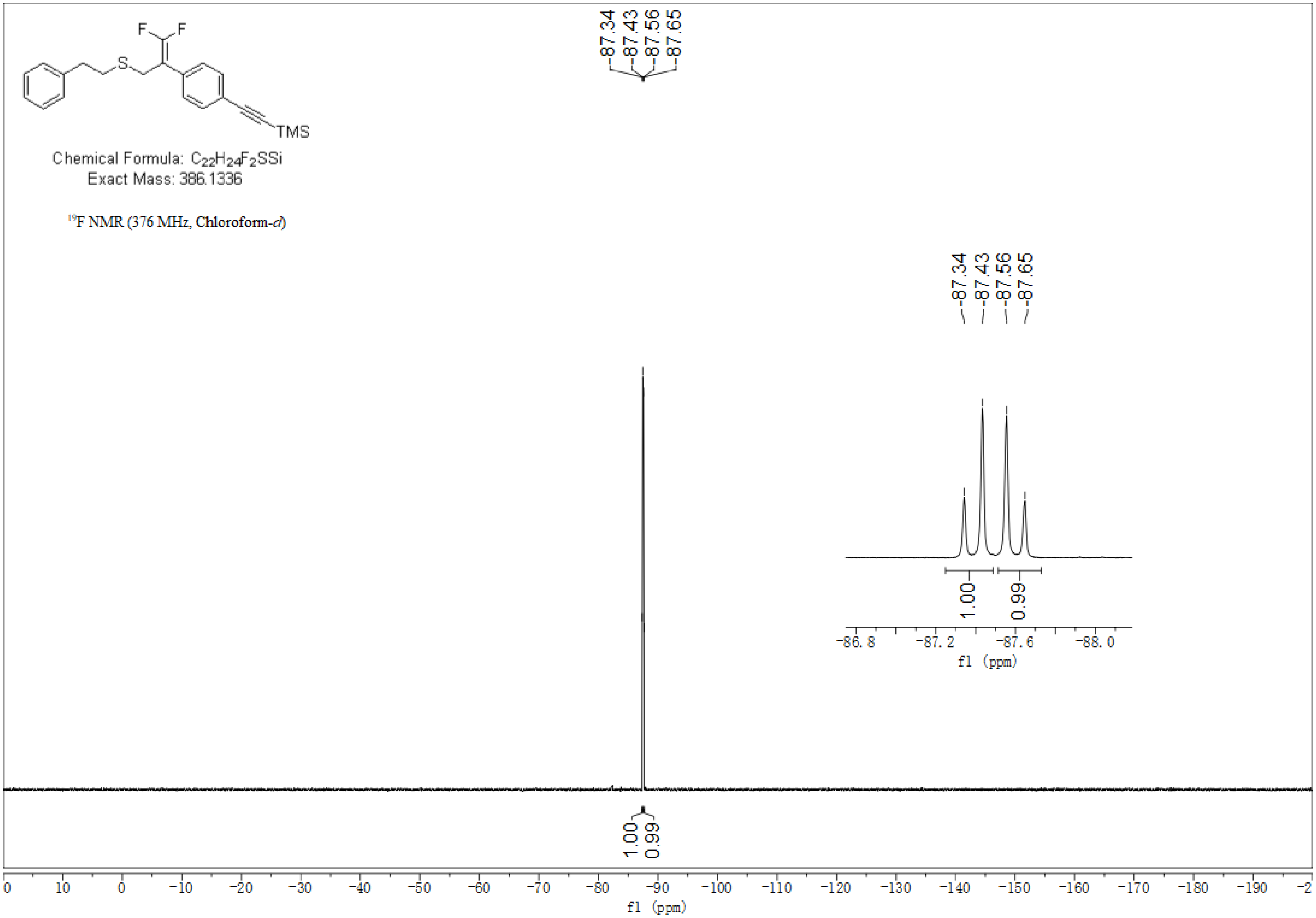

^{13}C NMR spectrum of **S-3** (126 MHz, $CDCl_3$)

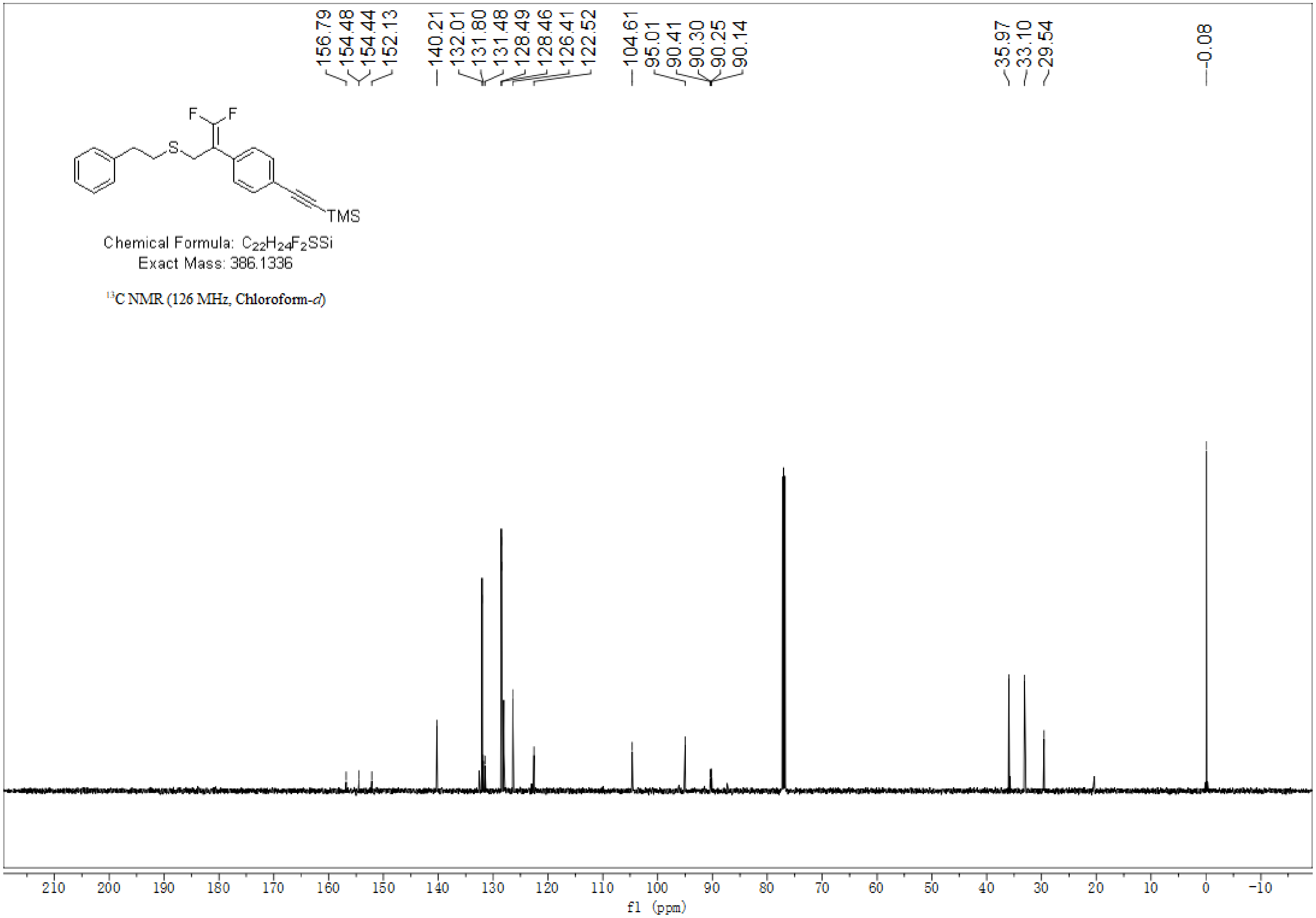

^{1}H NMR spectrum of **2f** (400 MHz, $CDCl_3$)

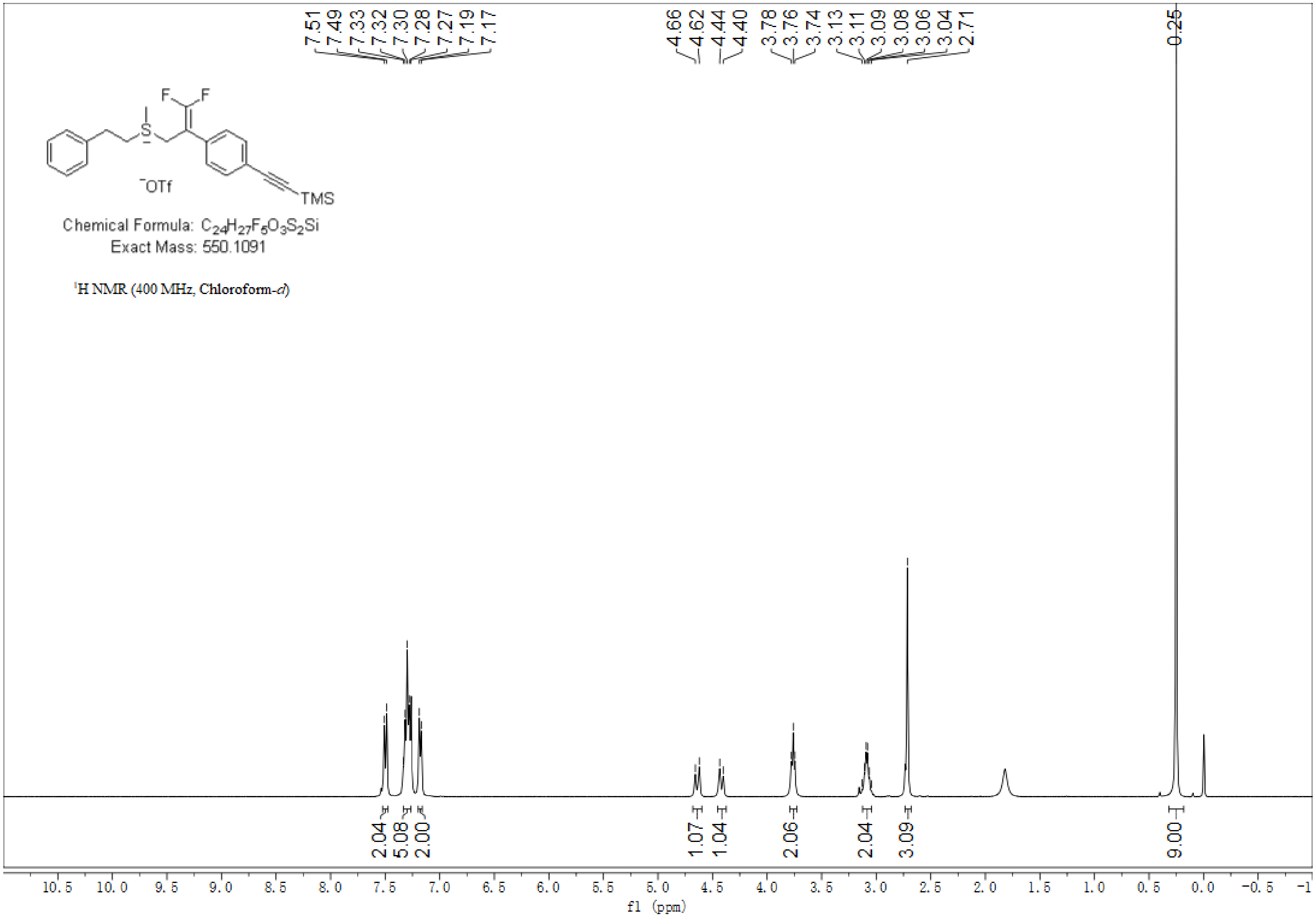

^{19}F NMR spectrum of **2f** (376 MHz, $CDCl_3$)

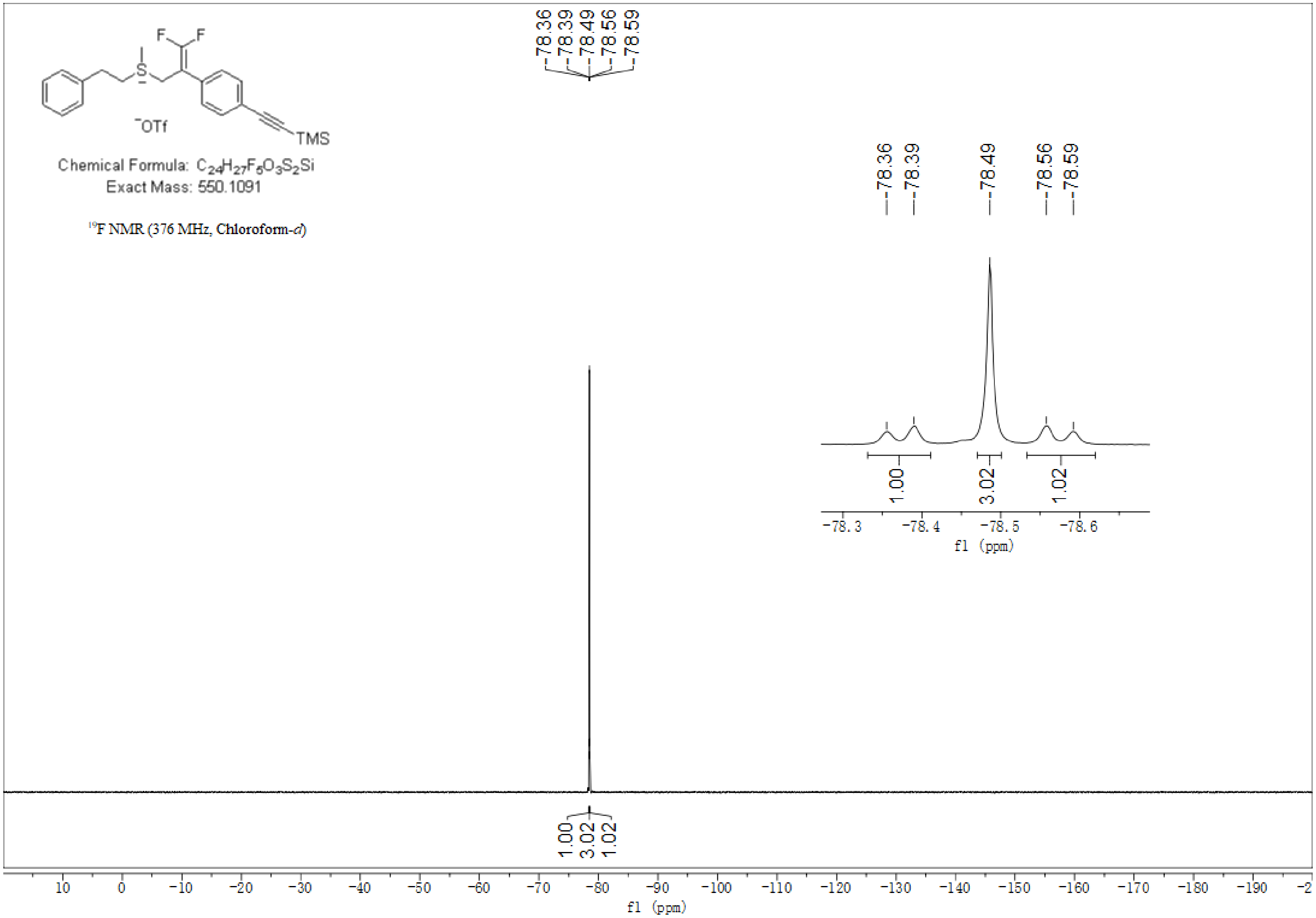

^{13}C NMR spectrum of **2f** (126 MHz, $CDCl_3$)

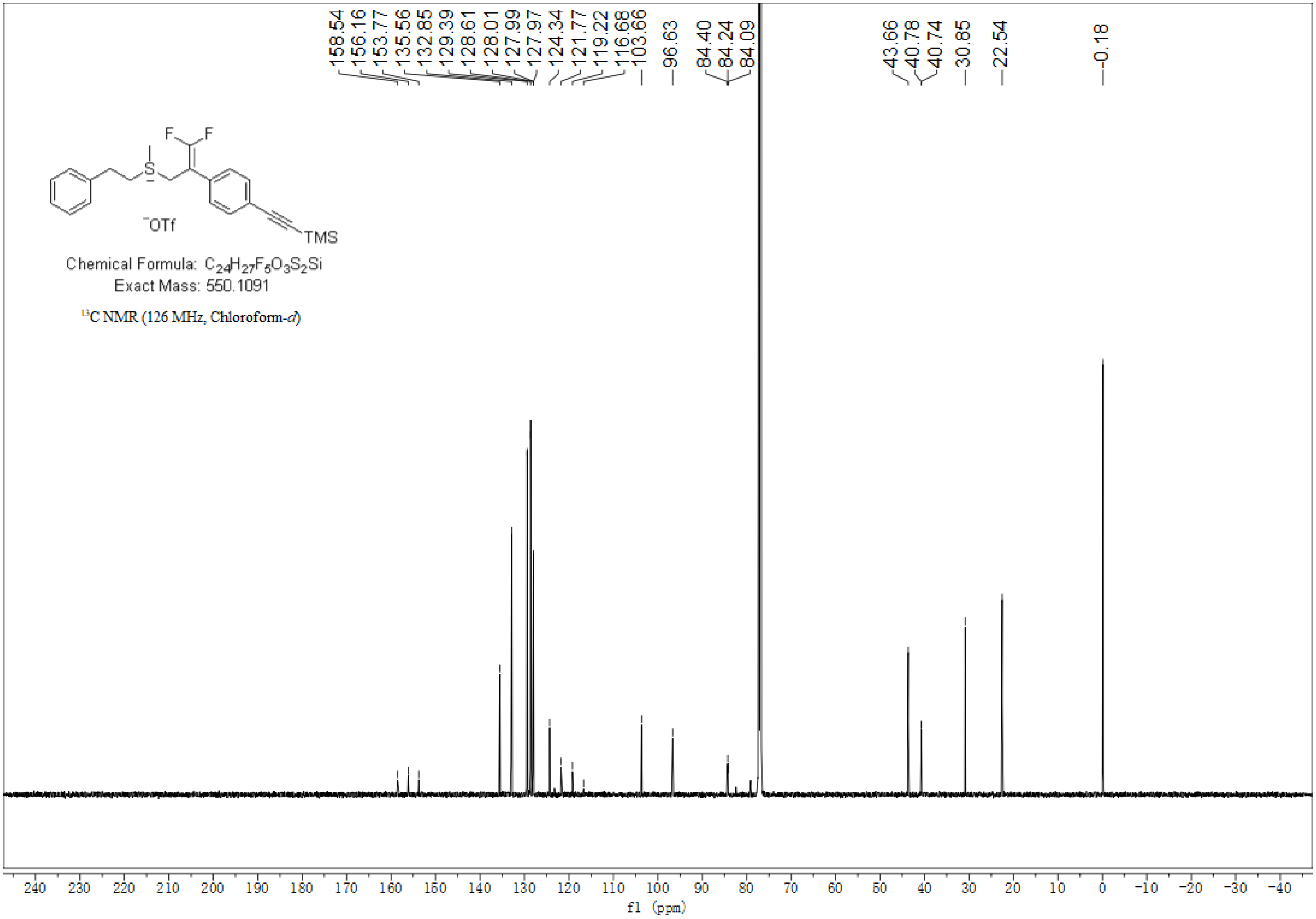

9. Copies of ^{1}H, ^{19}F, and ^{13}C NMR Spectra of Compounds 3-12.

^{1}H NMR spectrum of **3a** (400 MHz, $CDCl_3$)

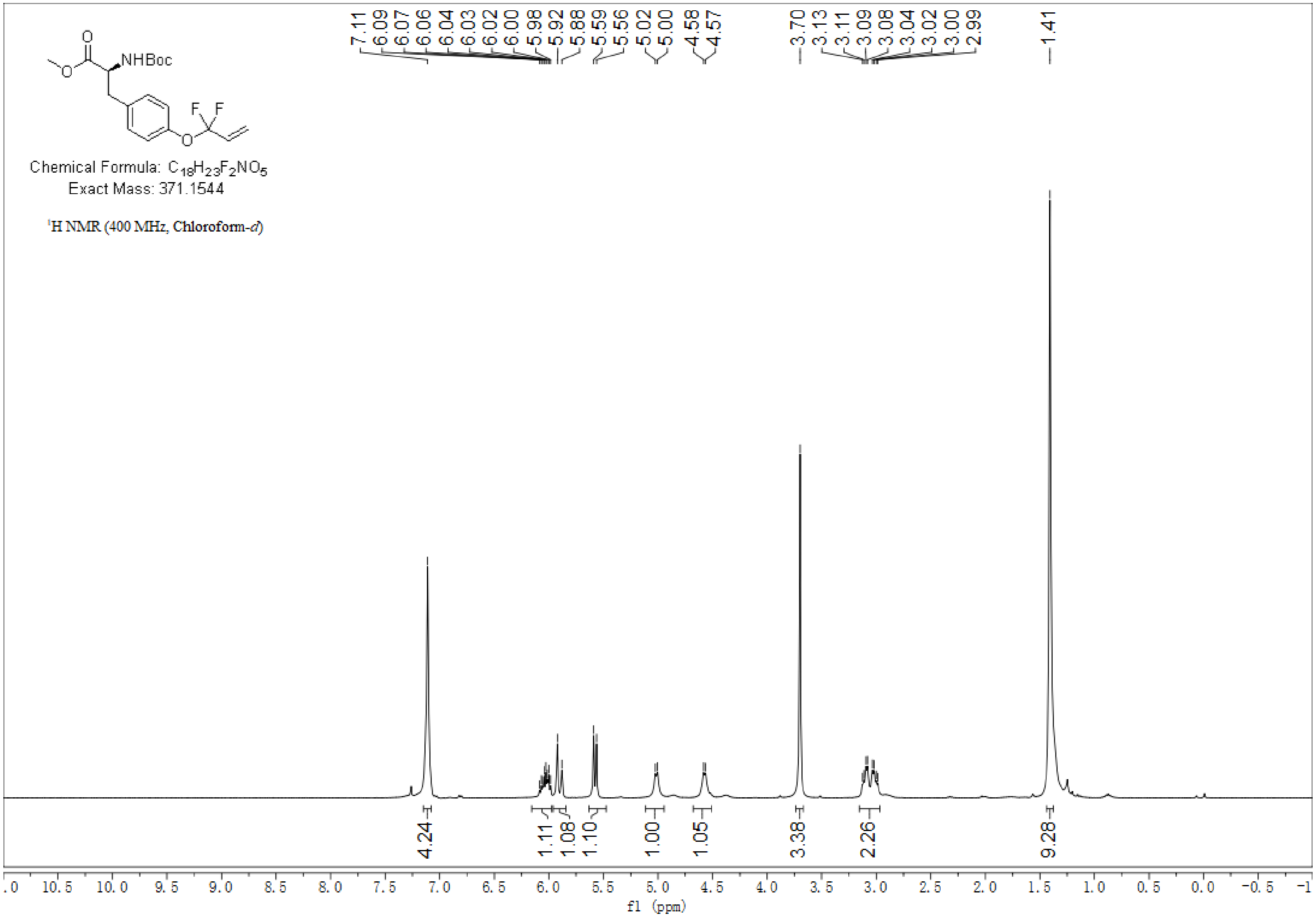

^{19}F NMR spectrum of **3a** (376 MHz, $CDCl_3$)

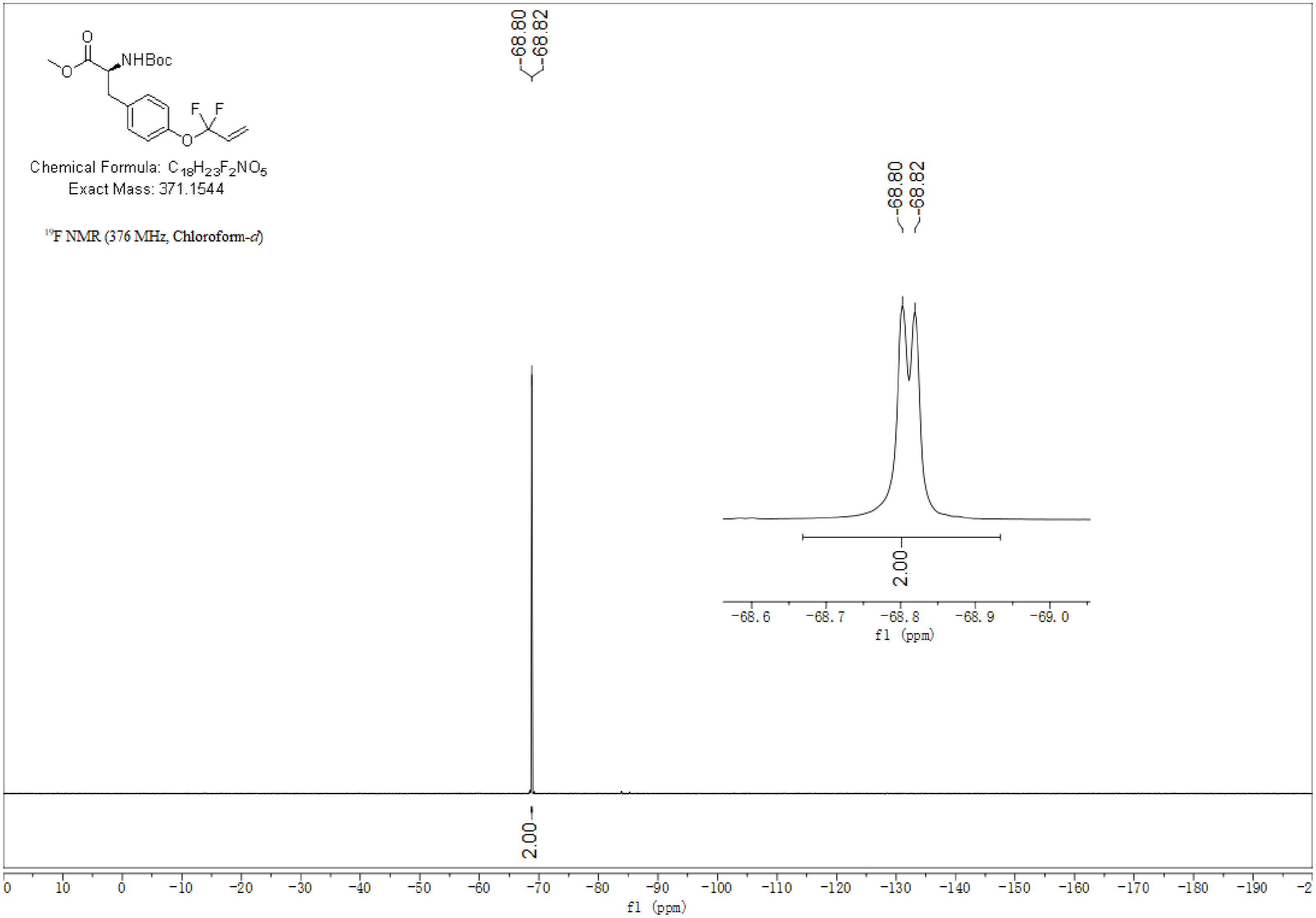

^{13}C NMR spectrum of **3a** (126 MHz, $CDCl_3$)

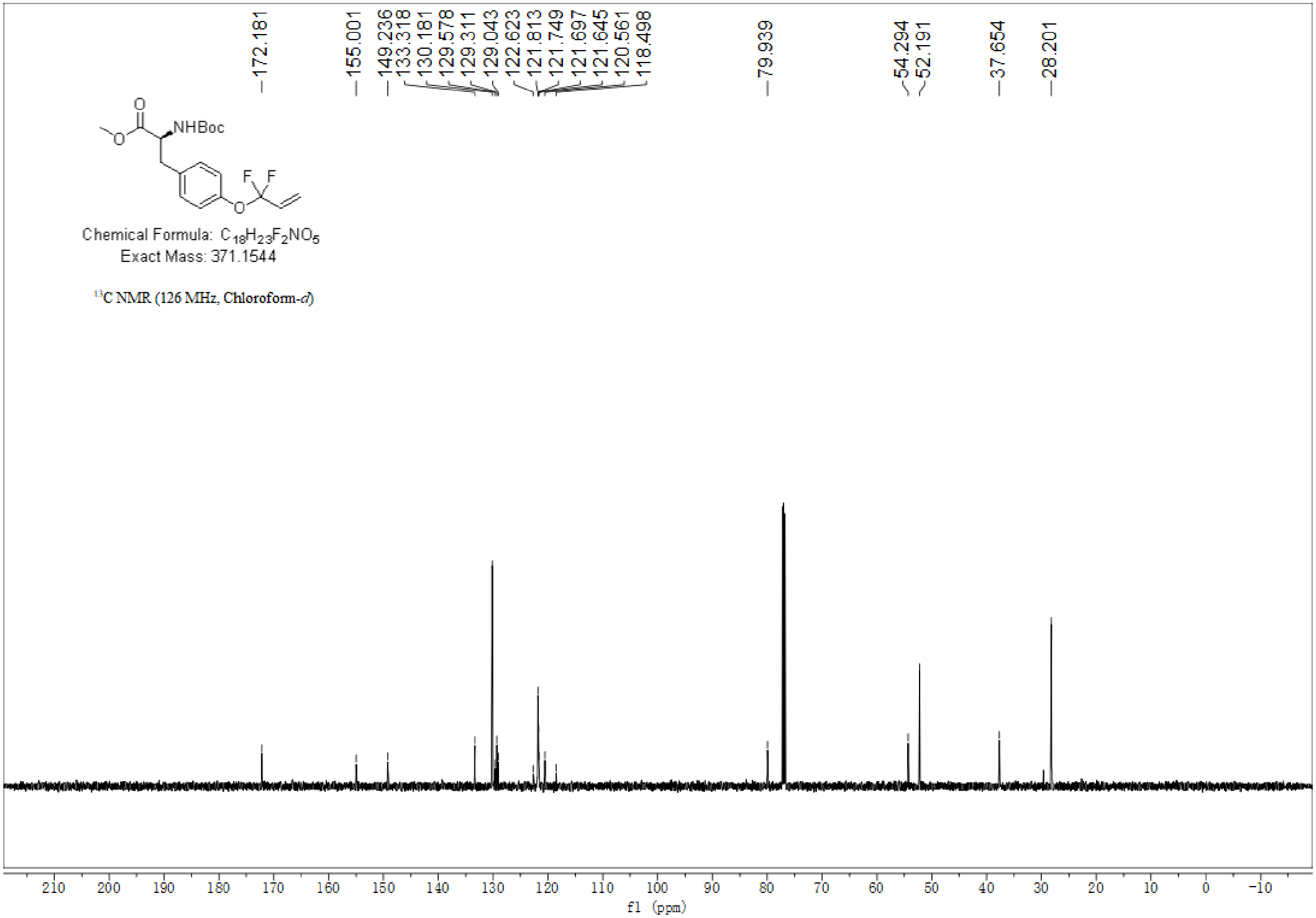

^1H NMR spectrum of **3b** (400 MHz, $CDCl_3$)

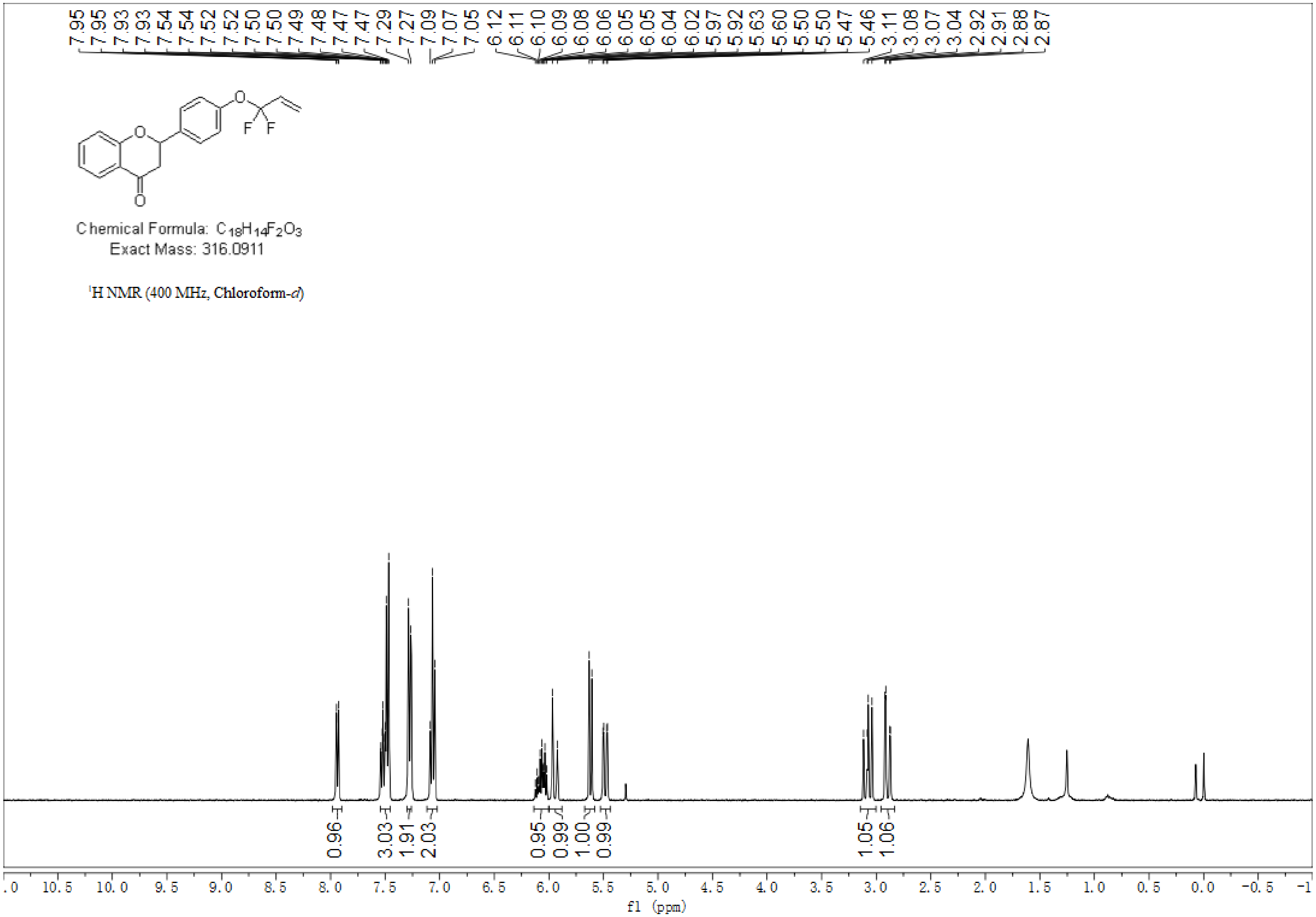

^{19}F NMR spectrum of **3b** (376 MHz, $CDCl_3$)

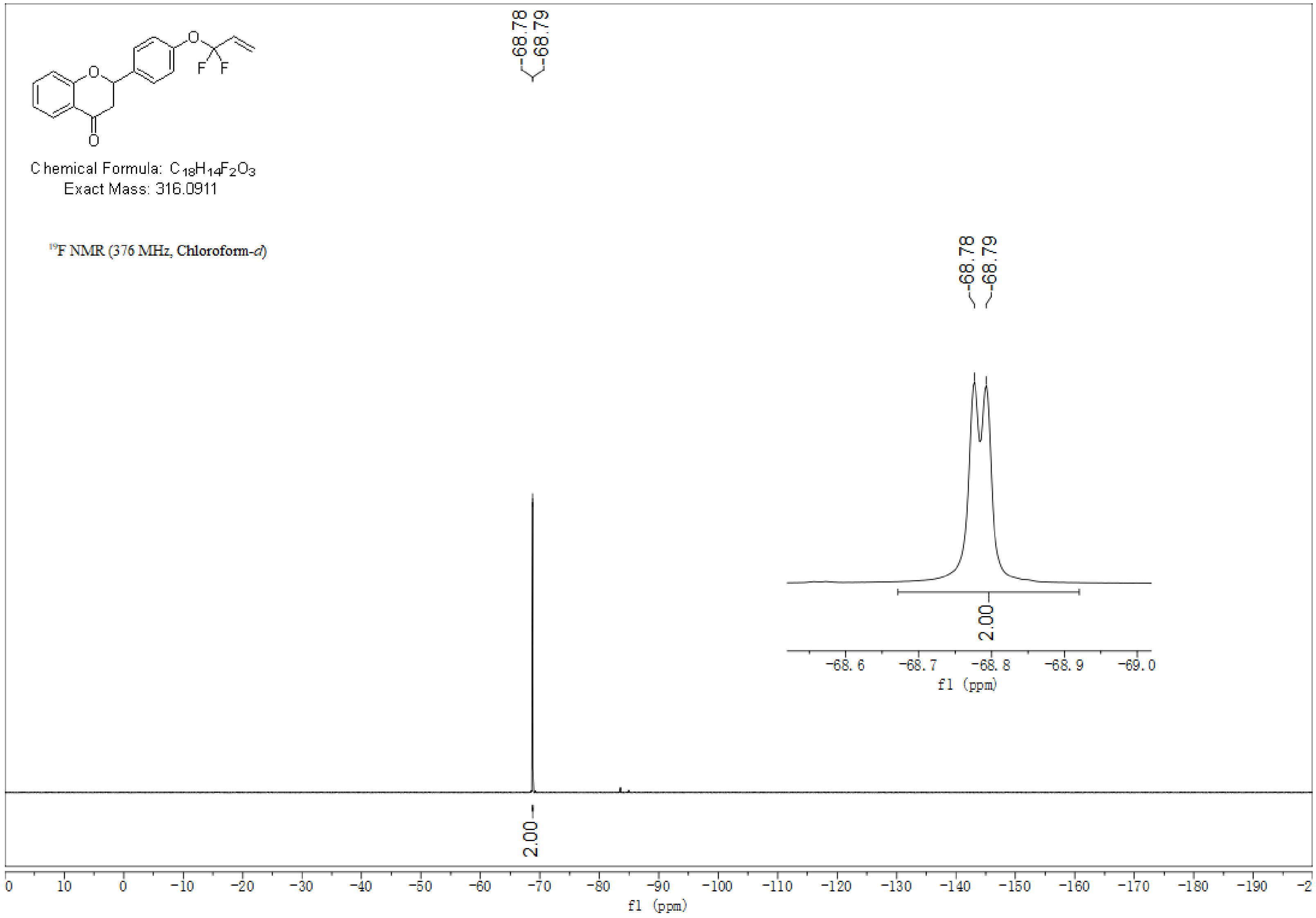

^{13}C NMR spectrum of **3b** (126 MHz, $CDCl_3$)

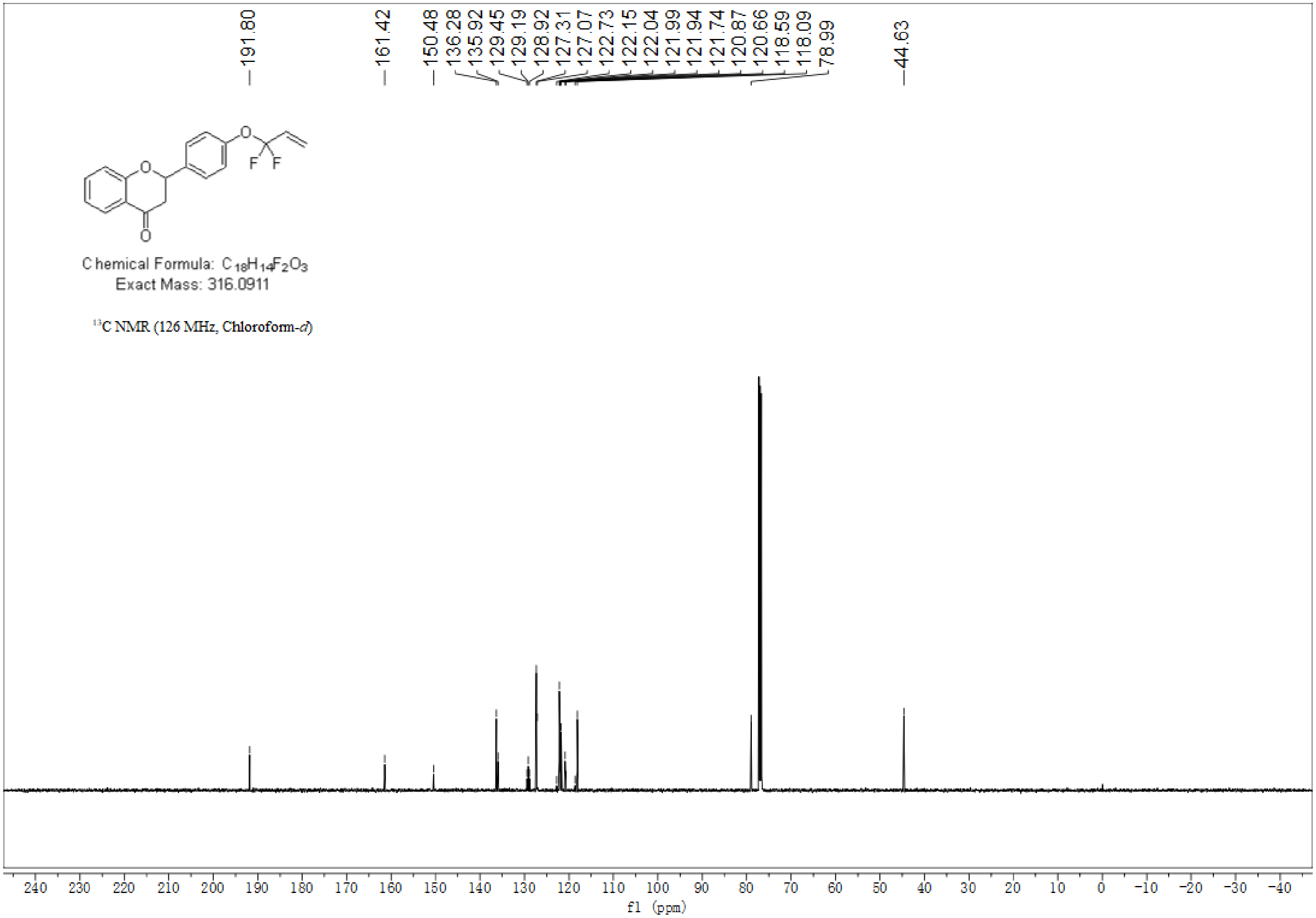

^{1}H NMR spectrum of **3c** (400 MHz, $CDCl_3$)

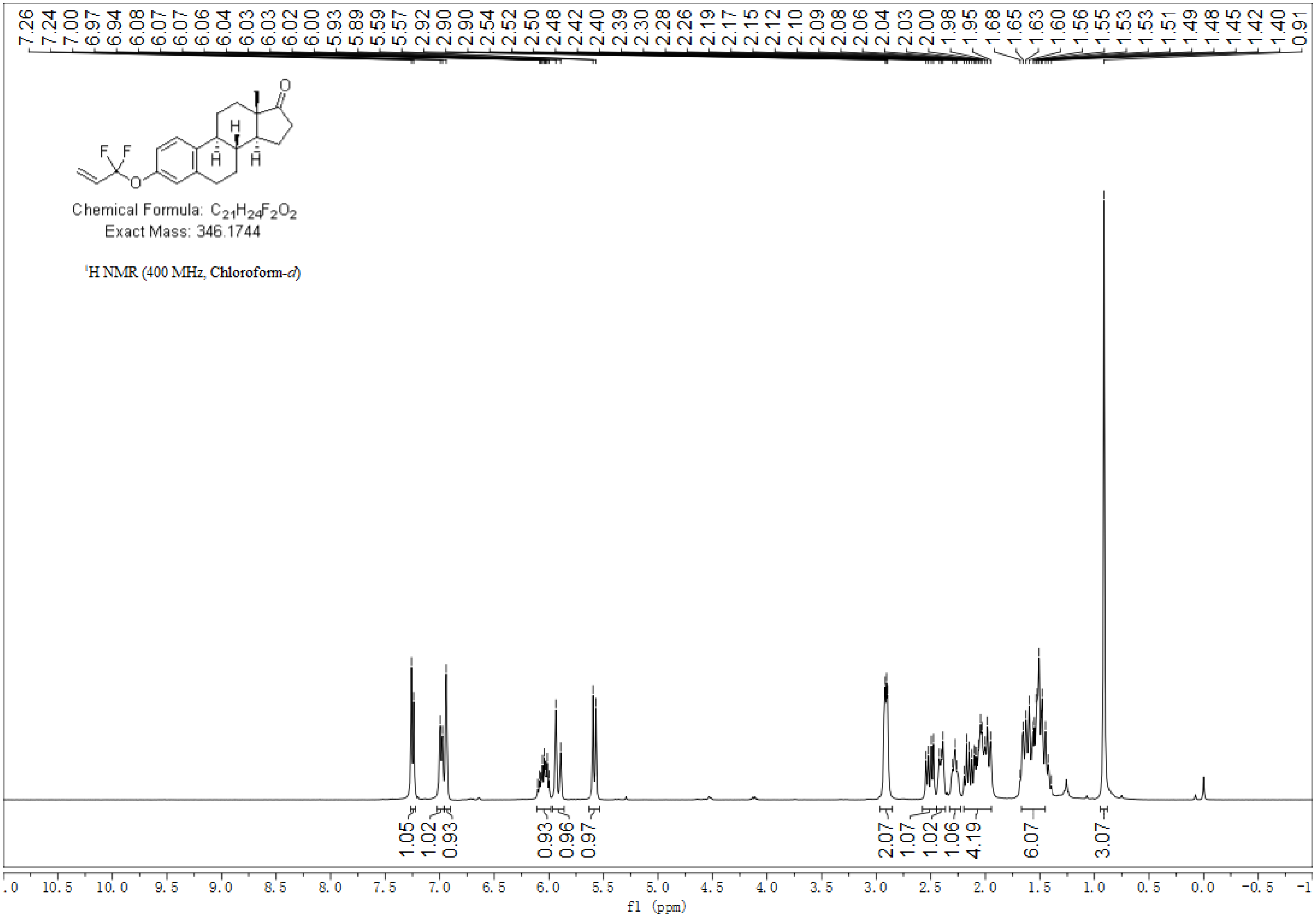

^{19}F NMR spectrum of **3c** (376 MHz, $CDCl_3$)

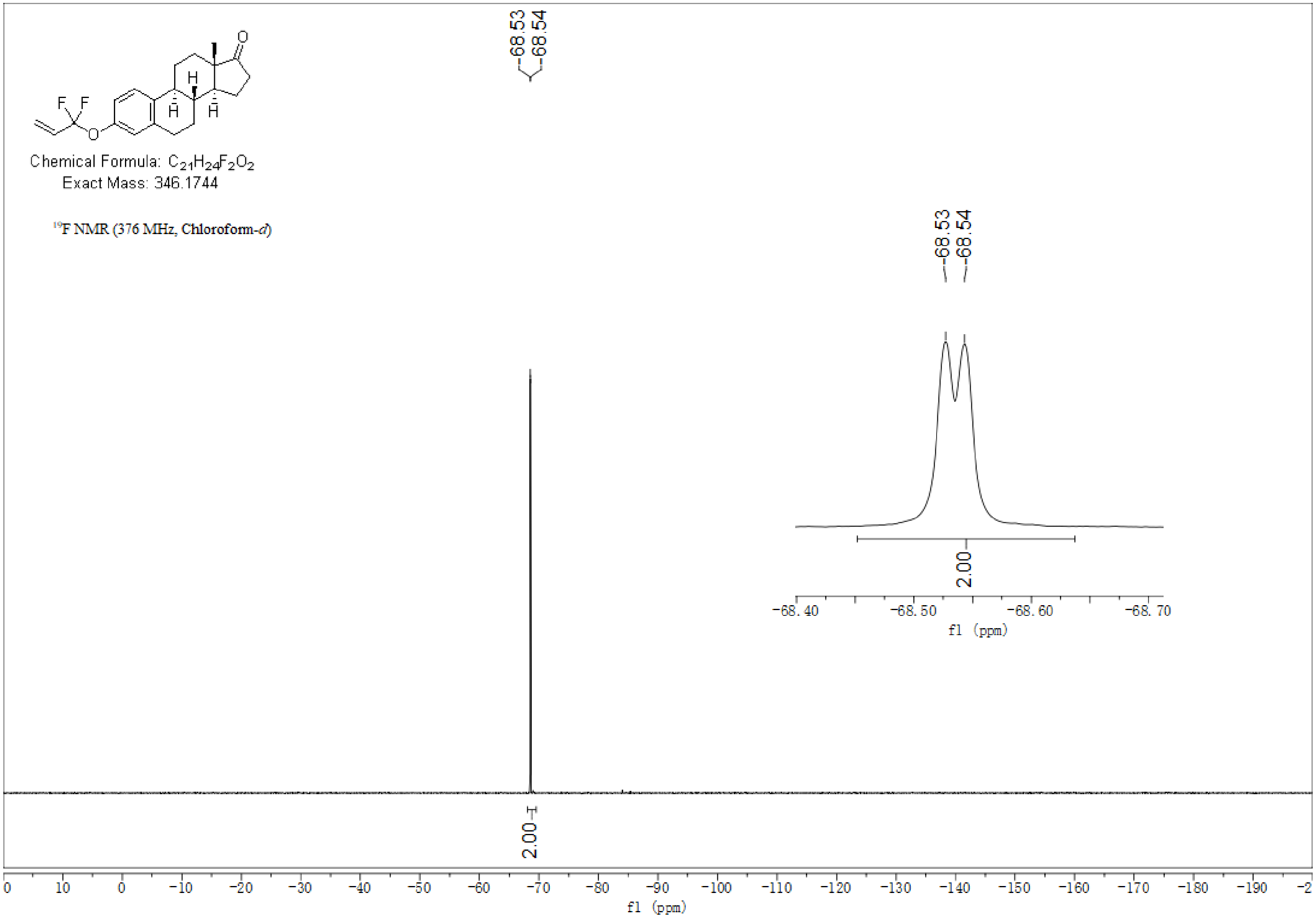

^{13}C NMR spectrum of **3c** (126 MHz, $CDCl_3$)

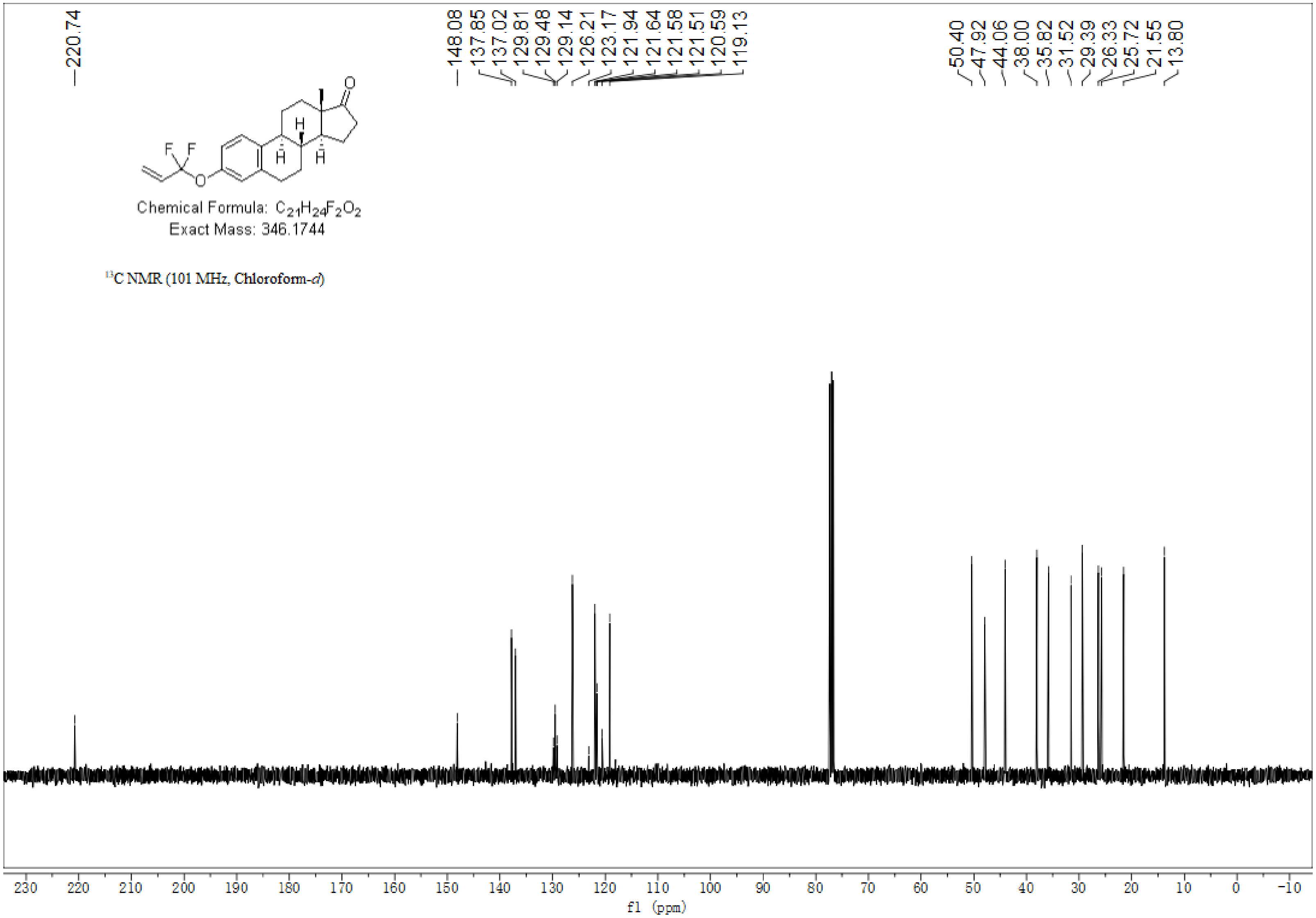

^{1}H NMR spectrum of **3d** (400 MHz, $CDCl_3$)

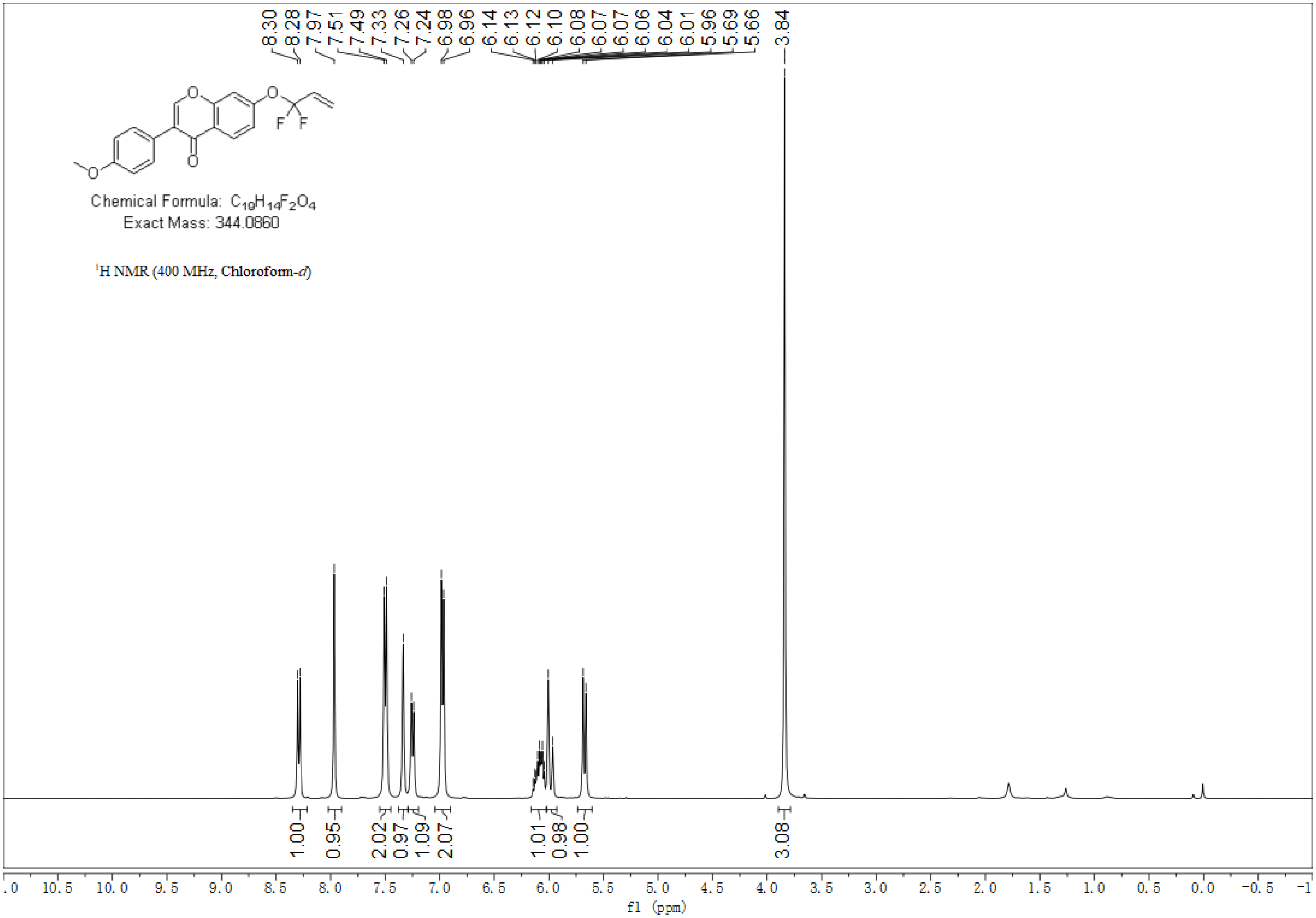

^{19}F NMR spectrum of **3d** (376 MHz, $CDCl_3$)

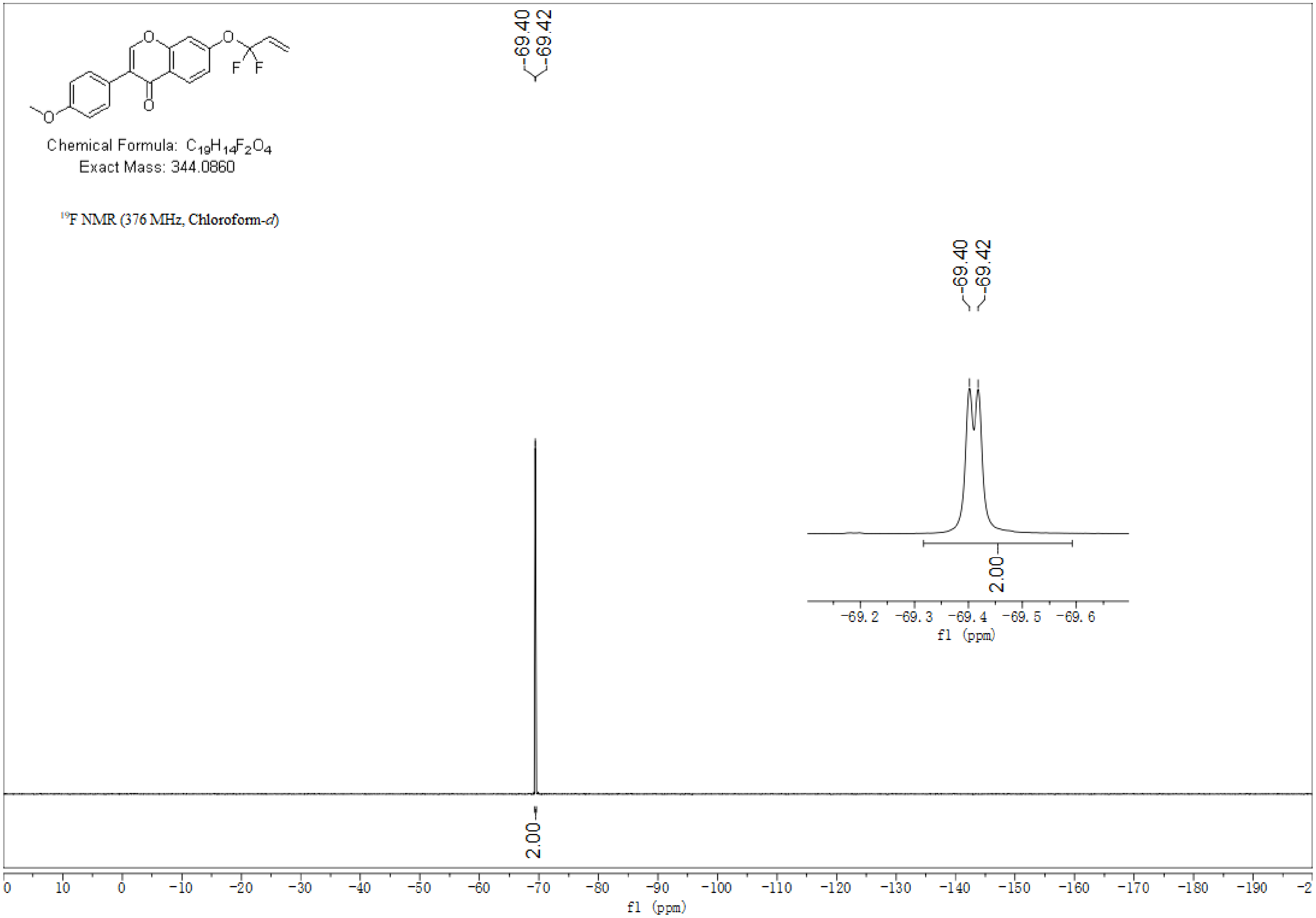

^{13}C NMR spectrum of **3d** (101 MHz, $CDCl_3$)

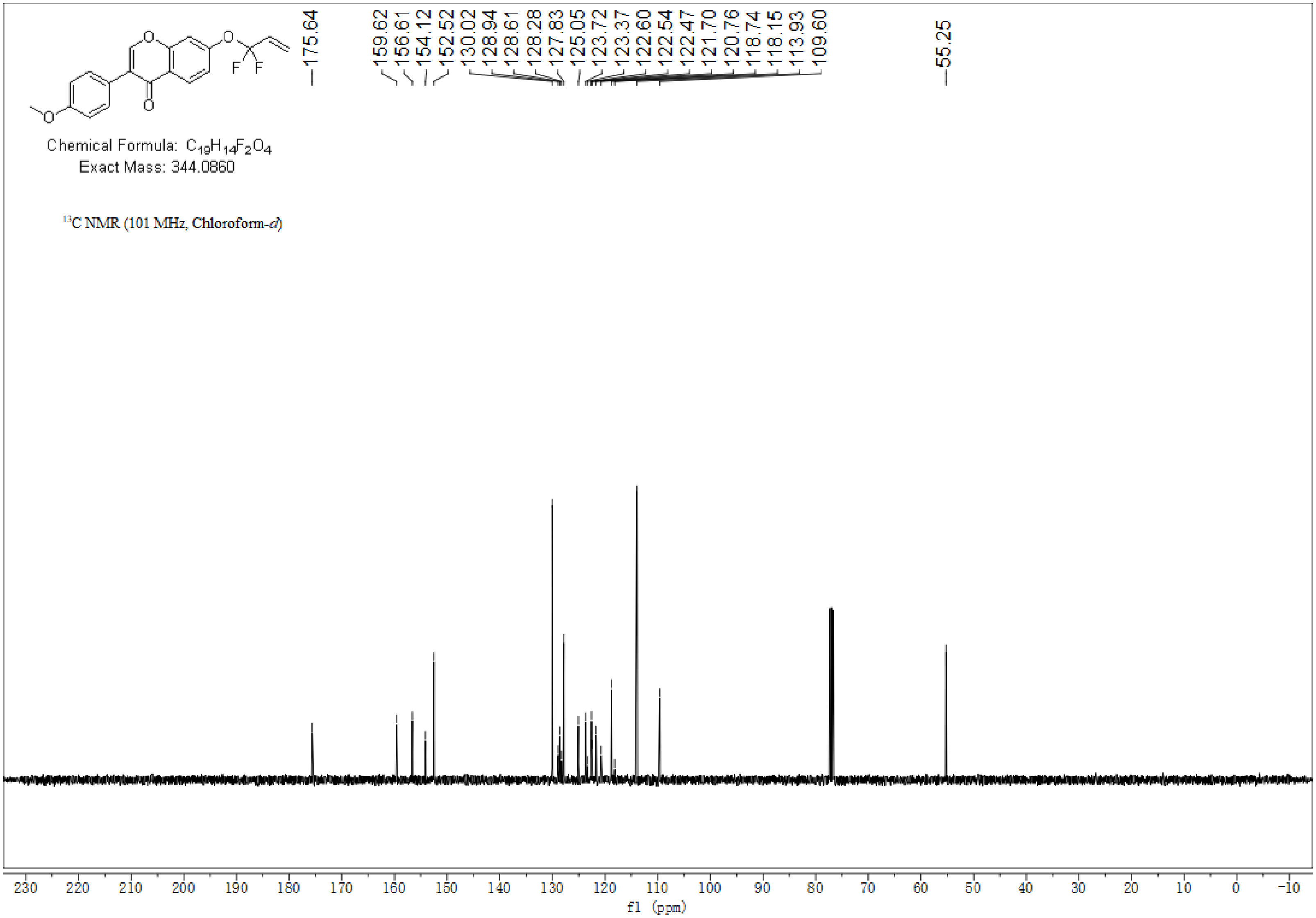

^1H NMR spectrum of **3e** (400 MHz, $CDCl_3$)

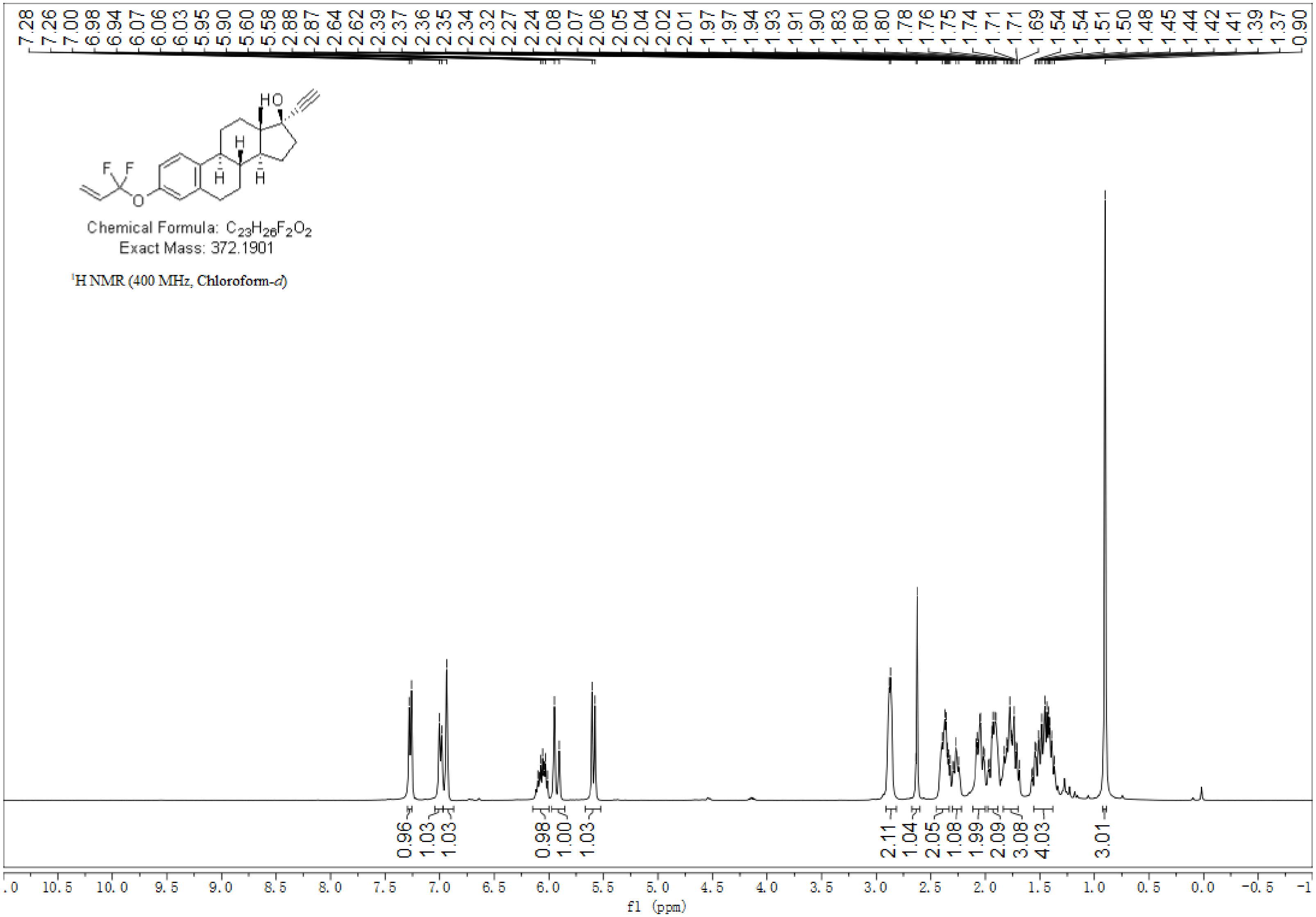

^{19}F NMR spectrum of **3e** (376 MHz, $CDCl_3$)

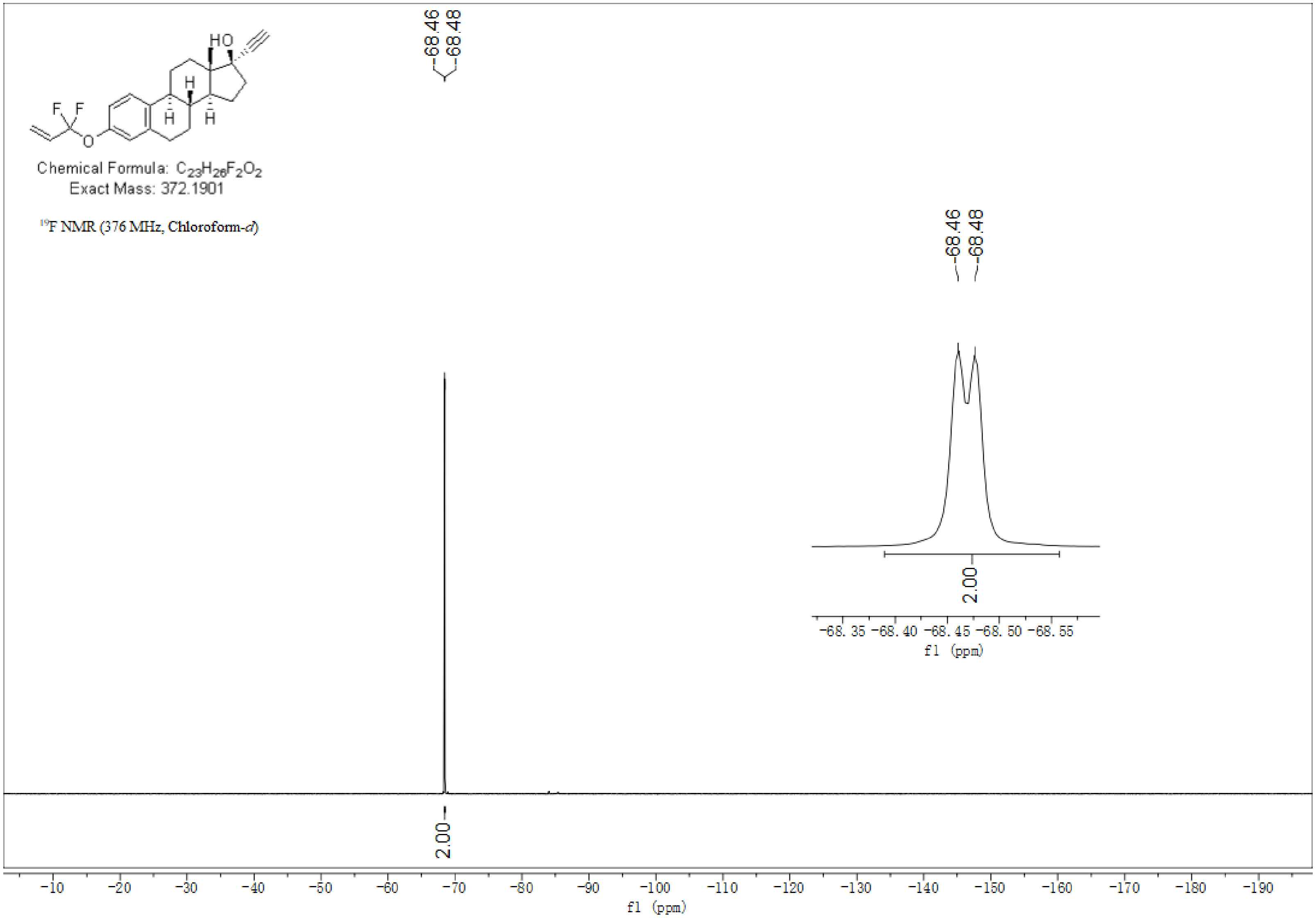

^{13}C NMR spectrum of **3e** (101 MHz, $CDCl_3$)

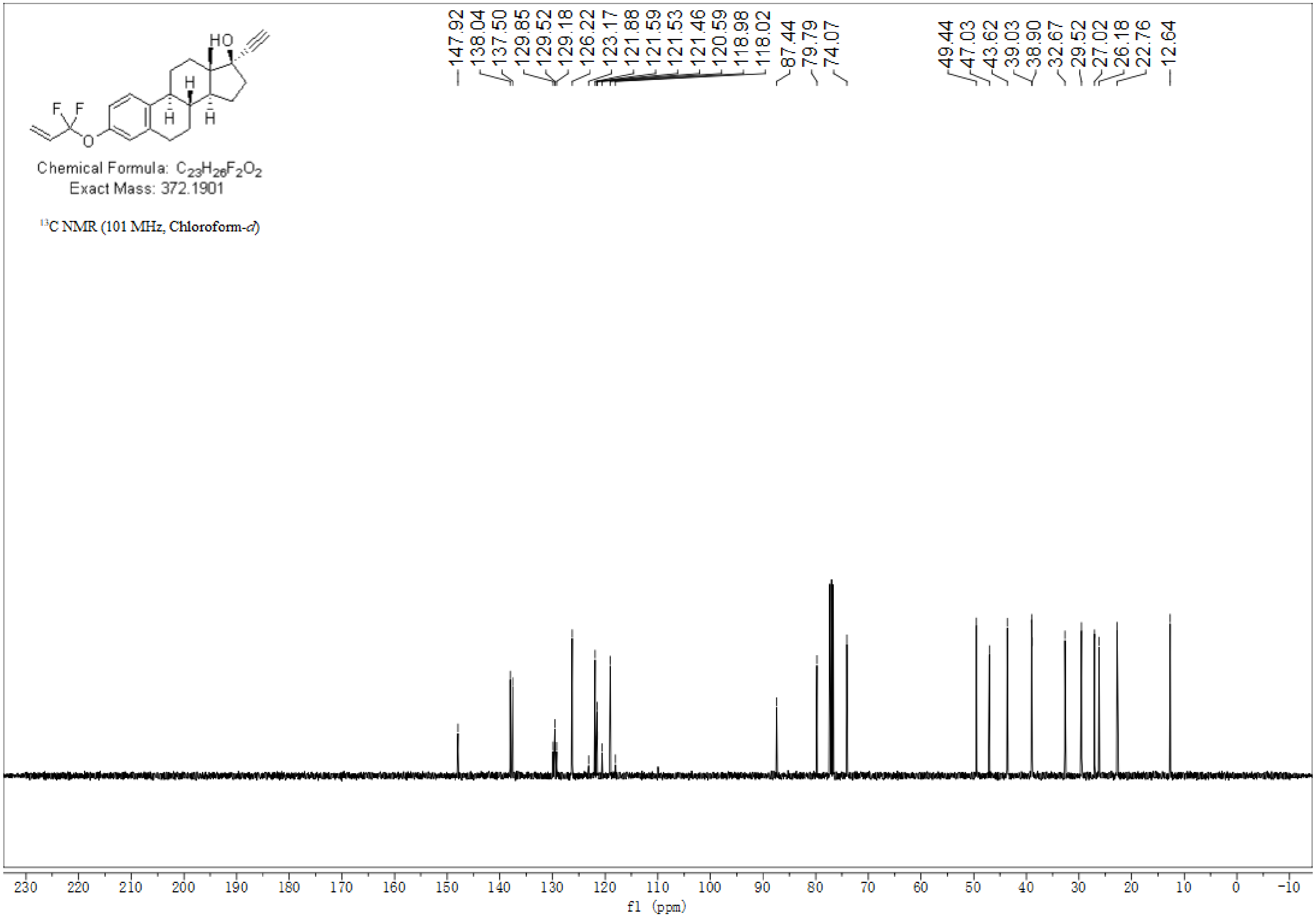

^{1}H NMR spectrum of **3f** (400 MHz, $CDCl_3$)

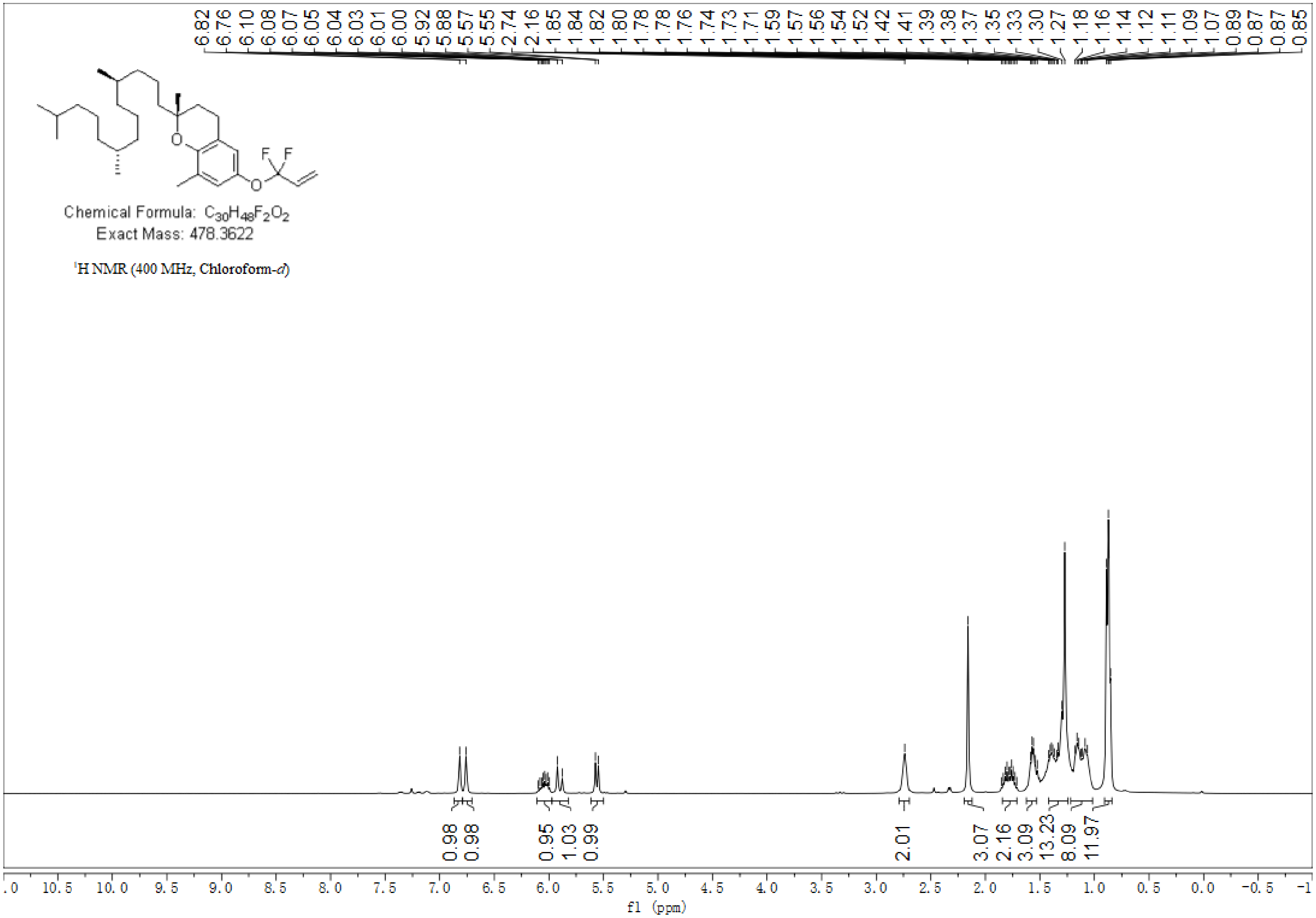

^{19}F NMR spectrum of **3f** (376 MHz, $CDCl_3$)

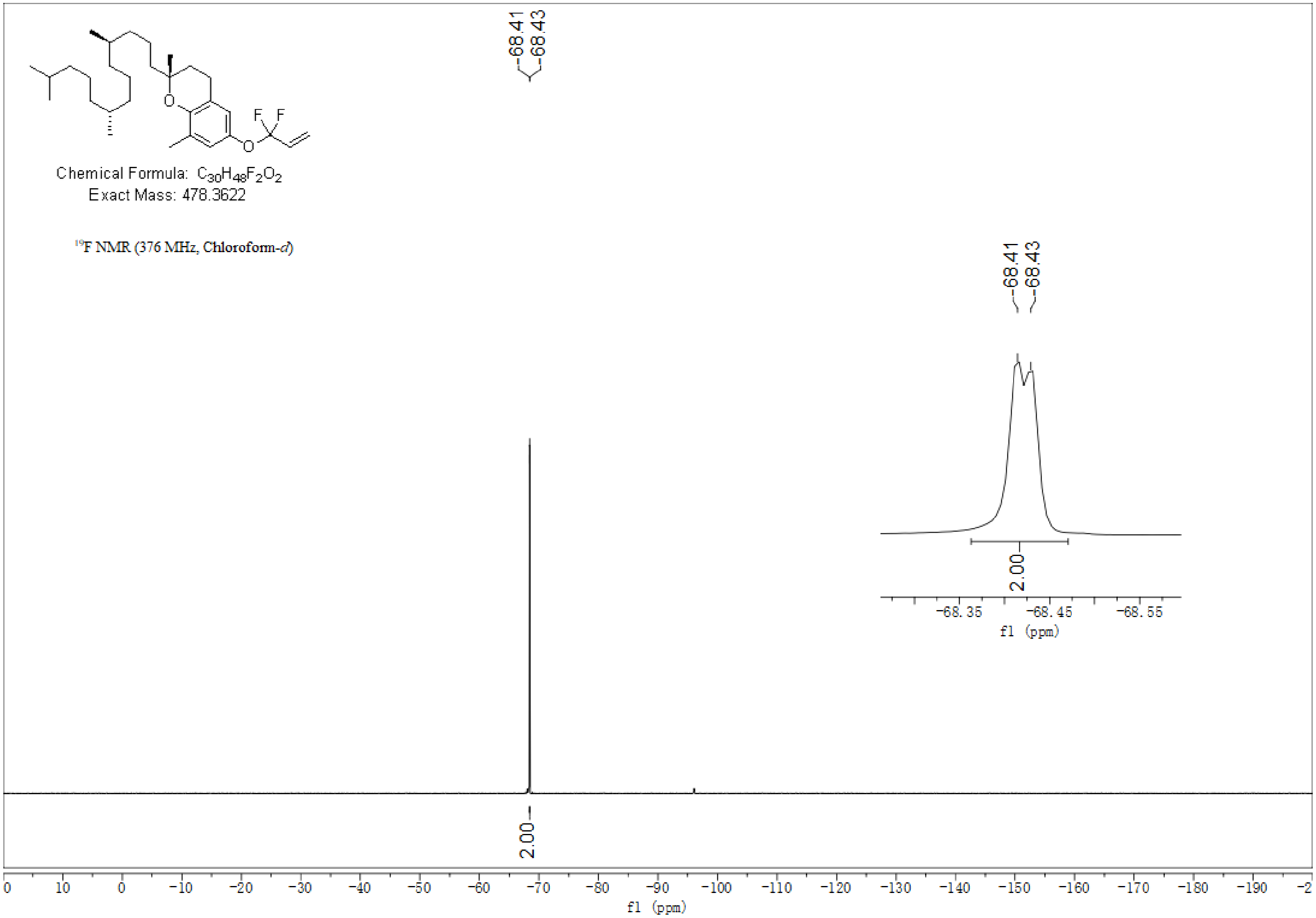

^{13}C NMR spectrum of **3f** (126 MHz, $CDCl_3$)

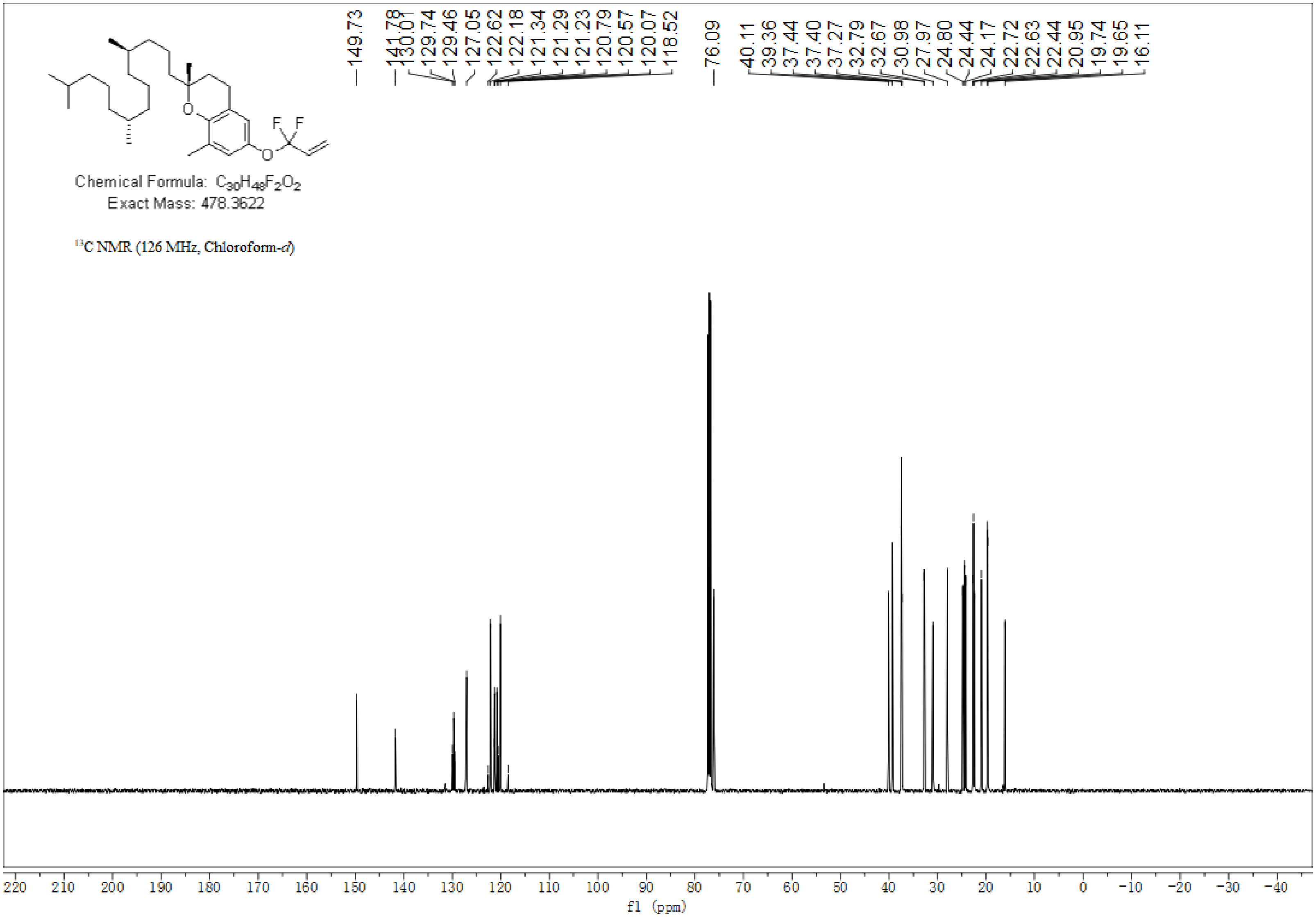

^{1}H NMR spectrum of **3g** (400 MHz, $CDCl_3$)

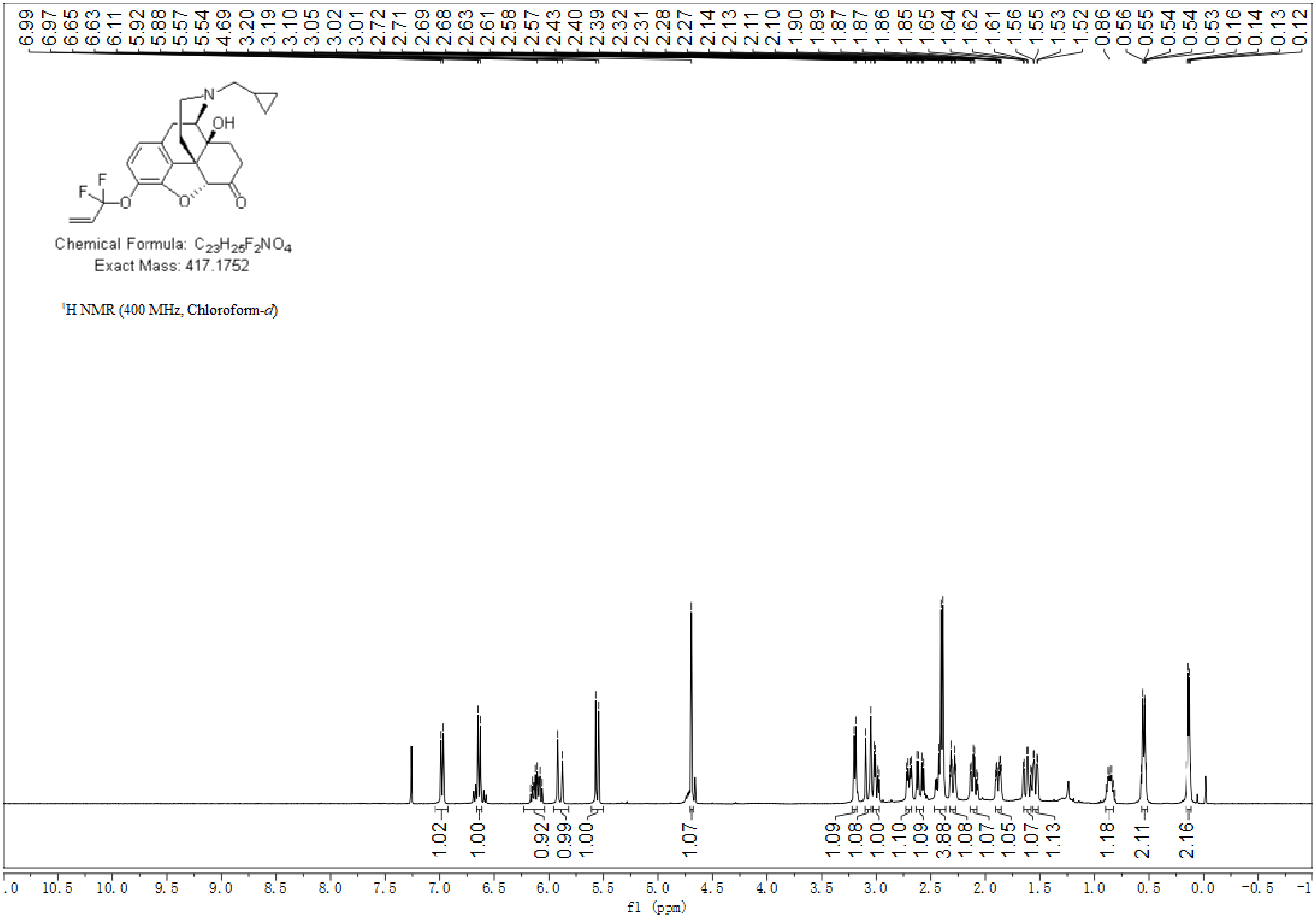

^{19}F NMR spectrum of **3g** (376 MHz, $CDCl_3$)

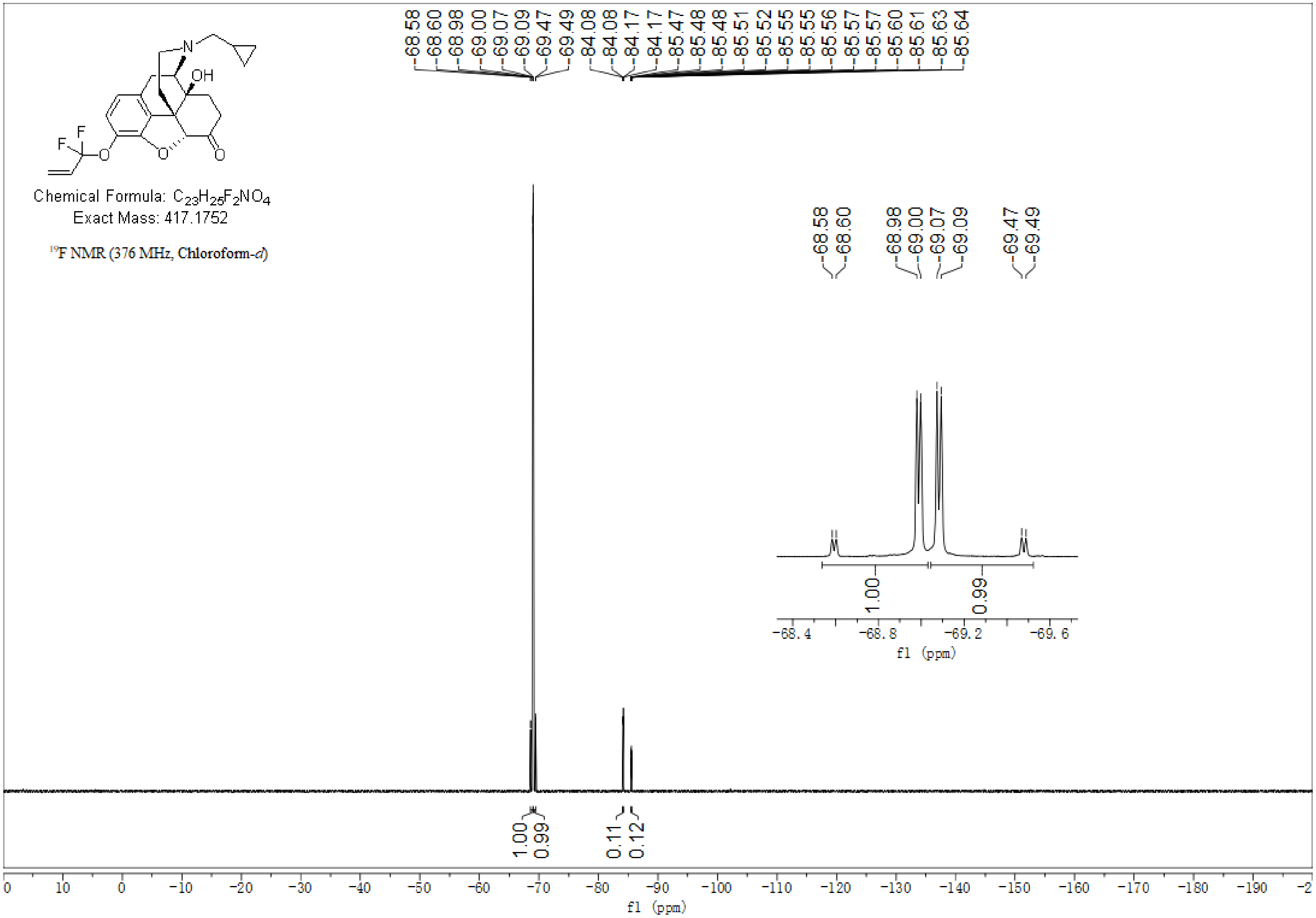

^{13}C NMR spectrum of **3g** (126 MHz, $CDCl_3$)

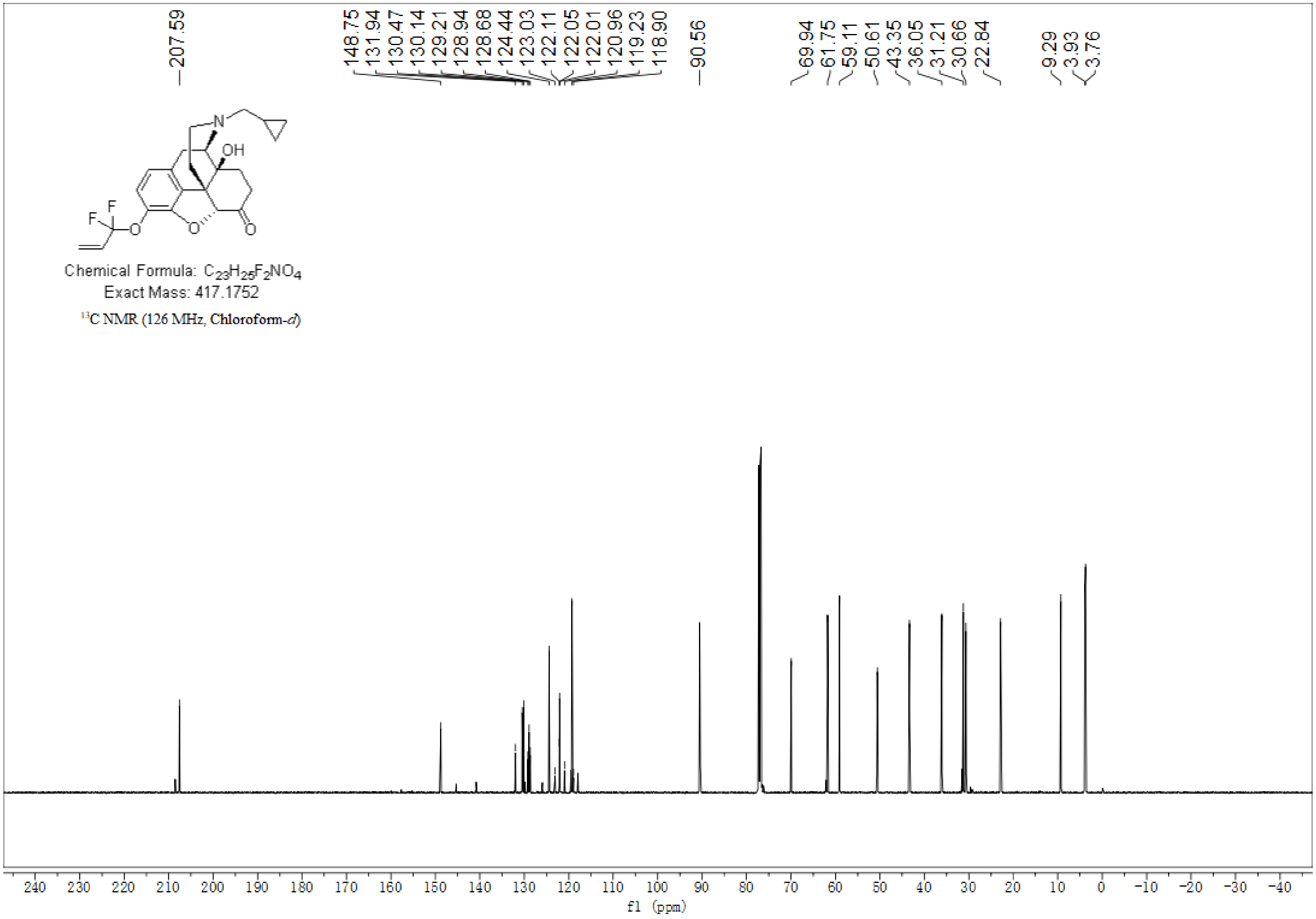

^{1}H NMR spectrum of **3h** (400 MHz, $CDCl_3$)

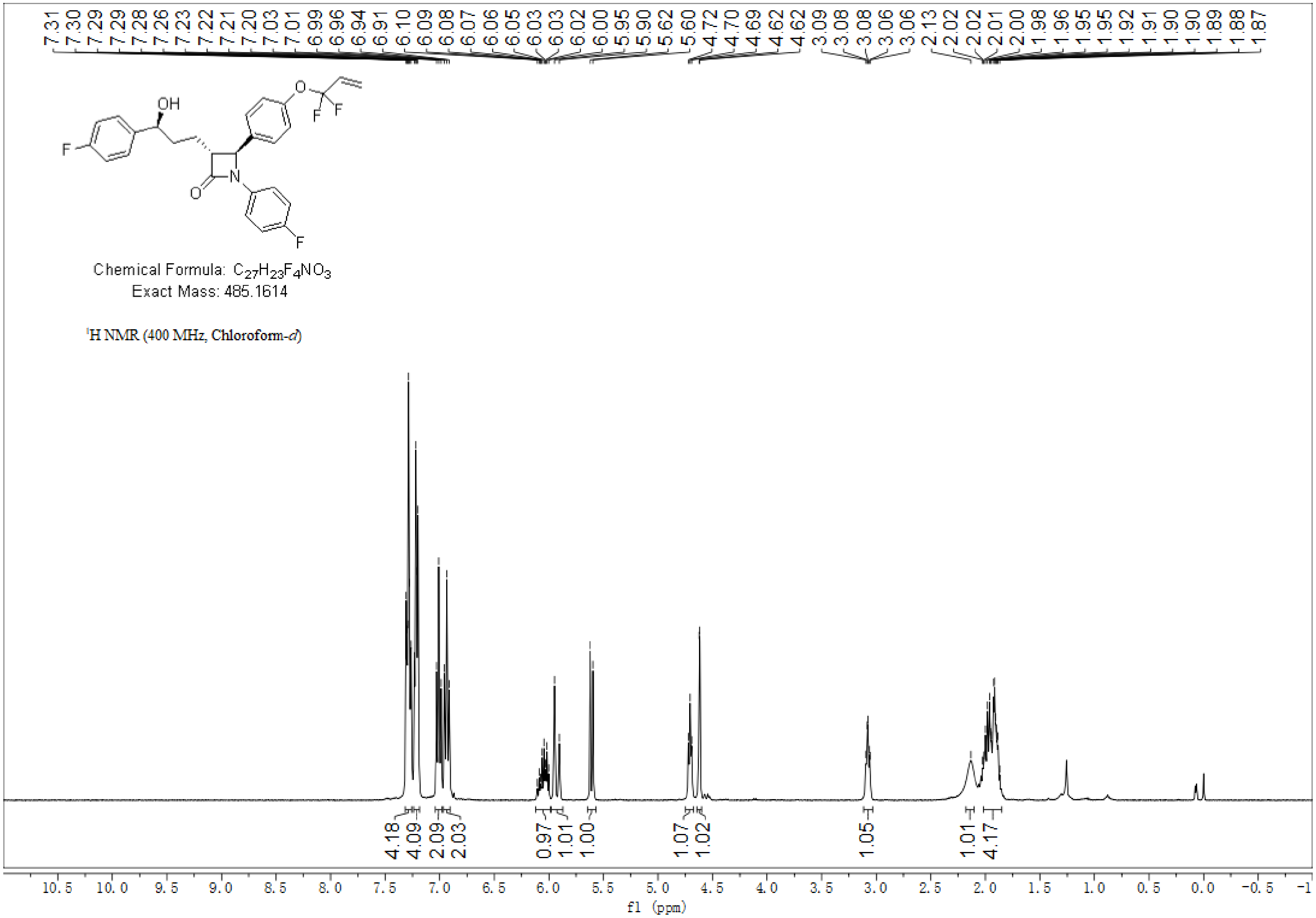

^{19}F NMR spectrum of **3h** (376 MHz, $CDCl_3$)

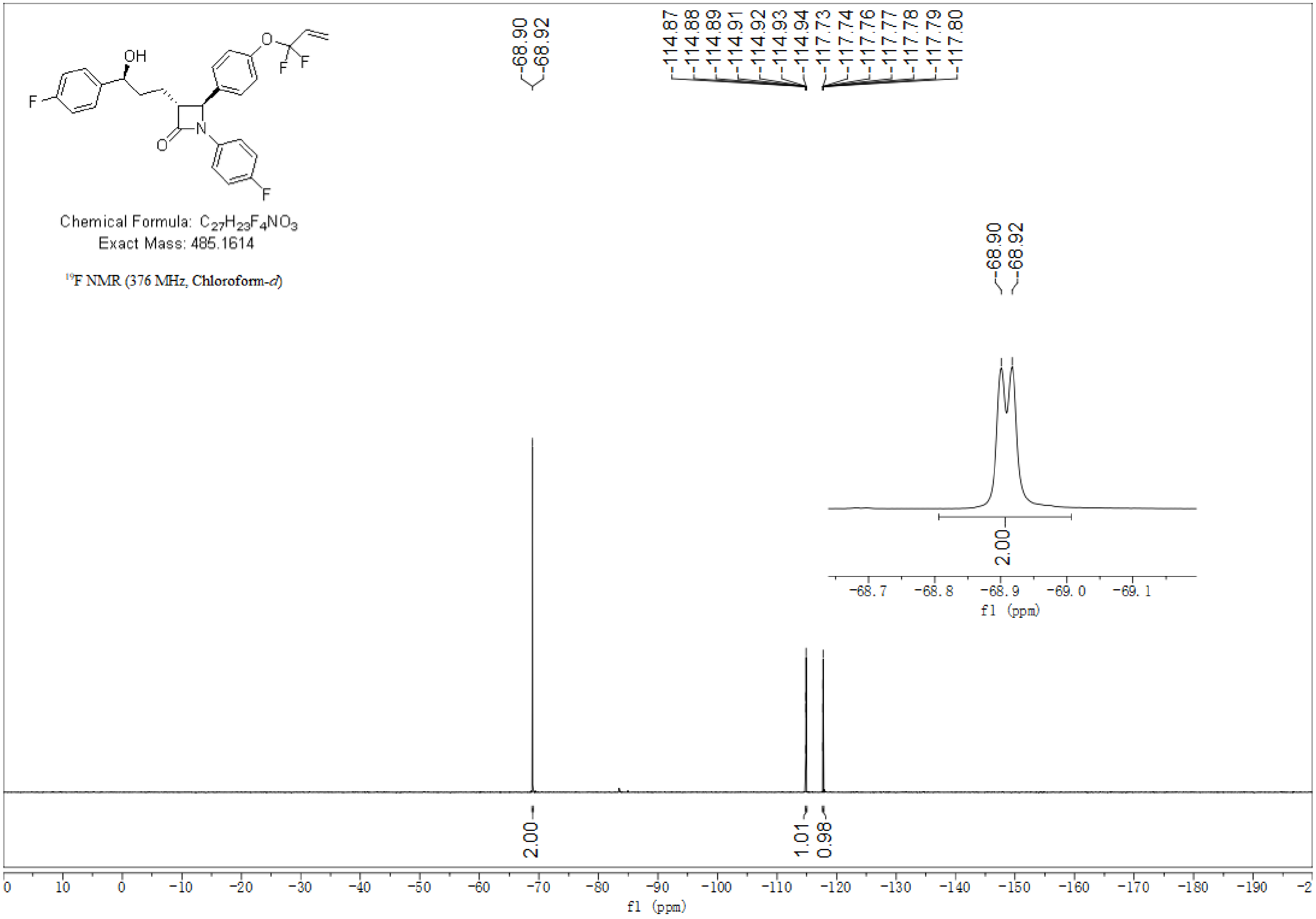

^{13}C NMR spectrum of **3h** (101 MHz, $CDCl_3$)

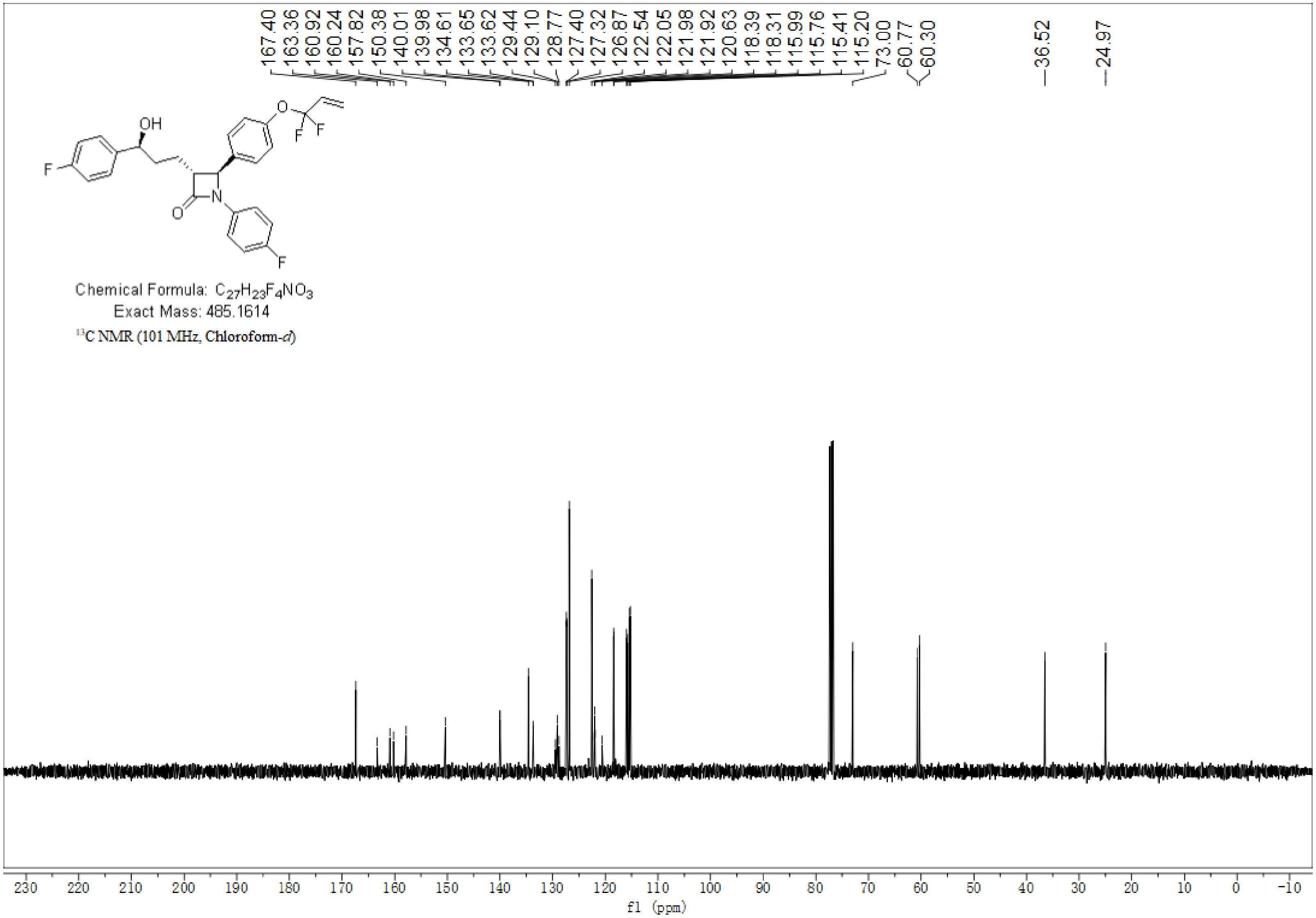

^{1}H NMR spectrum of **3i** (400 MHz, $CDCl_3$)

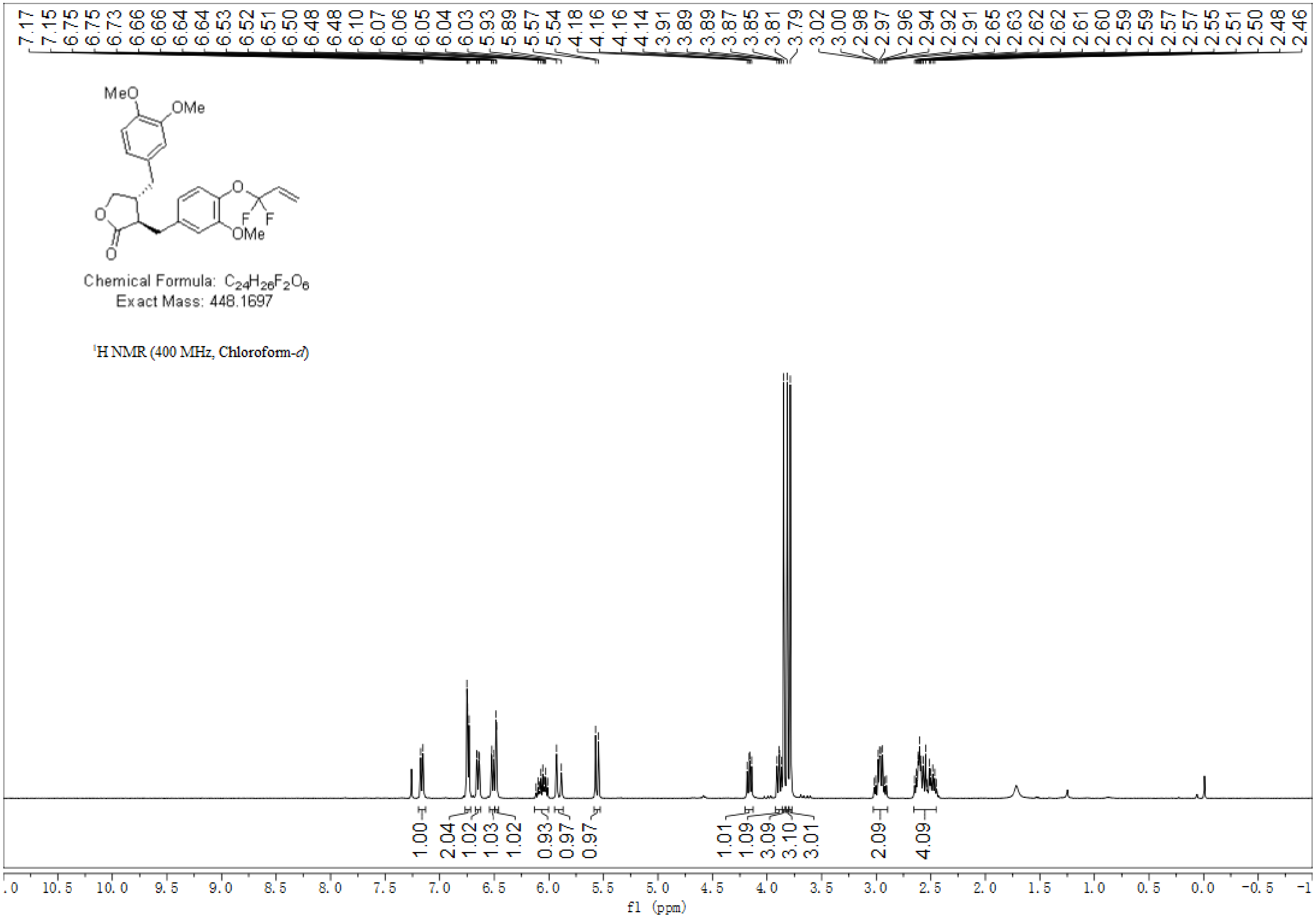

^{19}F NMR spectrum of **3i** (376 MHz, $CDCl_3$)

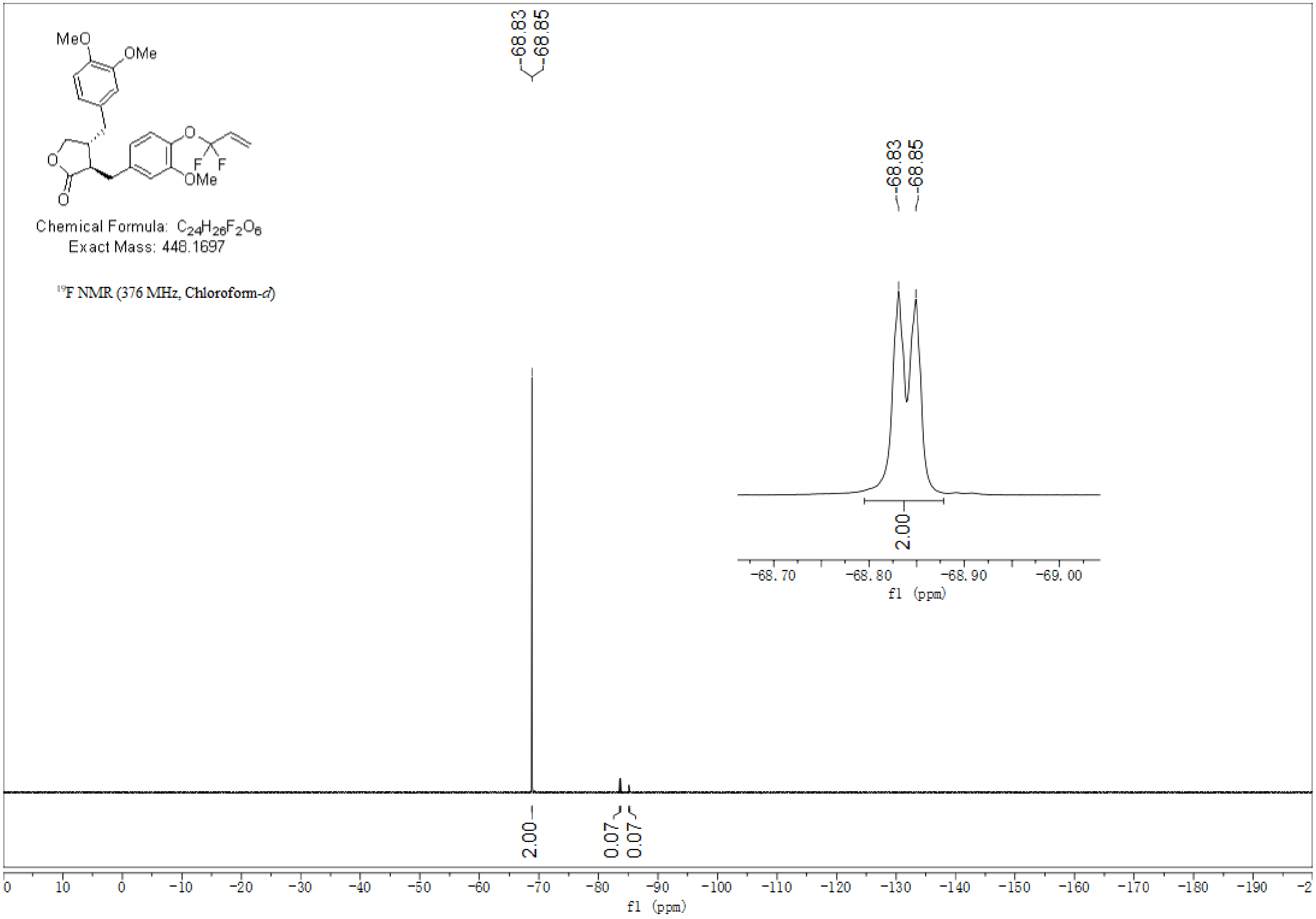

^{13}C NMR spectrum of **3i** (126 MHz, $CDCl_3$)

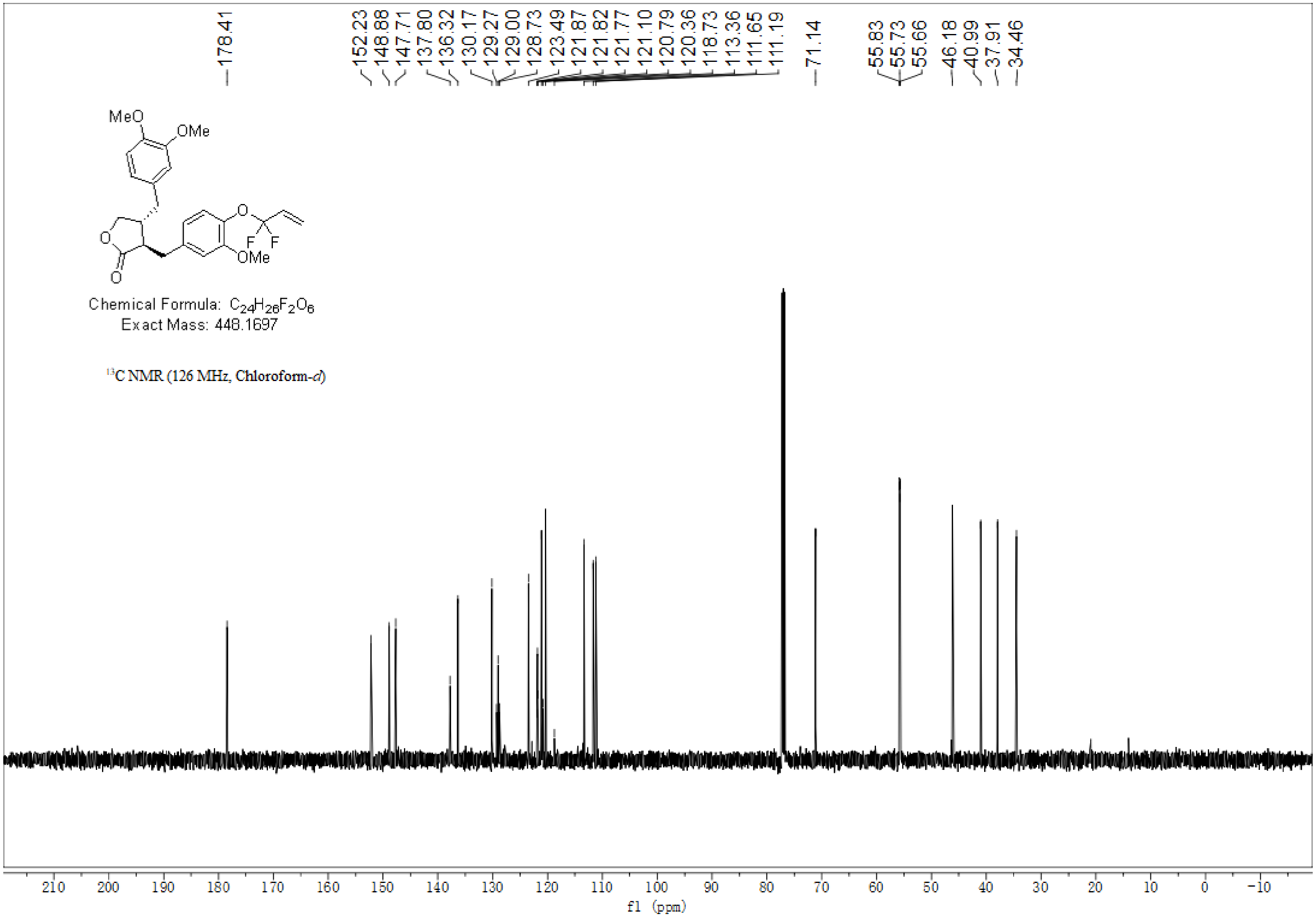

^{1}H NMR spectrum of **3j** (400 MHz, DMSO-d6)

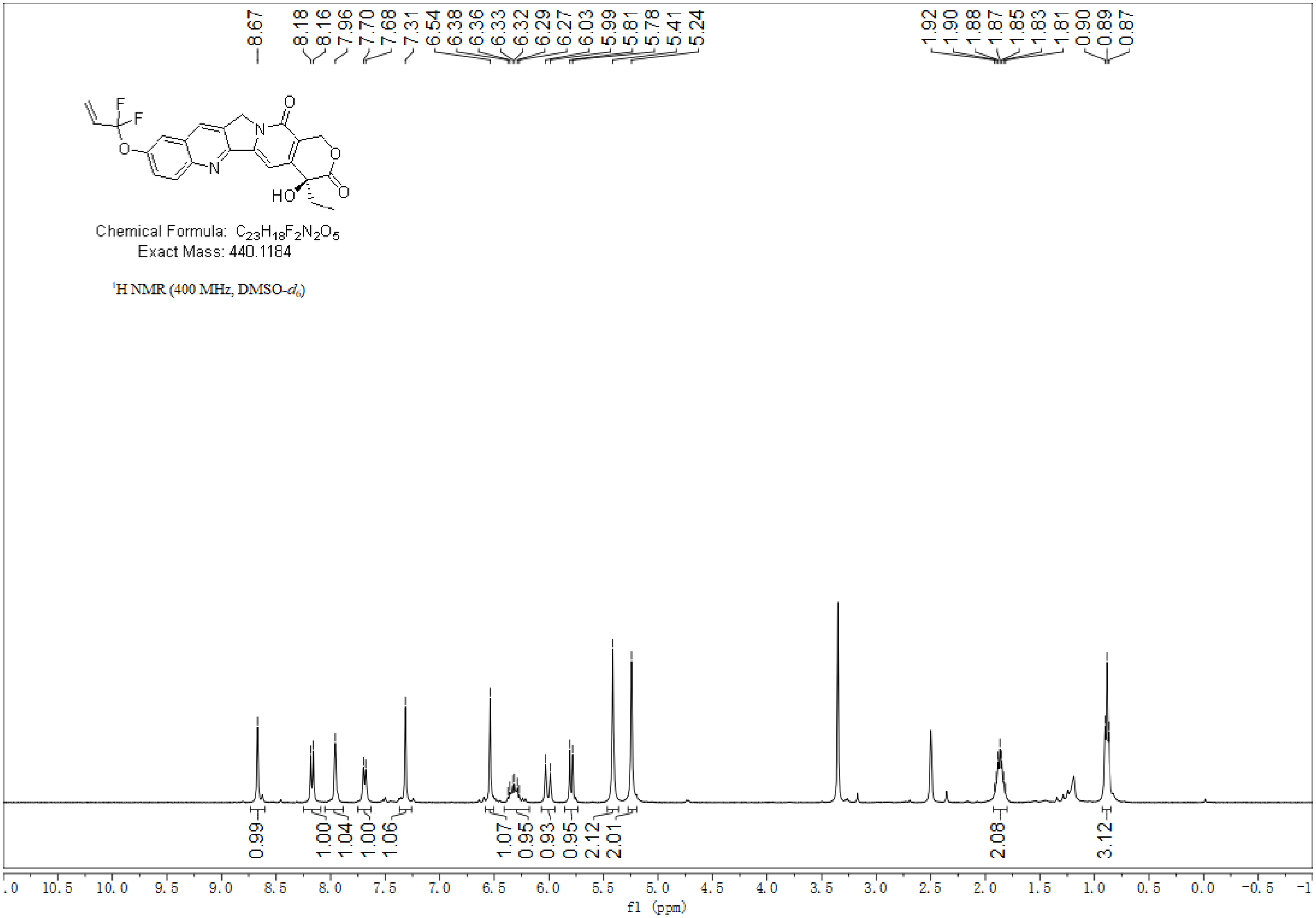

^{19}F NMR spectrum of **3j** (376 MHz, DMSO-d6)

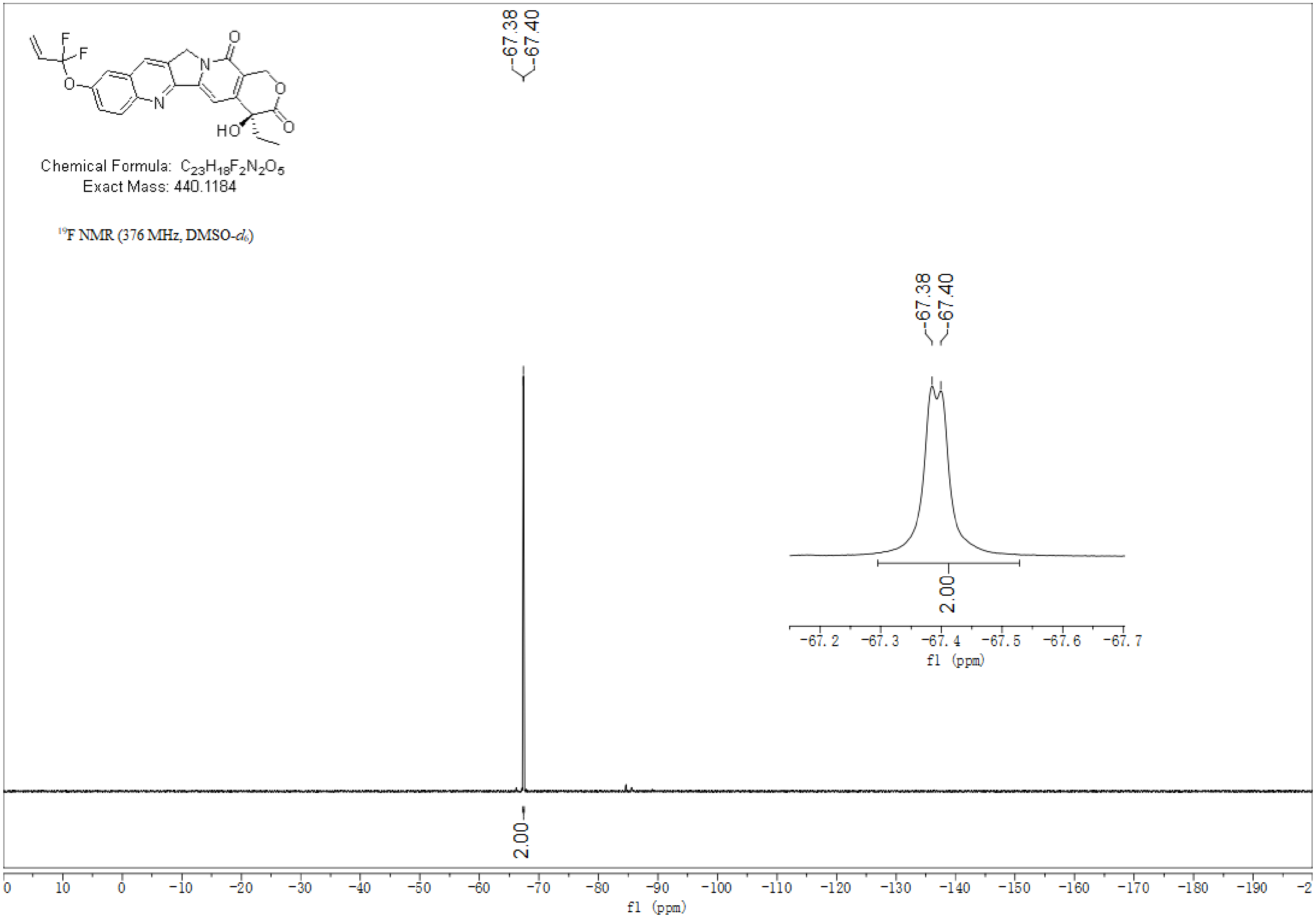

^{13}C NMR spectrum of **3j** (101 MHz, DMSO-d6)

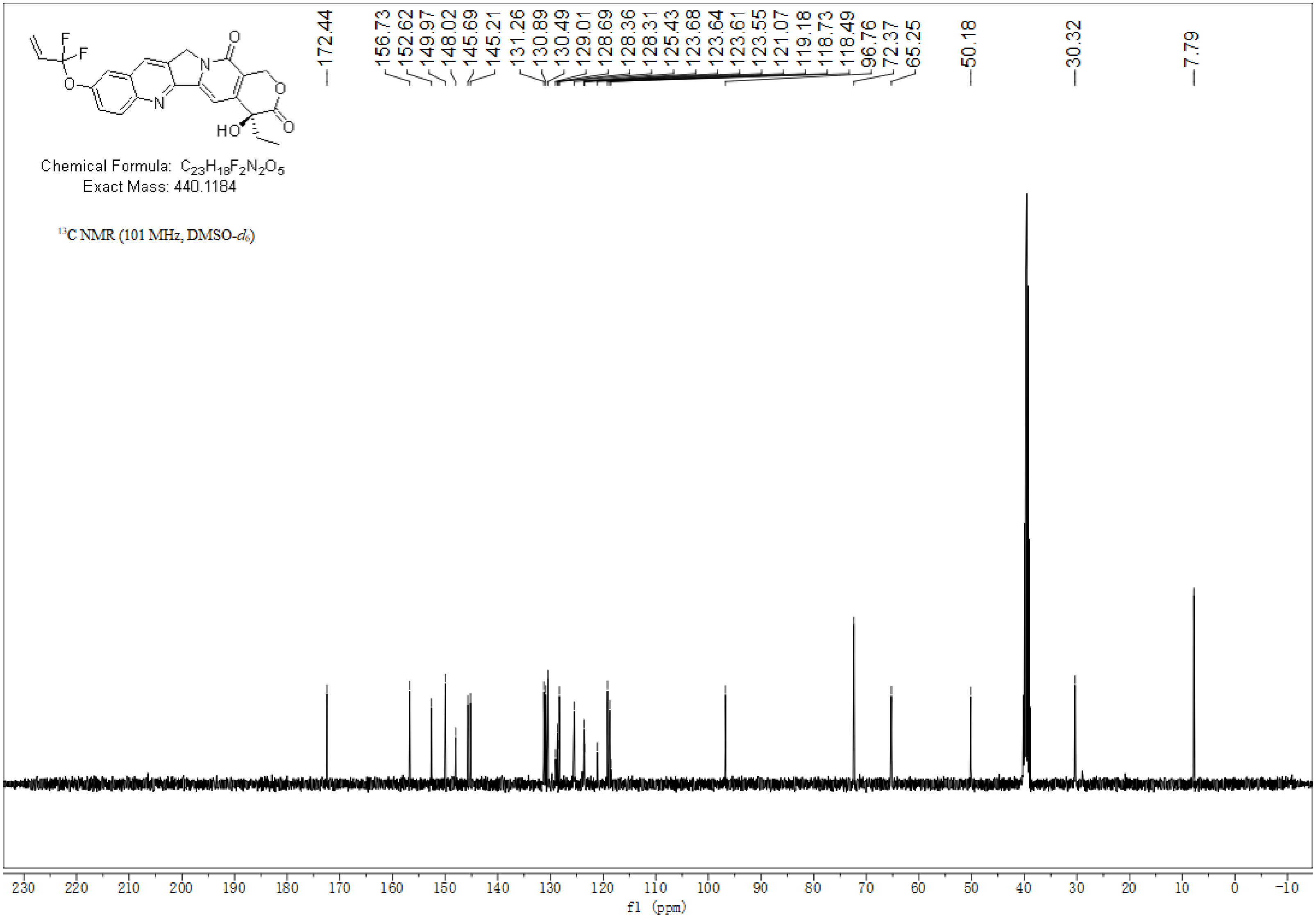

^{1}H NMR spectrum of **3k** (500 MHz, $CDCl_3$)

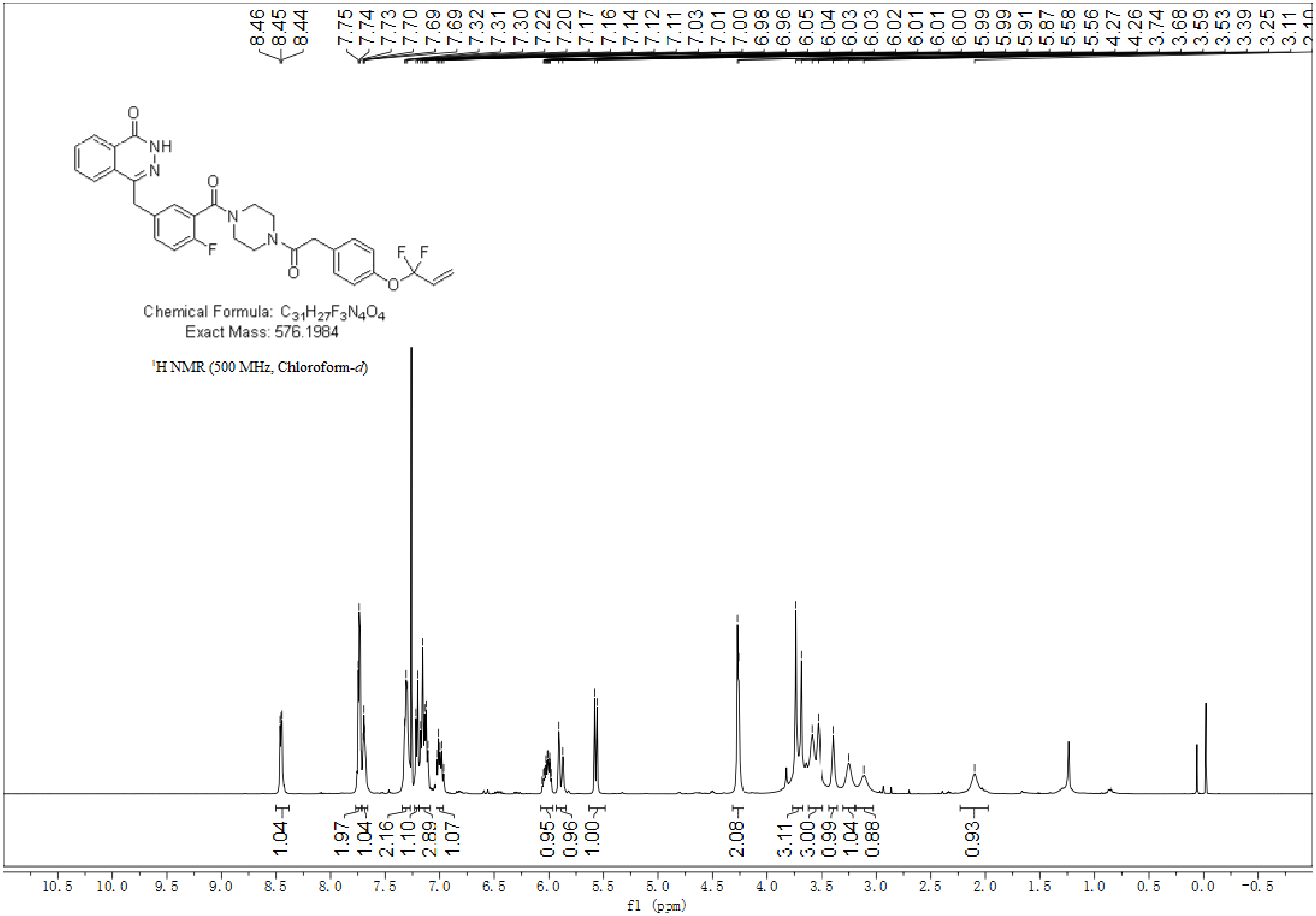

^{19}F NMR spectrum of **3k** (471 MHz, $CDCl_3$)

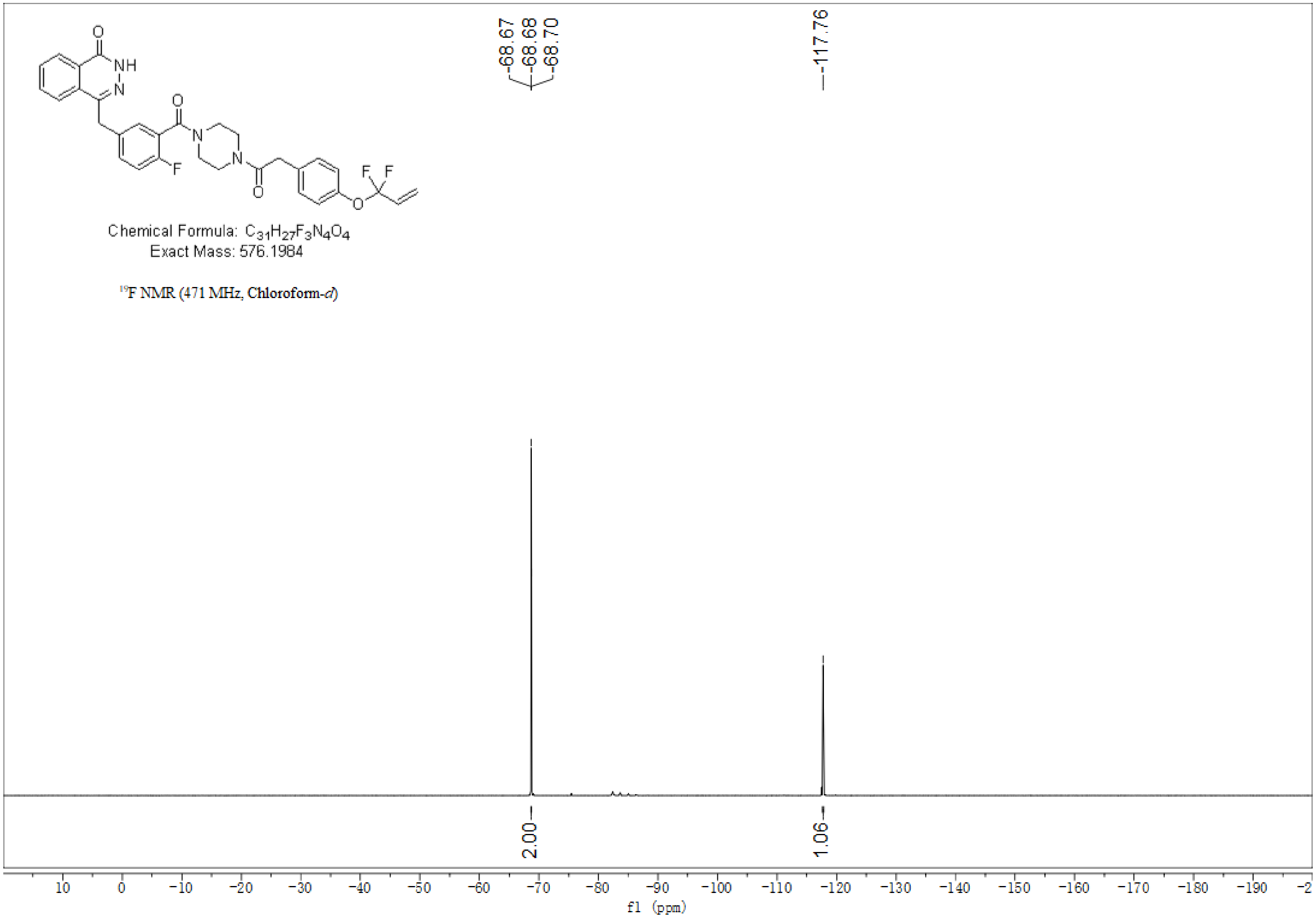

^{13}C NMR spectrum of **3k** (126 MHz, $CDCl_3$)

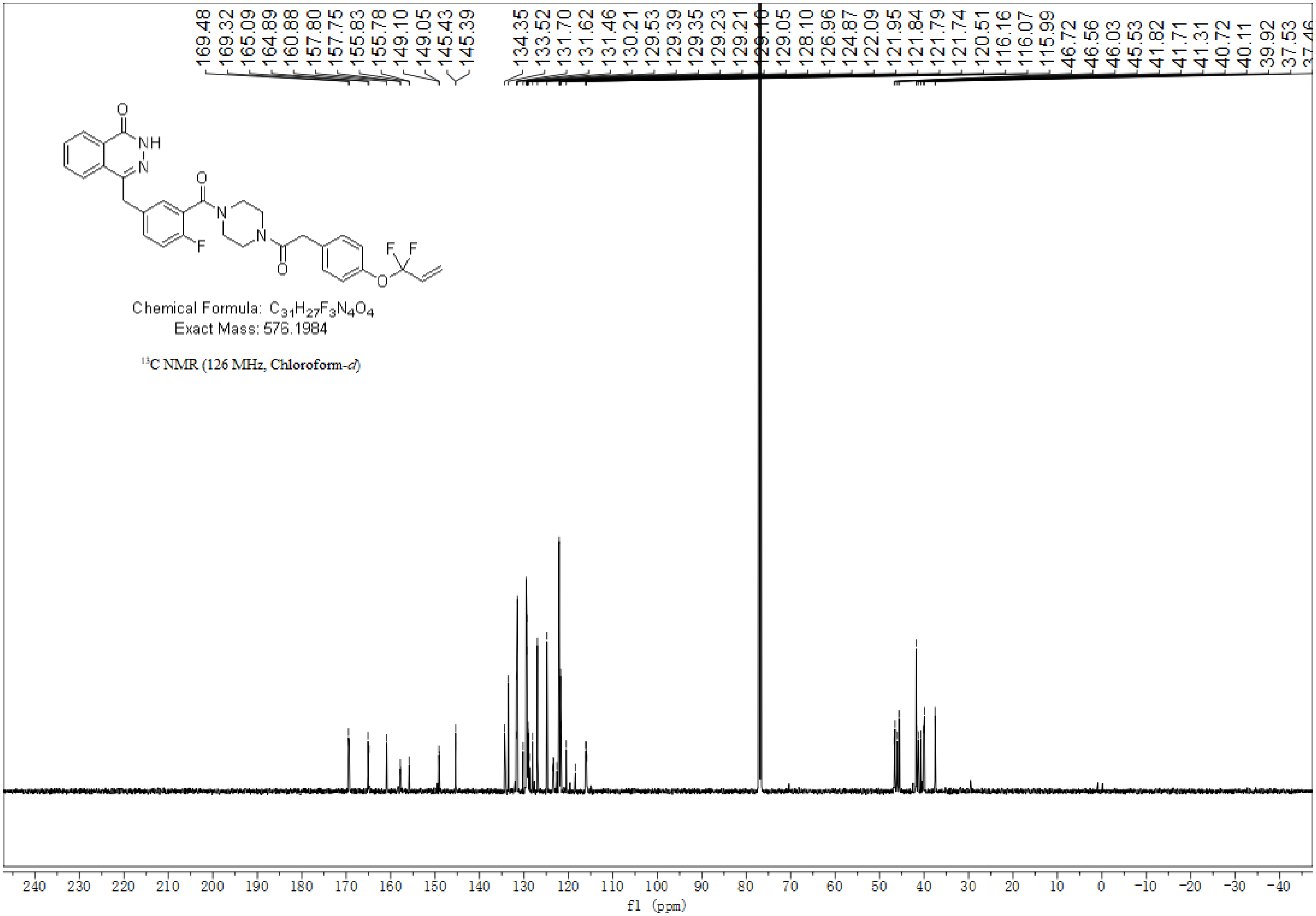

^{1}H NMR spectrum of **3l** (400 MHz, DMSO-d6)

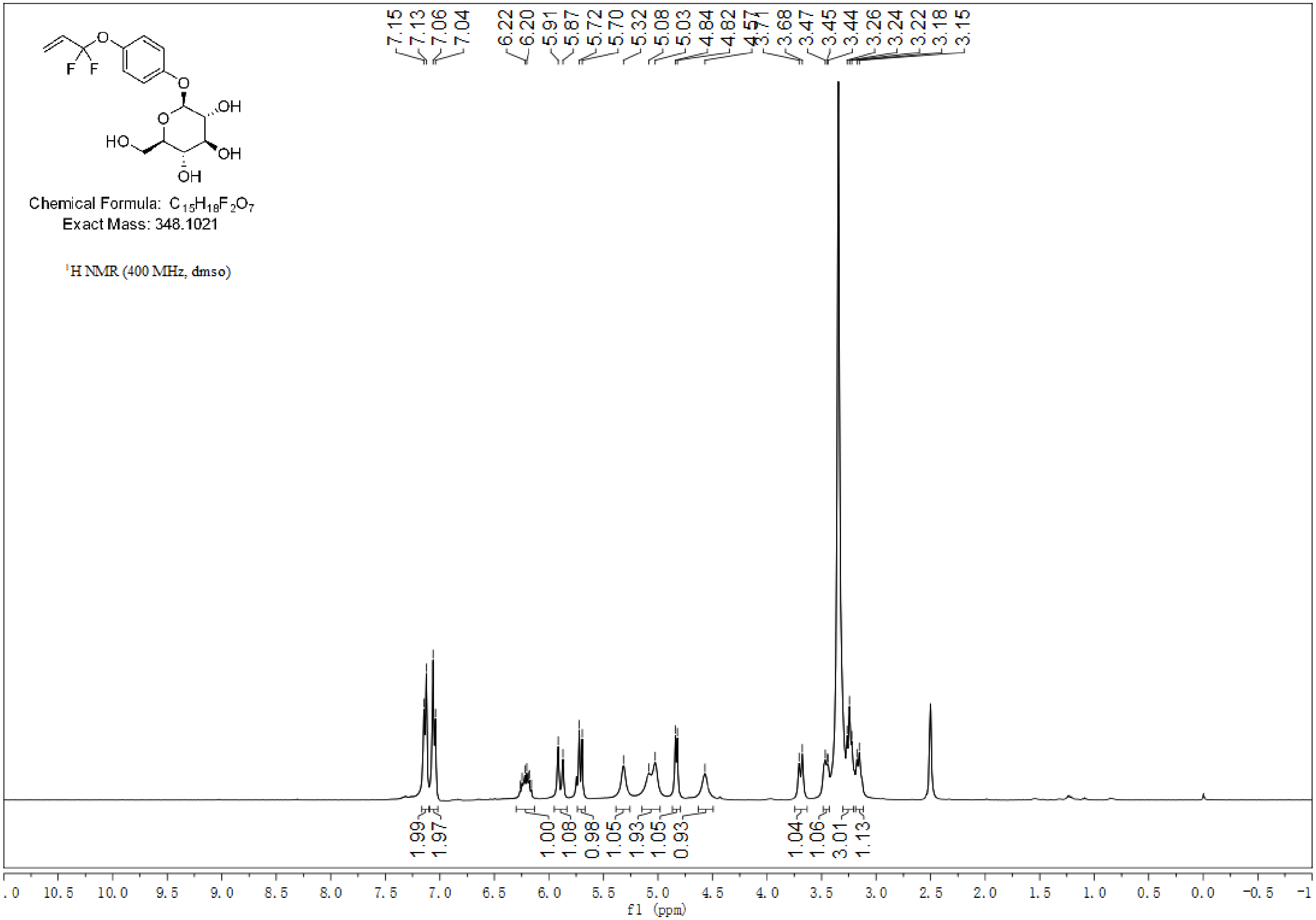

^{19}F NMR spectrum of **3l** (376 MHz, DMSO-d6)

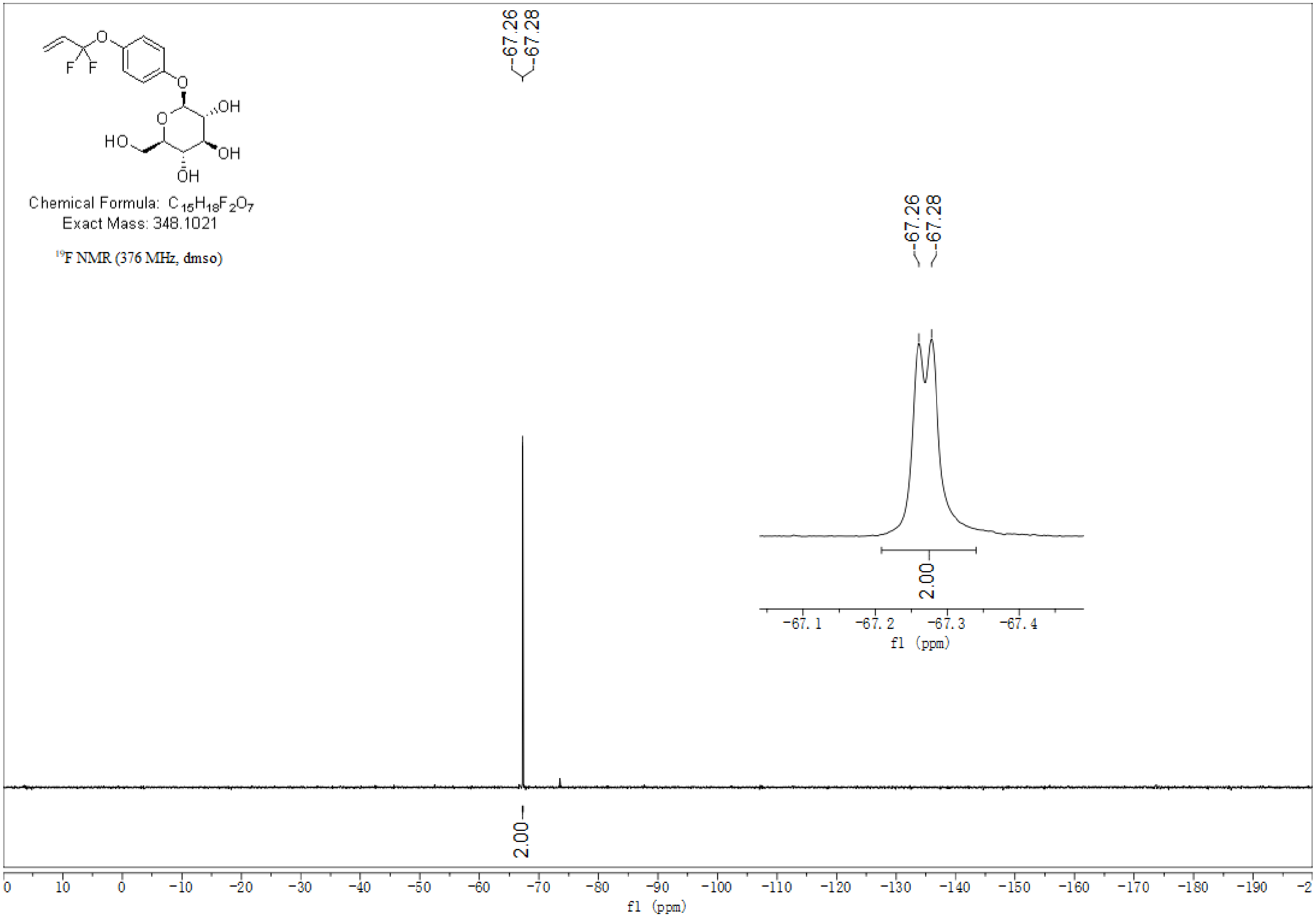

^{13}C NMR spectrum of **3l** (126 MHz, DMSO-d6)

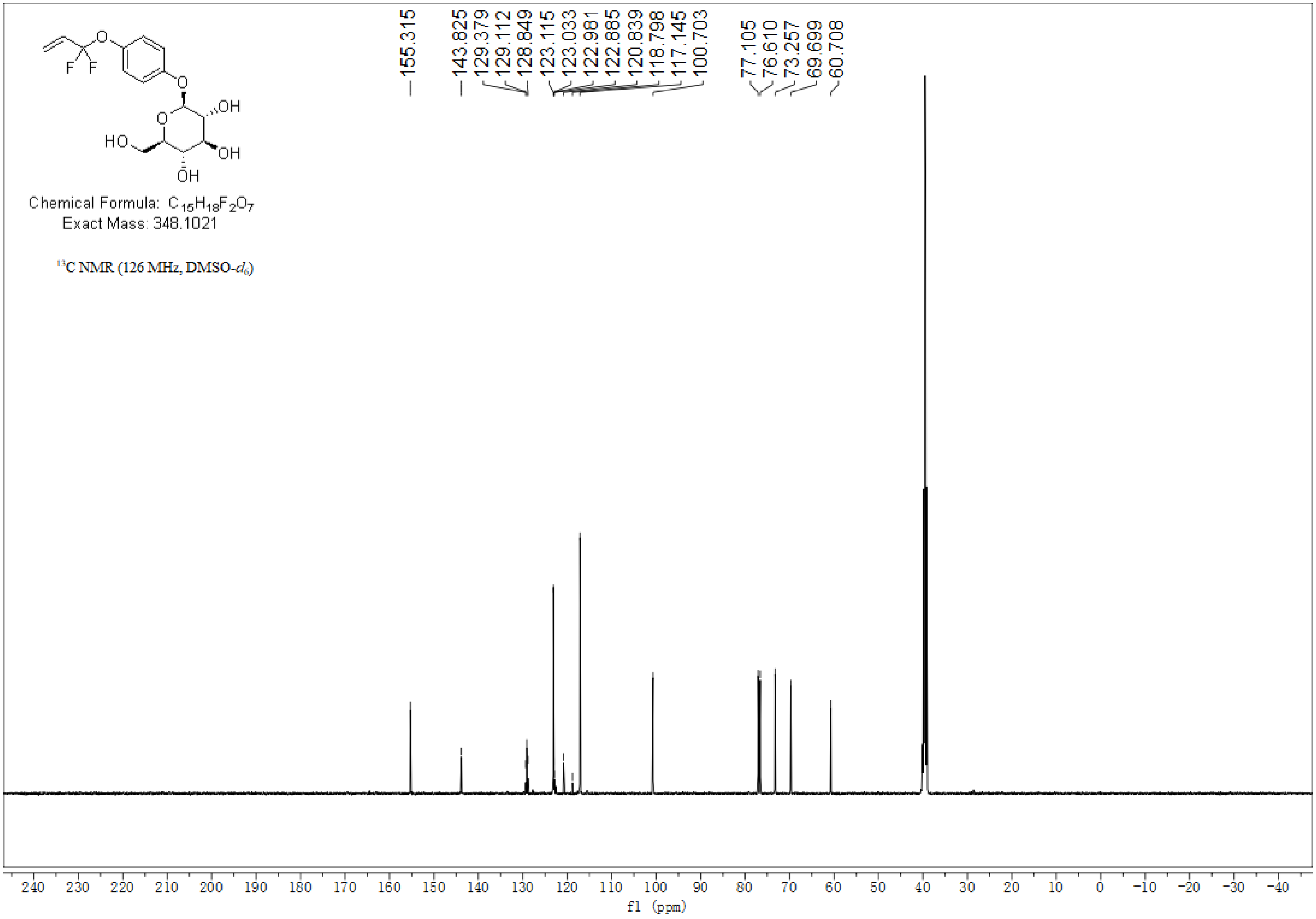

^{1}H NMR spectrum of **3l′** (400 MHz, $CDCl_3$)

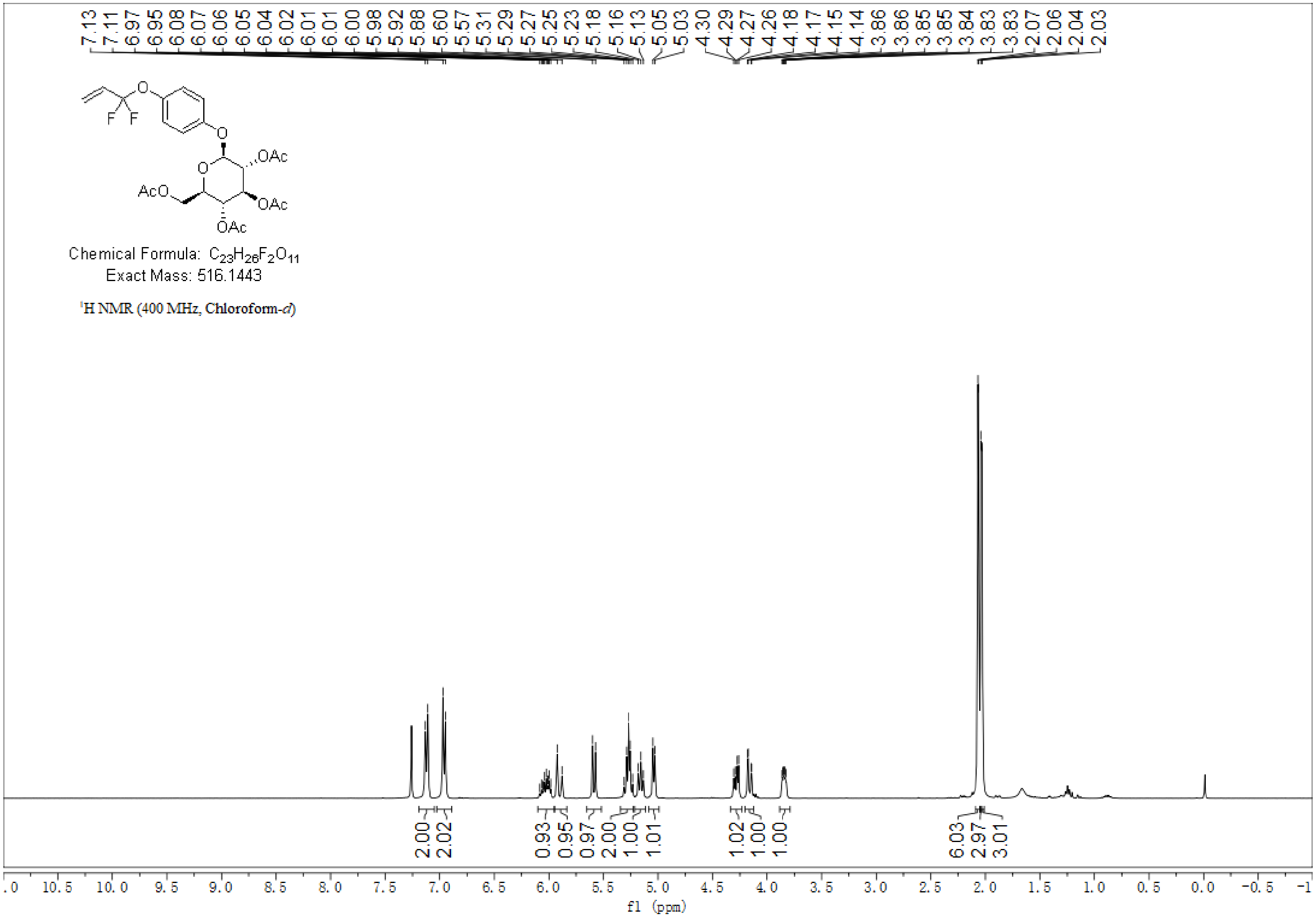

^{19}F NMR spectrum of **3l′** (376 MHz, $CDCl_3$)

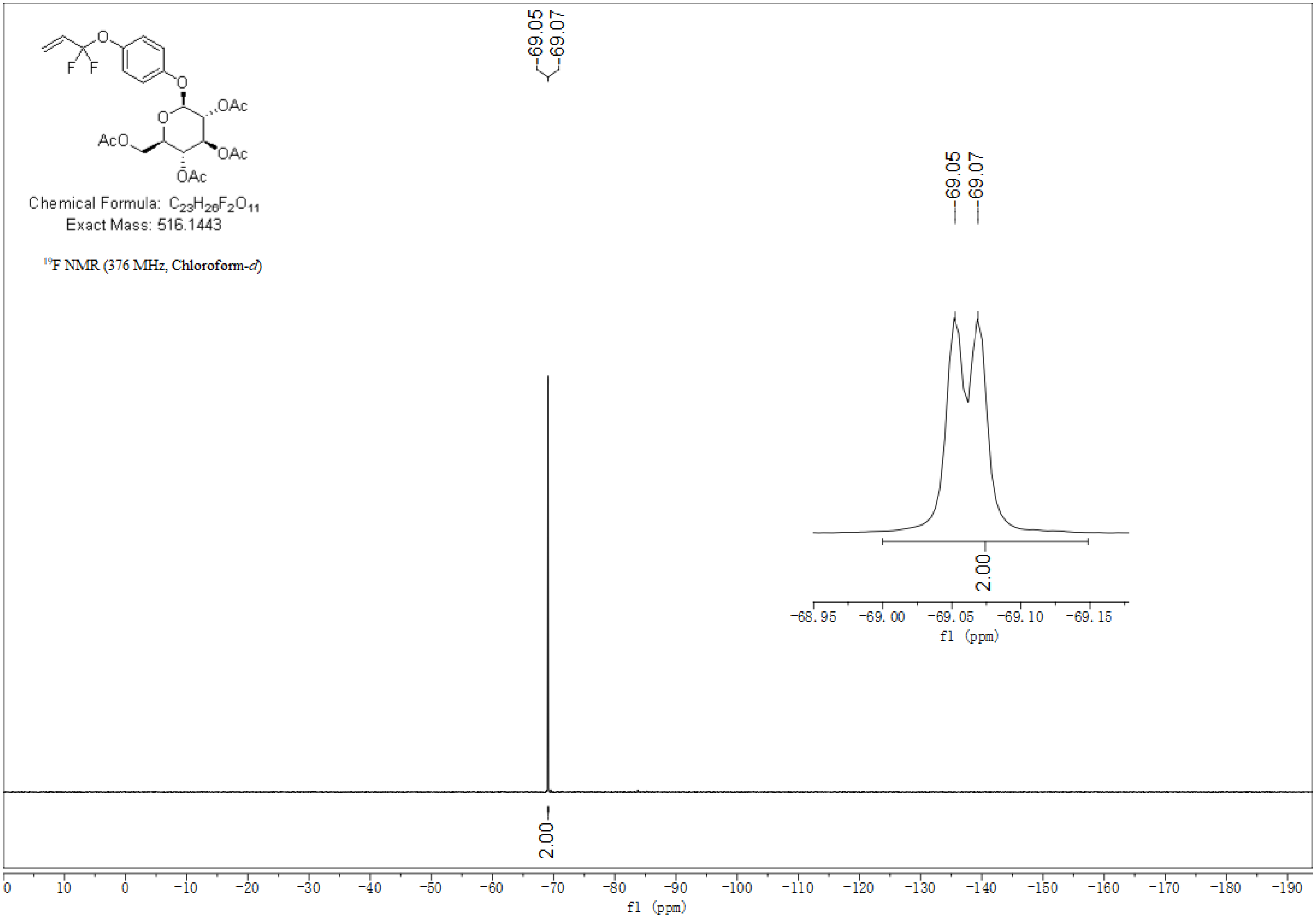

^{13}C NMR spectrum of **3l′** (101 MHz, $CDCl_3$)

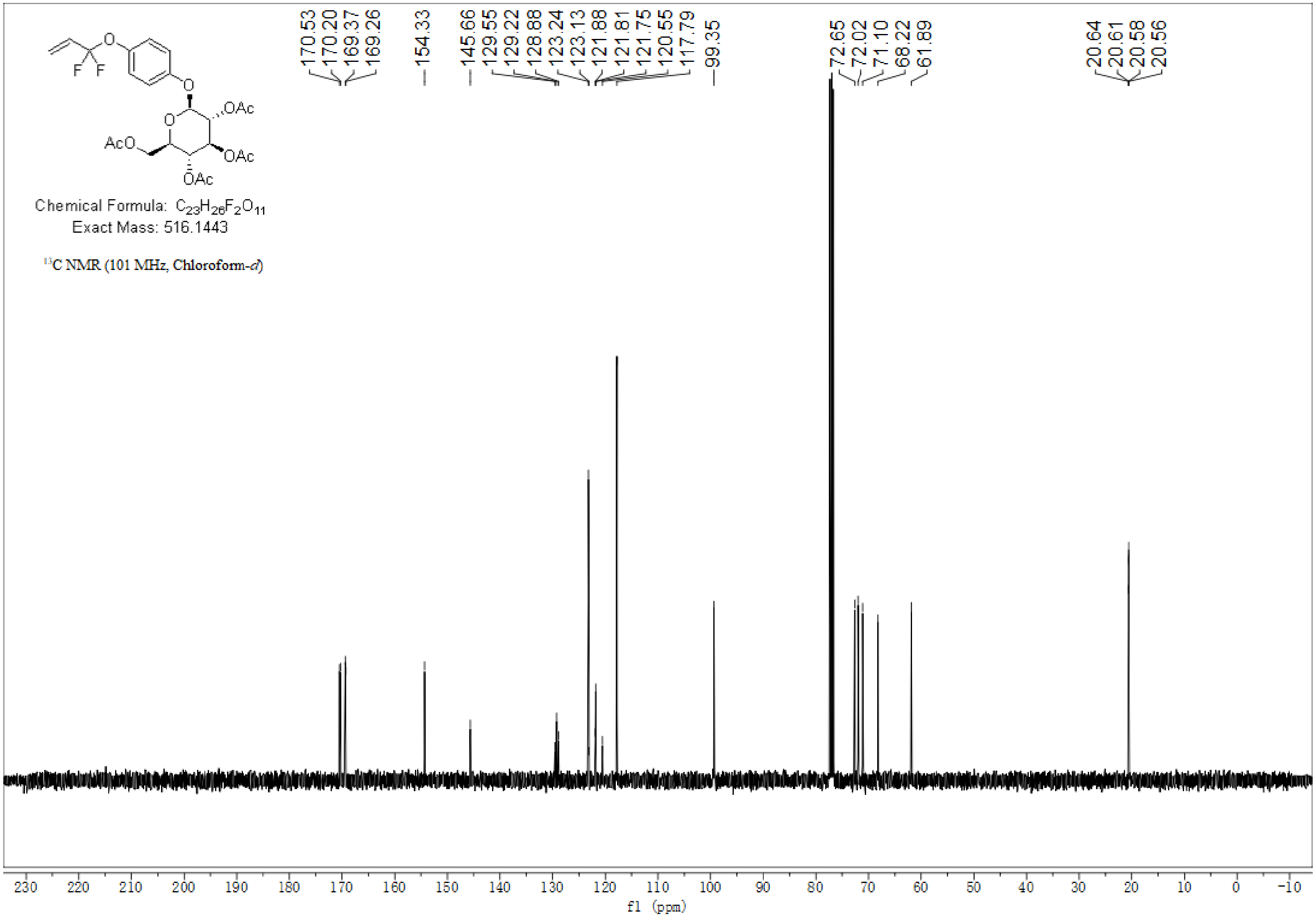

^{1}H NMR spectrum of **3m** (400 MHz, CD_3OD)

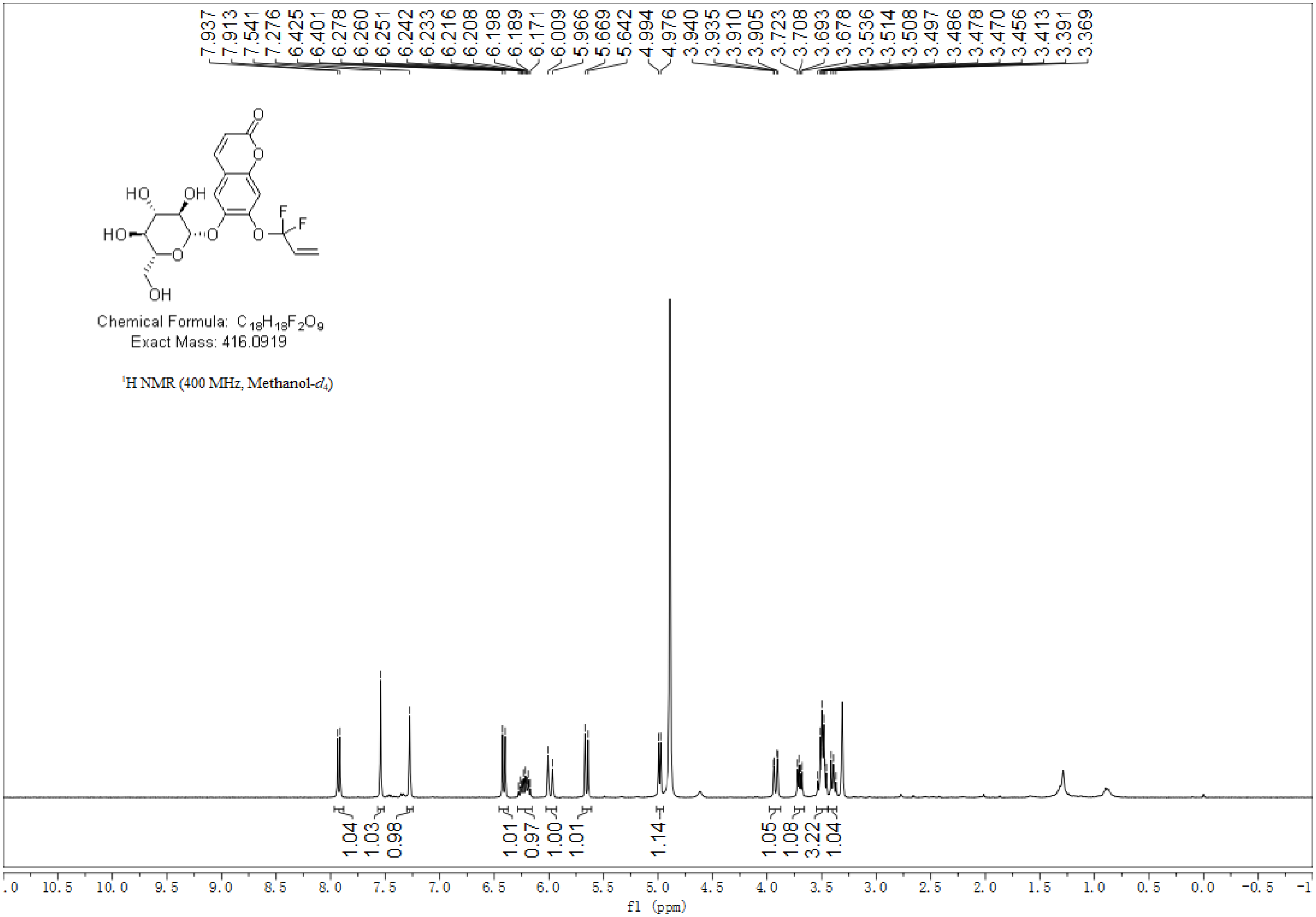

^{19}F NMR spectrum of **3m** (376 MHz, CD_3OD)

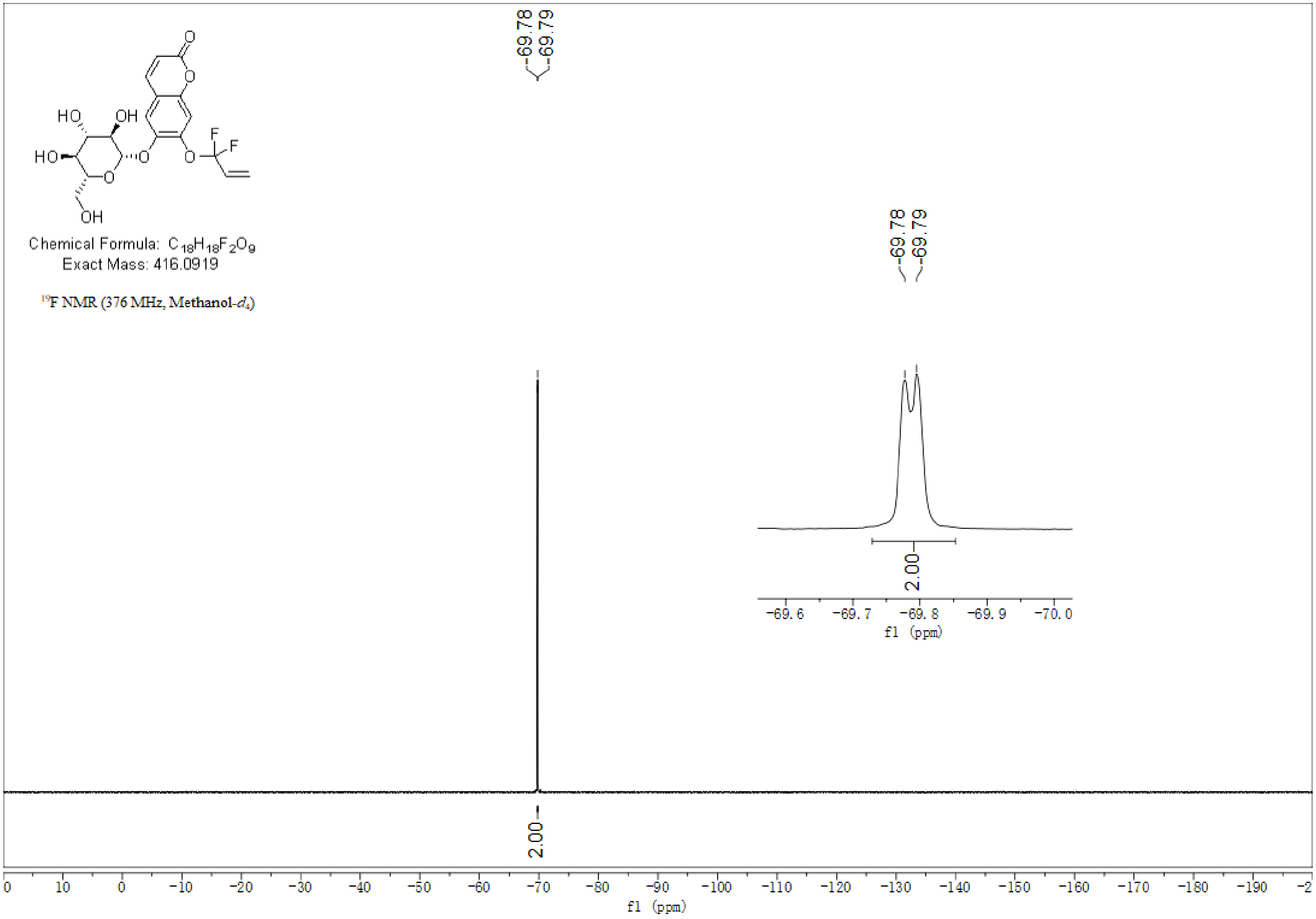

^{13}C NMR spectrum of **3m** (126 MHz, DMSO-d6)

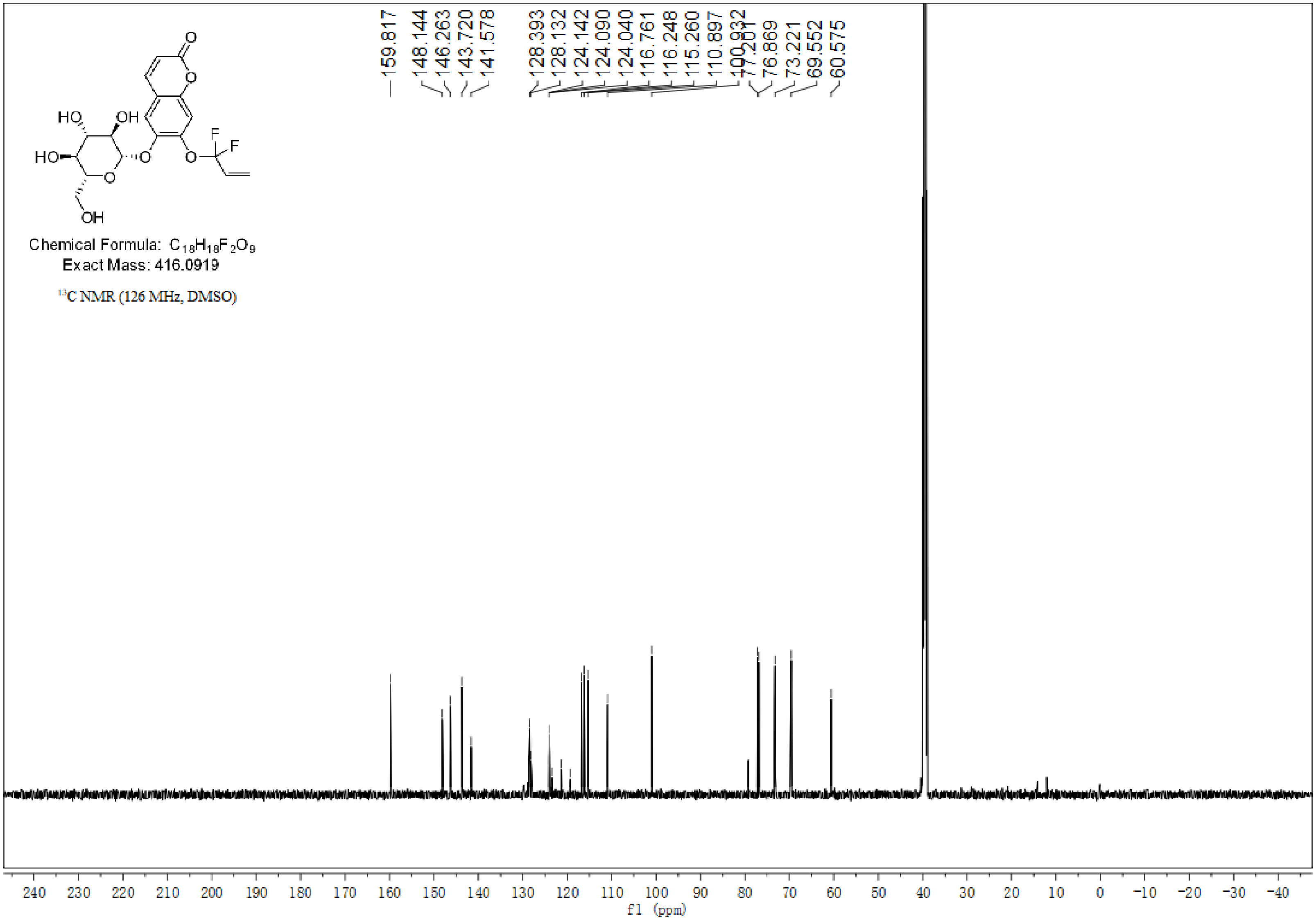

^{1}H NMR spectrum of **3m′** (400 MHz, $CDCl_3$)

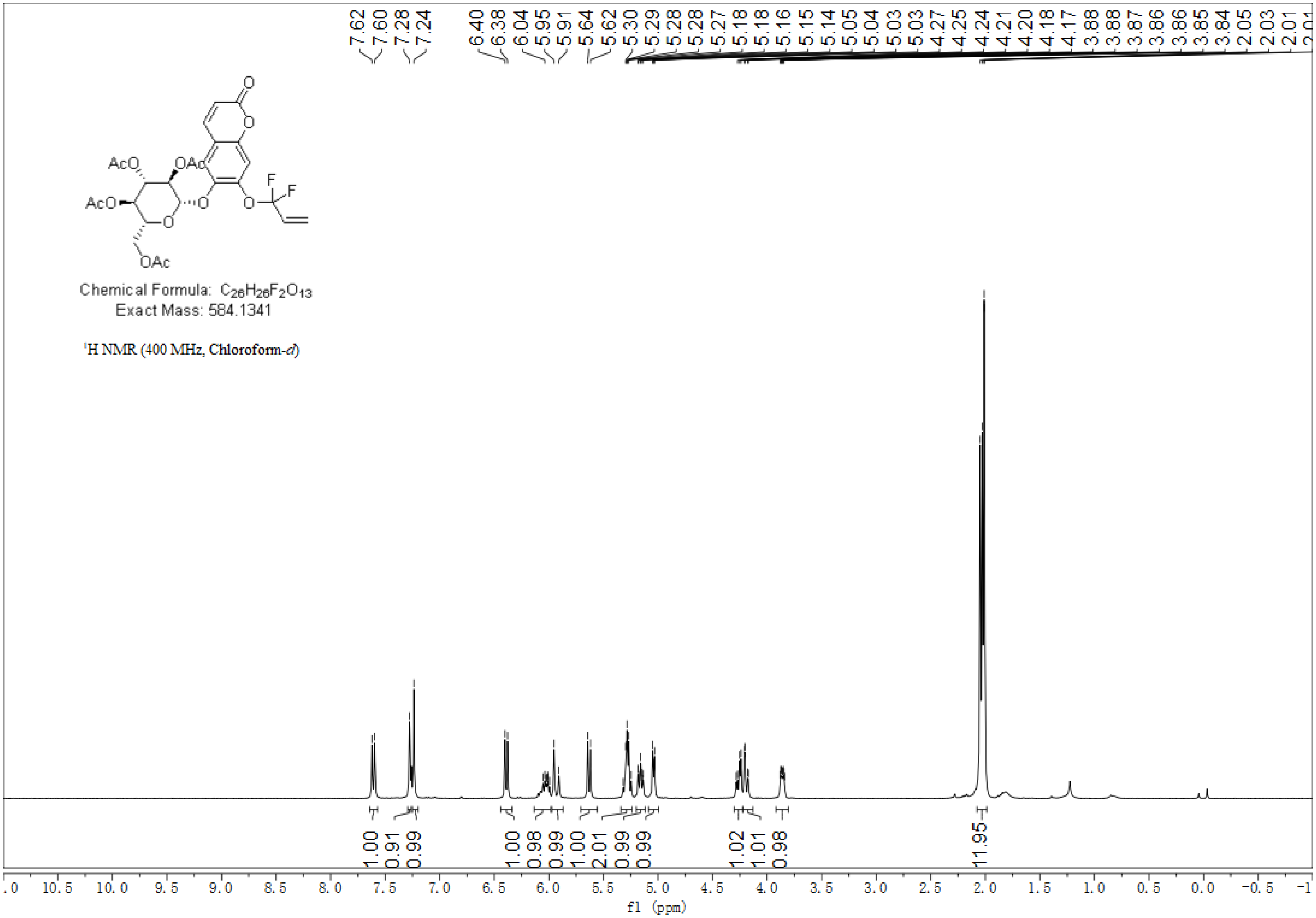

^{19}F NMR spectrum of **3m′** (376 MHz, $CDCl_3$)

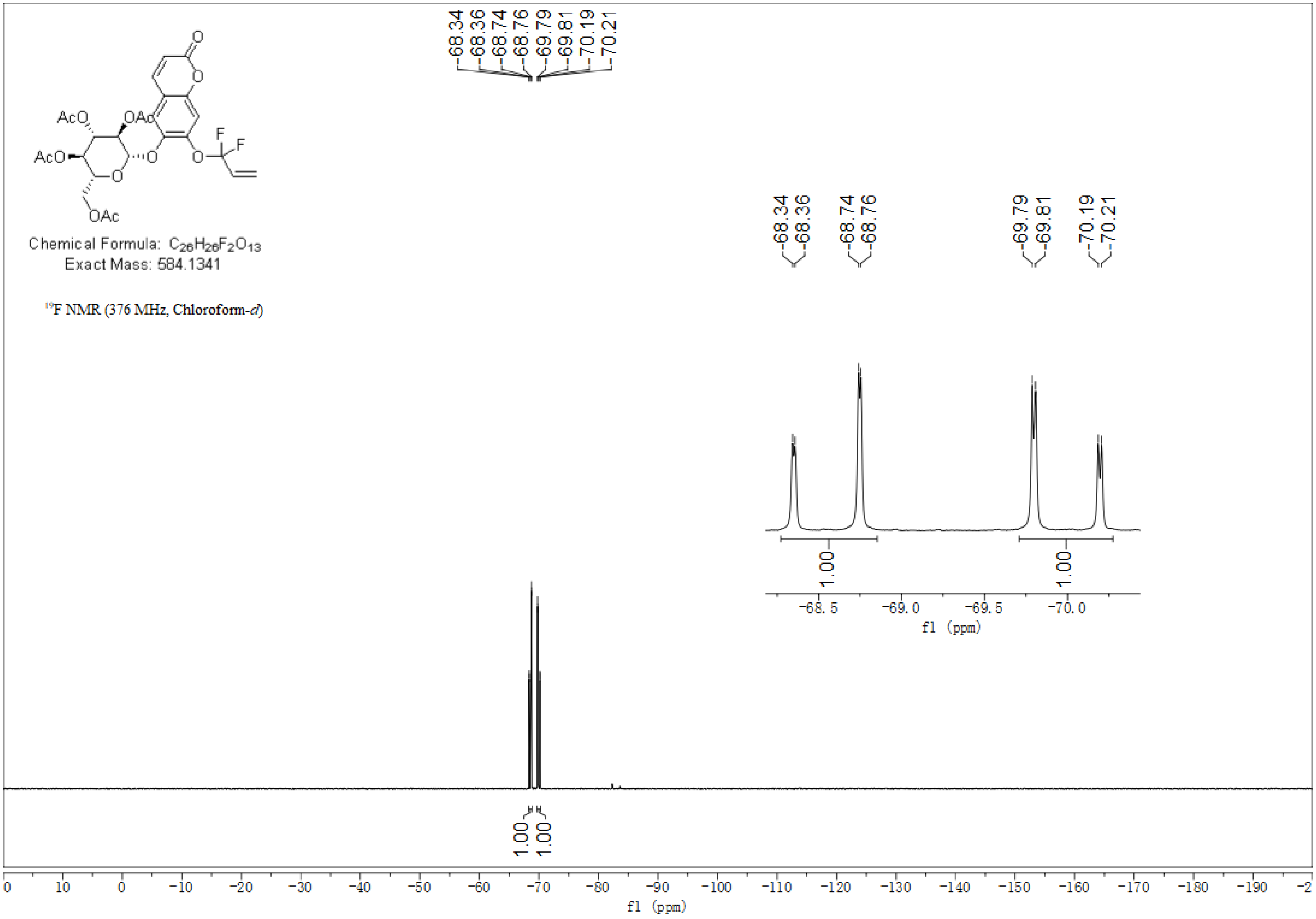

^{13}C NMR spectrum of **3m′** (126 MHz, $CDCl_3$)

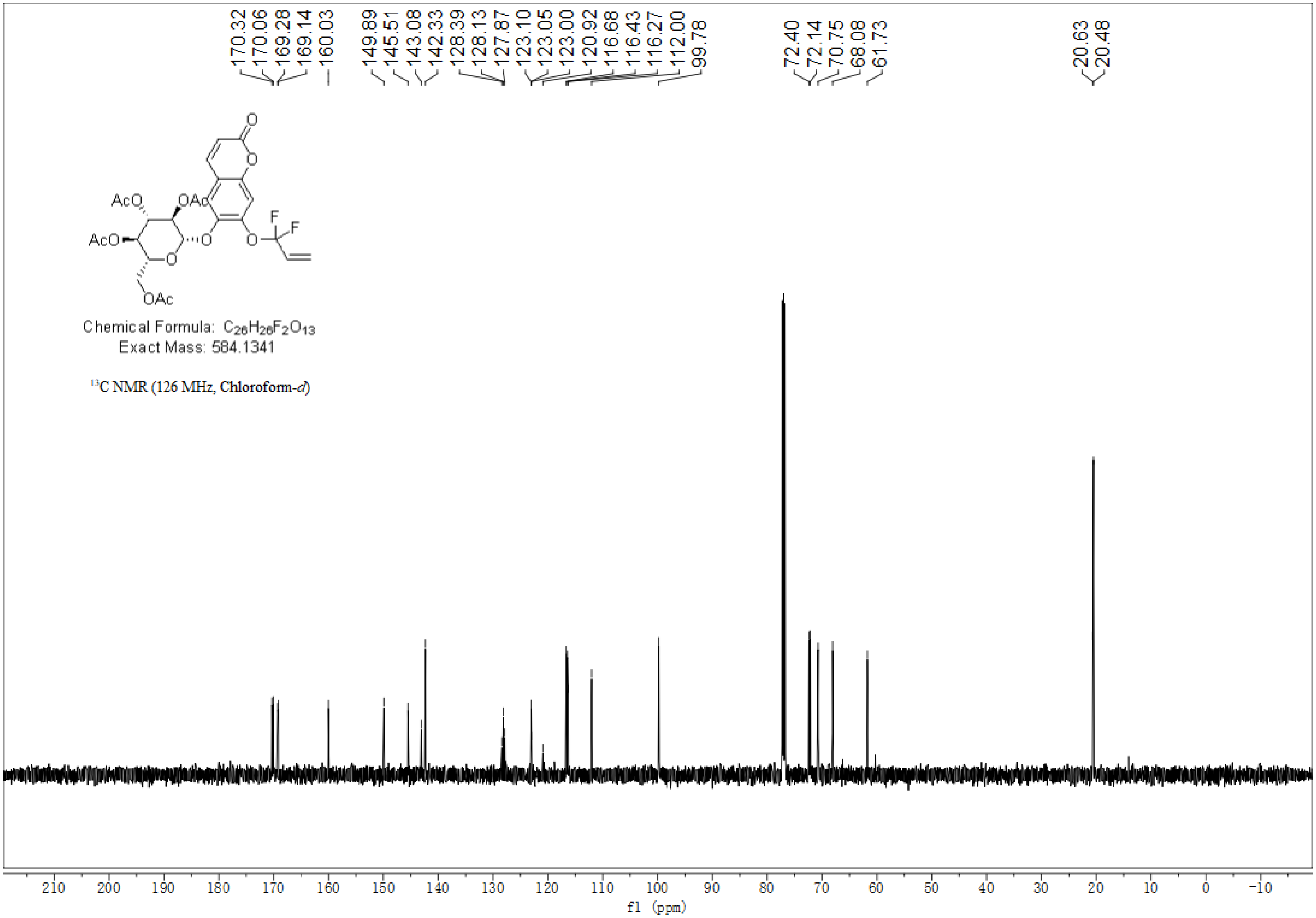

^{1}H NMR spectrum of **3n** (400 MHz, $CDCl_3$)

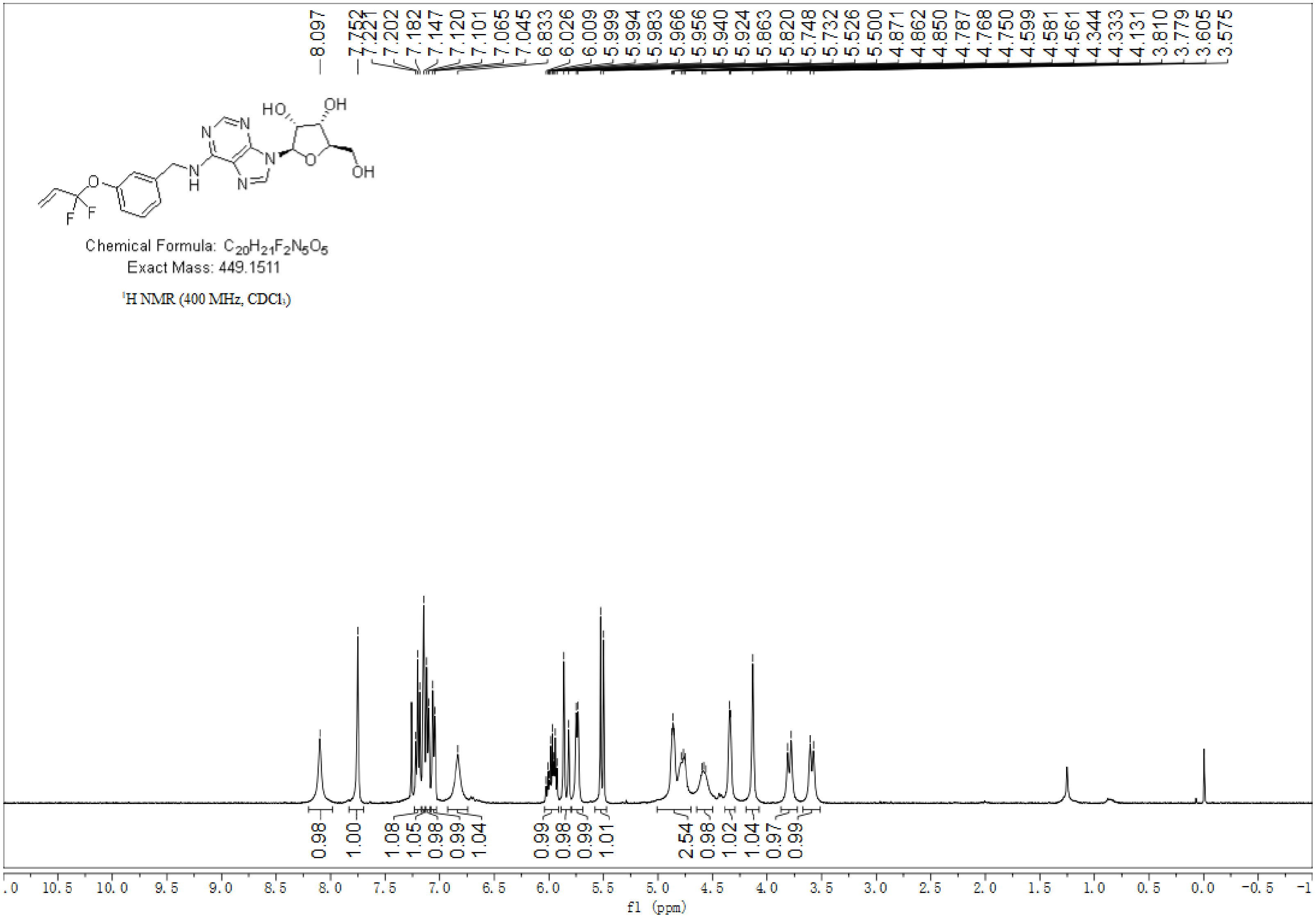

^{19}F NMR spectrum of **3n** (376 MHz, $CDCl_3$)

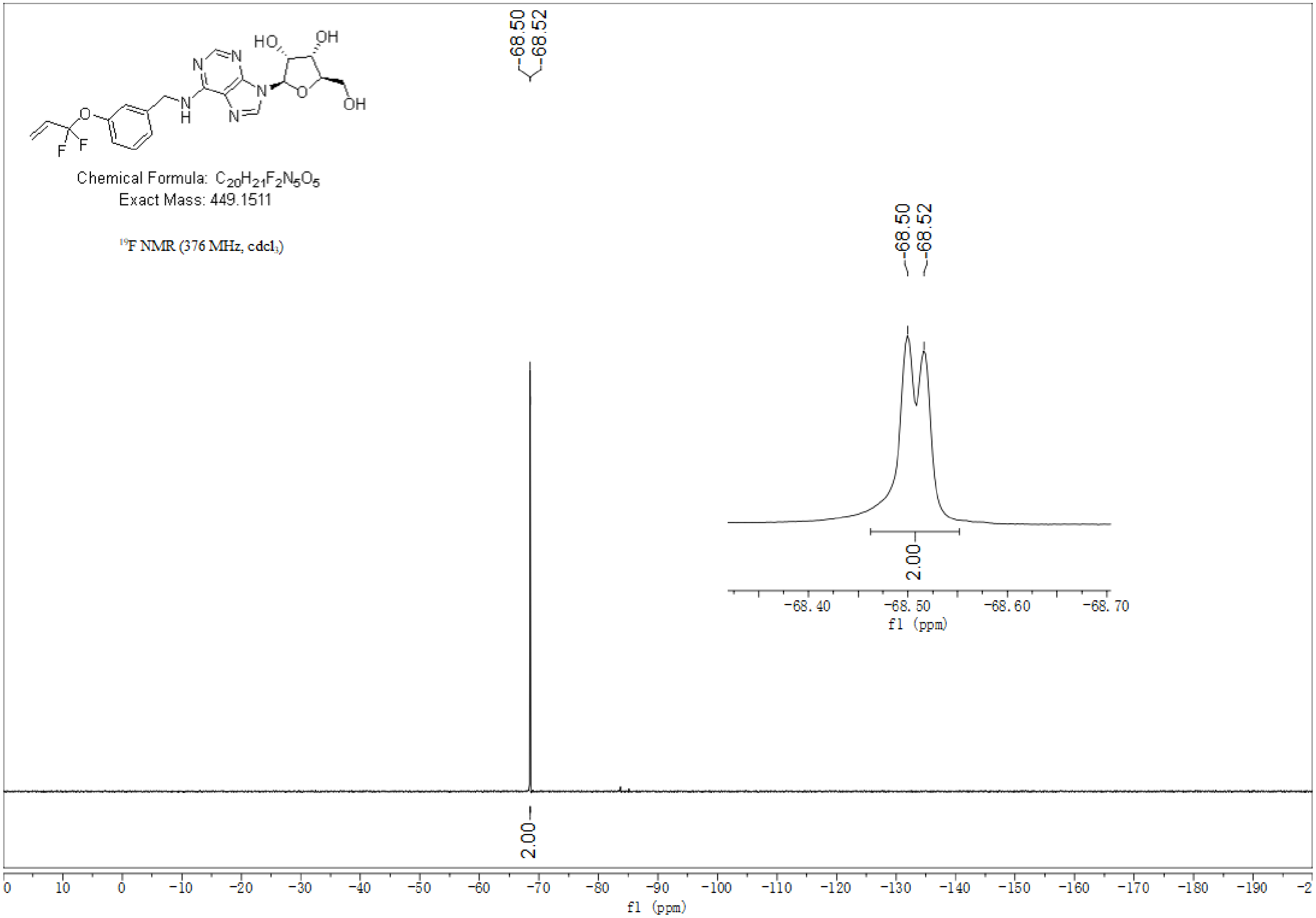

^{13}C NMR spectrum of **3n** (126 MHz, $CDCl_3$)

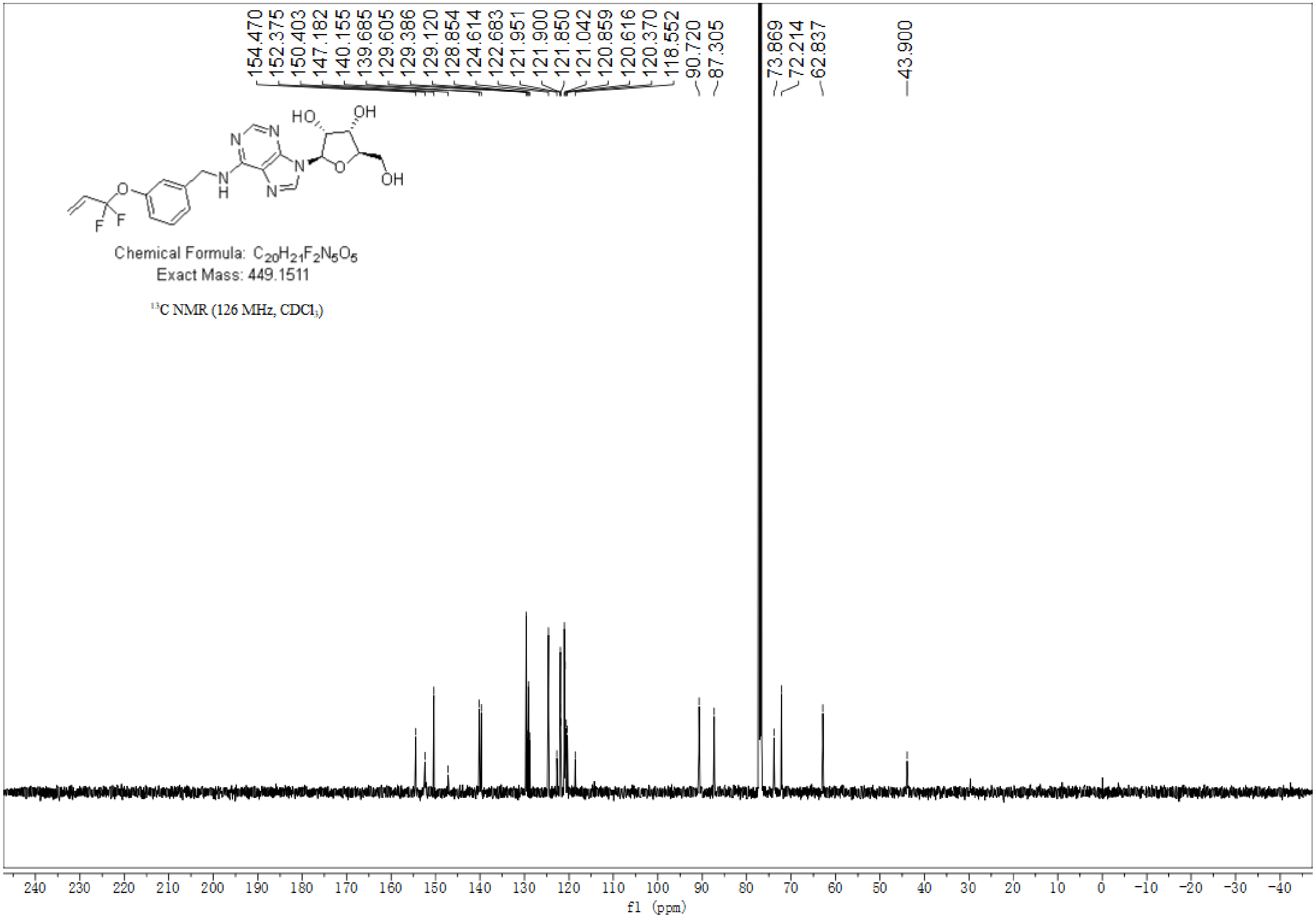

^{1}H NMR spectrum of **3n′** (400 MHz, $CDCl_3$)

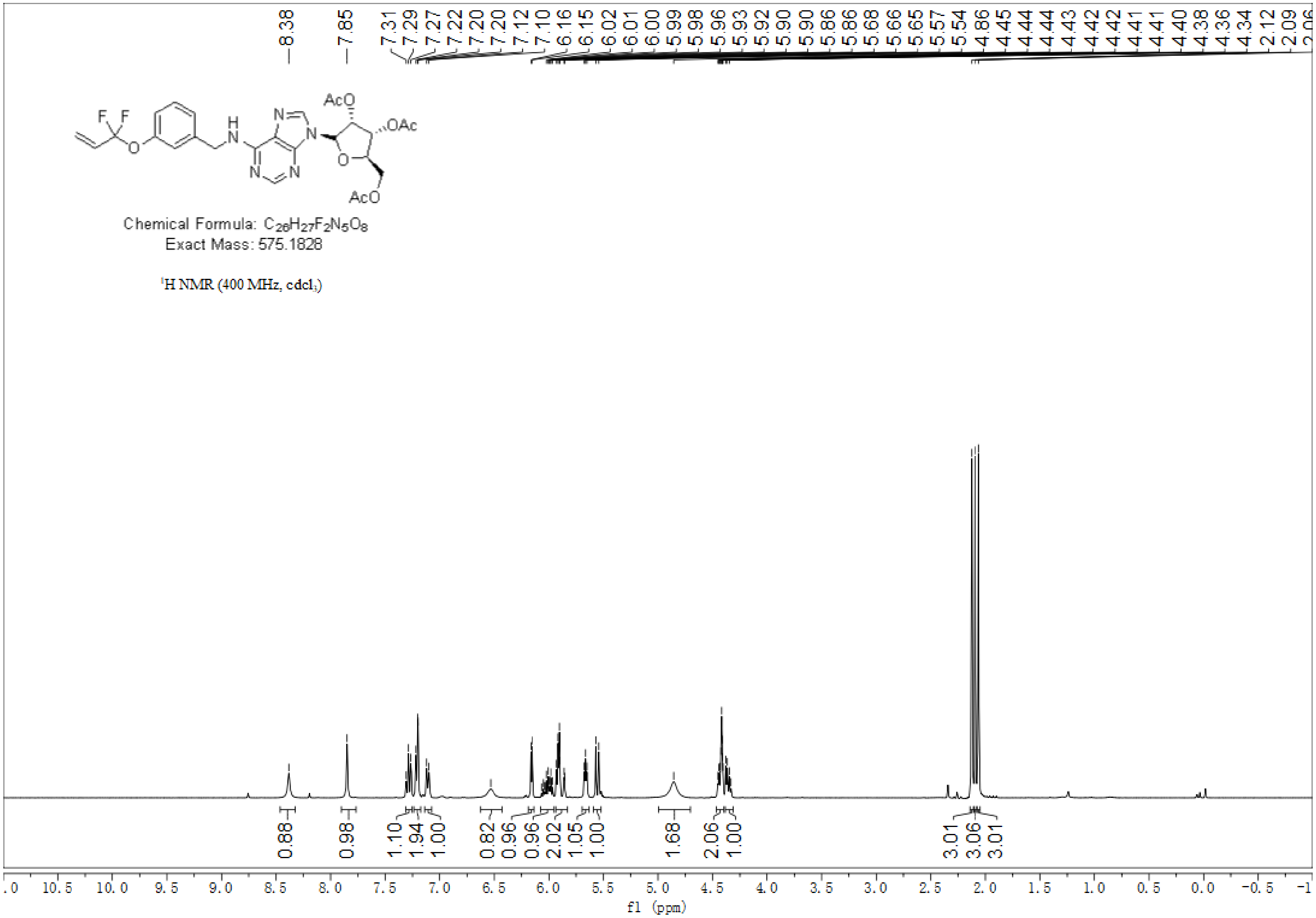

^{19}F NMR spectrum of **3n′** (376 MHz, $CDCl_3$)

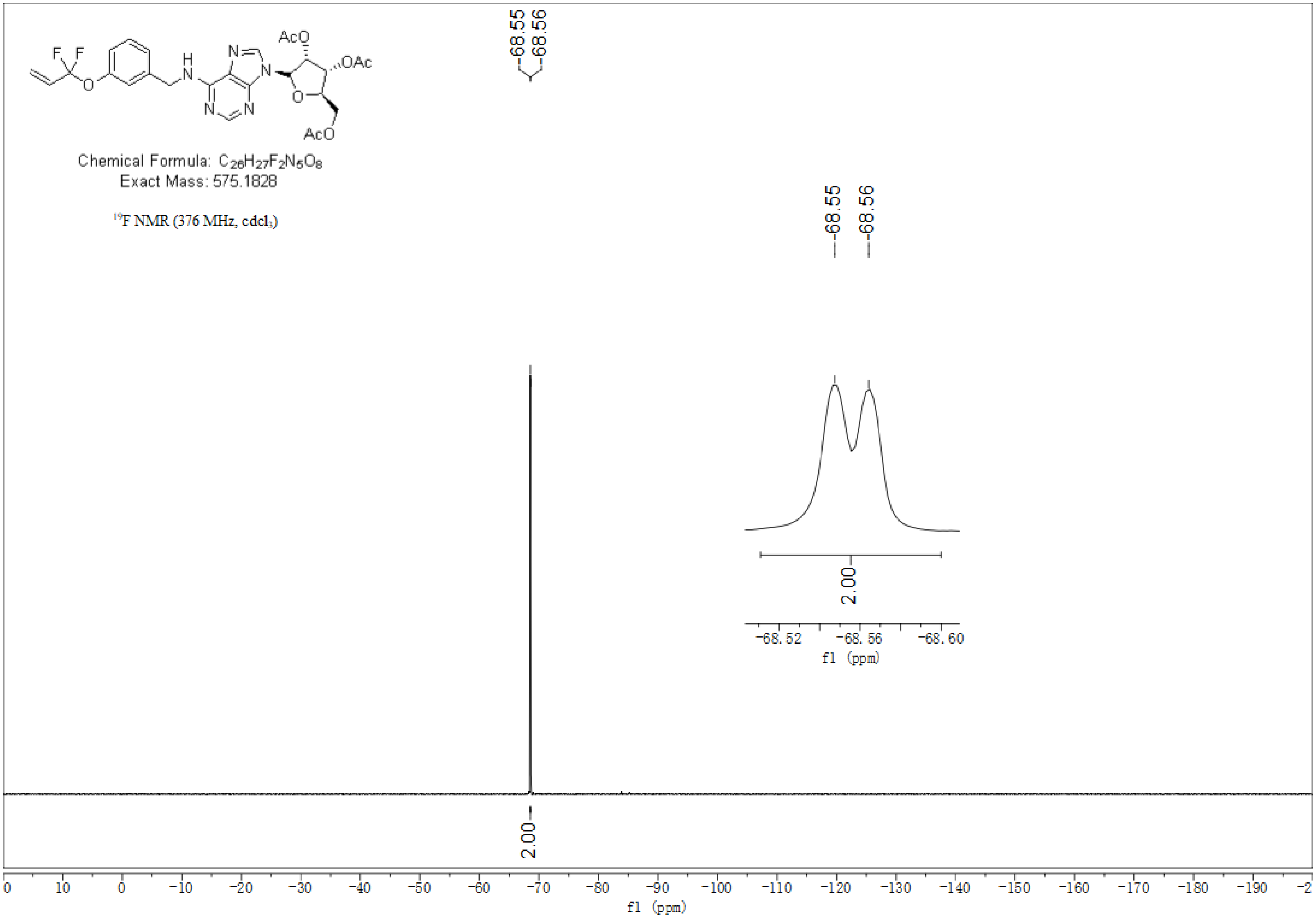

^{13}C NMR spectrum of **3n′** (151 MHz, $CDCl_3$)

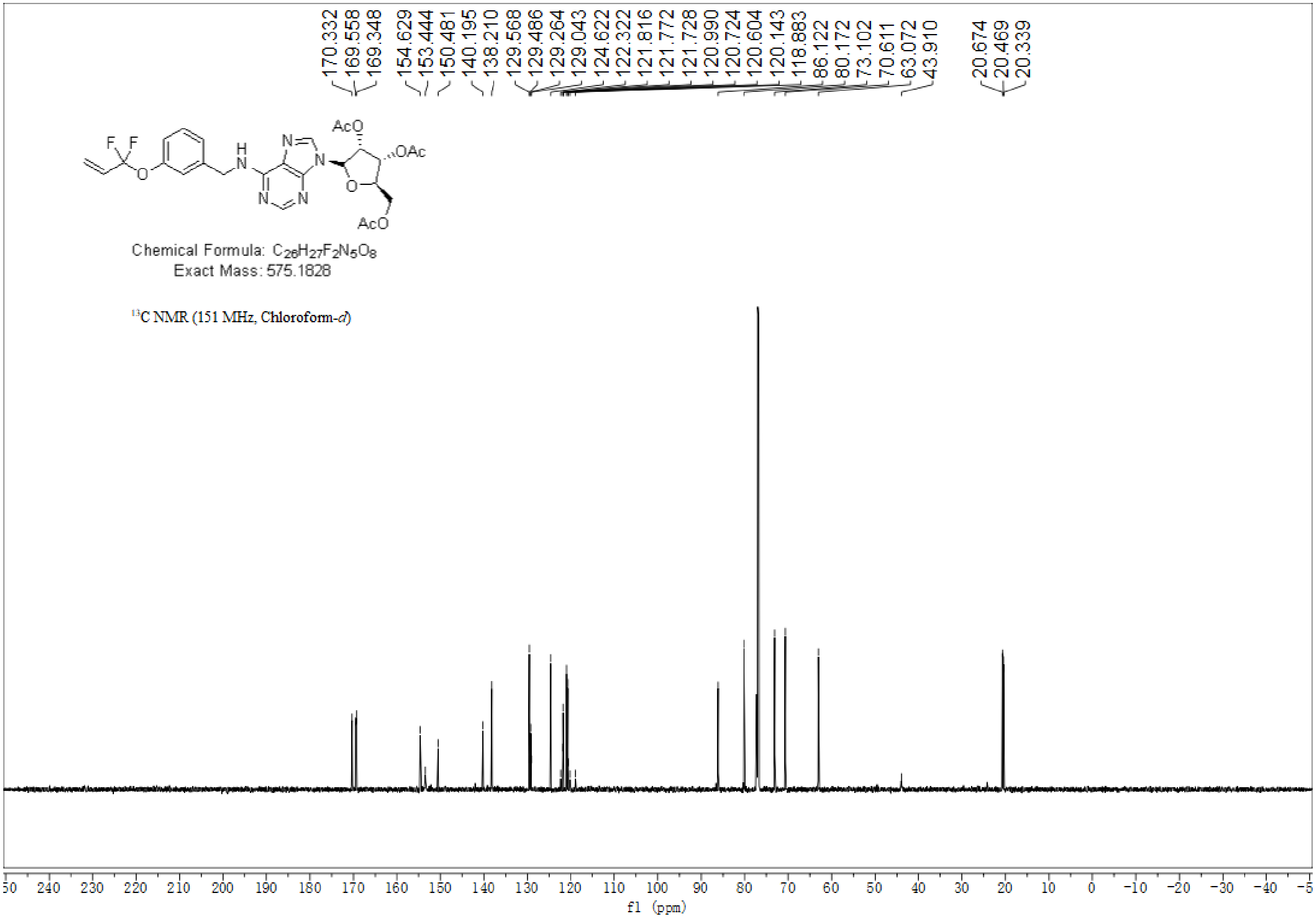

^{1}H NMR spectrum of **3o** (600 MHz, DMSO-d6)

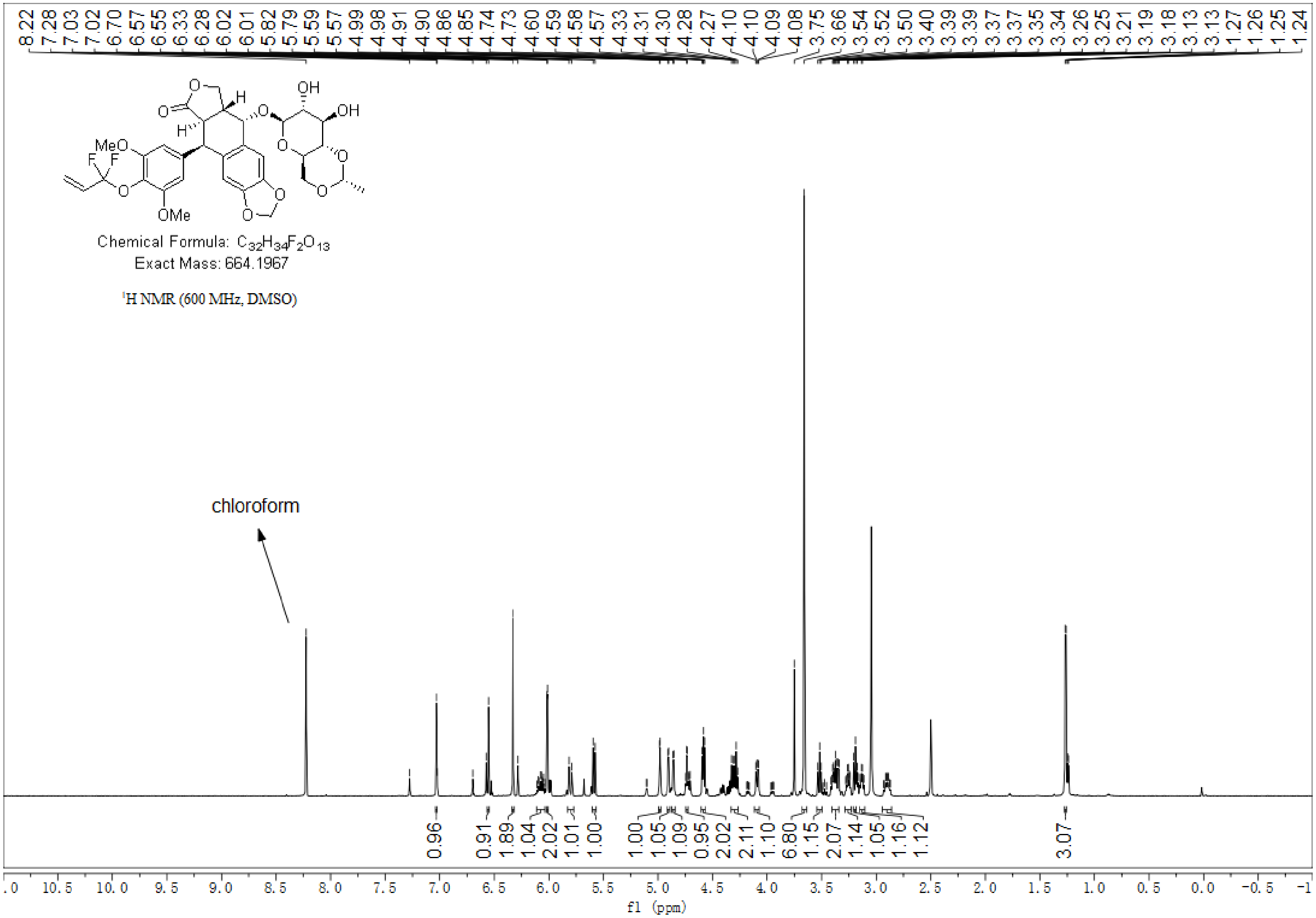

^{19}F NMR spectrum of **3o** (565 MHz, DMSO-d6)

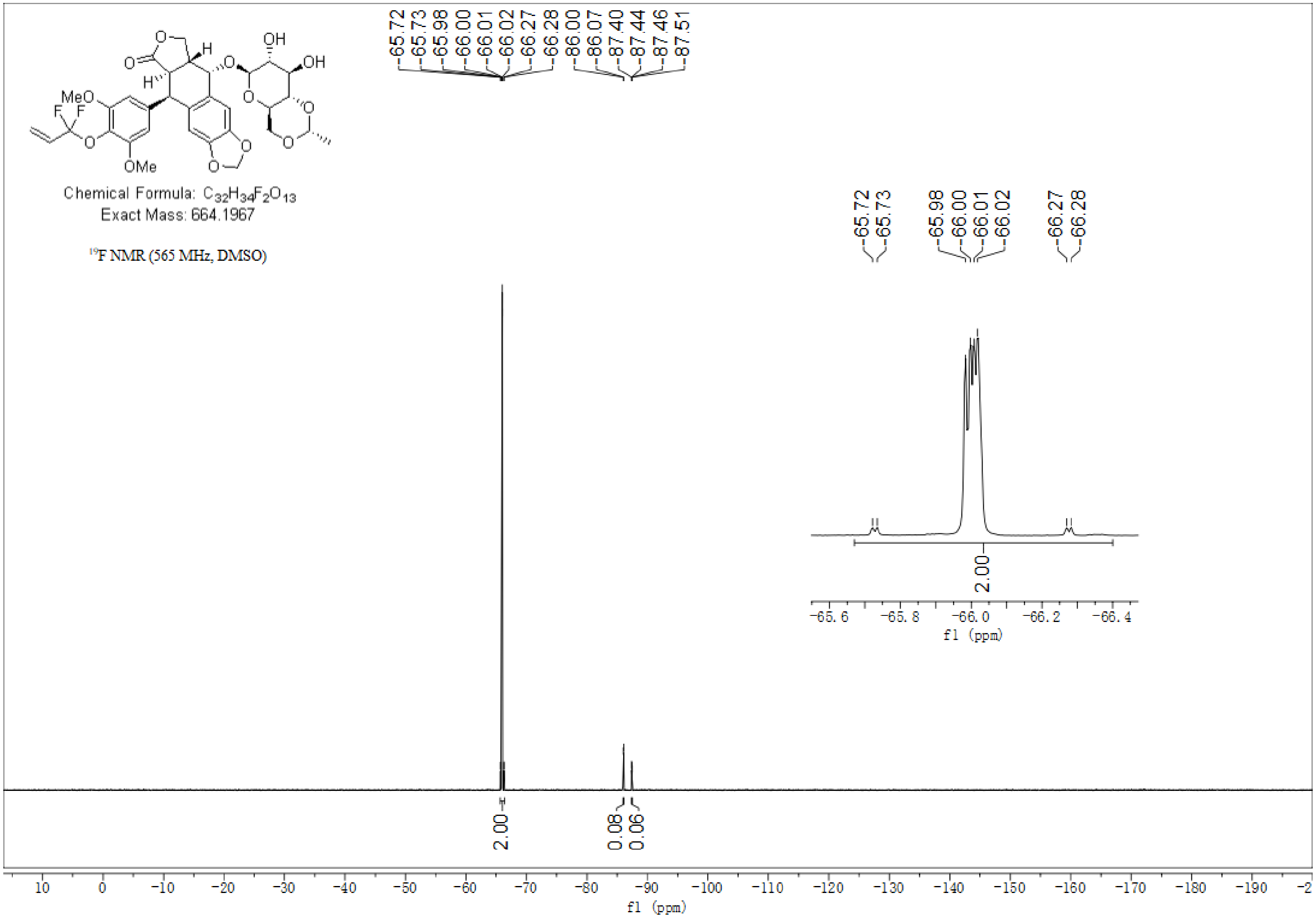

^{13}C NMR spectrum of **3o** (151 MHz, DMSO-d6)

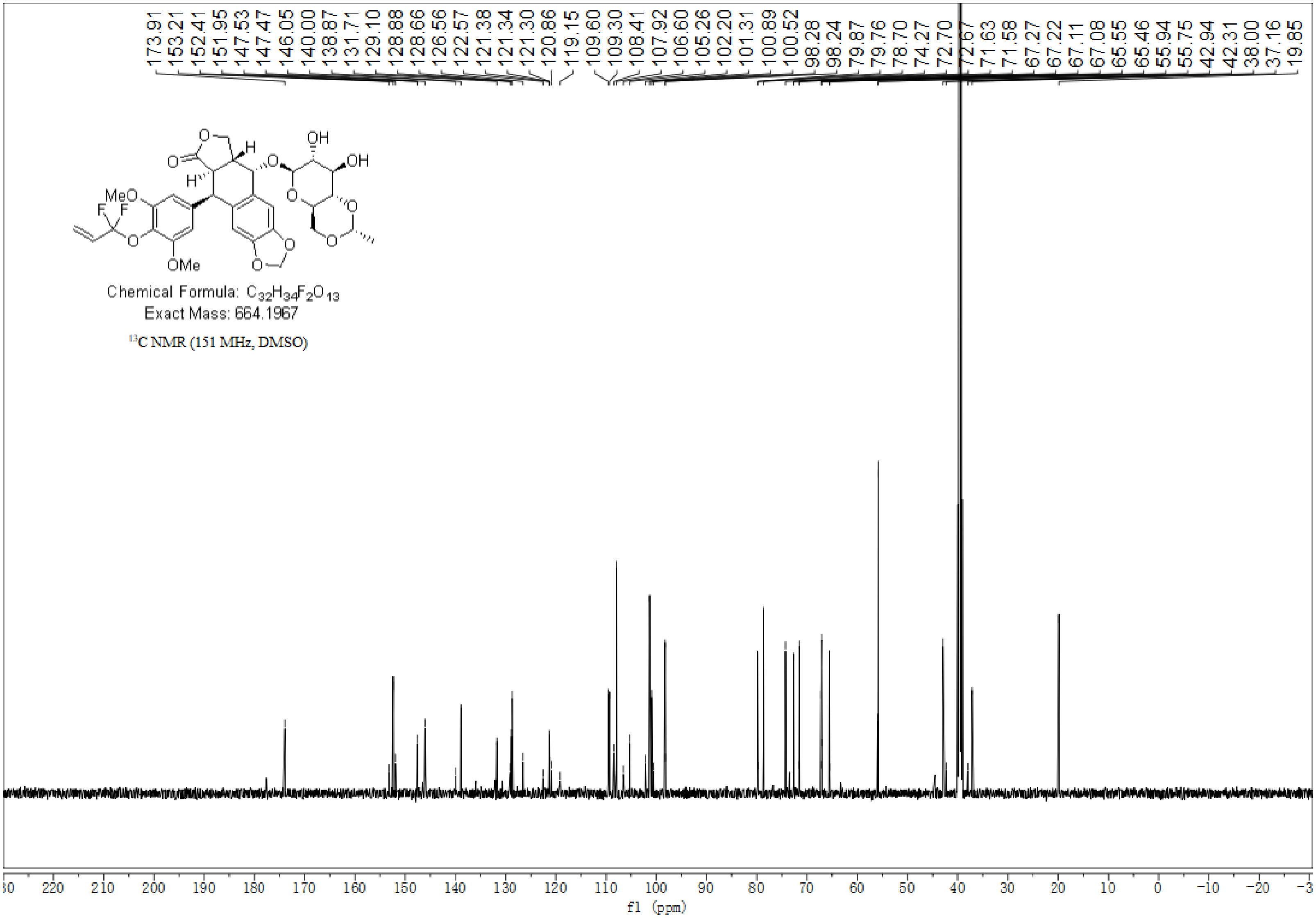

^{1}H NMR spectrum of **3o′** (400 MHz, $CDCl_3$)

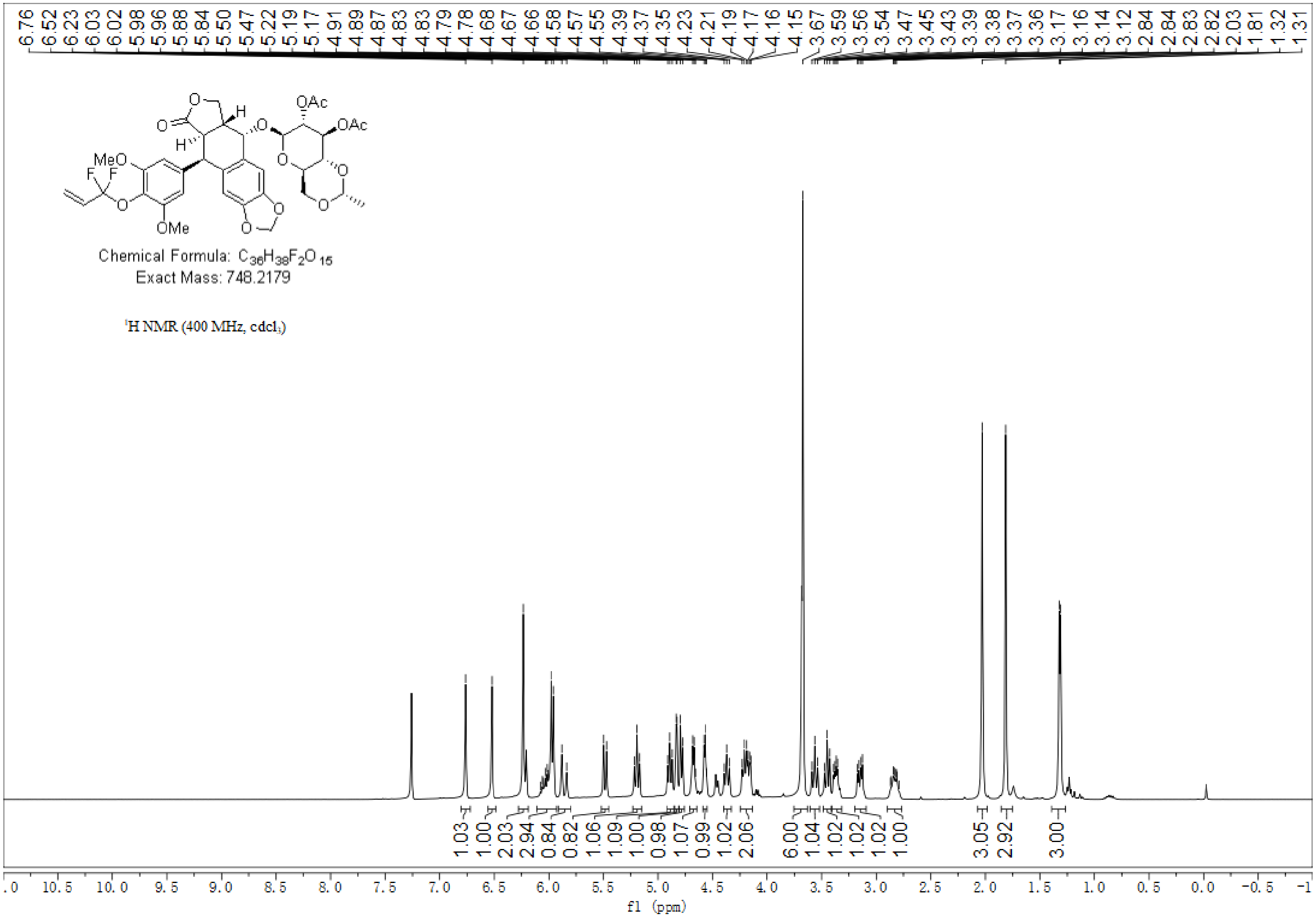

^{19}F NMR spectrum of **3o′** (376 MHz, $CDCl_3$)

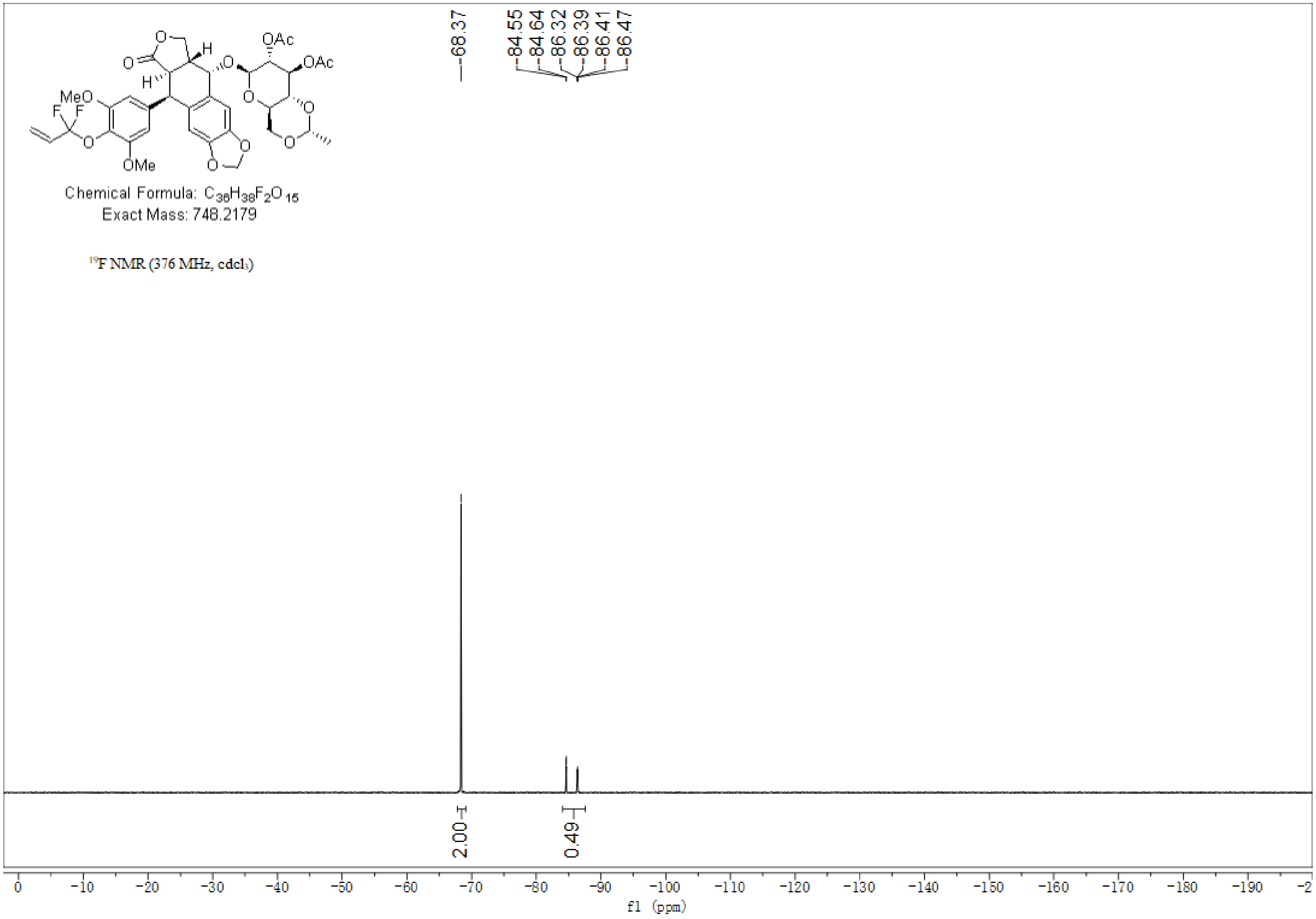

^{13}C NMR spectrum of **3o′**(126 MHz, $CDCl_3$)

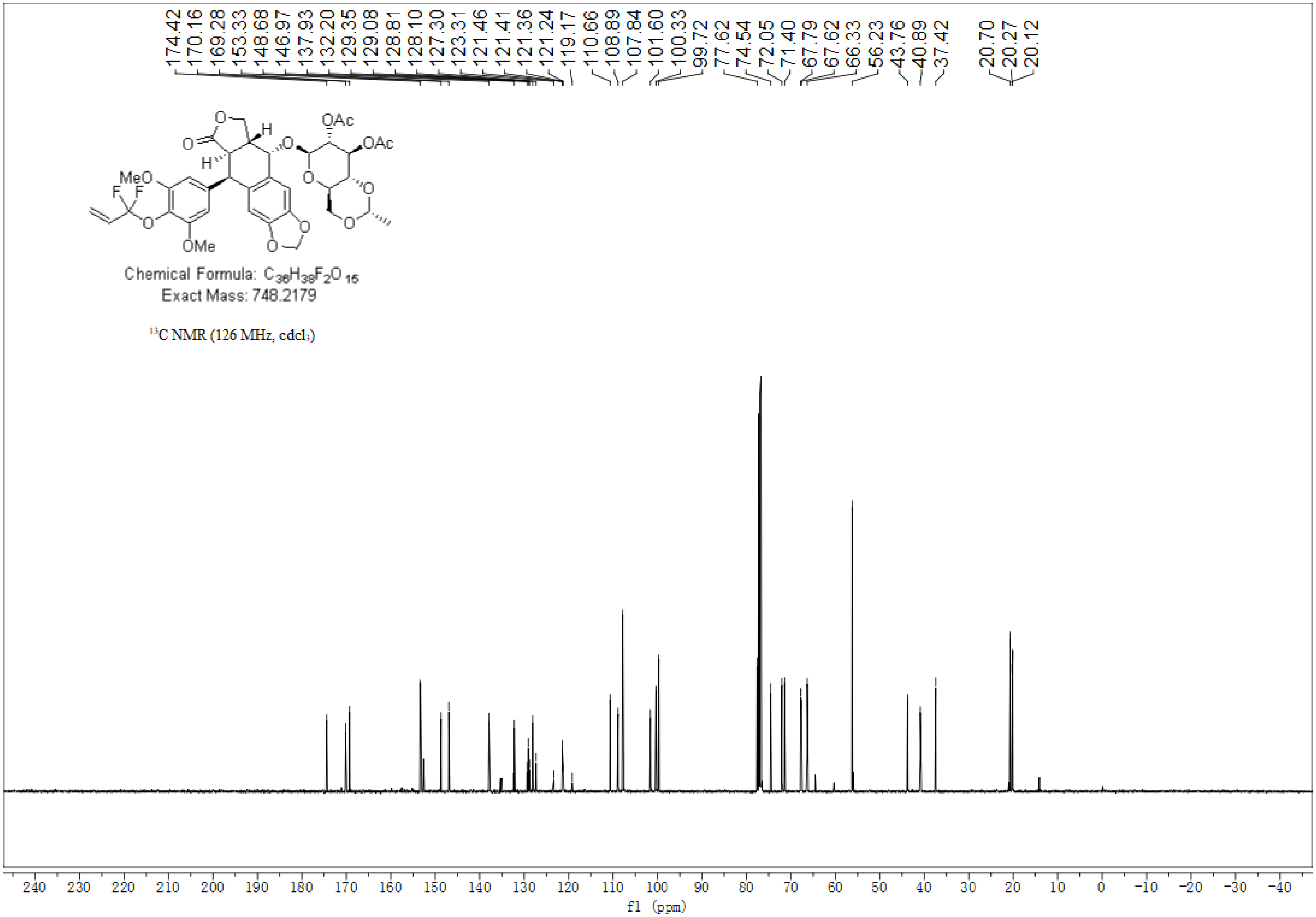

^{1}H NMR spectrum of **3p** (400 MHz, $CDCl_3$)

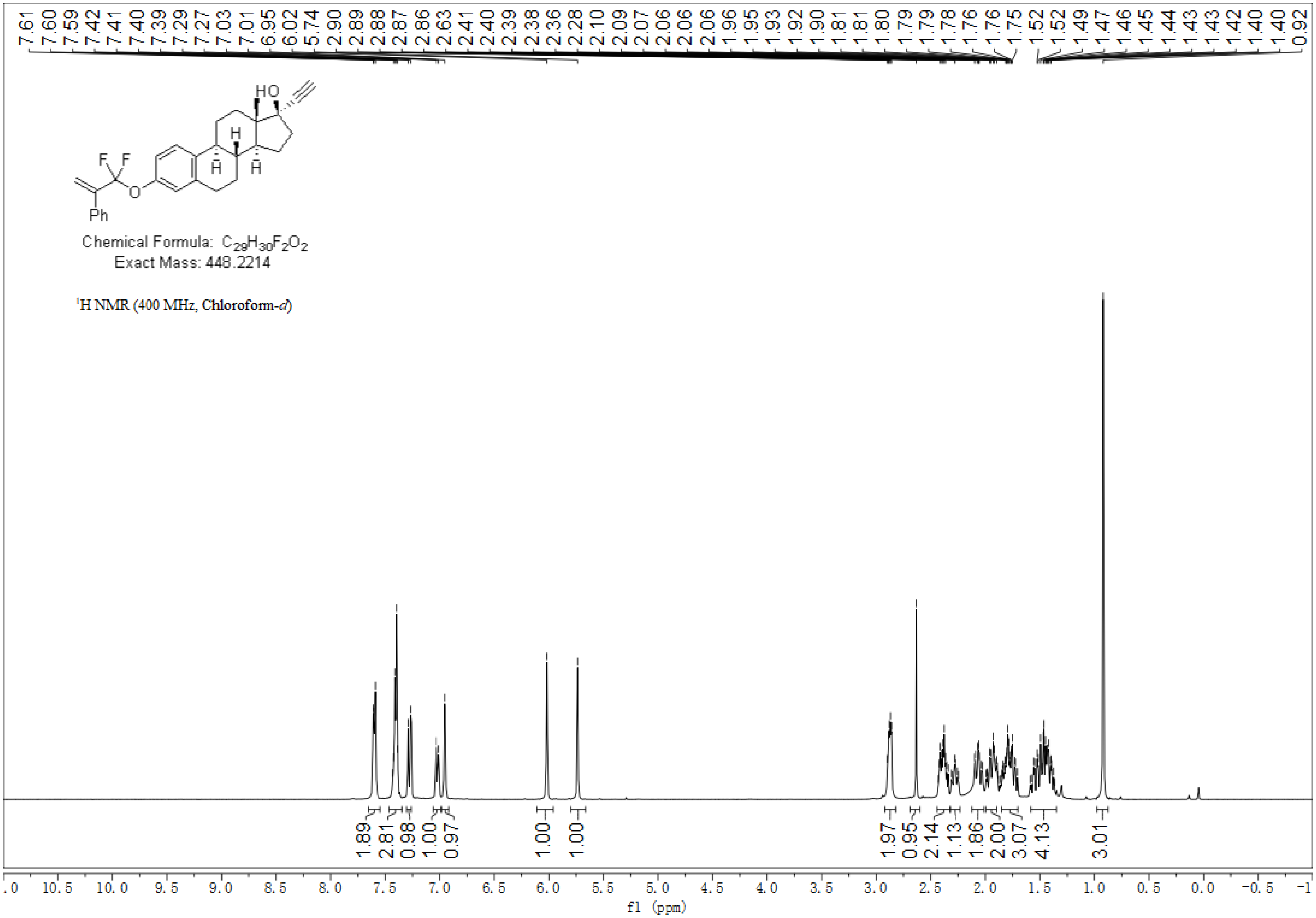

^{19}F NMR spectrum of **3p** (376 MHz, $CDCl_3$)

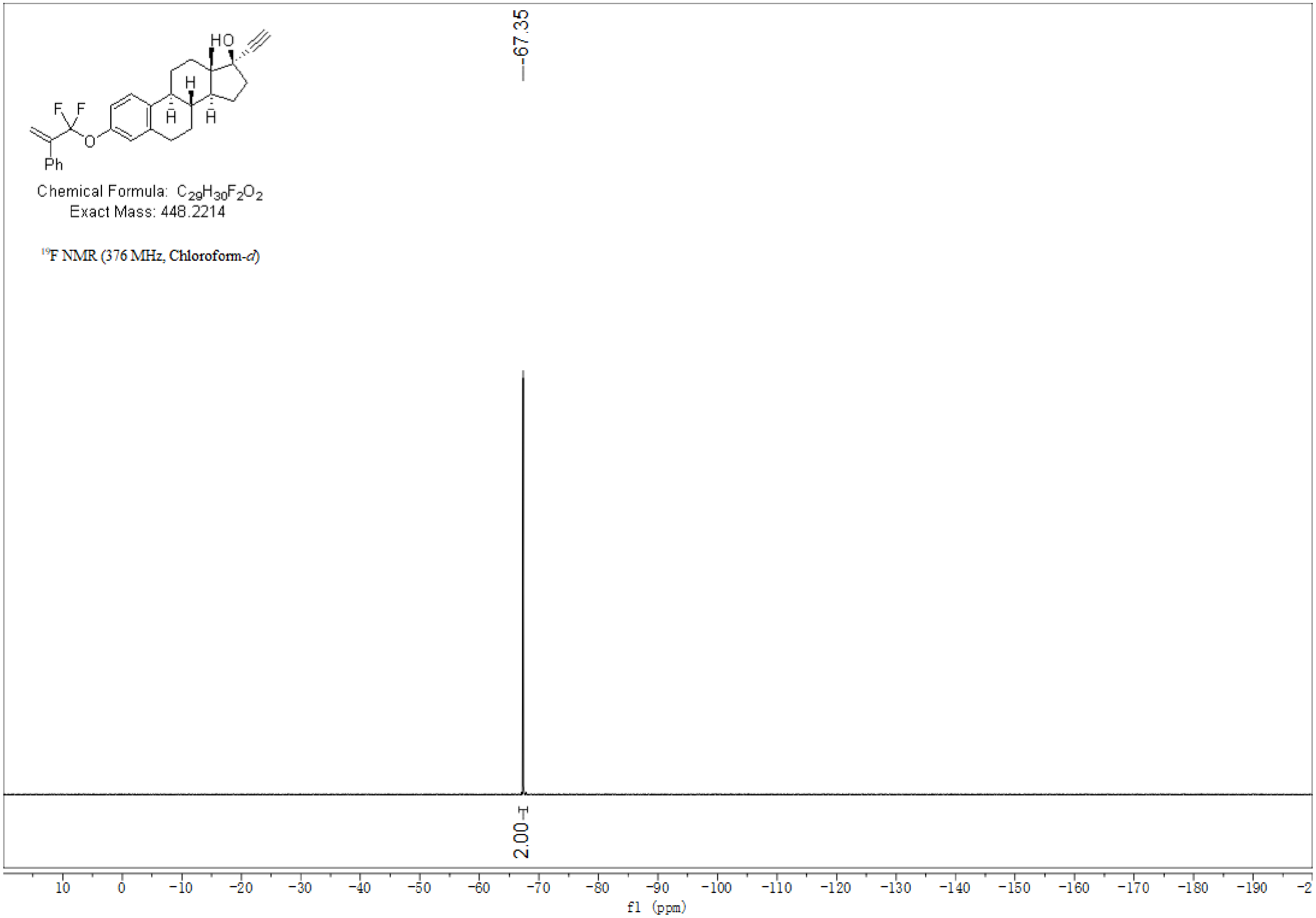

^{13}C NMR spectrum of **3p** (126 MHz, $CDCl_3$)

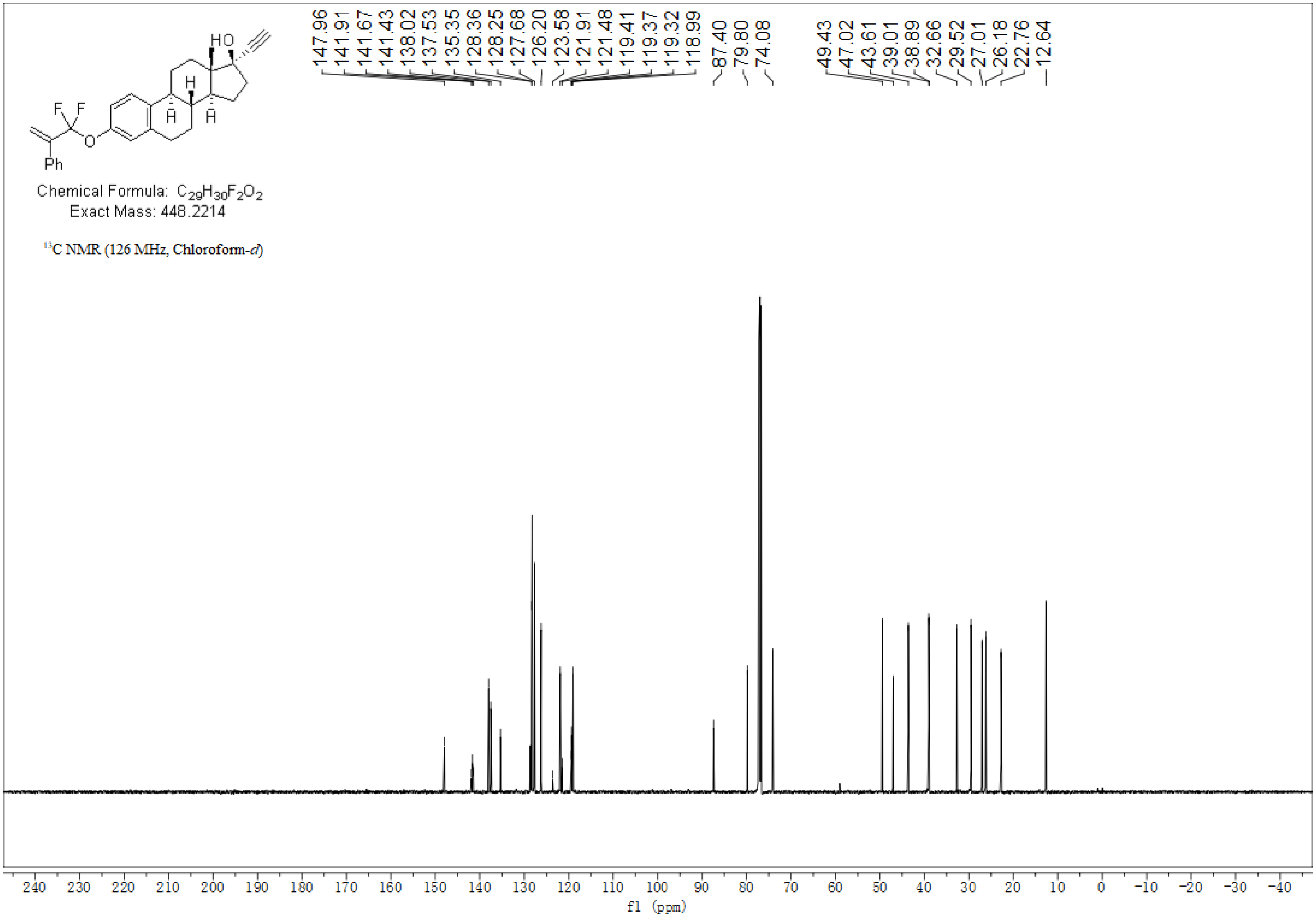

^{1}H NMR spectrum of **3q** (400 MHz, $CDCl_3$)

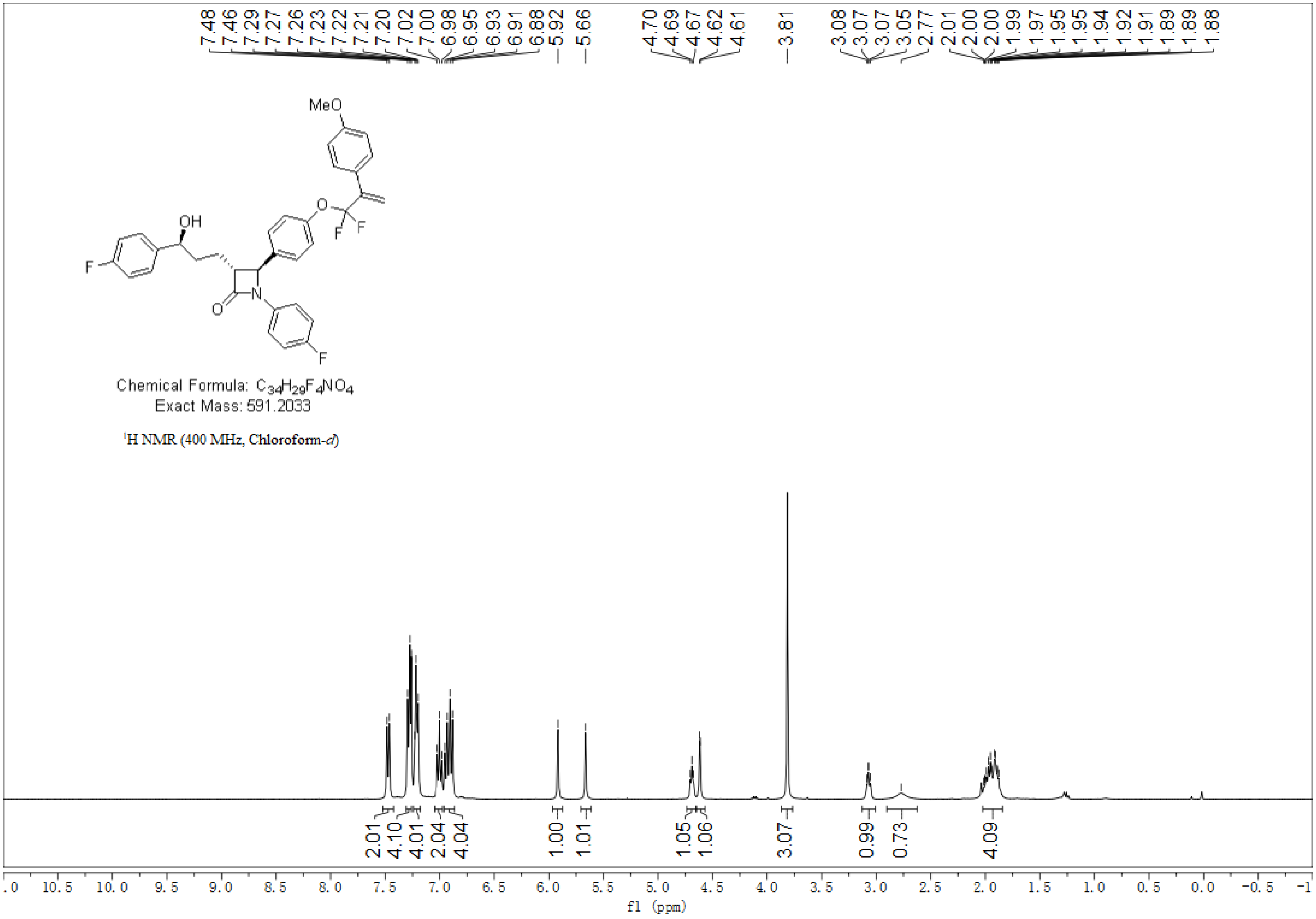

^{19}F NMR spectrum of **3q** (376 MHz, $CDCl_3$)

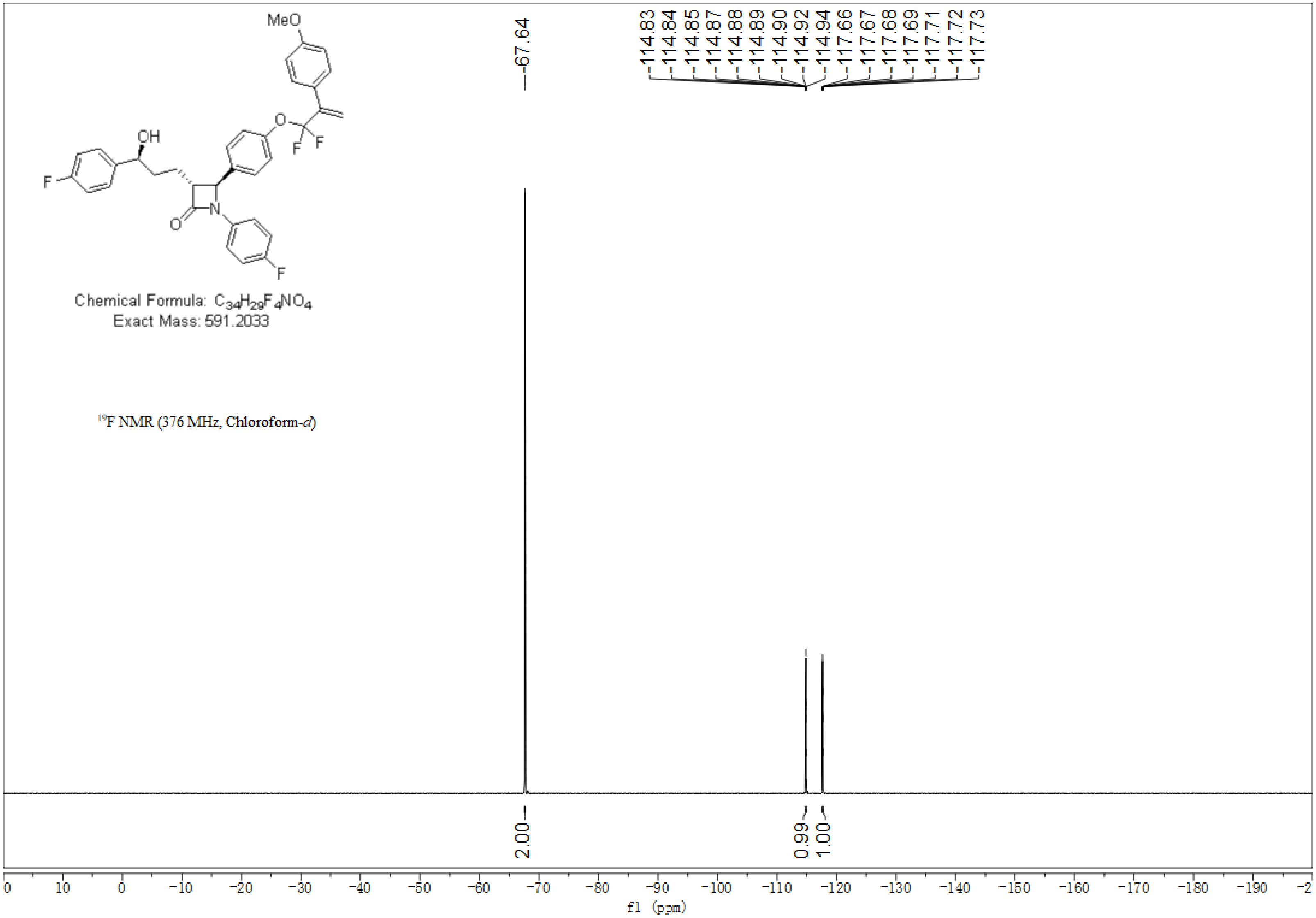

^{13}C NMR spectrum of **3q** (126 MHz, $CDCl_3$)

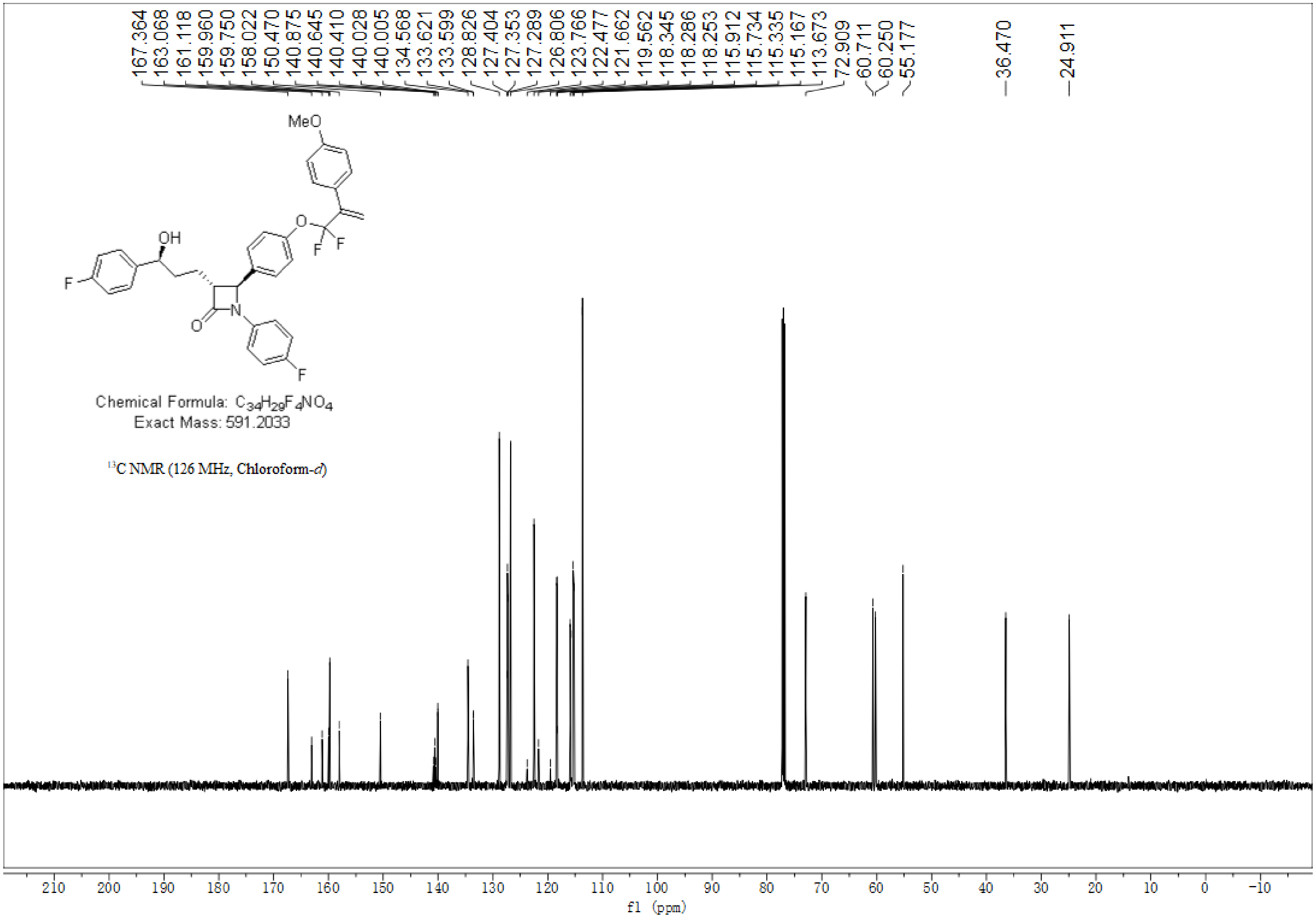

^{1}H NMR spectrum of **3r** (400 MHz, $CDCl_3$)

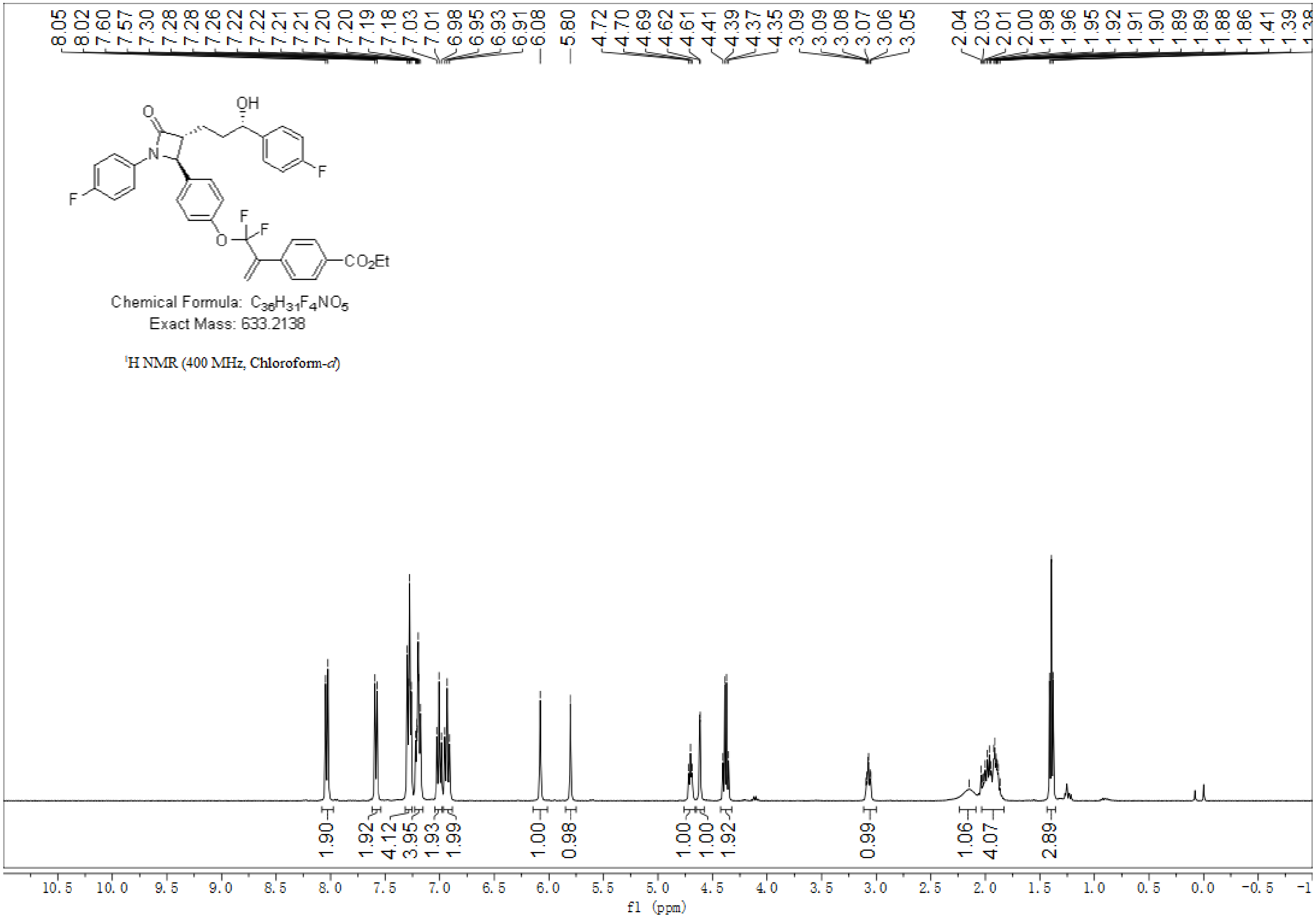

^{19}F NMR spectrum of **3r** (376 MHz, $CDCl_3$)

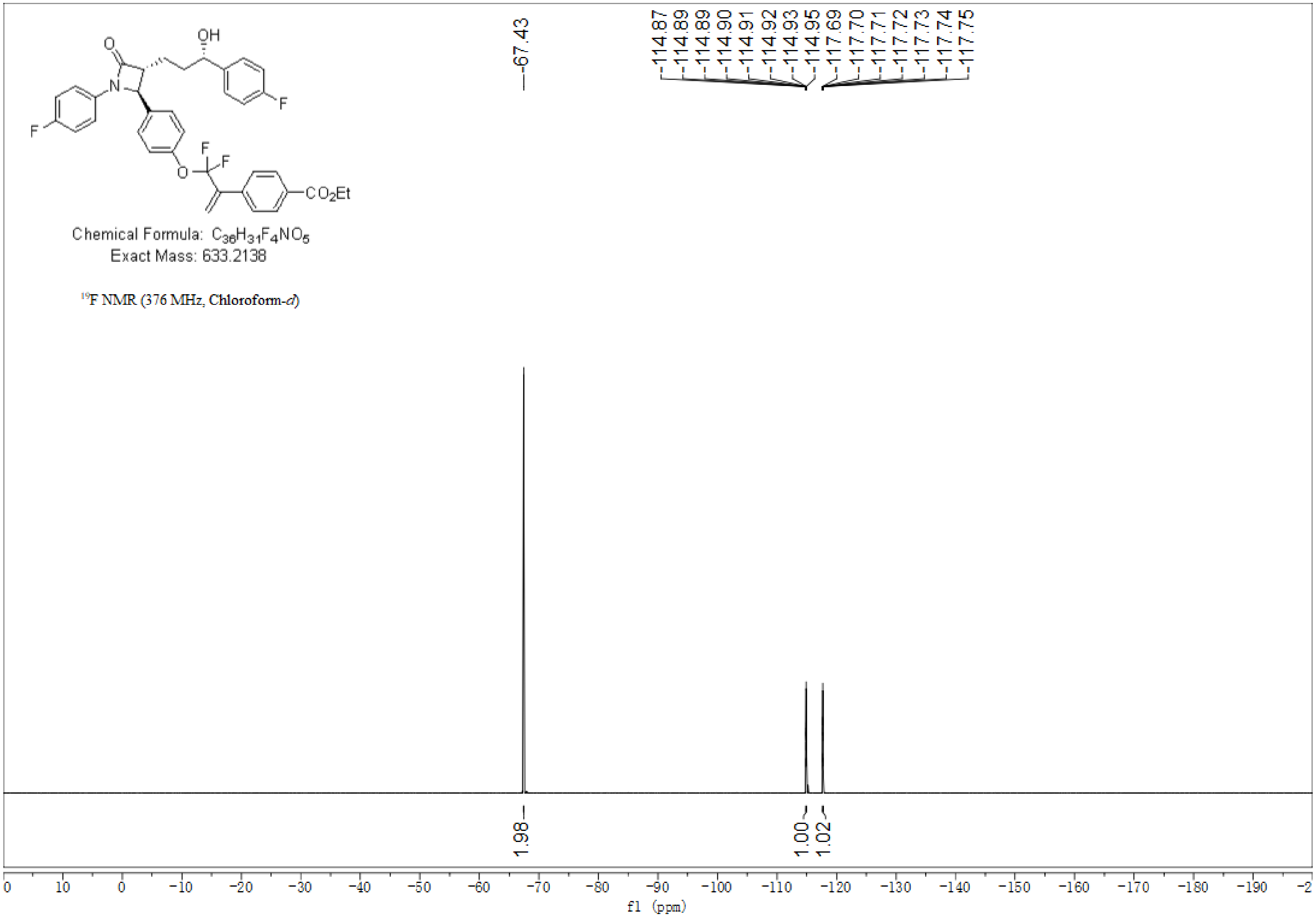

^{13}C NMR spectrum of **3r** (126 MHz, $CDCl_3$)

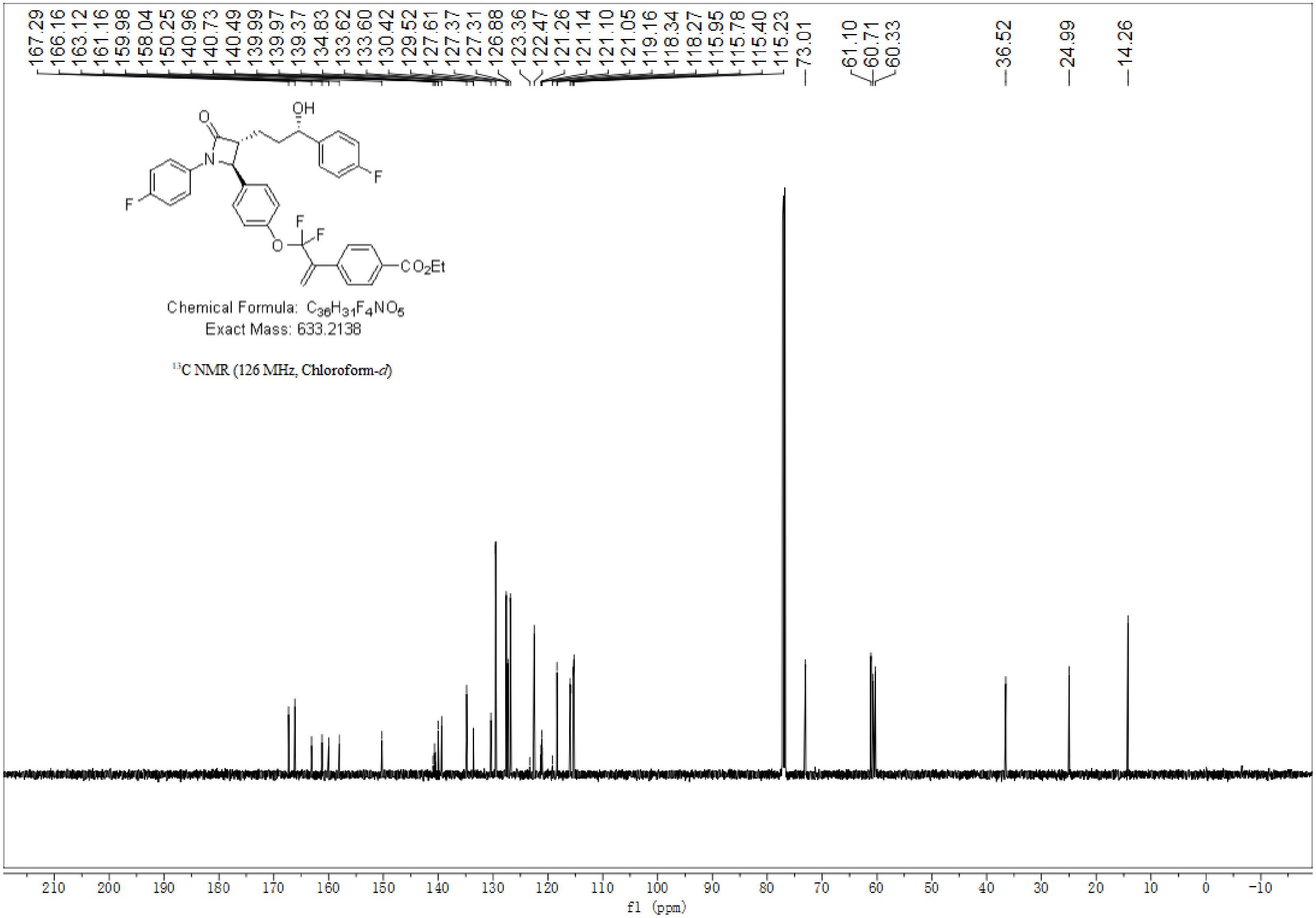

^{1}H NMR spectrum of **3s** (500 MHz, $CDCl_3$)

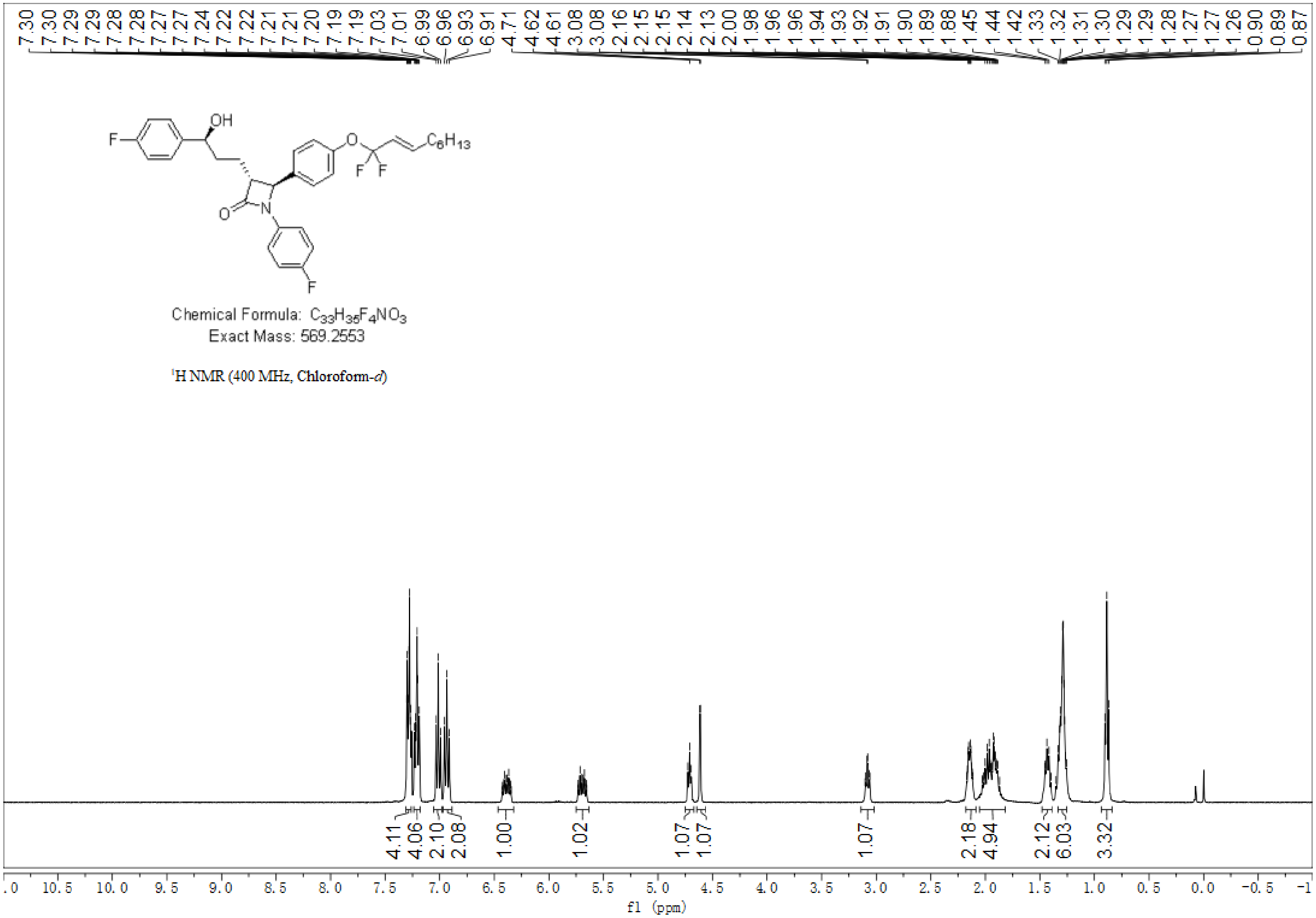

^{19}F NMR spectrum of **3s** (376 MHz, $CDCl_3$)

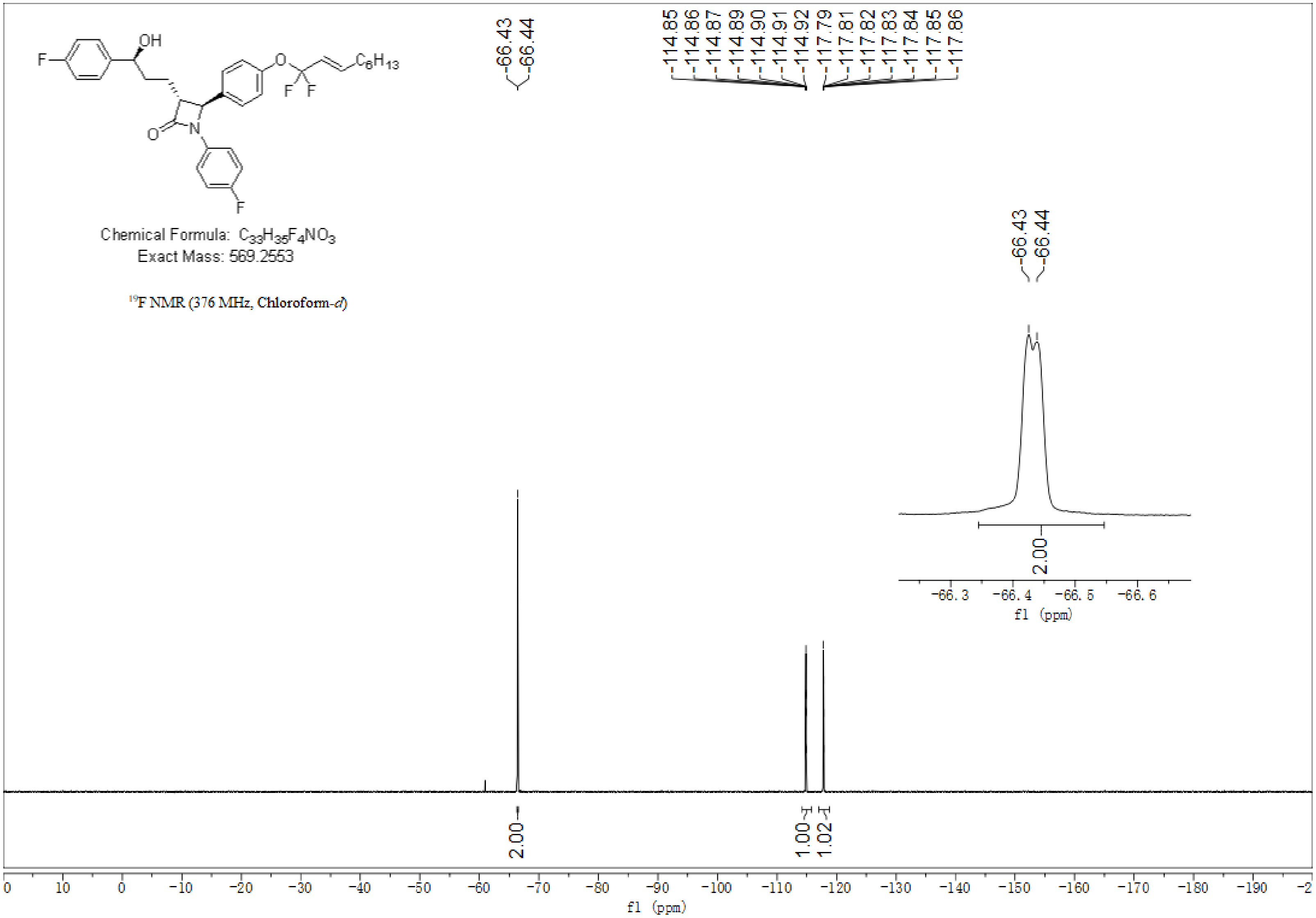

^{13}C NMR spectrum of **3s** (151 MHz, $CDCl_3$)

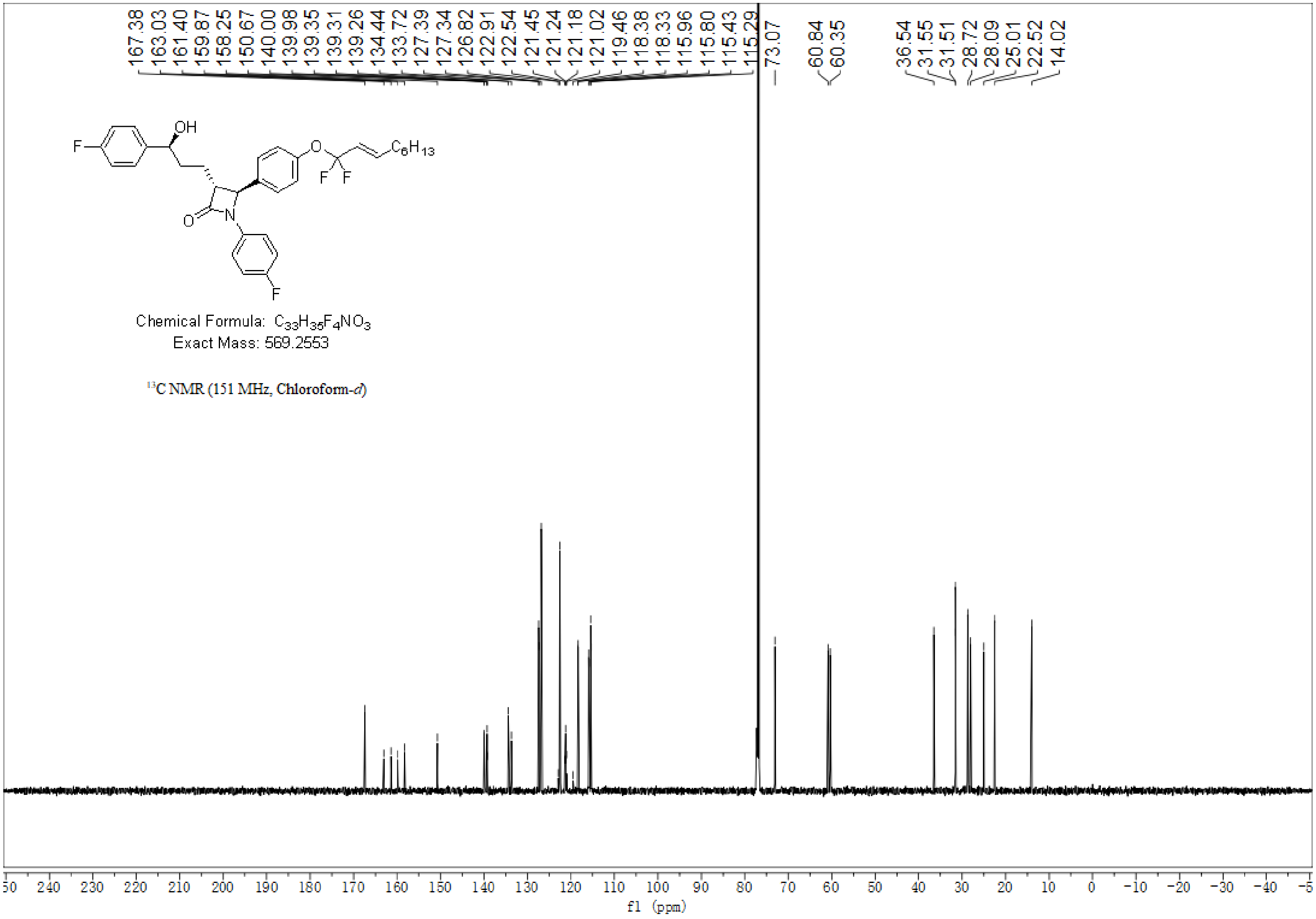

^{1}H NMR spectrum of **3t** (400 MHz, $CDCl_3$)

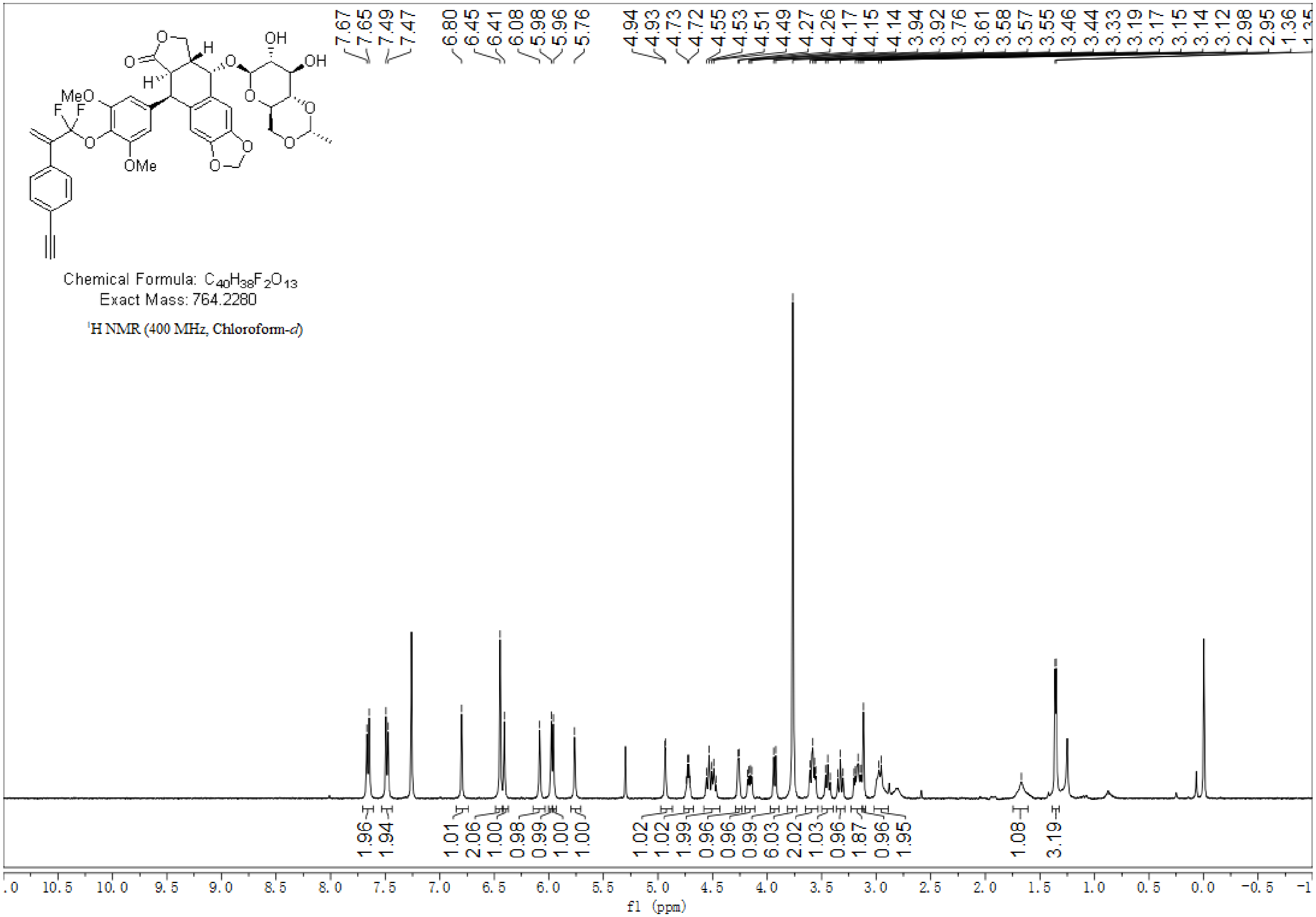

^{19}F NMR spectrum of **3t** (376 MHz, $CDCl_3$)

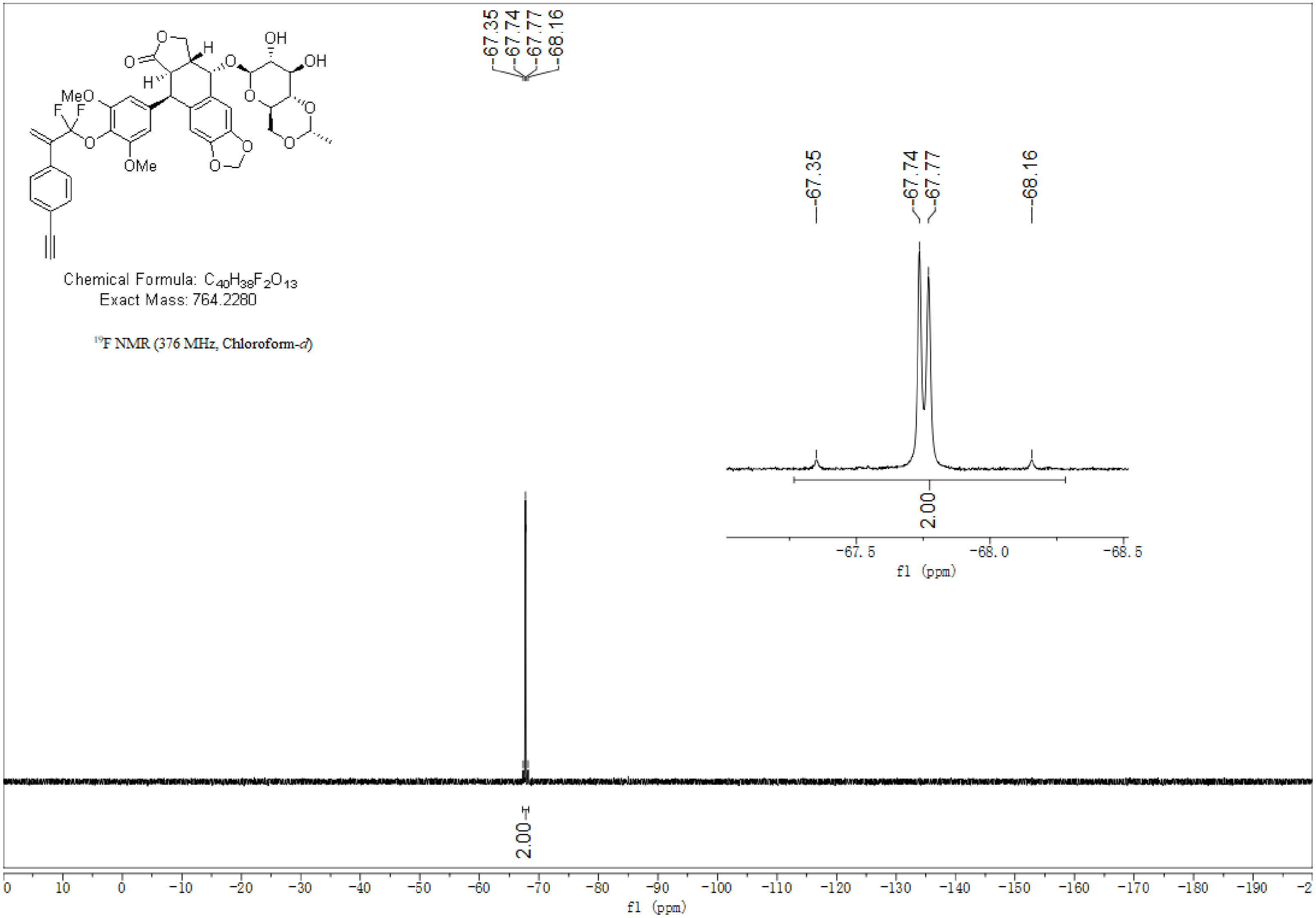

^{13}C NMR spectrum of **3t** (126 MHz, $CDCl_3$)

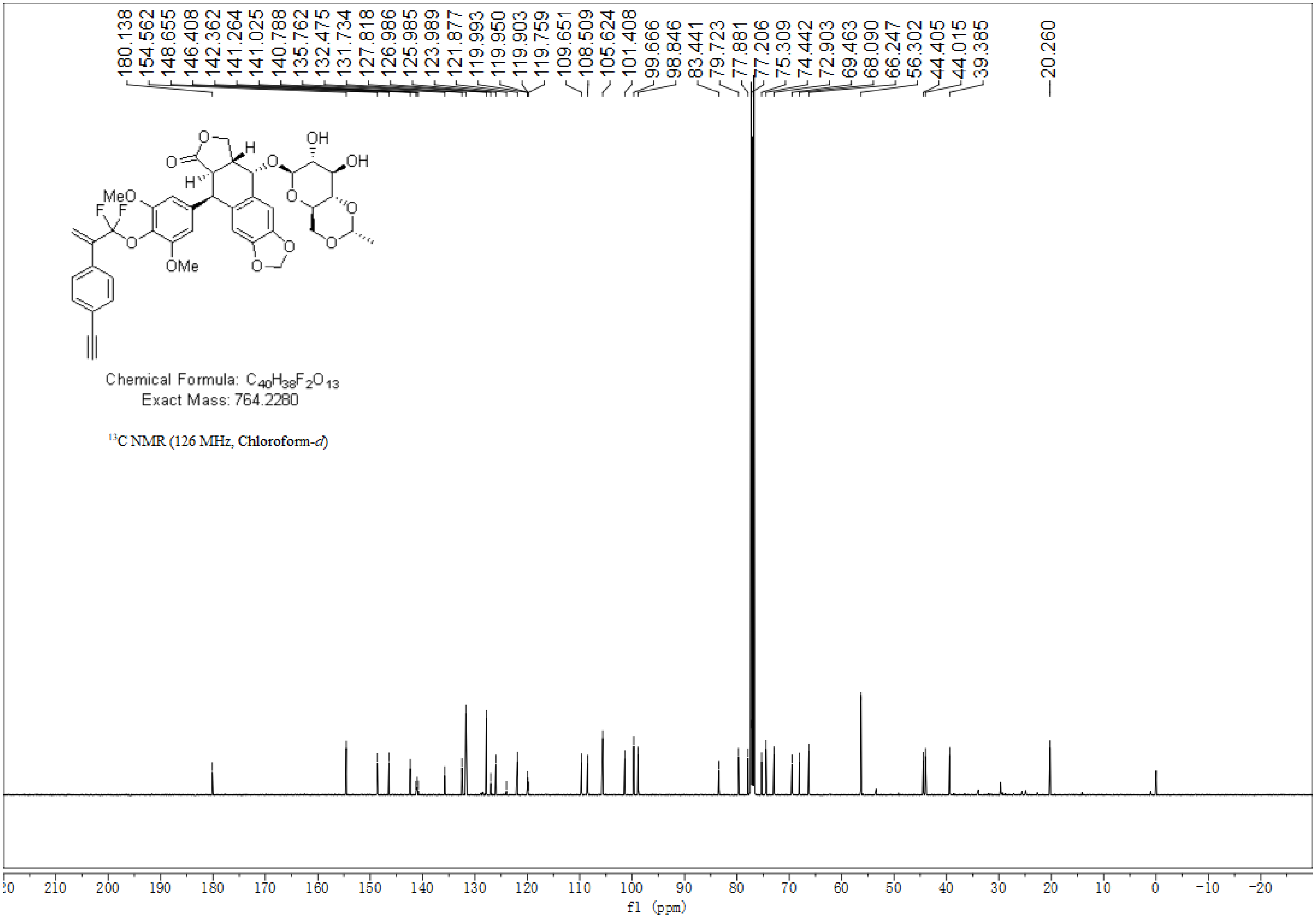

^{1}H NMR spectrum of **3u** (400 MHz, $CDCl_3$)

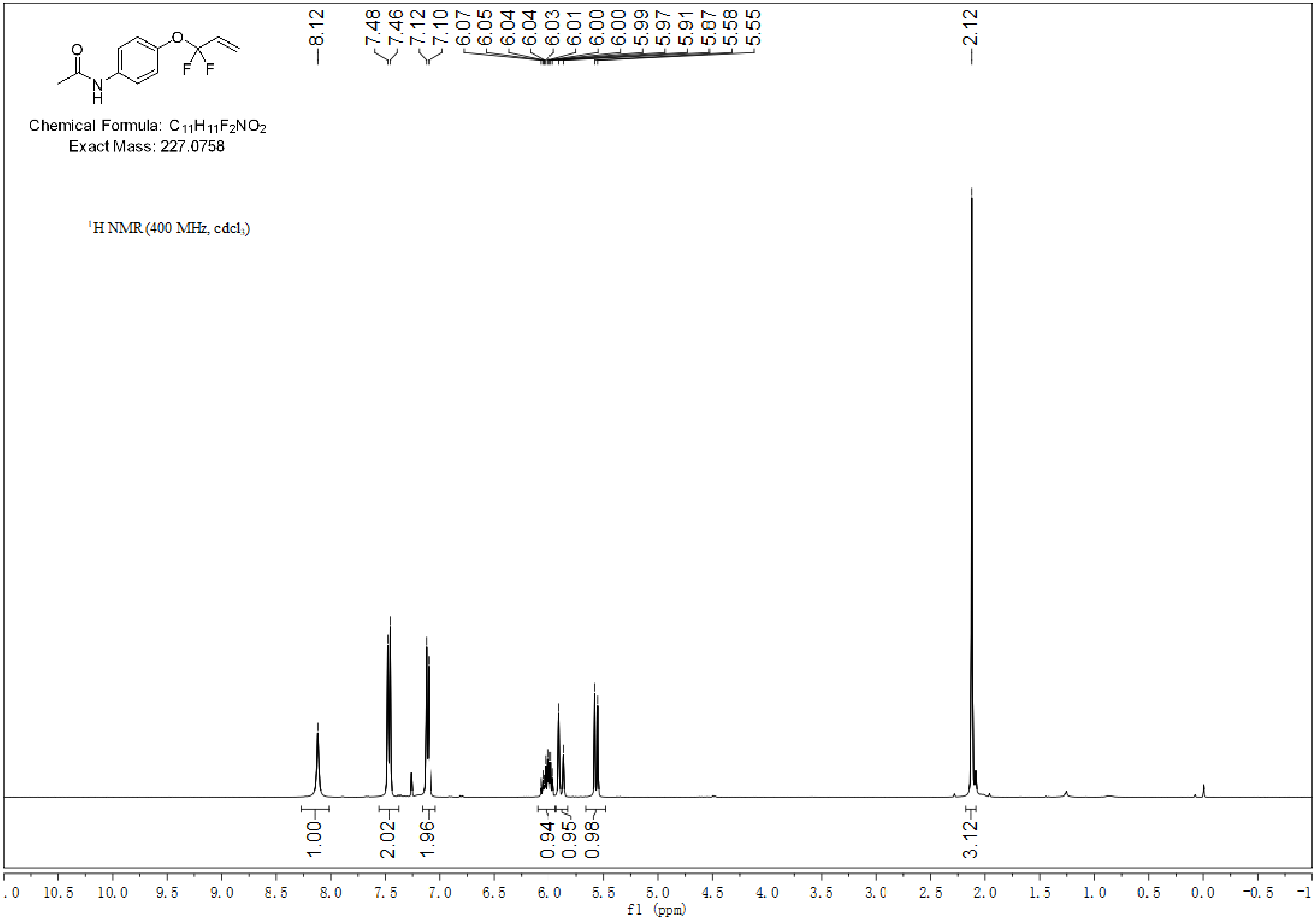

^{19}F NMR spectrum of **3u** (376 MHz, $CDCl_3$)

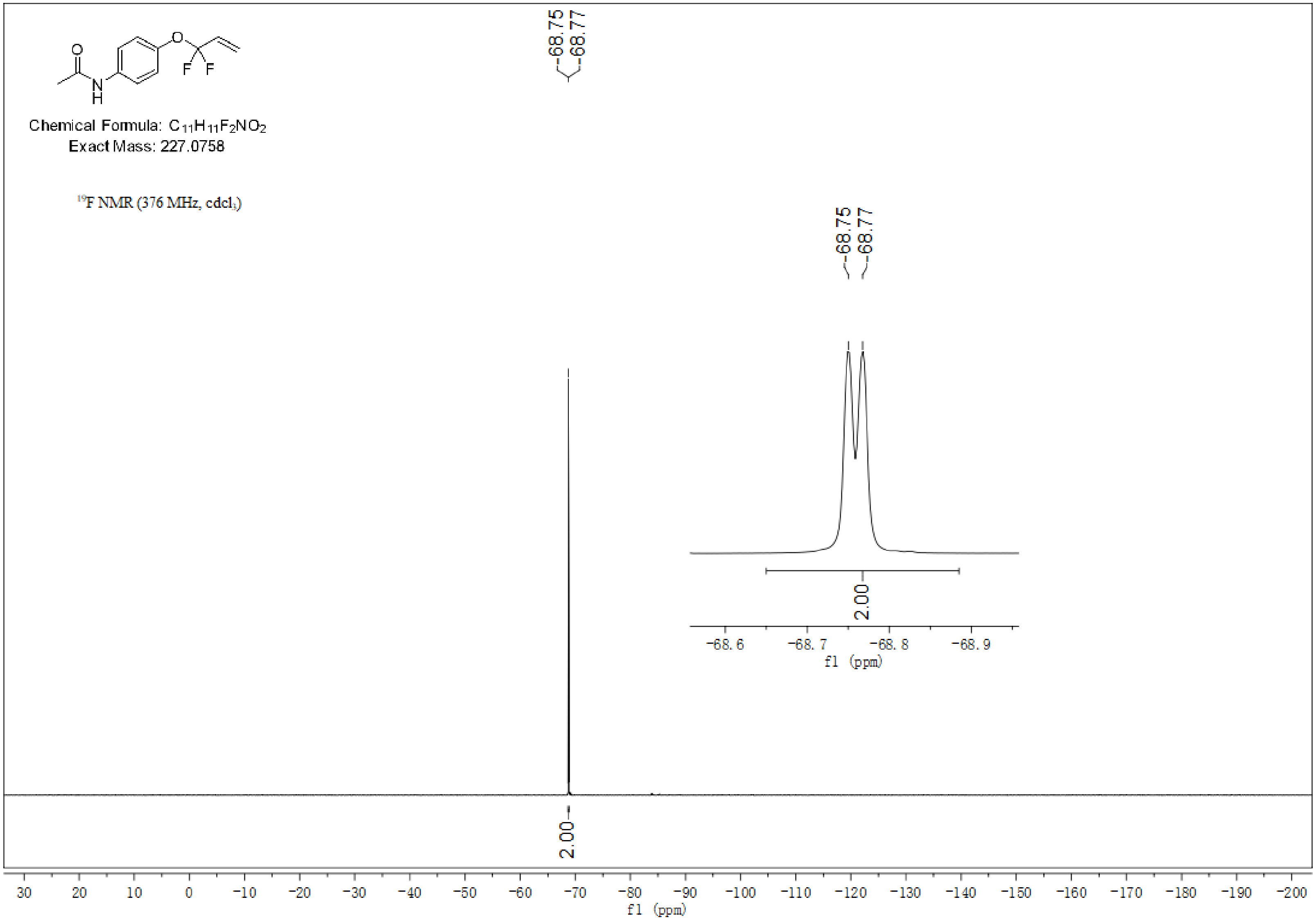

^{13}C NMR spectrum of **3u** (126 MHz, $CDCl_3$)

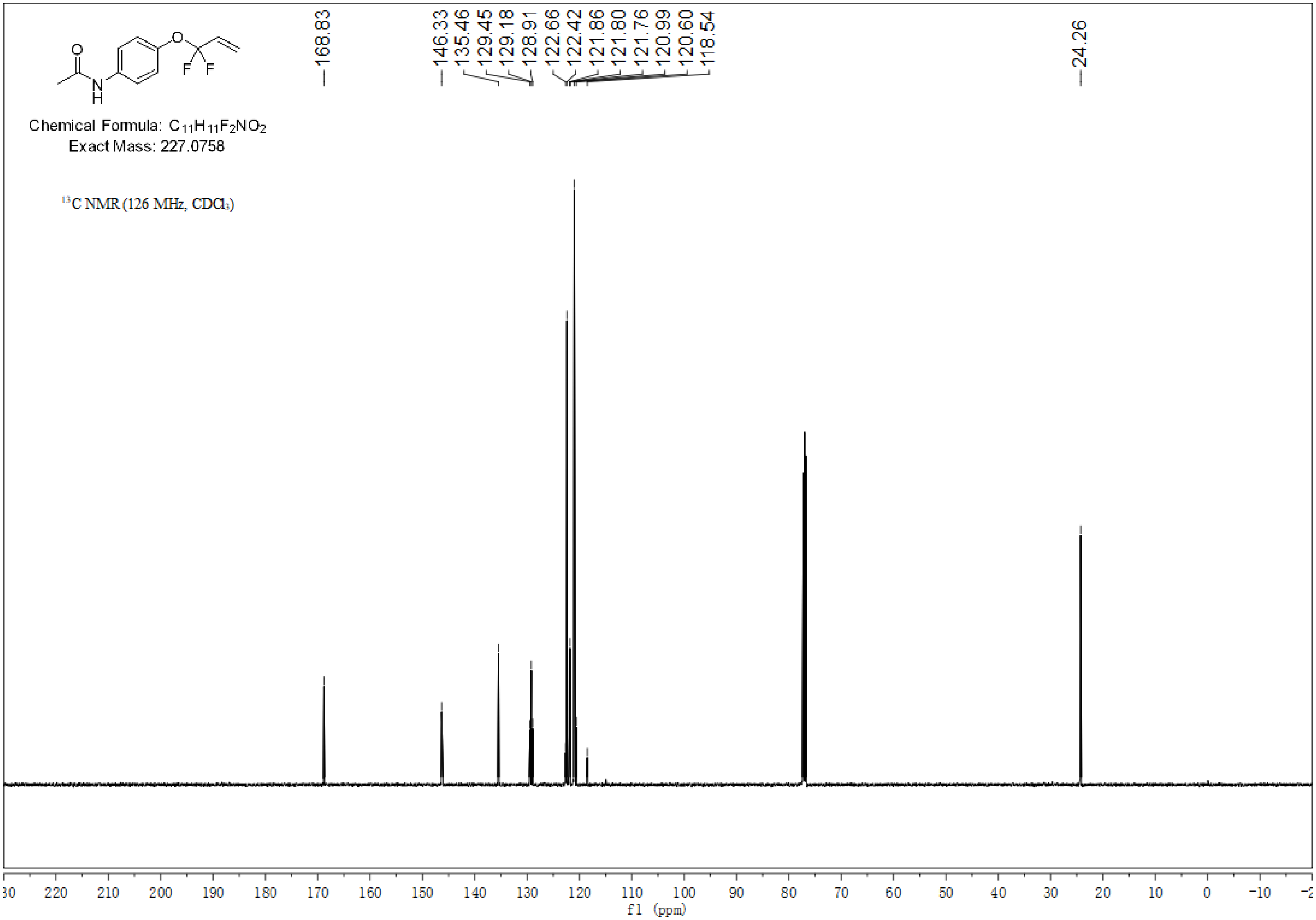

^{1}H NMR spectrum of **3v** (400 MHz, $CDCl_3$)

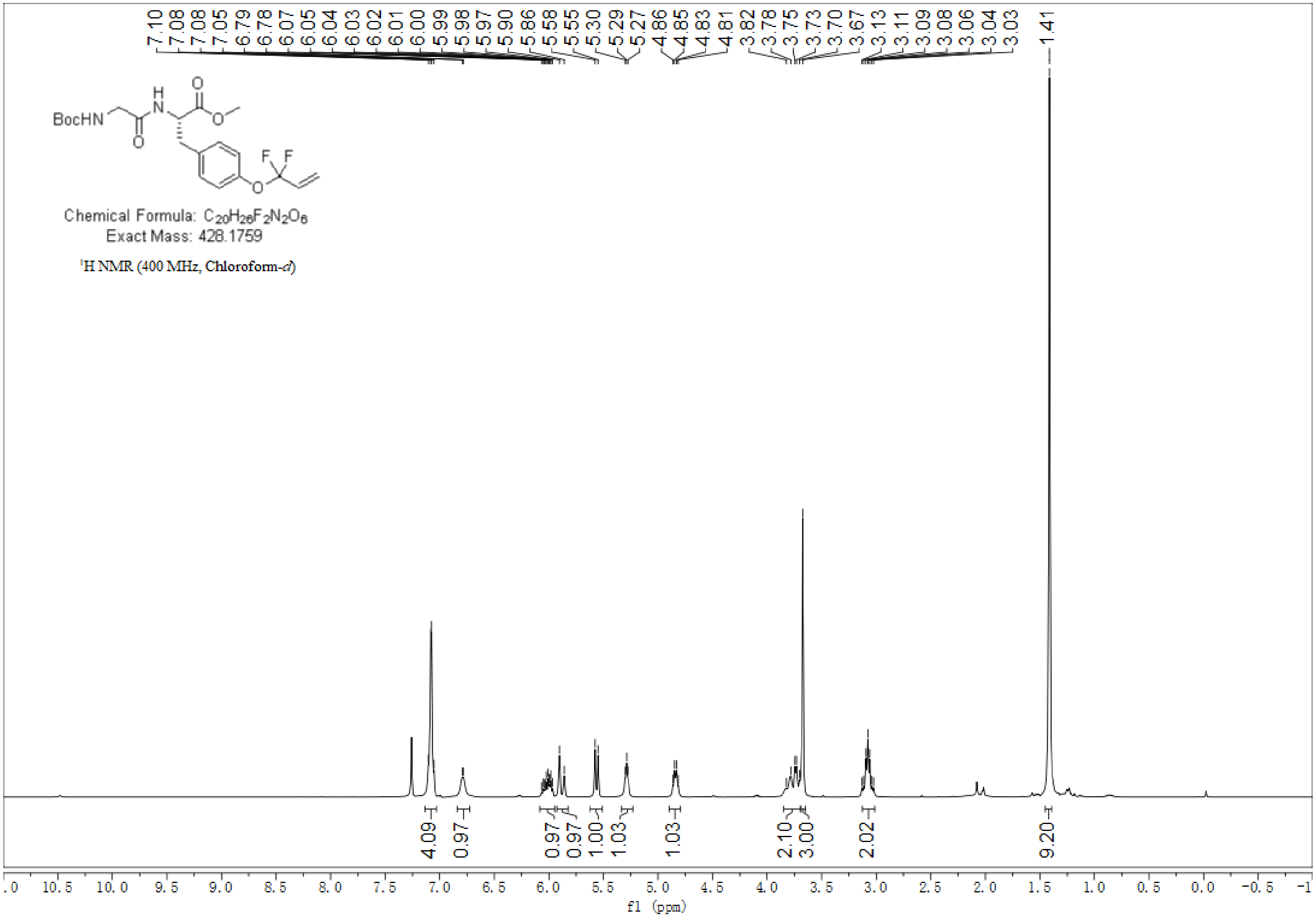

^{19}F NMR spectrum of **3v** (376 MHz, $CDCl_3$)

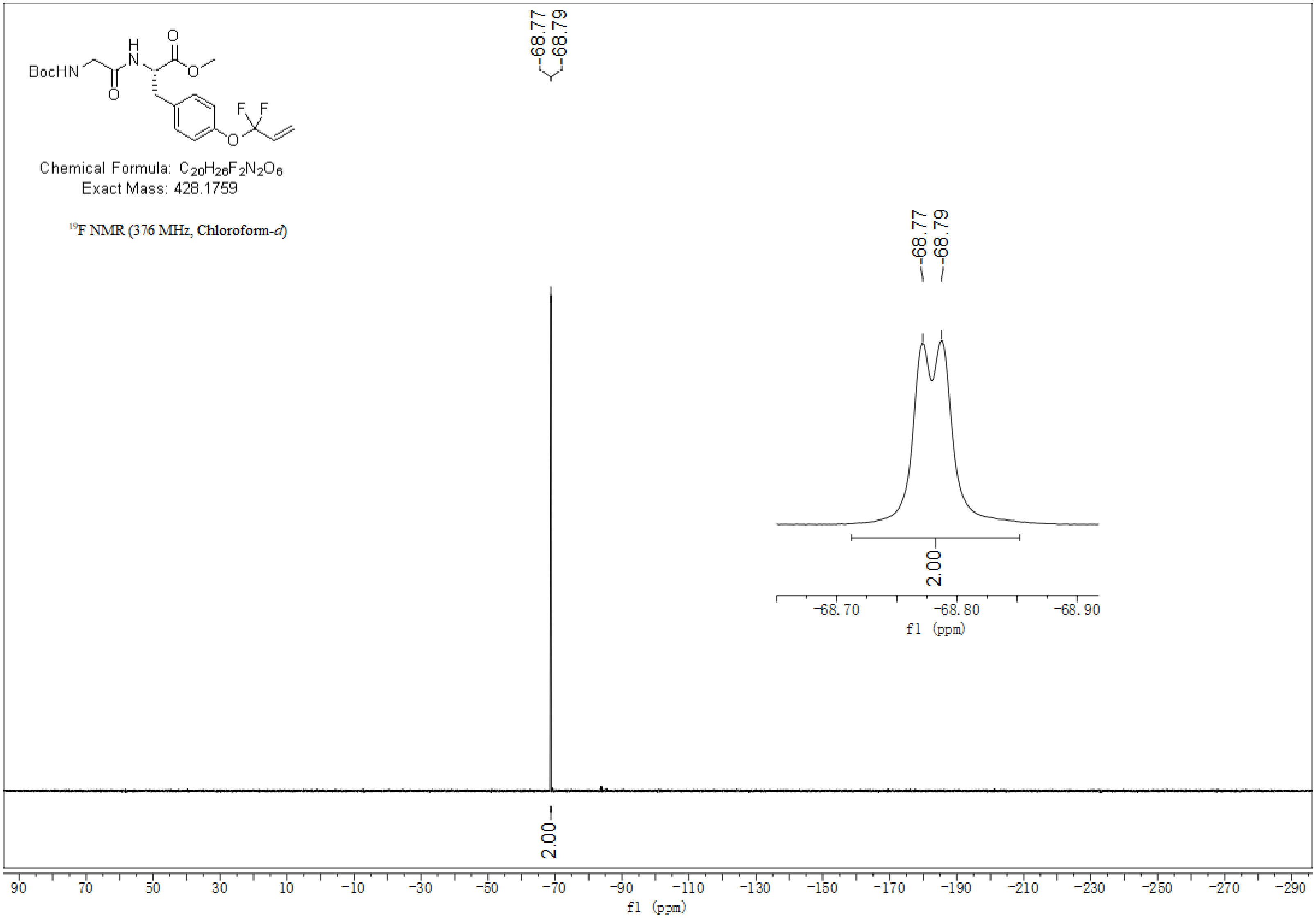

^{13}C NMR spectrum of **3v** (126 MHz, $CDCl_3$)

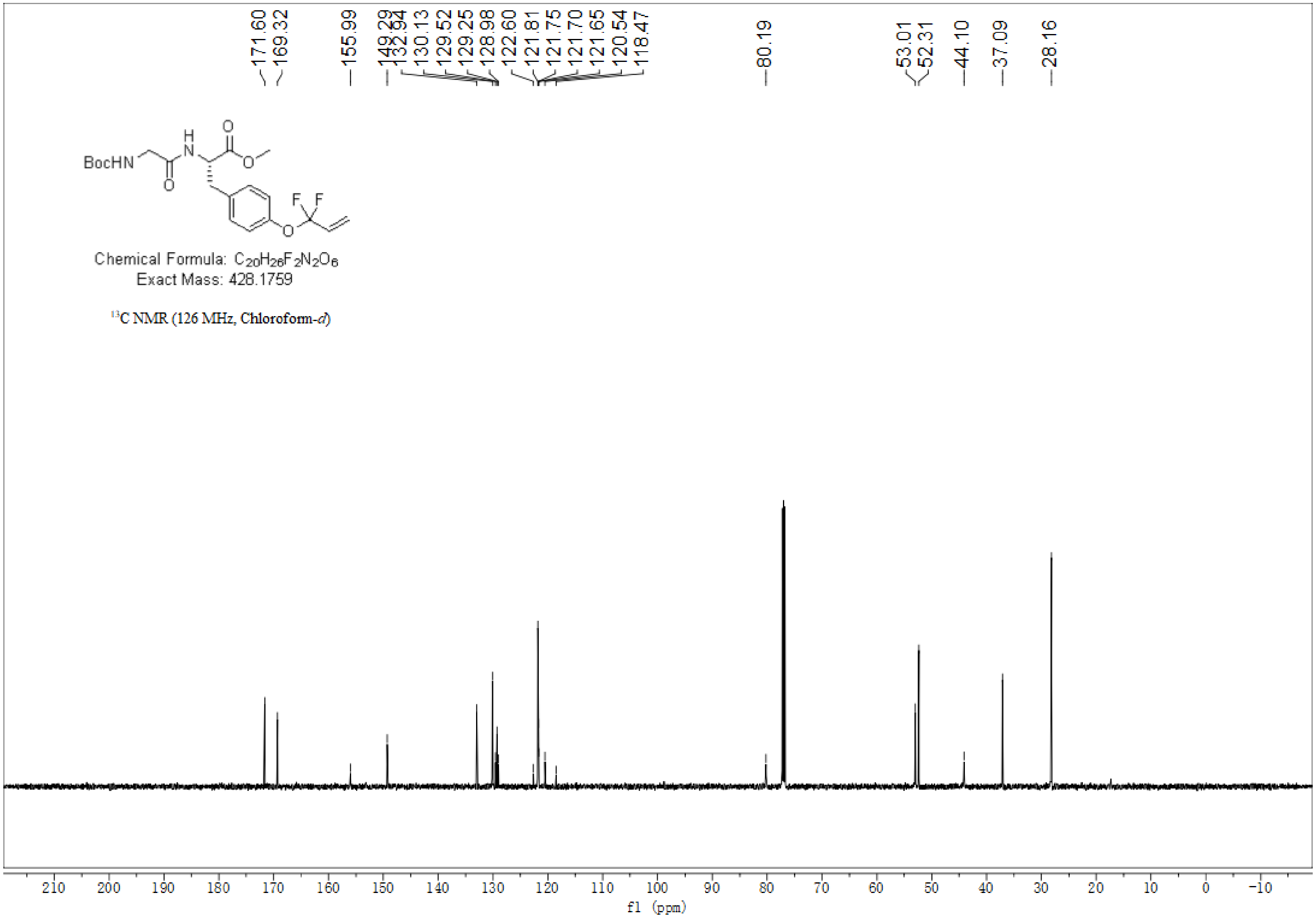

^{1}H NMR spectrum of **3w** (400 MHz, $CDCl_3$)

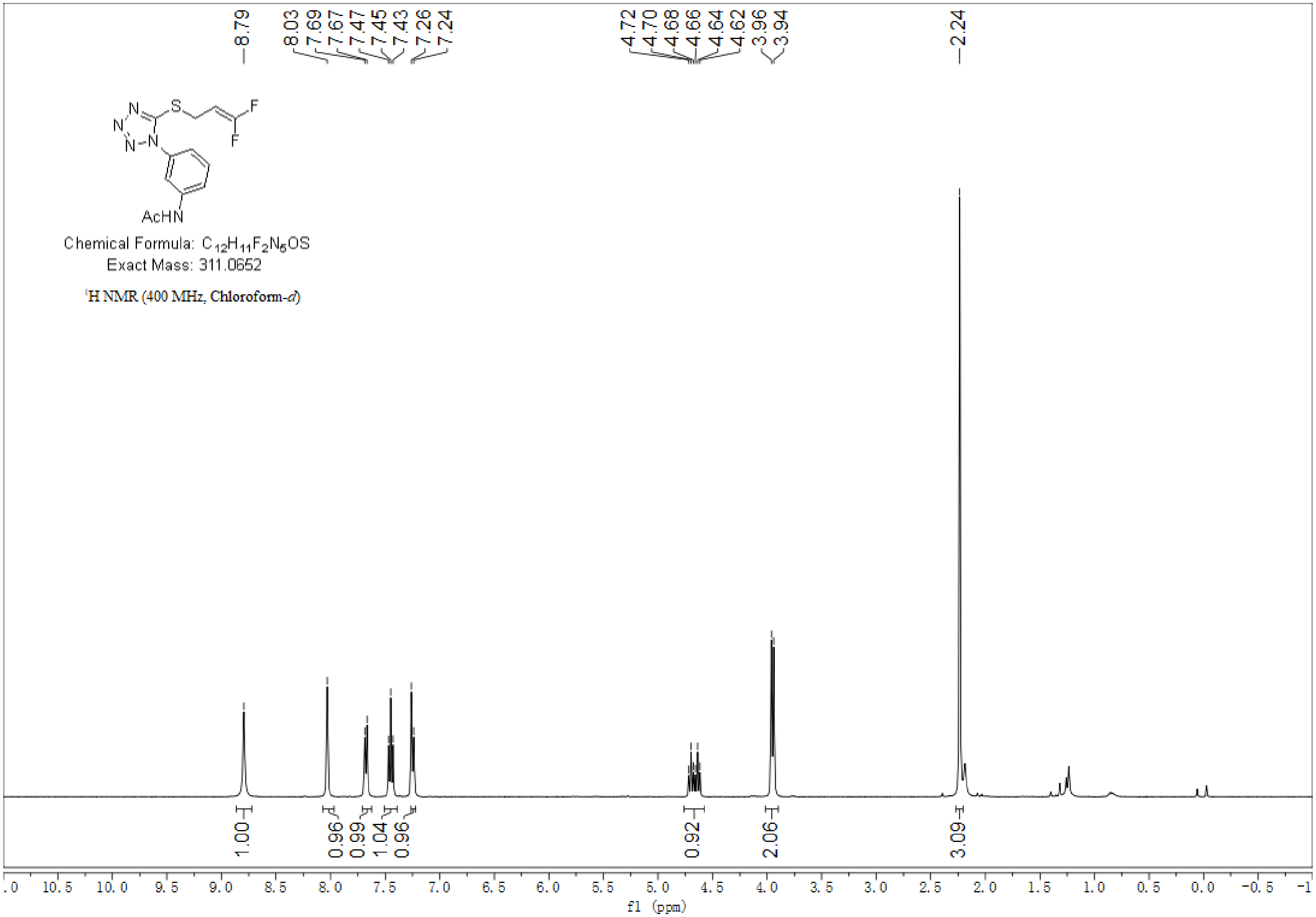

^{19}F NMR spectrum of **3w** (376 MHz, $CDCl_3$)

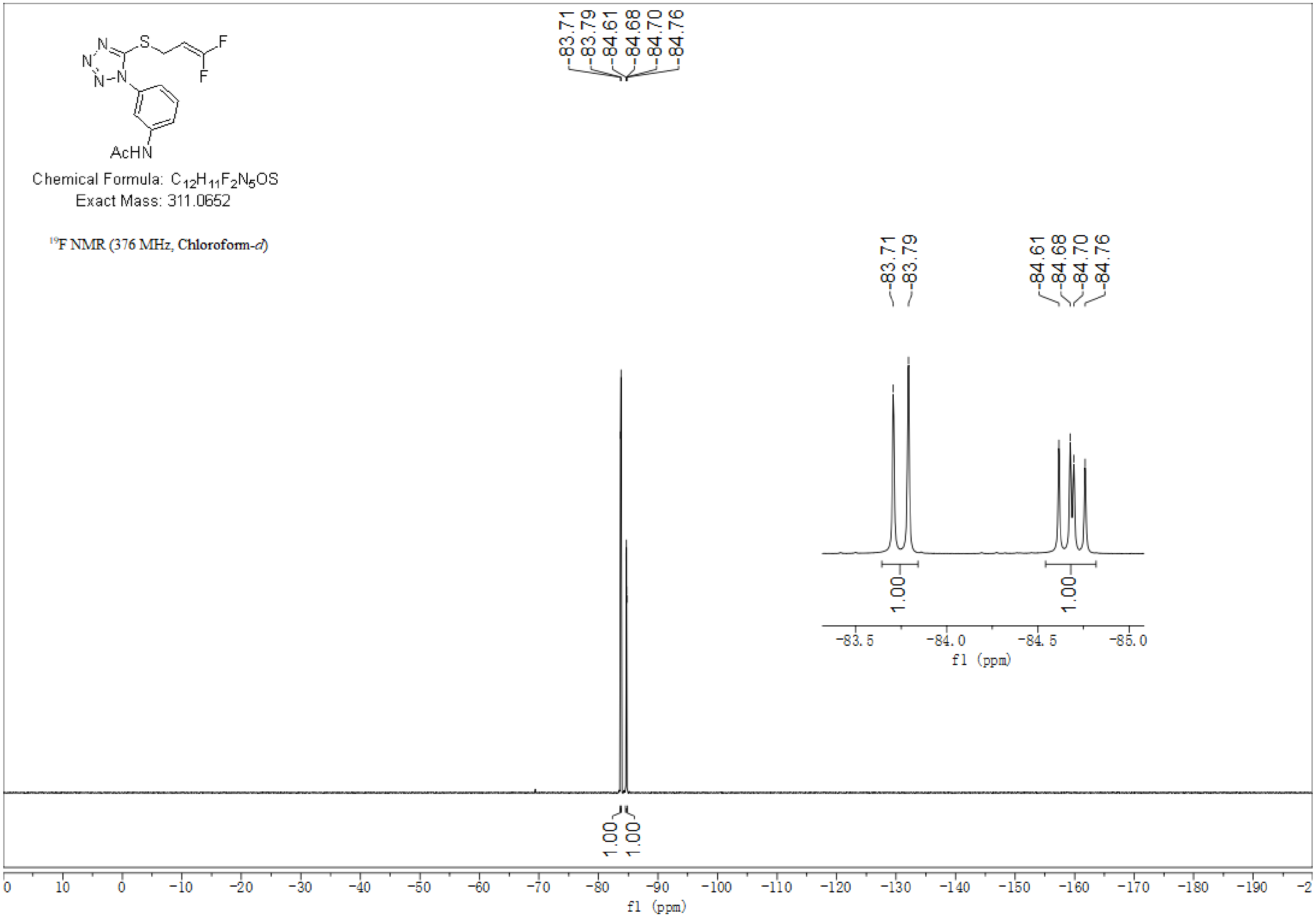

^{13}C NMR spectrum of **3w** (126 MHz, $CDCl_3$)

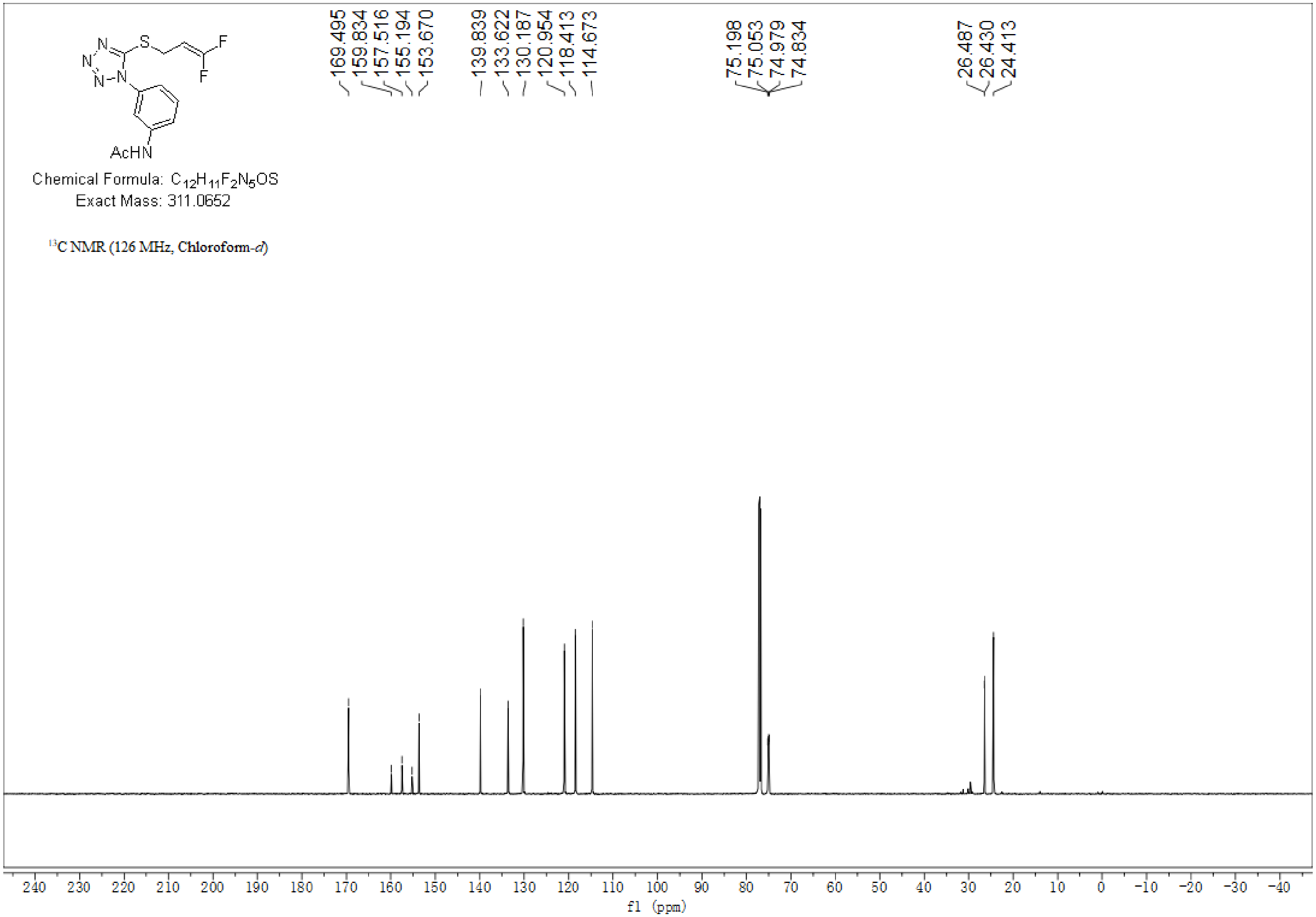

^{1}H NMR spectrum of **3x** (500 MHz, $CDCl_3$)

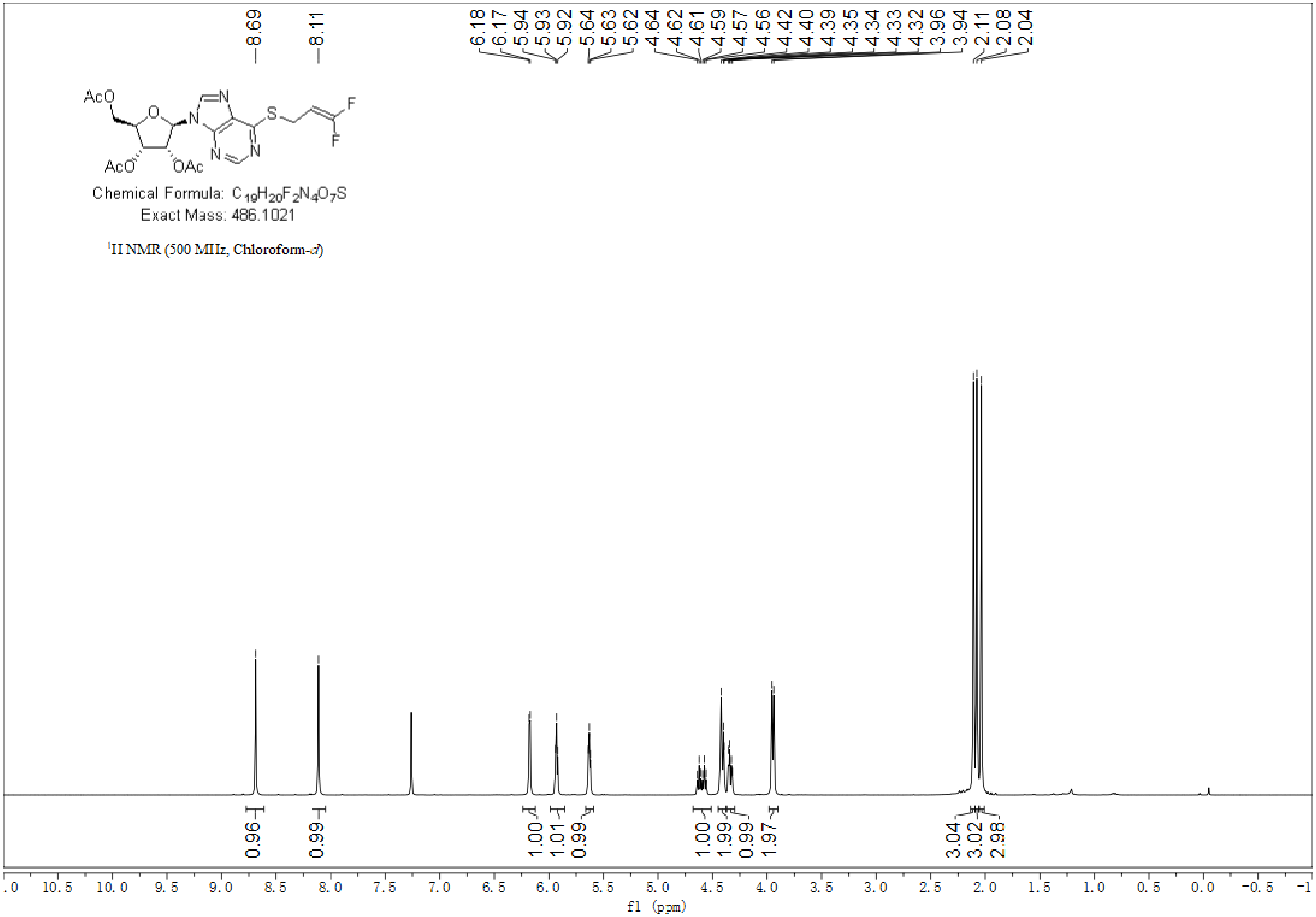

^{19}F NMR spectrum of **3x** (376 MHz, $CDCl_3$)

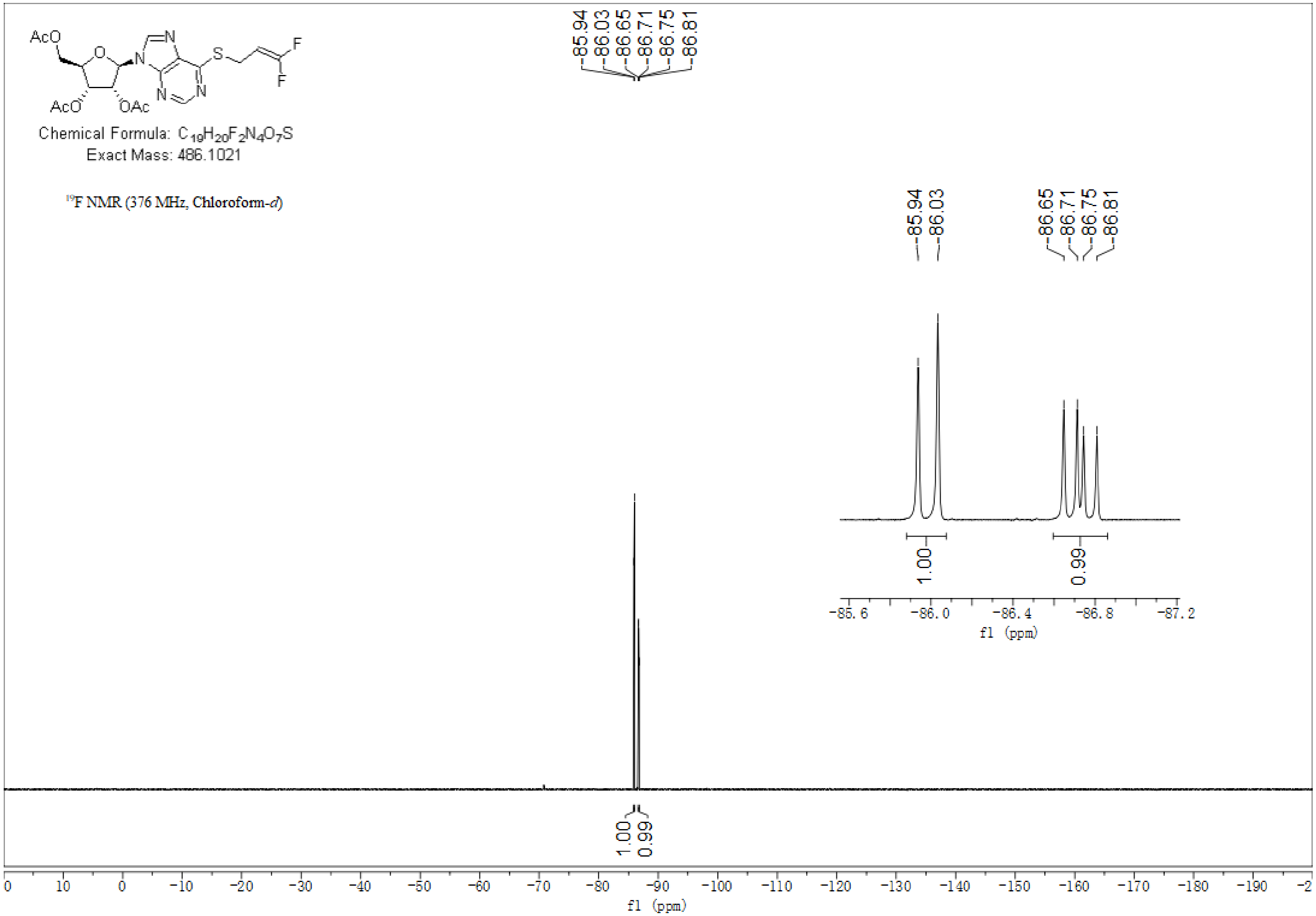

^{13}C NMR spectrum of **3x** (126 MHz, $CDCl_3$)

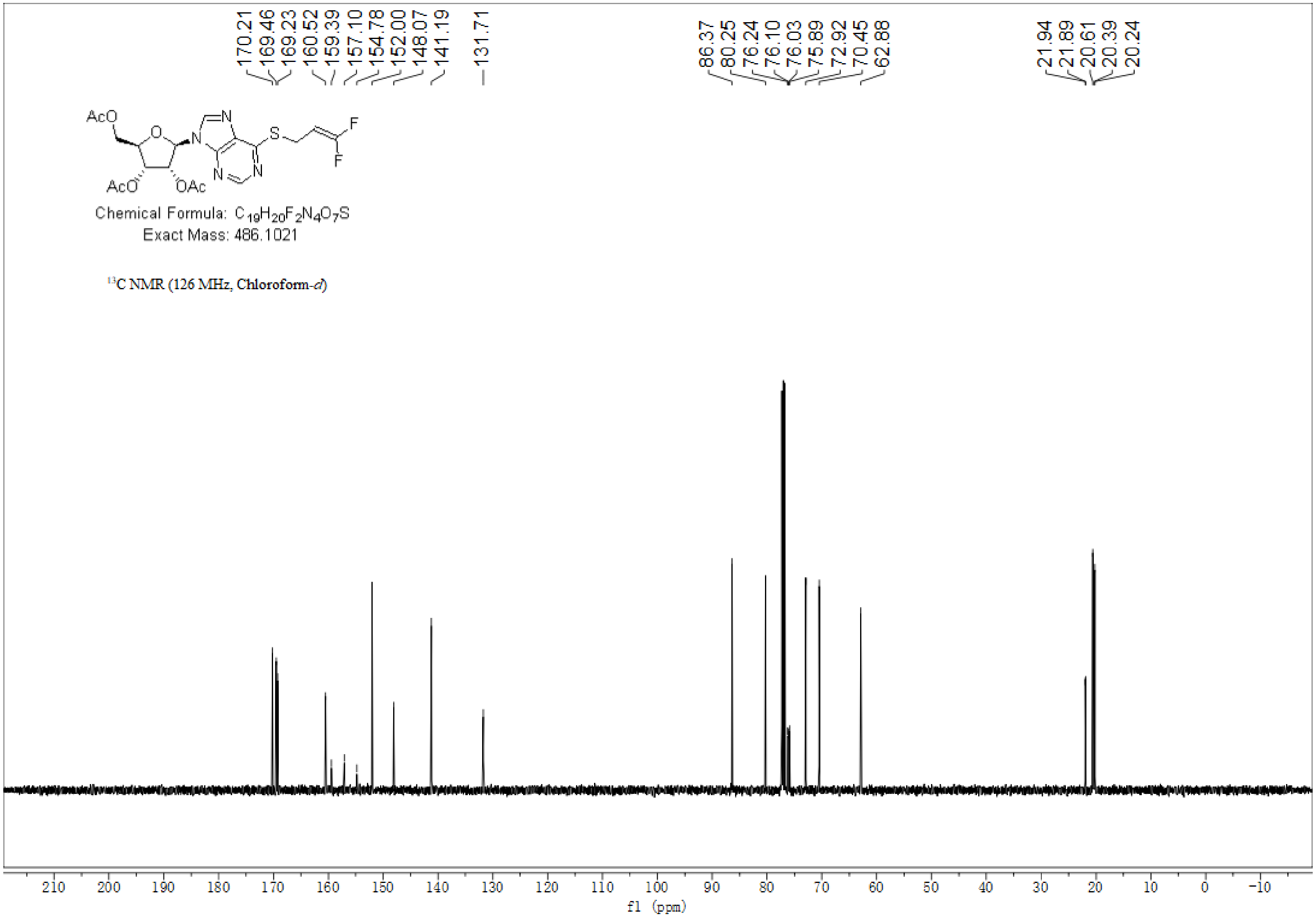

^{1}H NMR spectrum of **5a** (400 MHz, CD_3OD)

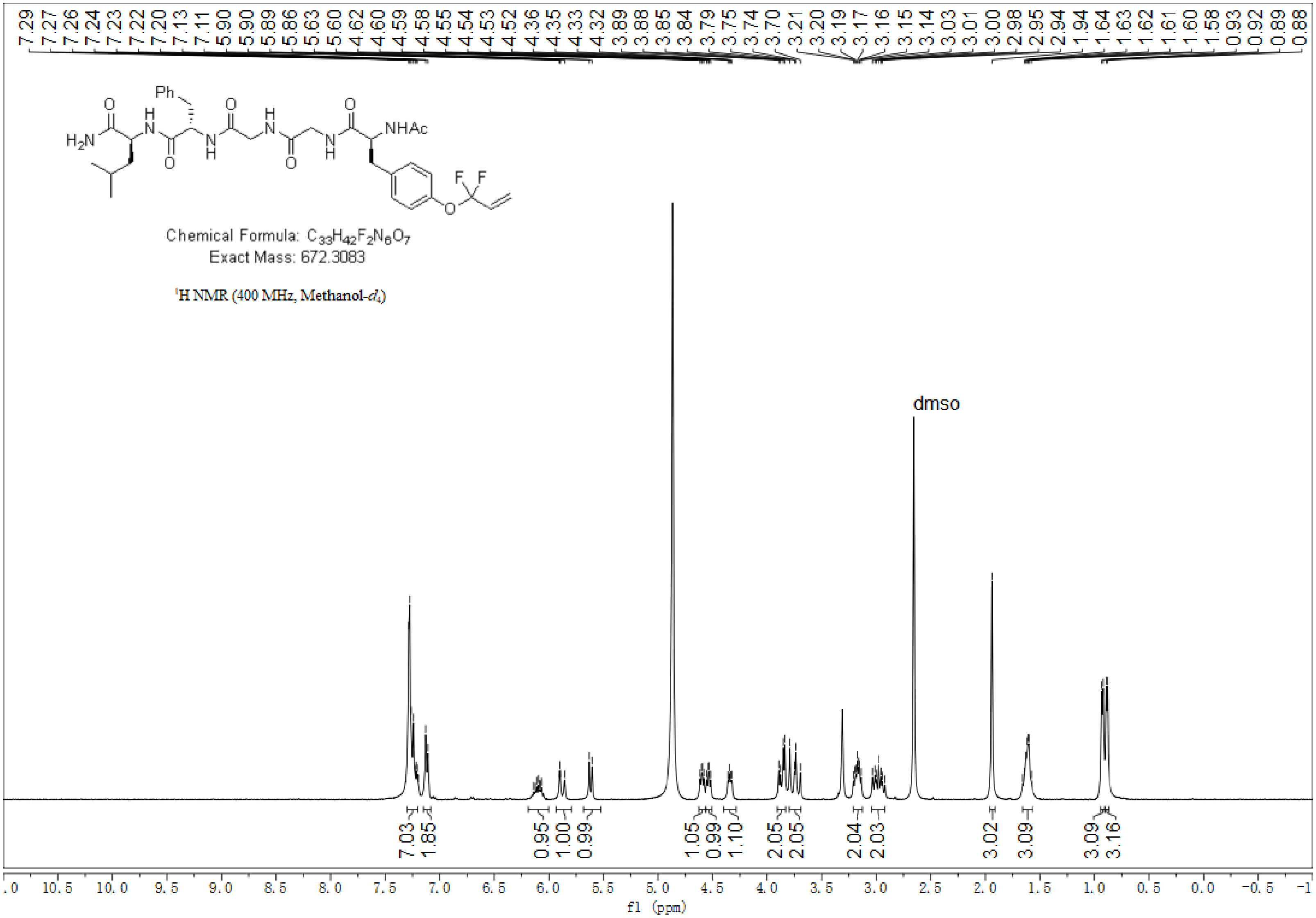

^{19}F NMR spectrum of **5a** (376 MHz, CD_3OD)

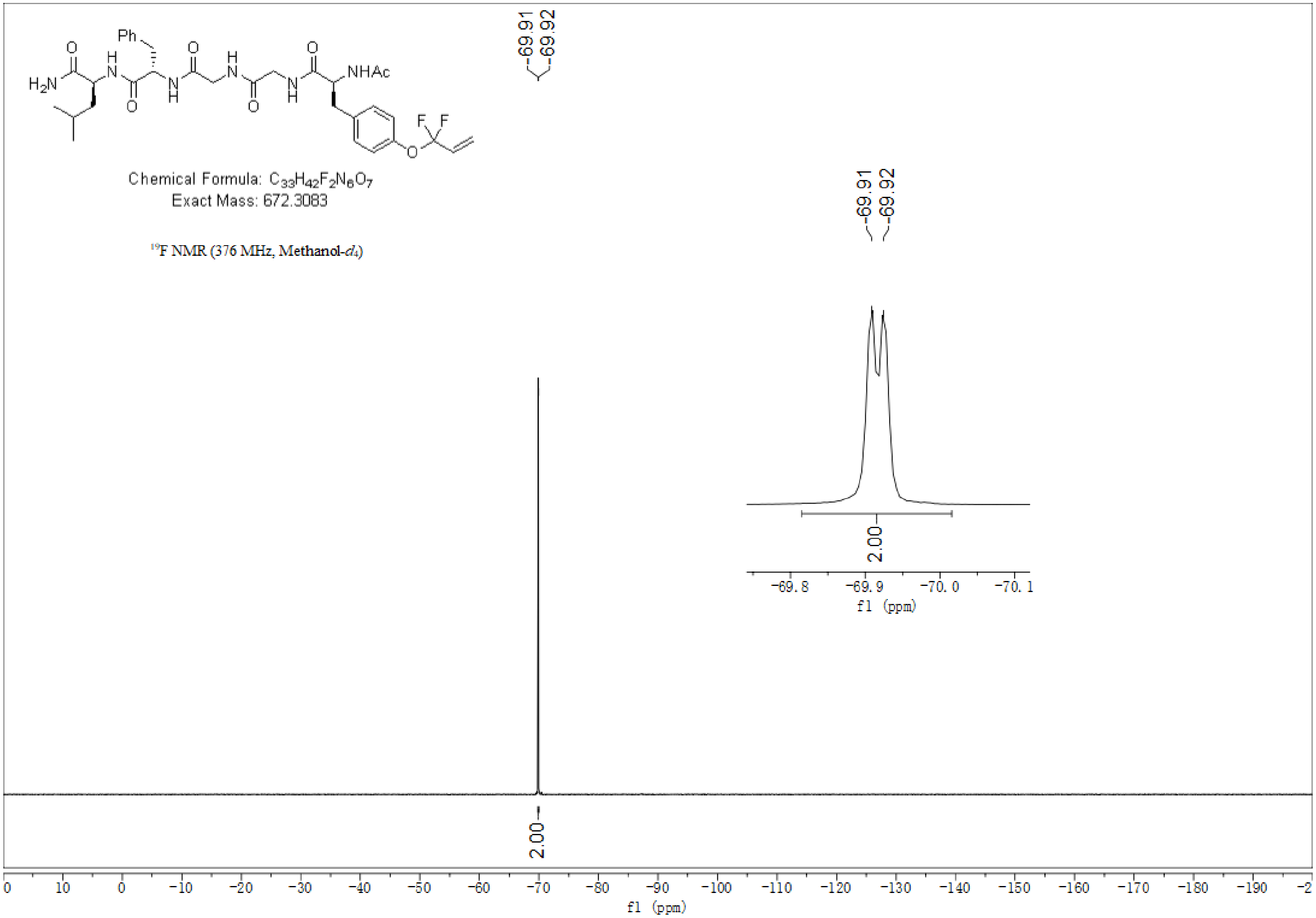

^{13}C NMR spectrum of **5a** (126 MHz, CD_3OD)

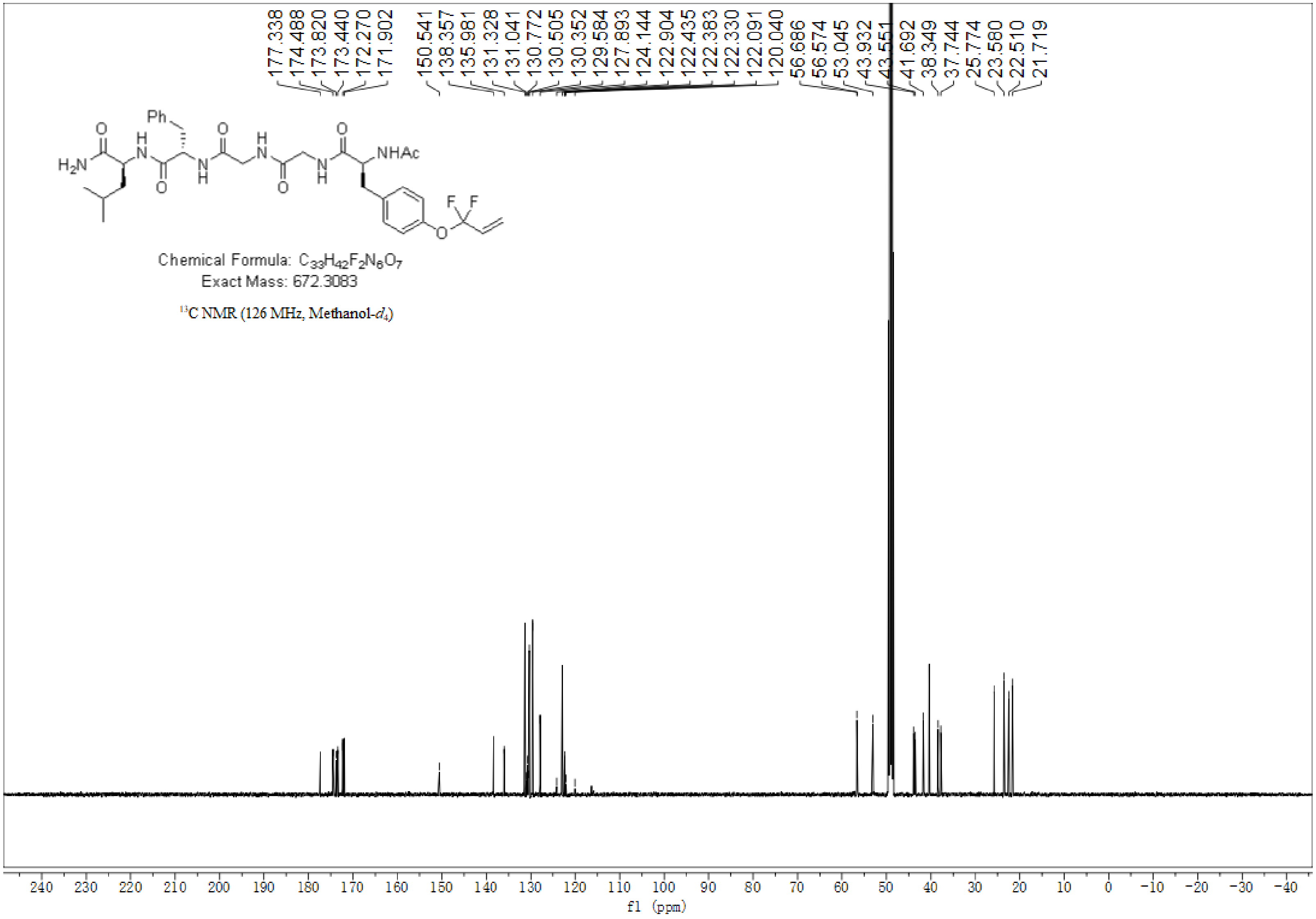

^{1}H NMR spectrum of **5b** (400 MHz, DMSO-d6)

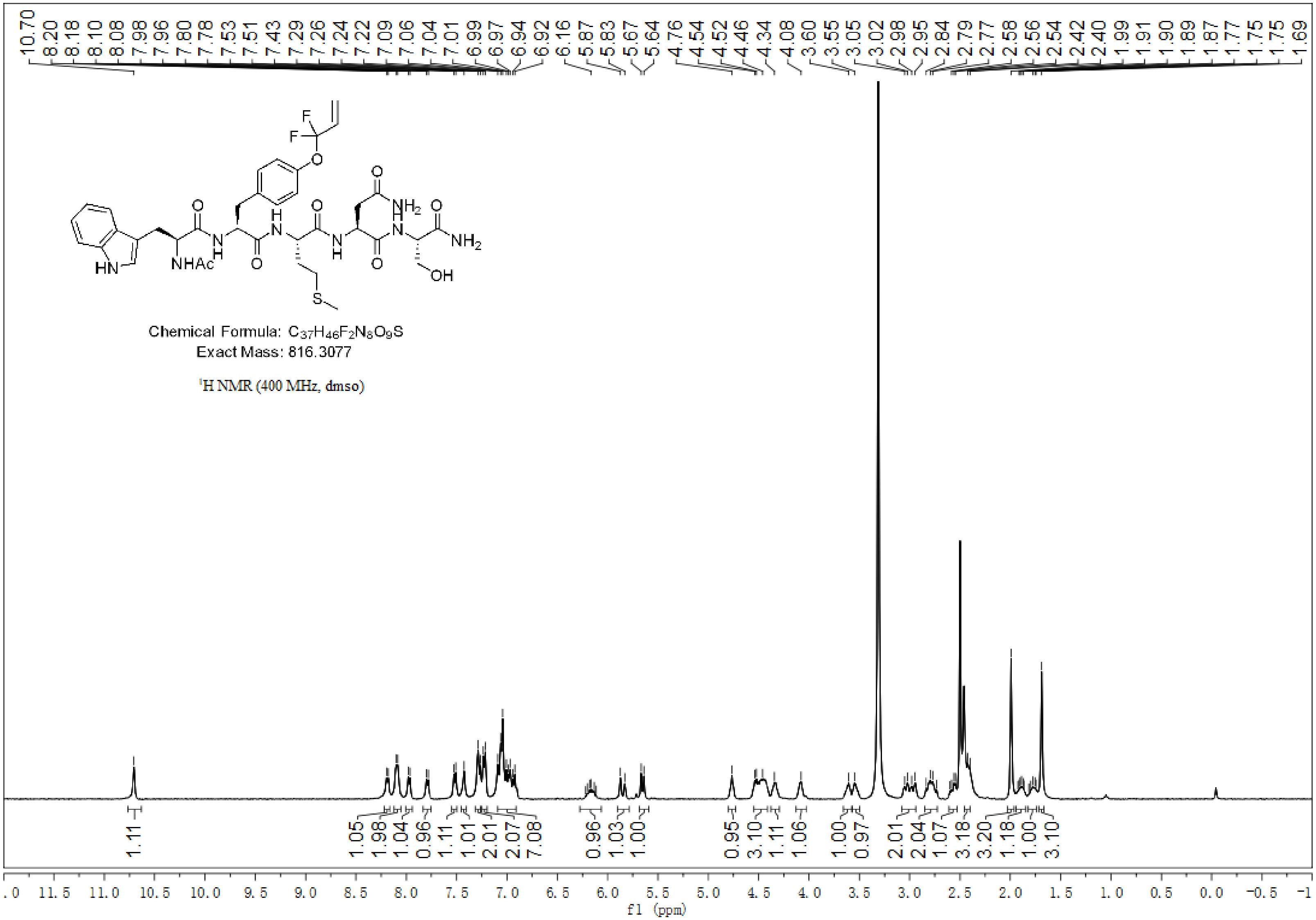

^{19}F NMR spectrum of **5b** (376 MHz, DMSO-d6)

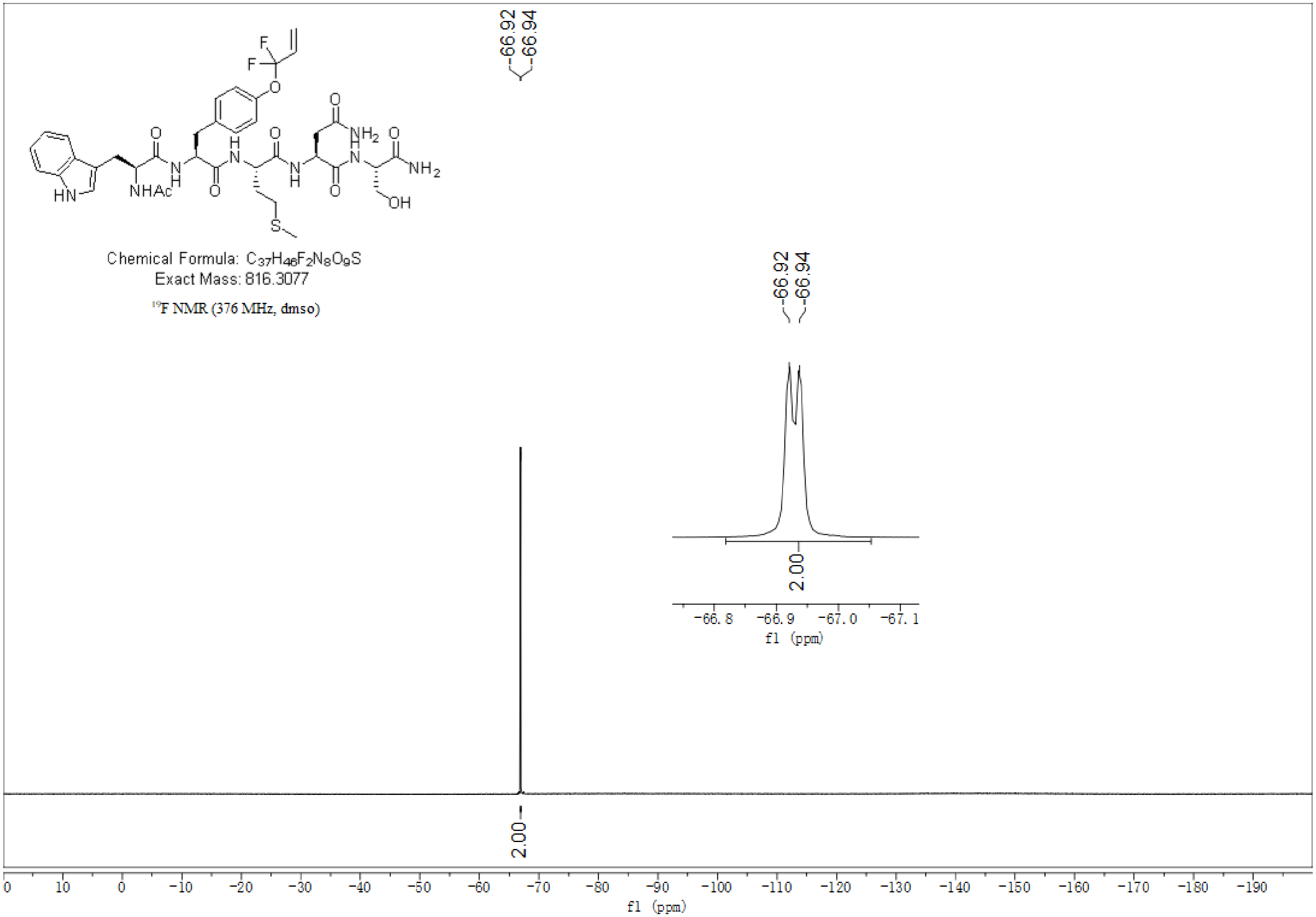

^{13}C NMR spectrum of **5b** (126 MHz, DMSO-d6)

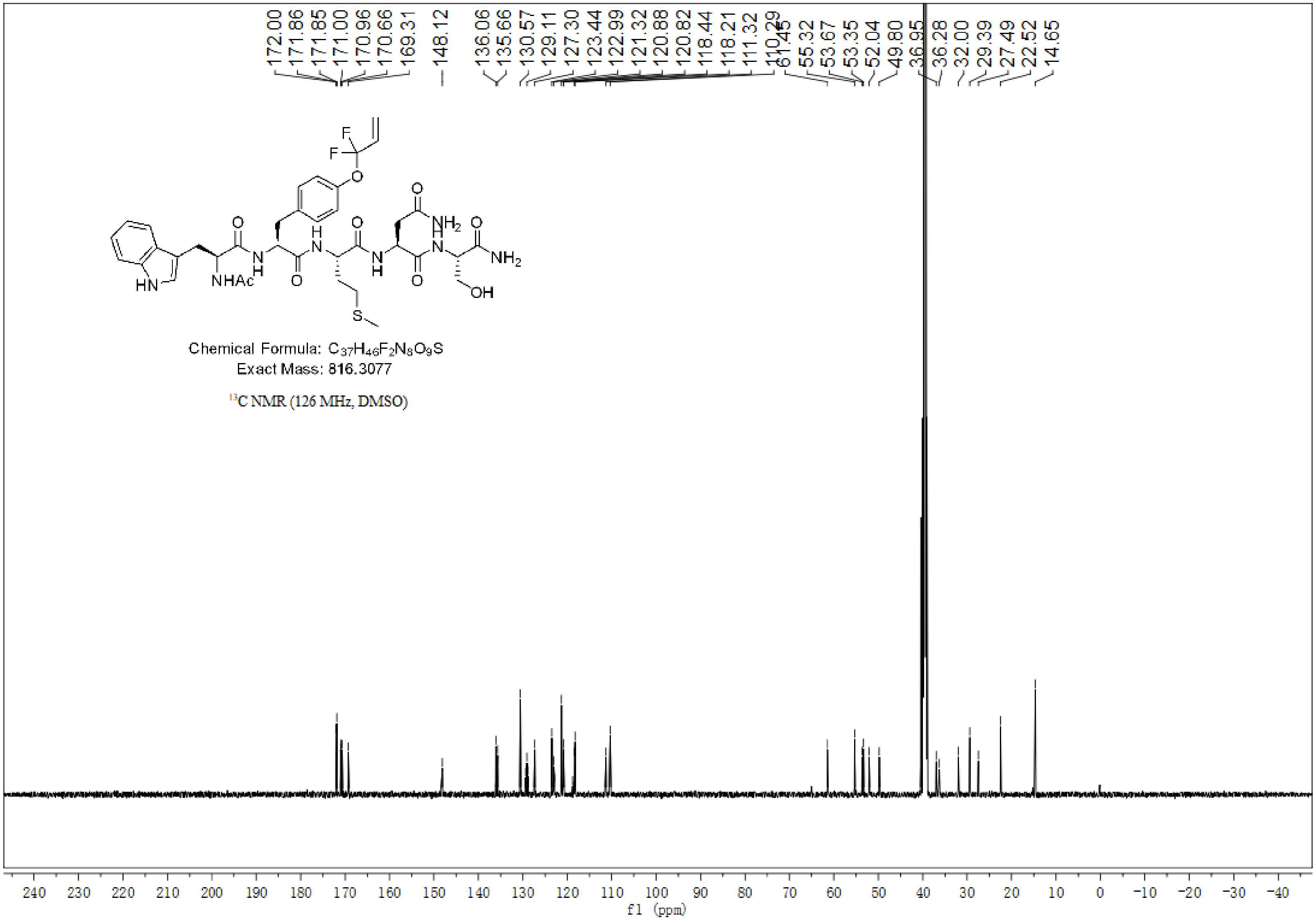

^{1}H NMR spectrum of **5c** (600 MHz, DMSO-d6)

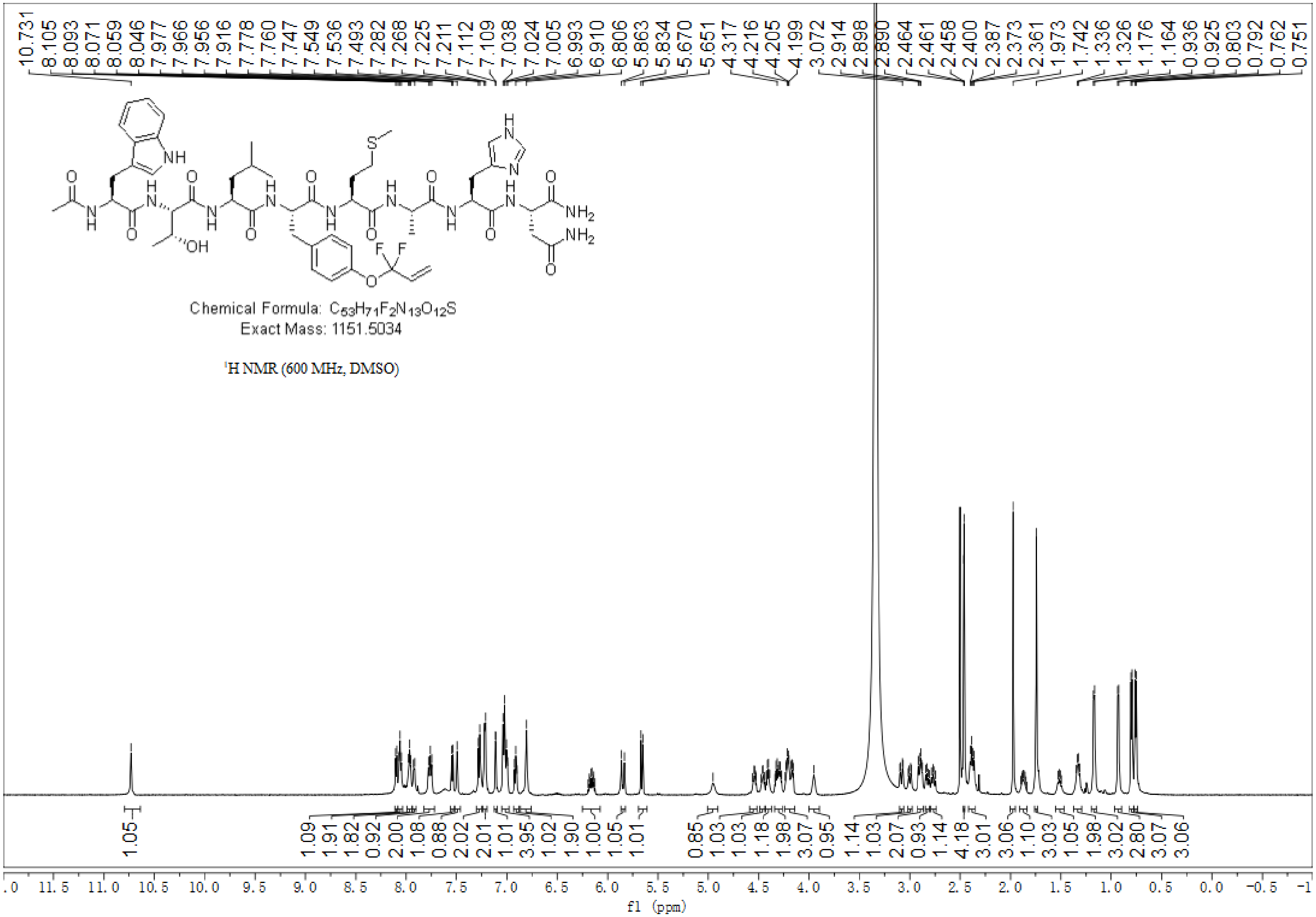

^{19}F NMR spectrum of **5c** (376 MHz, DMSO-d6)

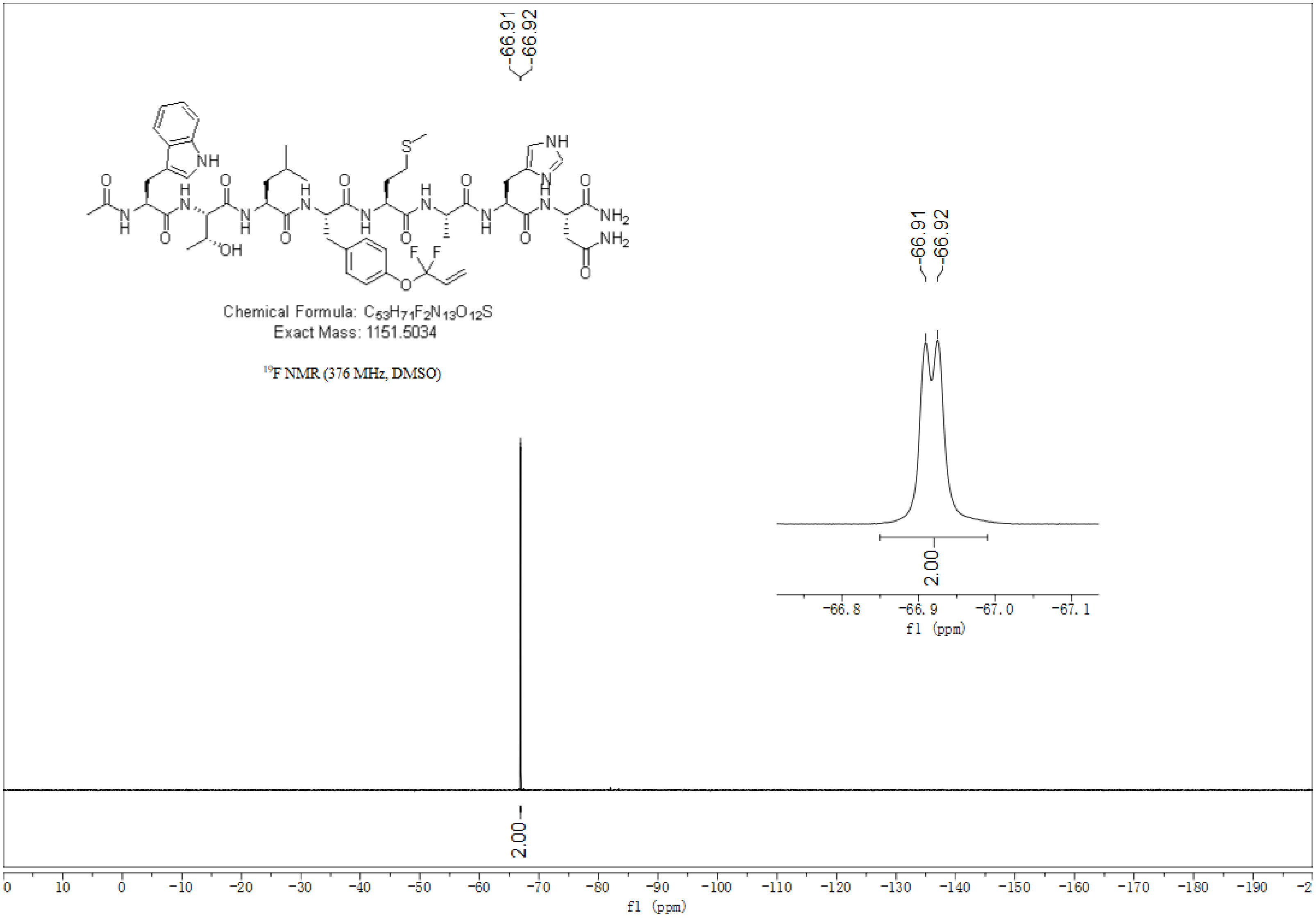

^{13}C NMR spectrum of **5c** (151 MHz, DMSO-d6)

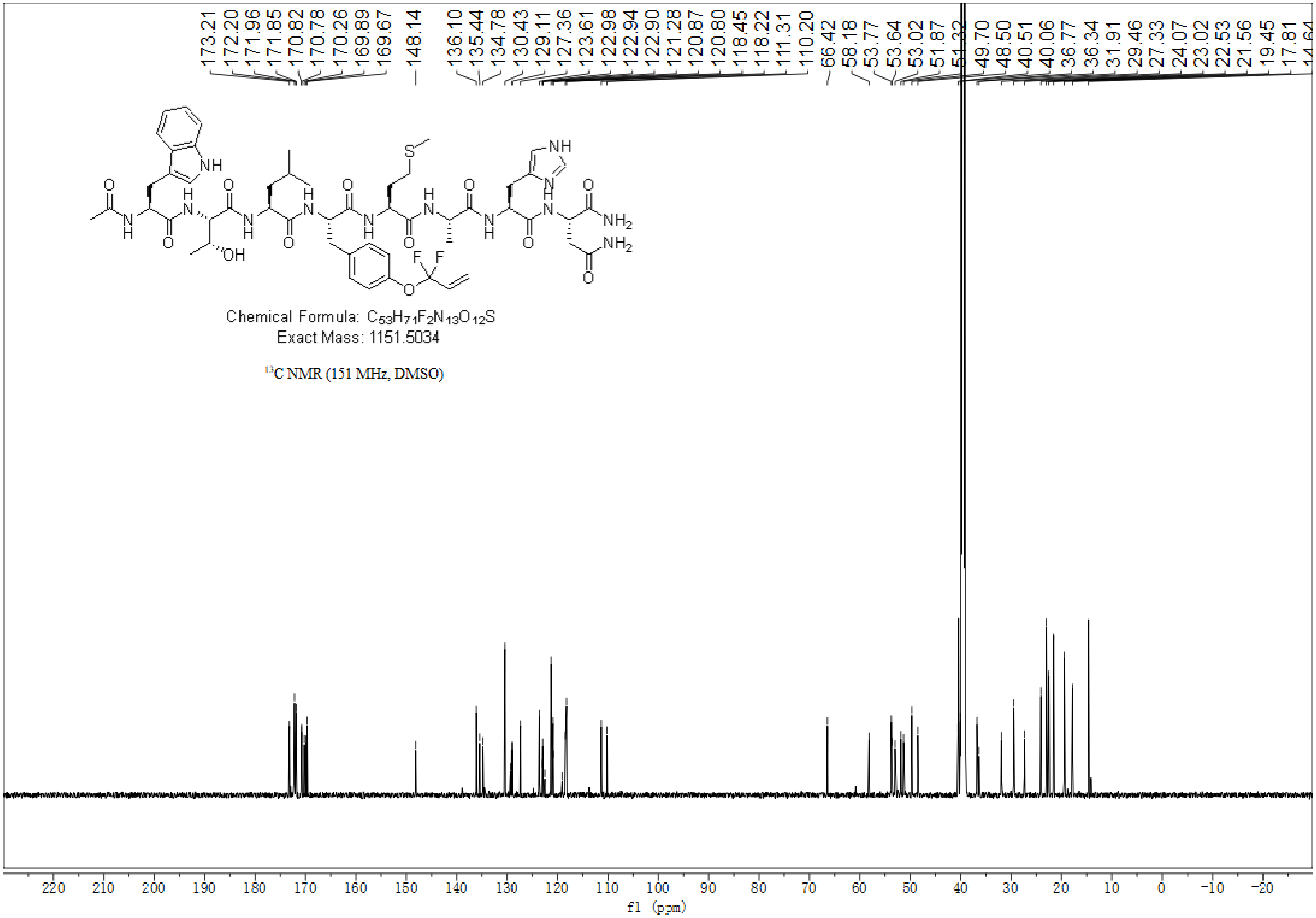

^{1}H NMR spectrum of **5d** (400 MHz, CD_3OD)

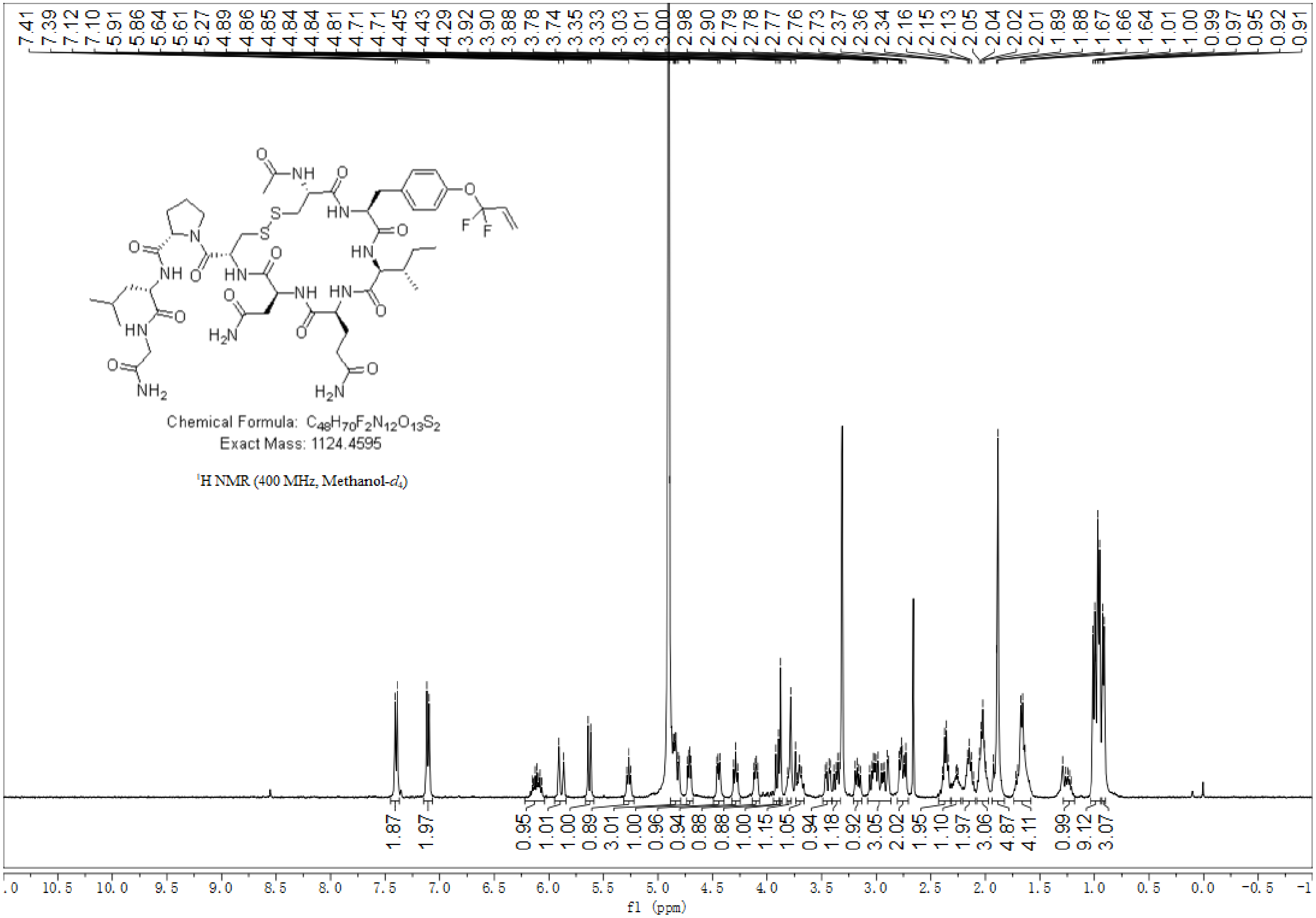

^{19}F NMR spectrum of **5d** (376 MHz, CD_3OD)

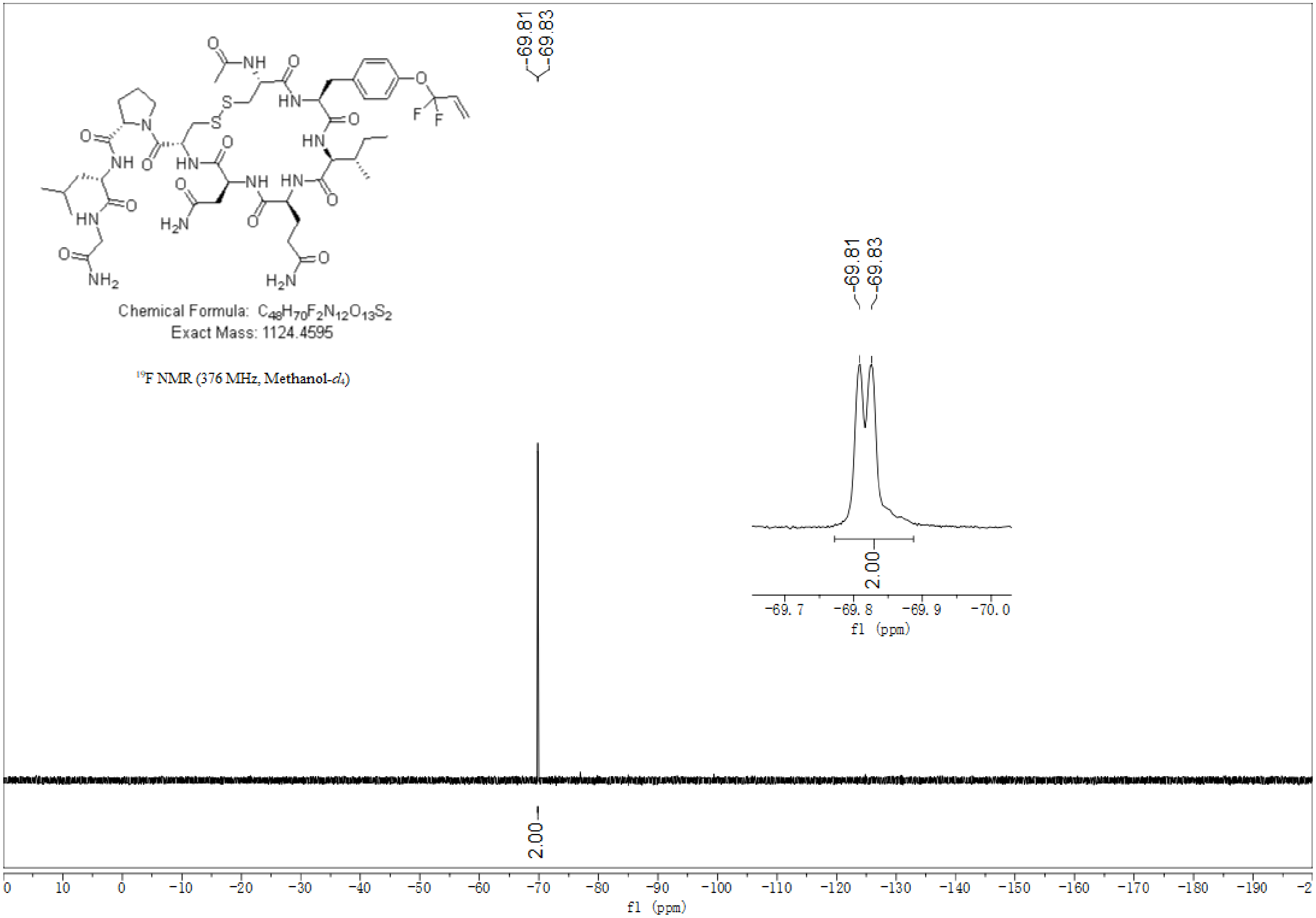

^{13}C NMR spectrum of **5d** (126 MHz, CD_3OD)

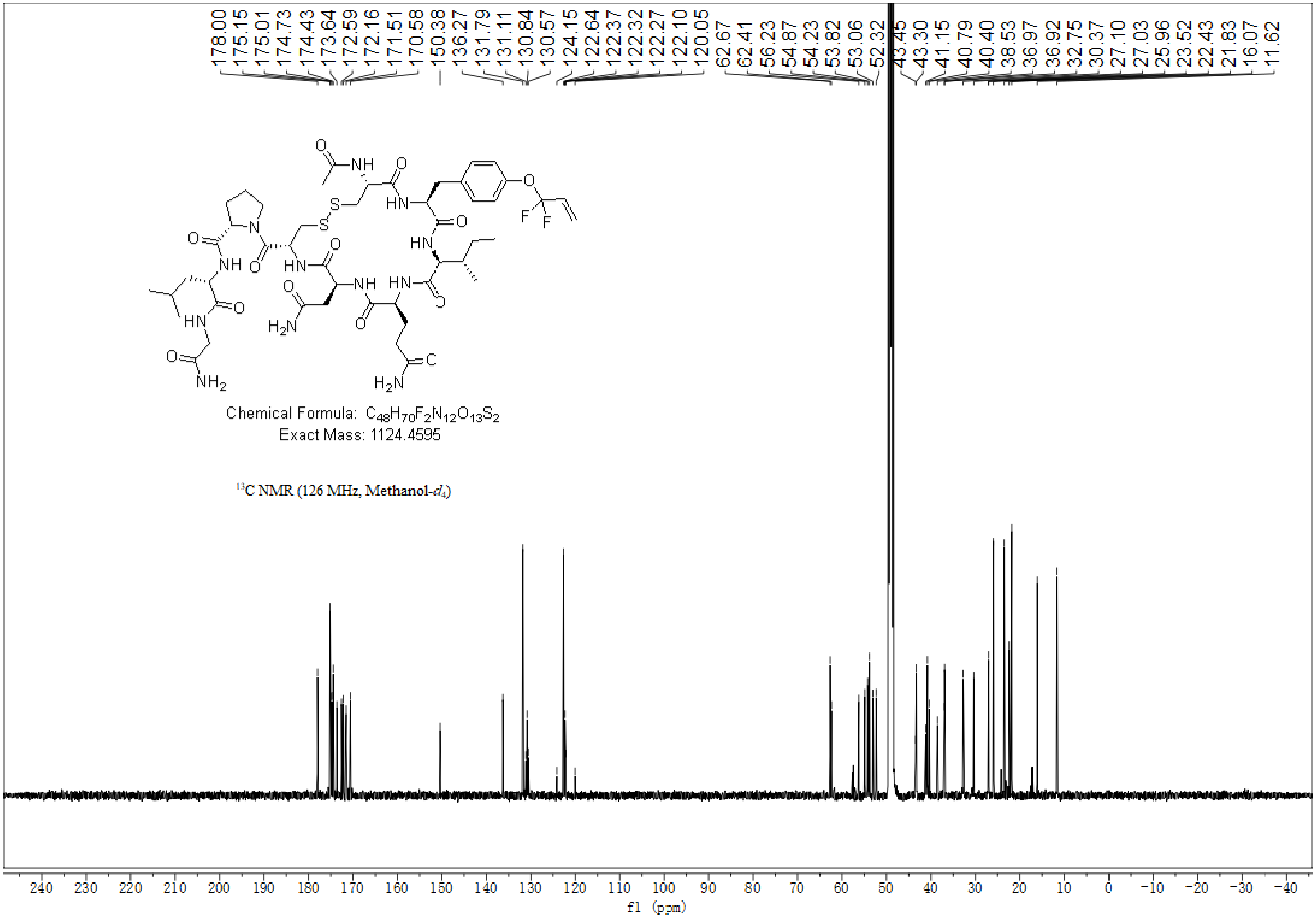

^{1}H NMR spectrum of **5e** (600 MHz, DMSO-d6)

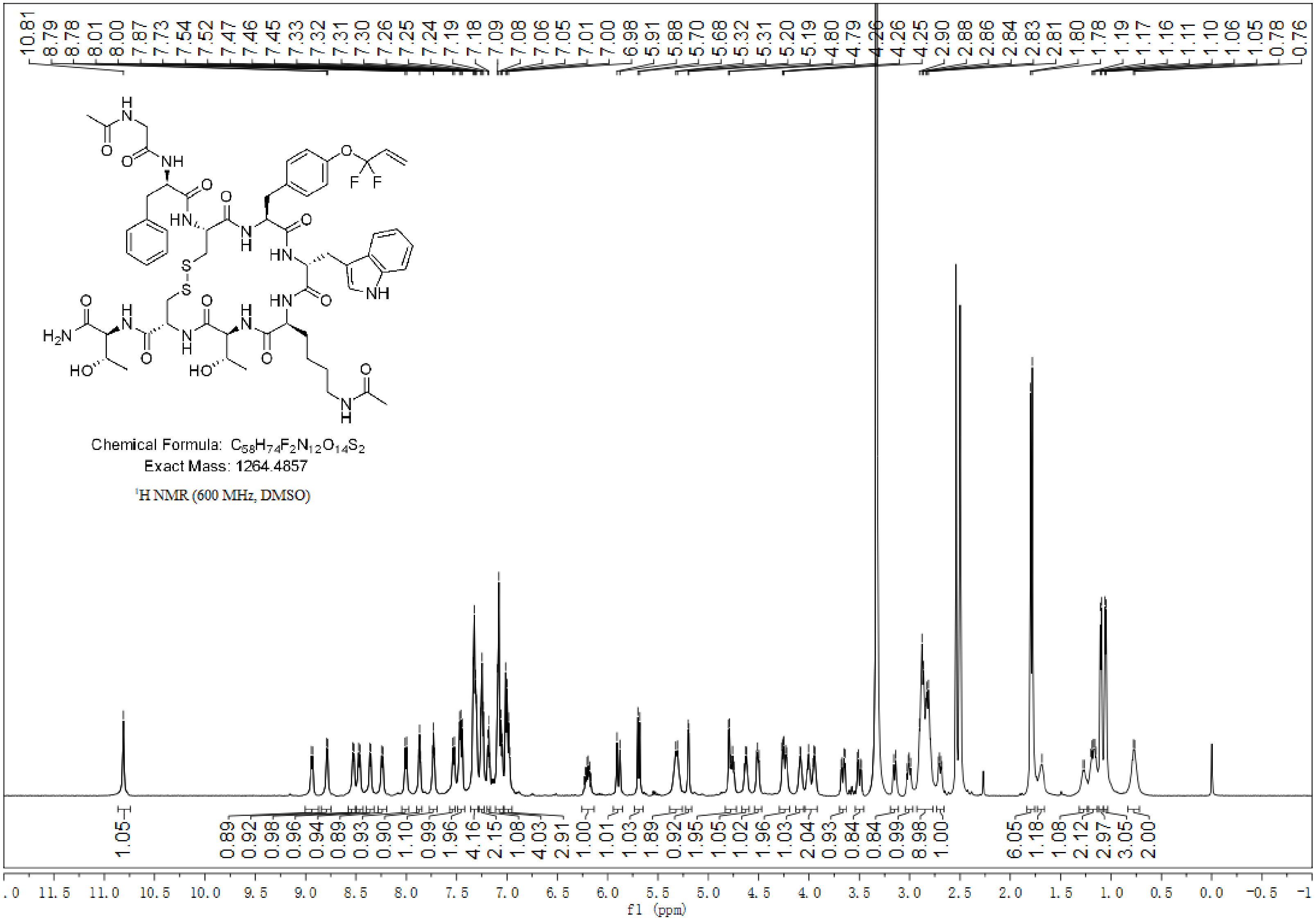

^{19}F NMR spectrum of **5e** (565 MHz, DMSO-d6)

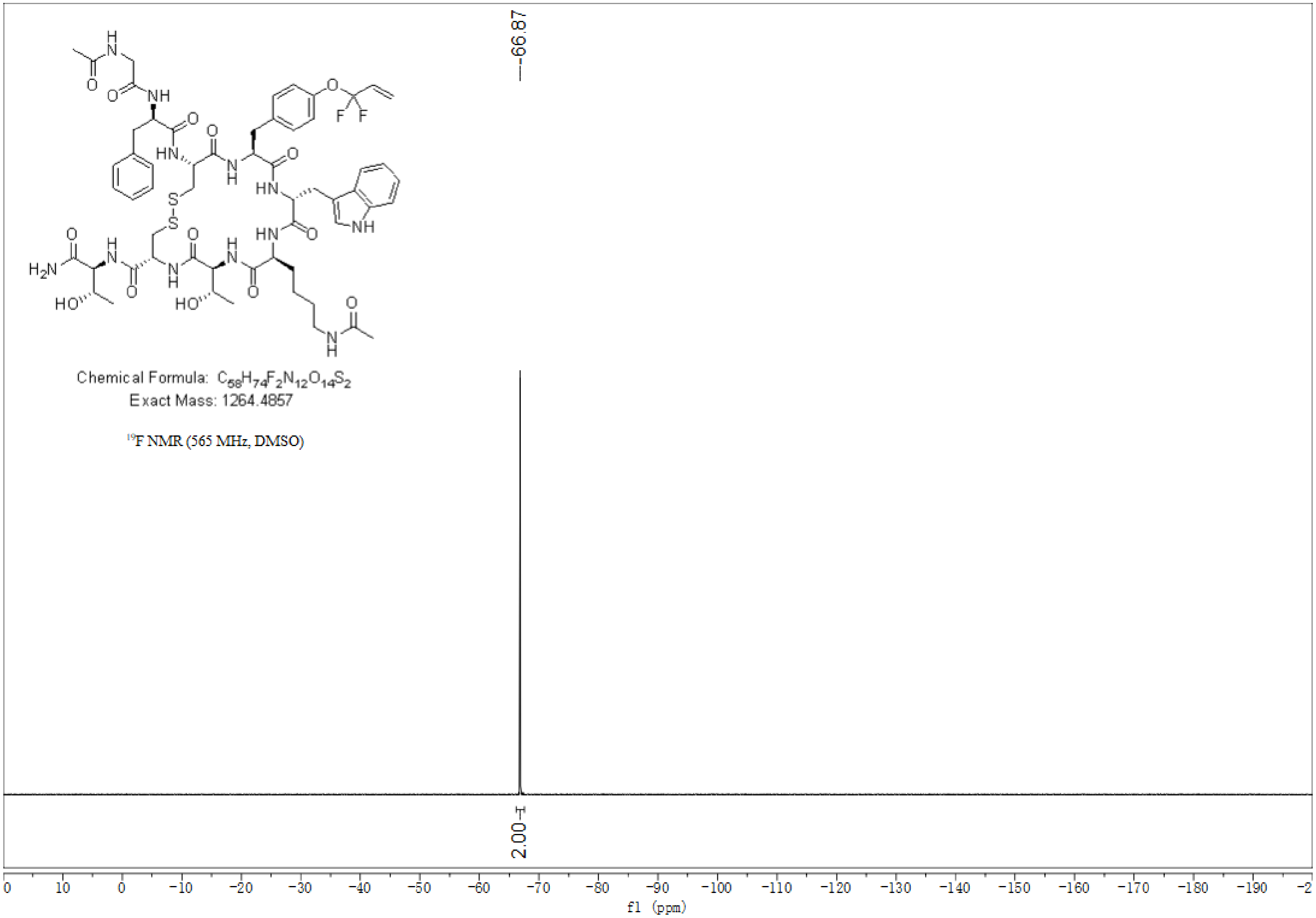

^{13}C NMR spectrum of **5e** (151 MHz, DMSO-d6)

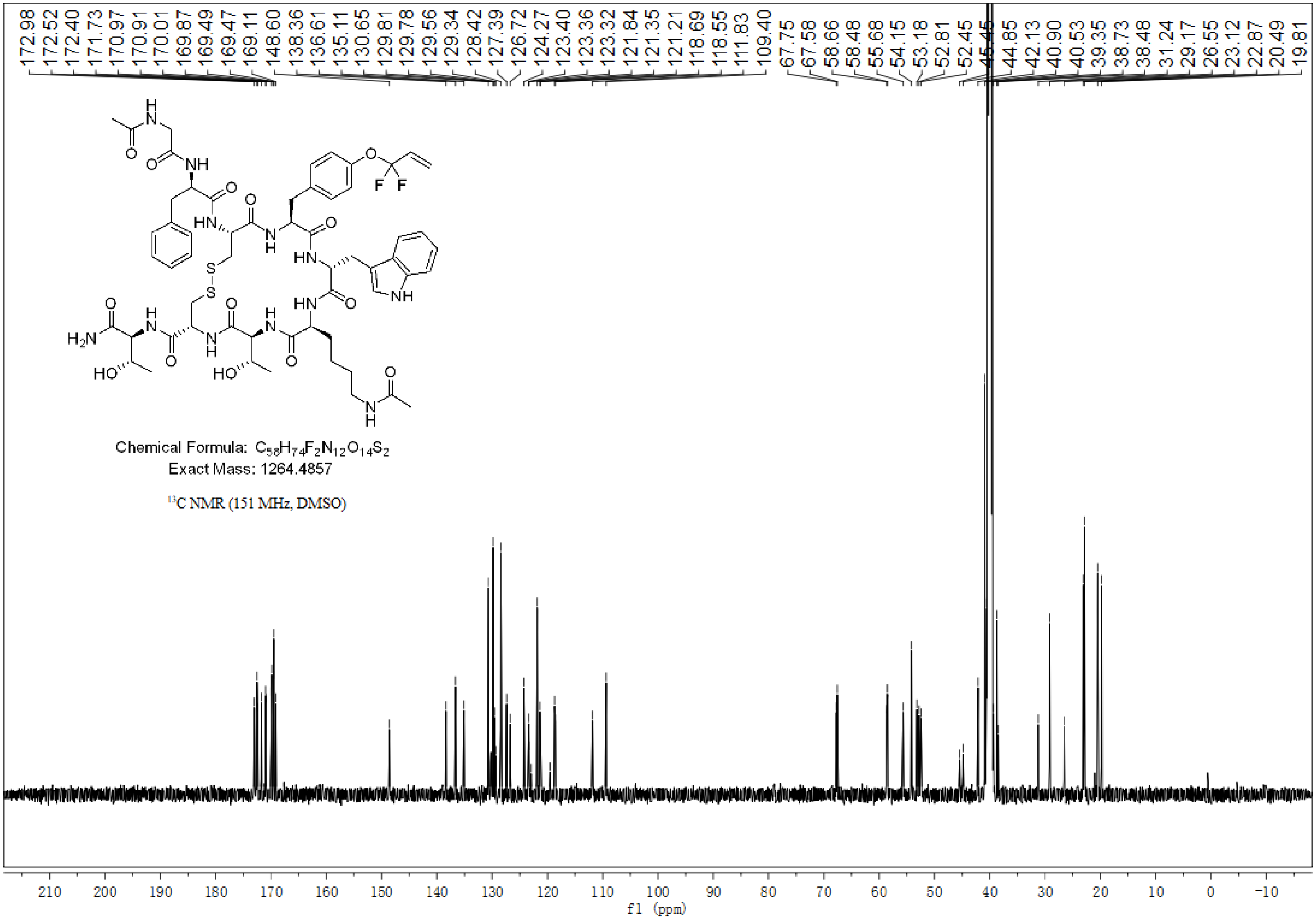

^{1}H NMR spectrum of **5f** (500 MHz, DMSO-d6)

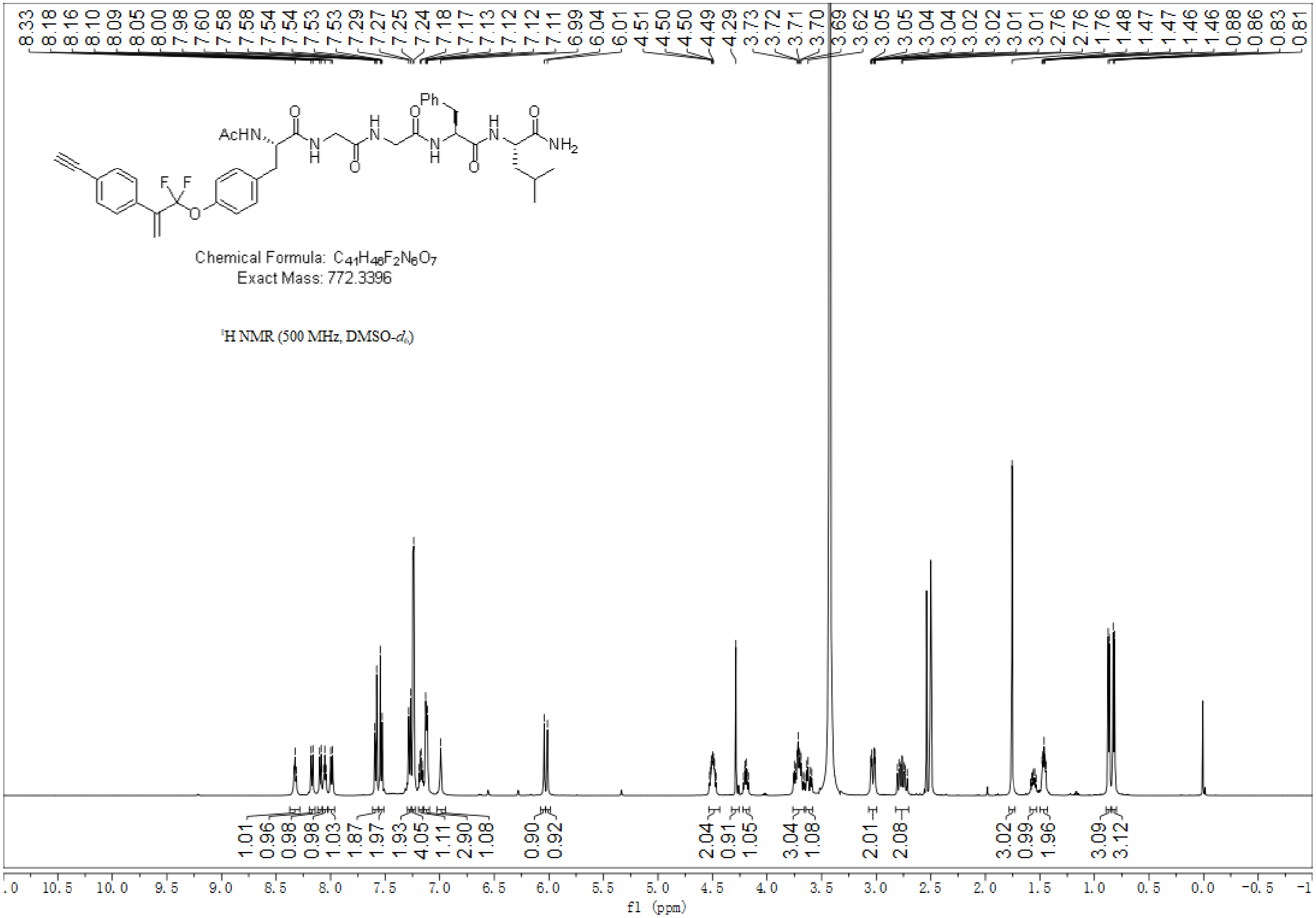

^{19}F NMR spectrum of **5f** (471 MHz, DMSO-d6)

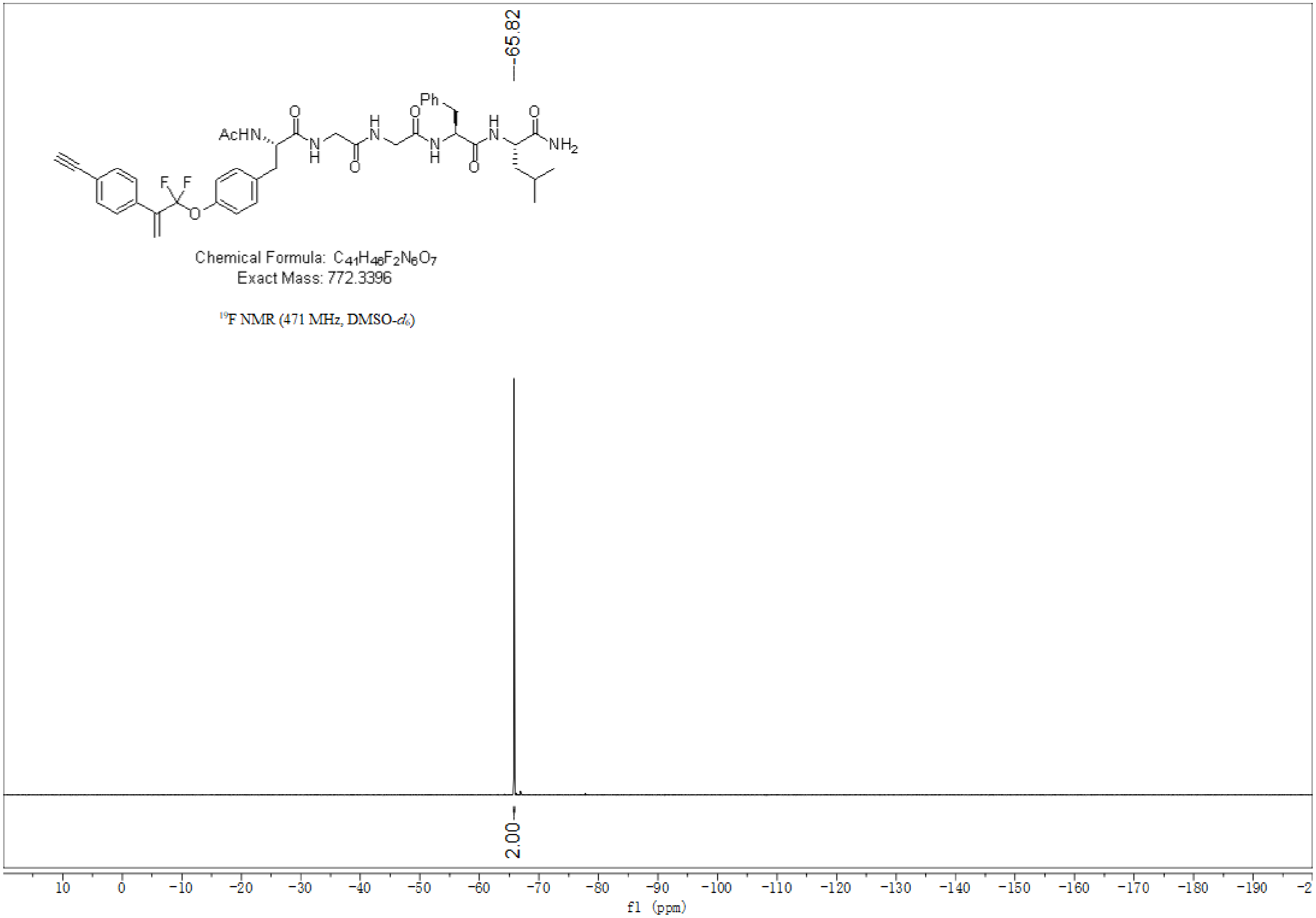

^{13}C NMR spectrum of **5f** (126 MHz, DMSO-d6)

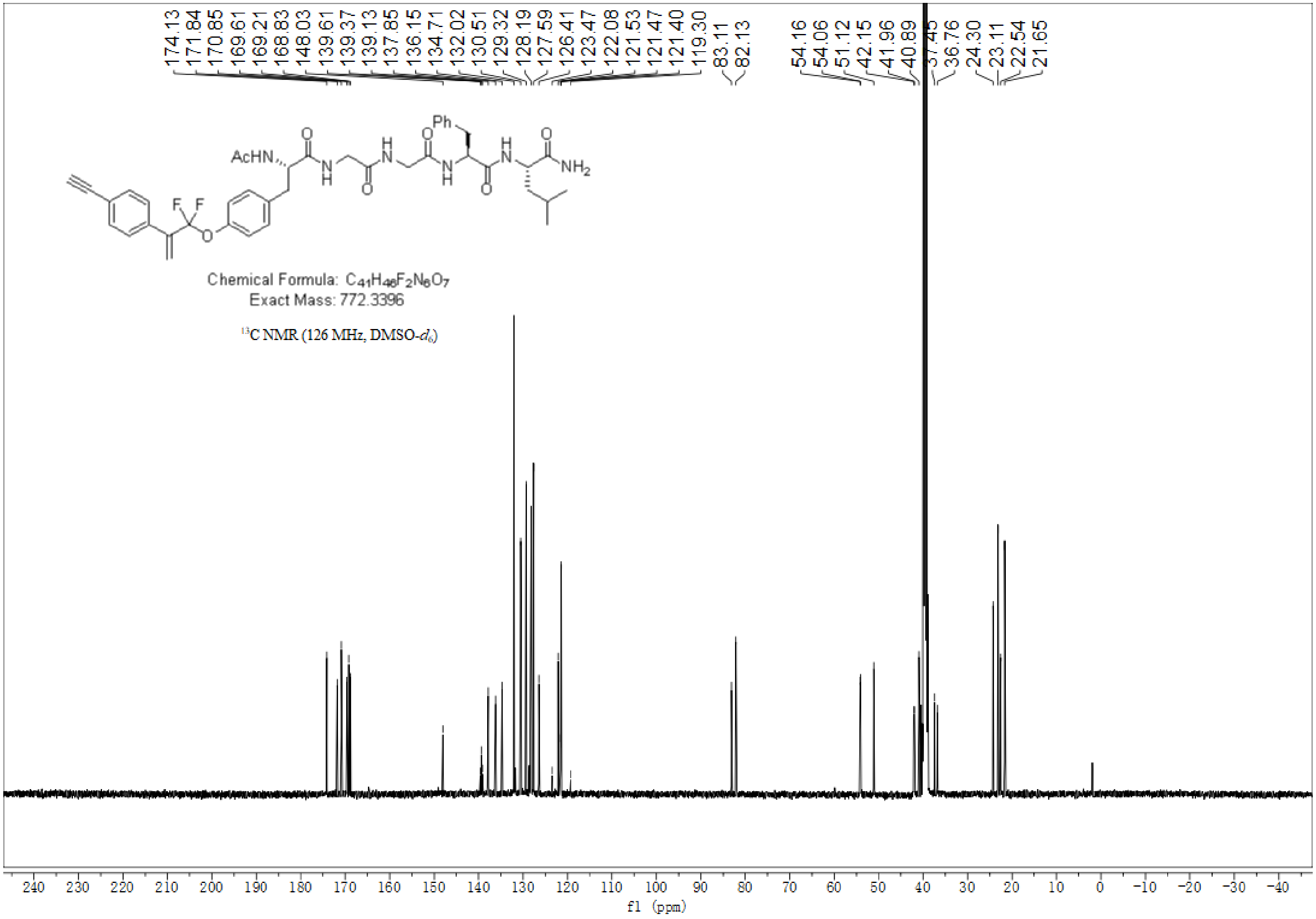

^{1}H NMR spectrum of **8** (400 MHz, CD_3OD)

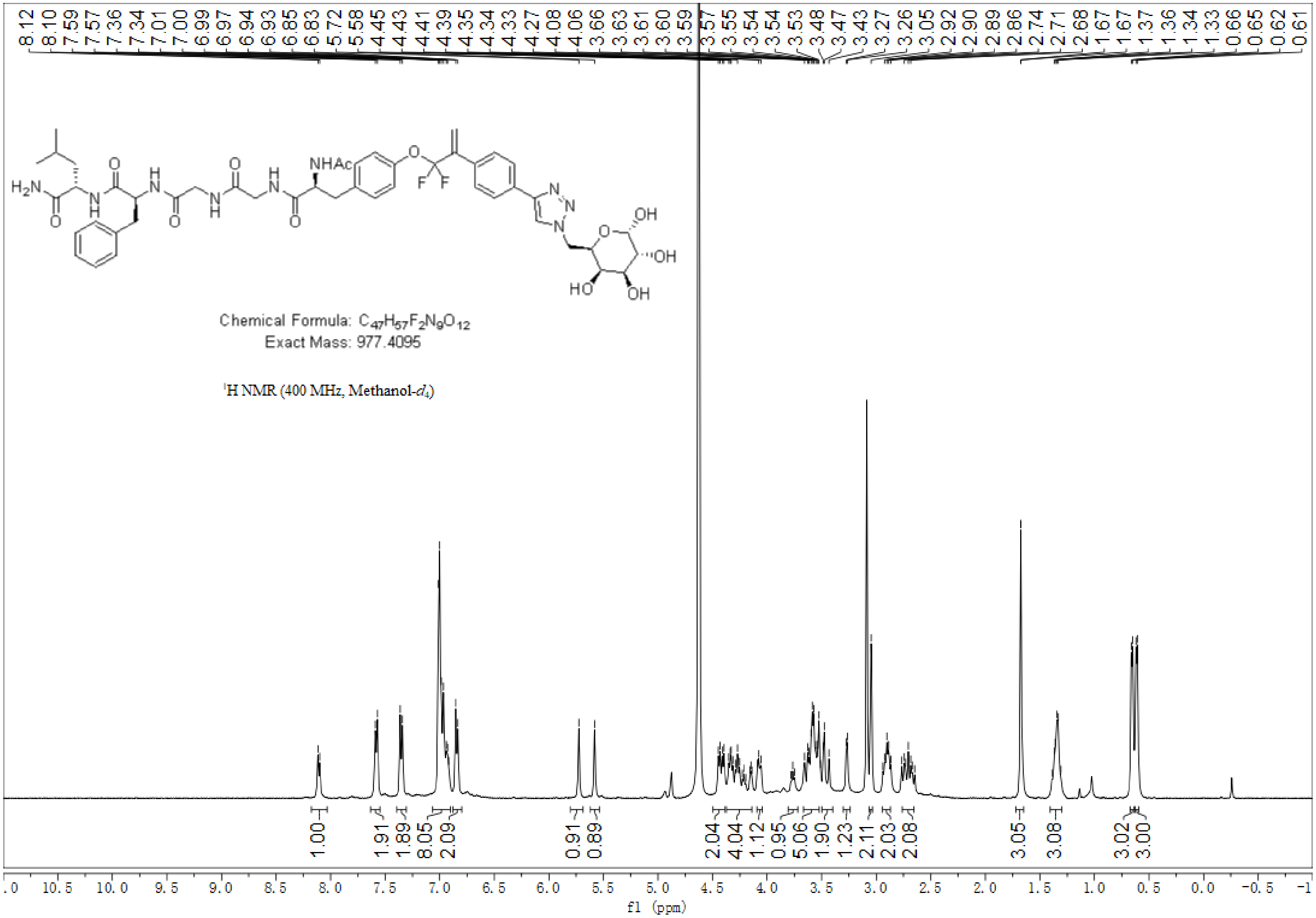

^{19}F NMR spectrum of **8** (376 MHz, CD_3OD)

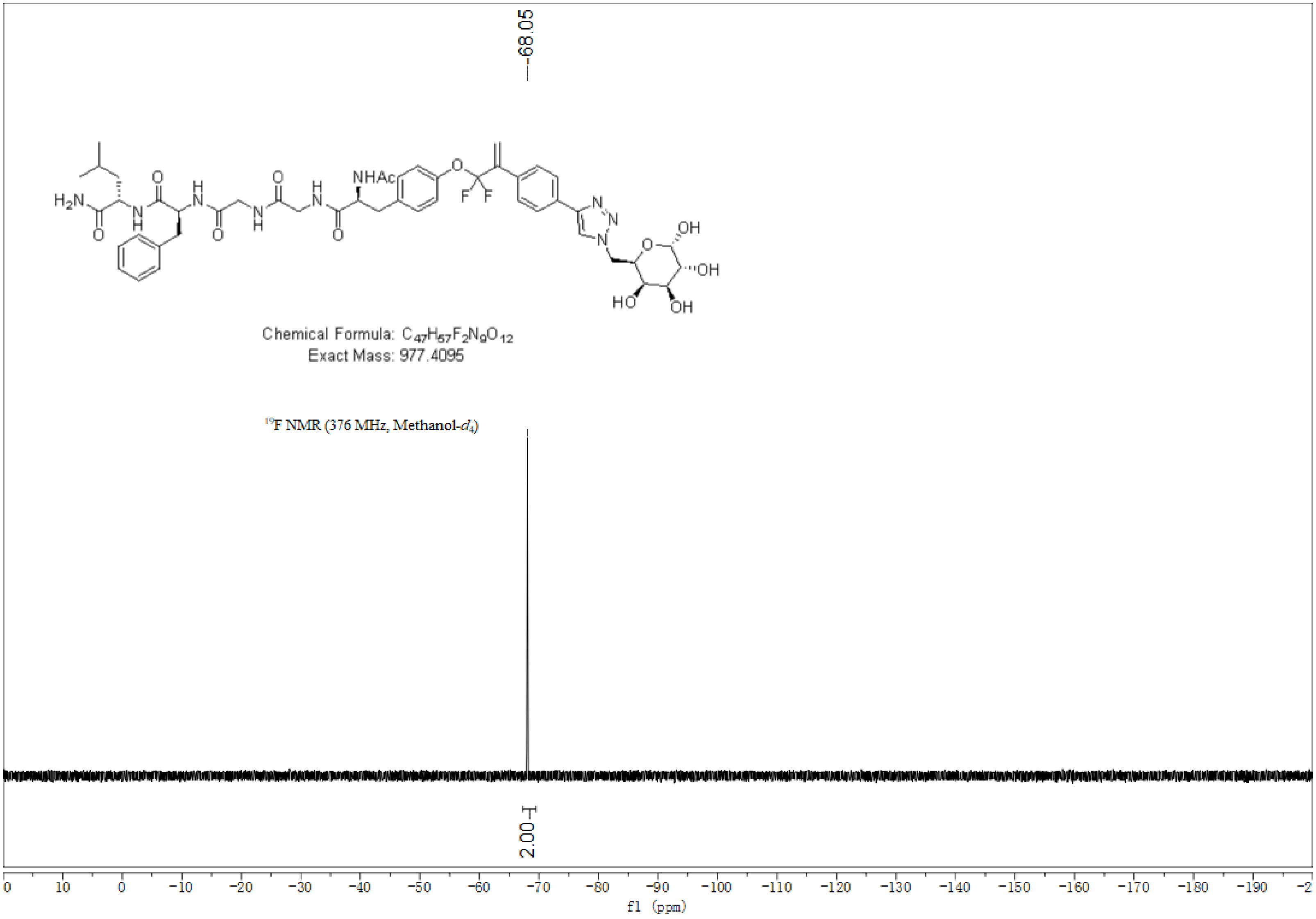

^{13}C NMR spectrum of **8** (126 MHz, CD_3OD)

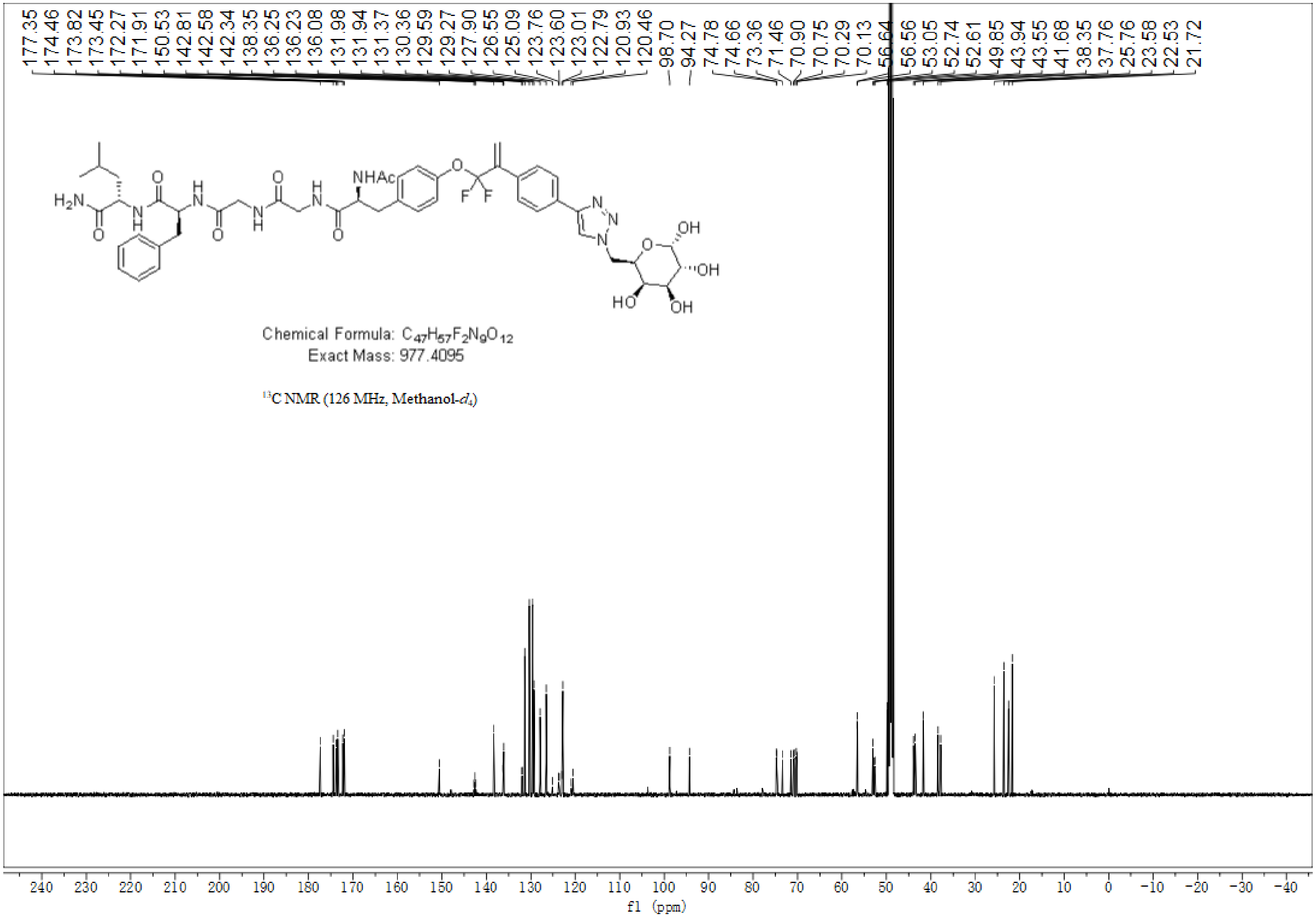

^{1}H NMR spectrum of **9** (500 MHz, $CDCl_3$)

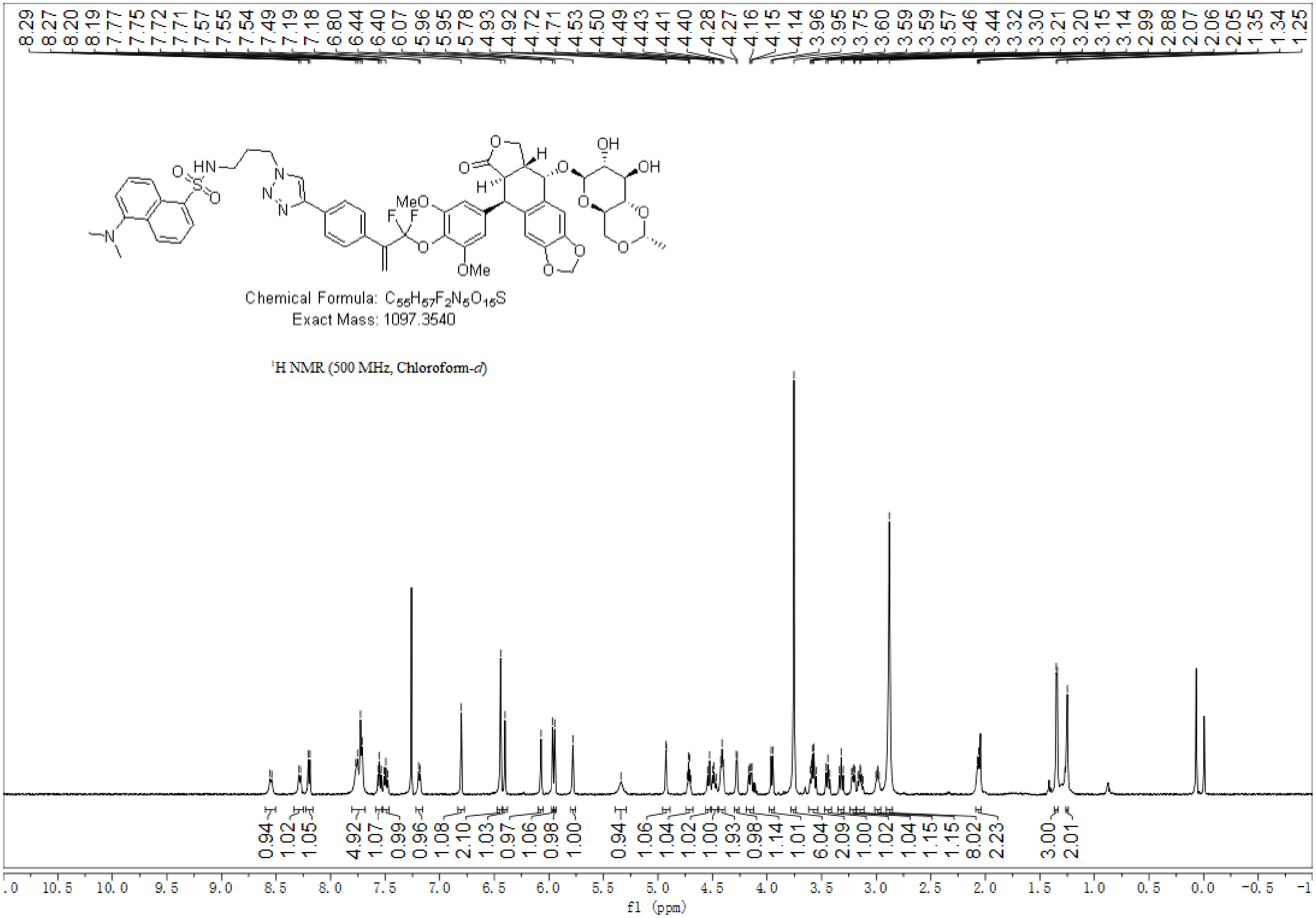

^{19}F NMR spectrum of **9** (376 MHz, $CDCl_3$)

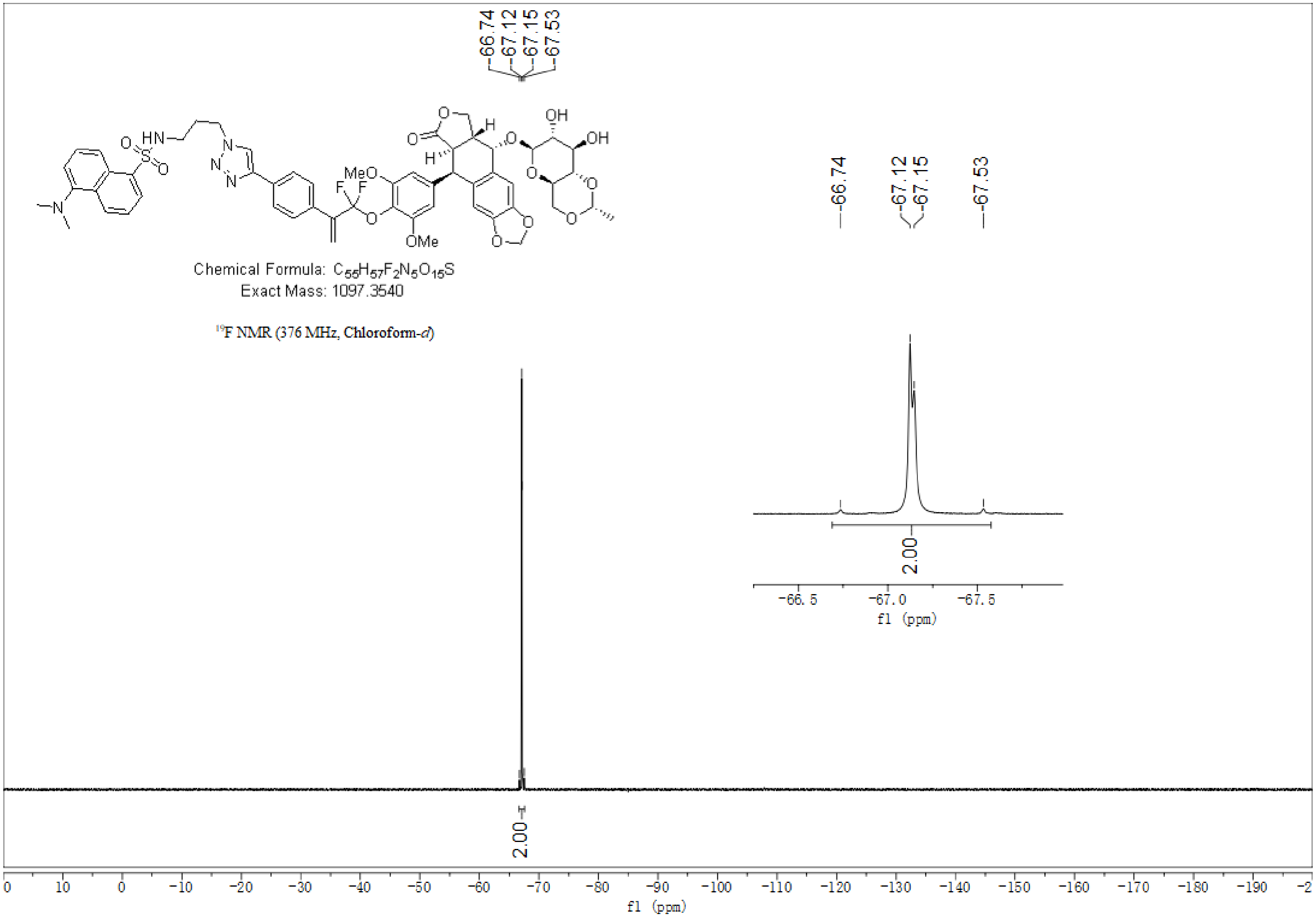

^{13}C NMR spectrum of **9** (126 MHz, $CDCl_3$)

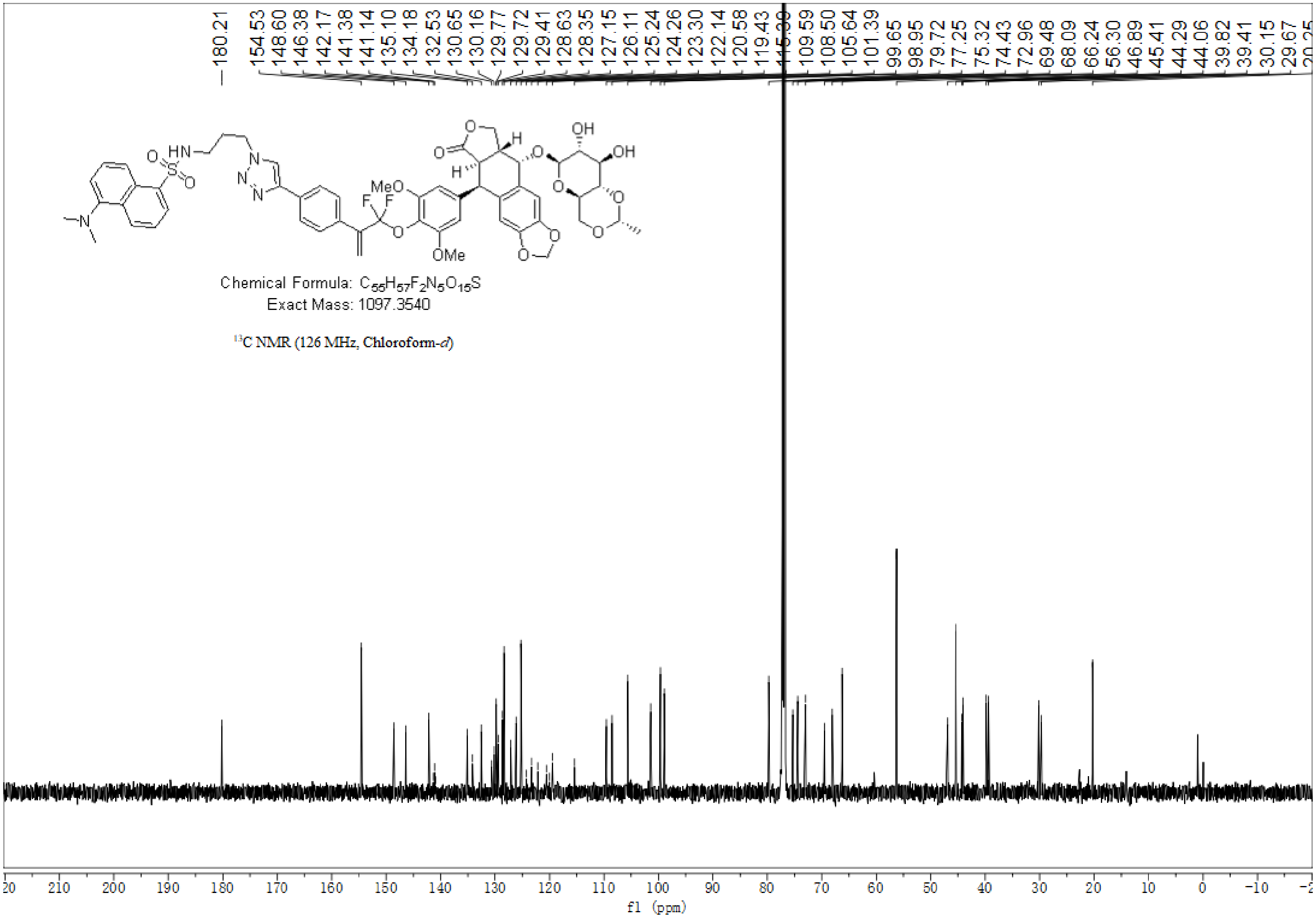

^{1}H NMR spectrum of **11a** (400 MHz, DMSO-d6)

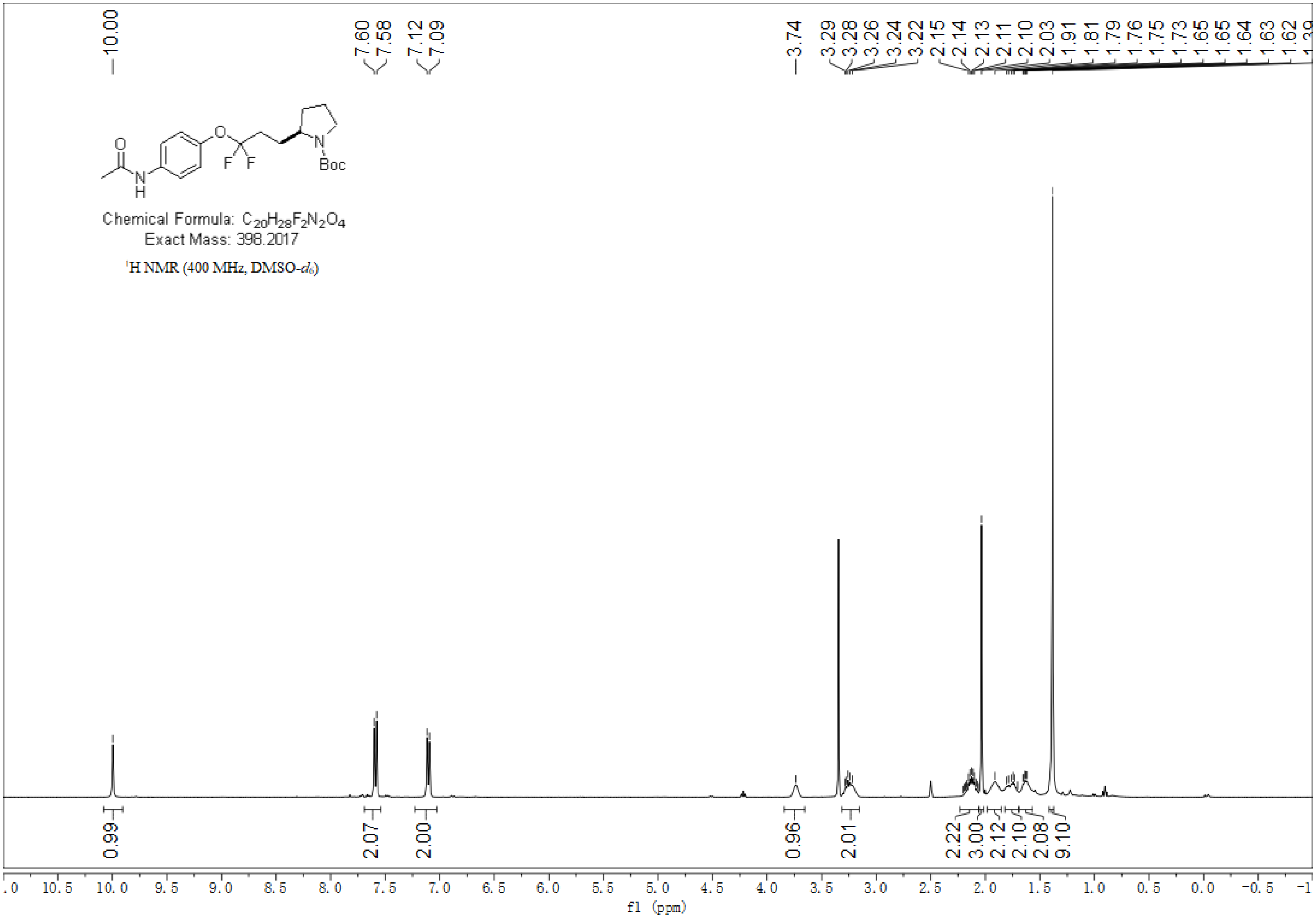

^{19}F NMR spectrum of **11a** (565 MHz, DMSO-d6, 80 °C)

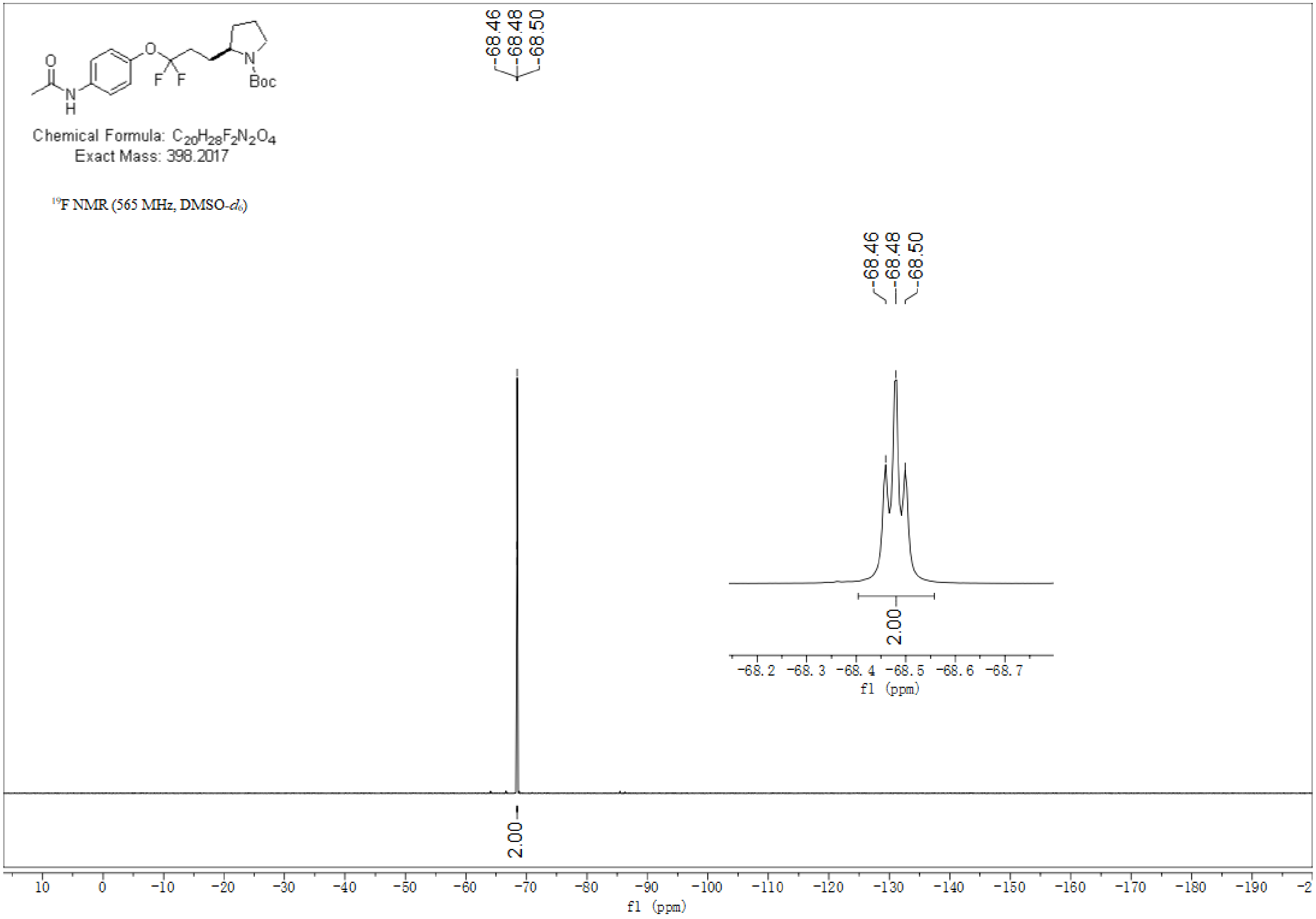

^{13}C NMR spectrum of **11a** (151 MHz, DMSO-d6, 80 °C)

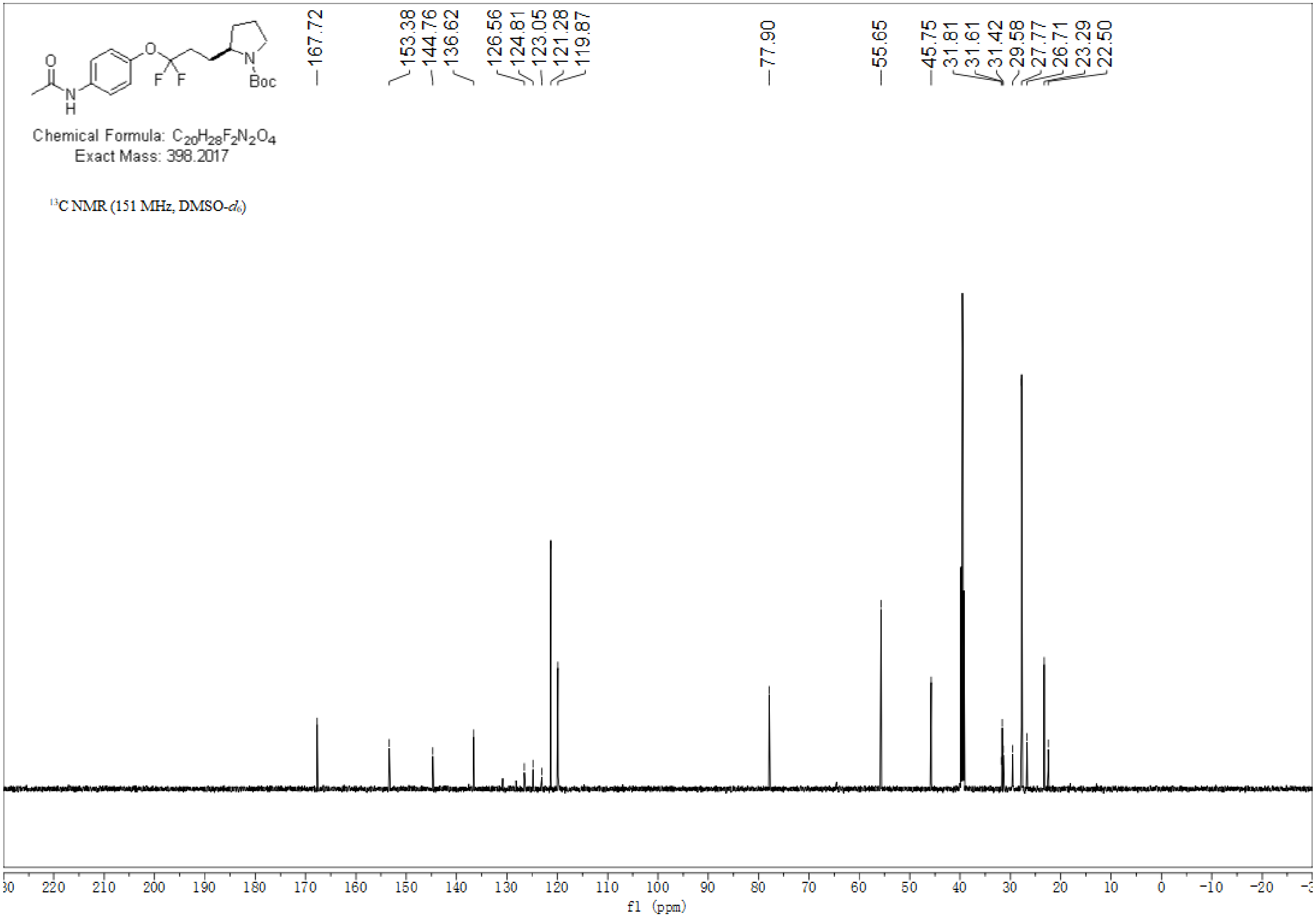

^{1}H NMR spectrum of **11b** (400 MHz, $CDCl_3$)

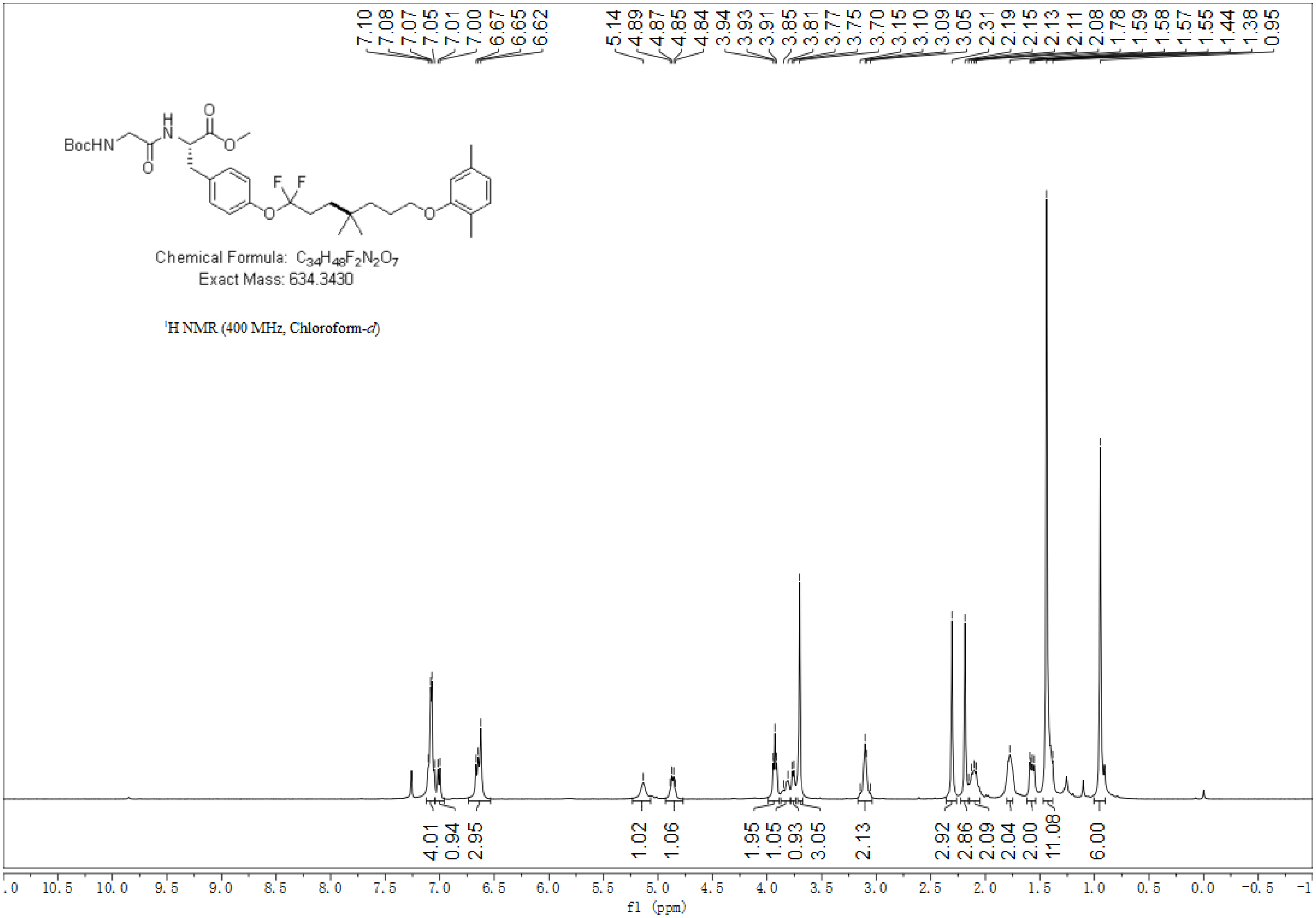

^{19}F NMR spectrum of **11b** (376 MHz, $CDCl_3$)

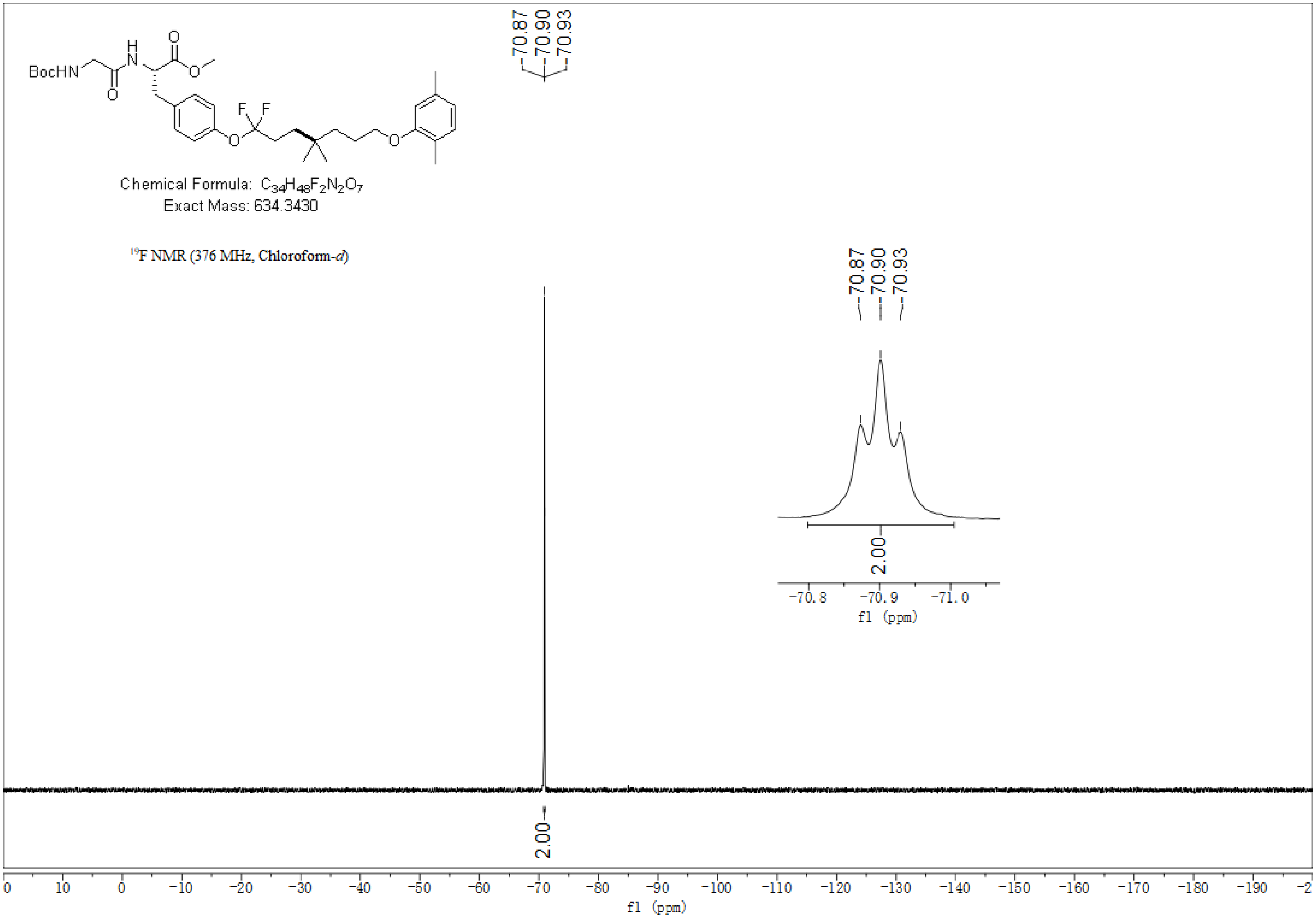

^{13}C NMR spectrum of **11b** (126 MHz, $CDCl_3$)

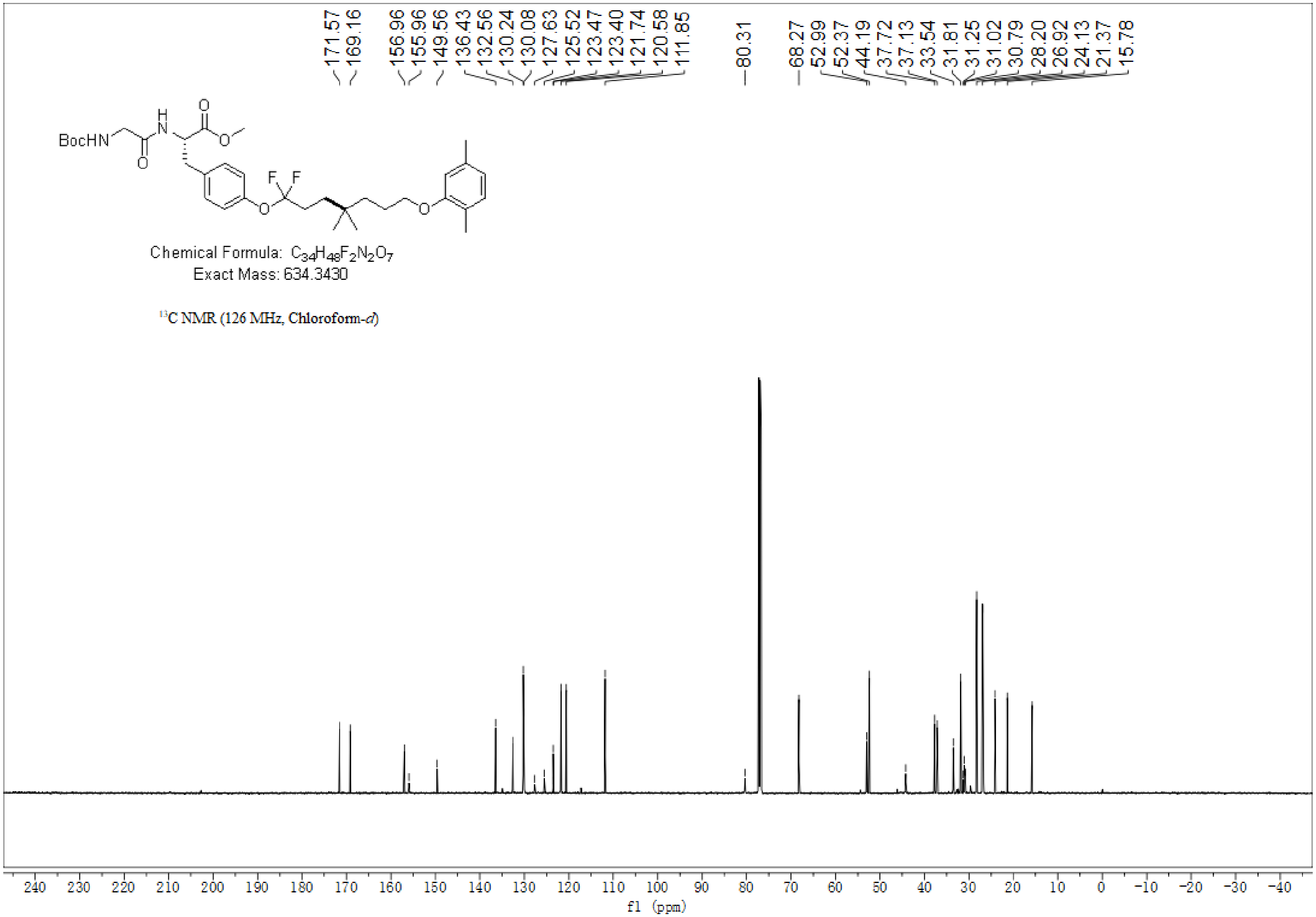

^{1}H NMR spectrum of **11c** (400 MHz, $CDCl_3$)

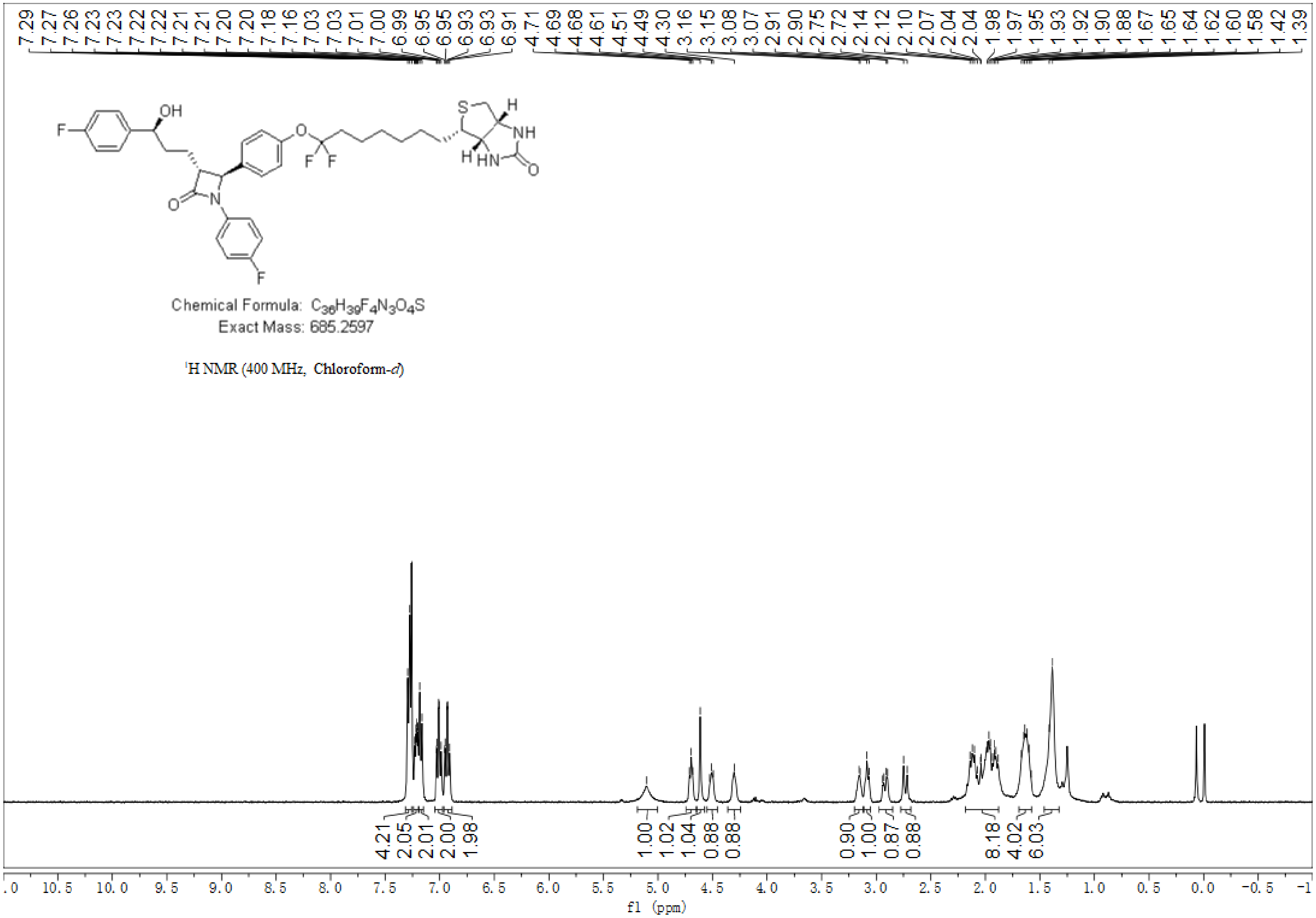

^{19}F NMR spectrum of **11c** (376 MHz, $CDCl_3$)

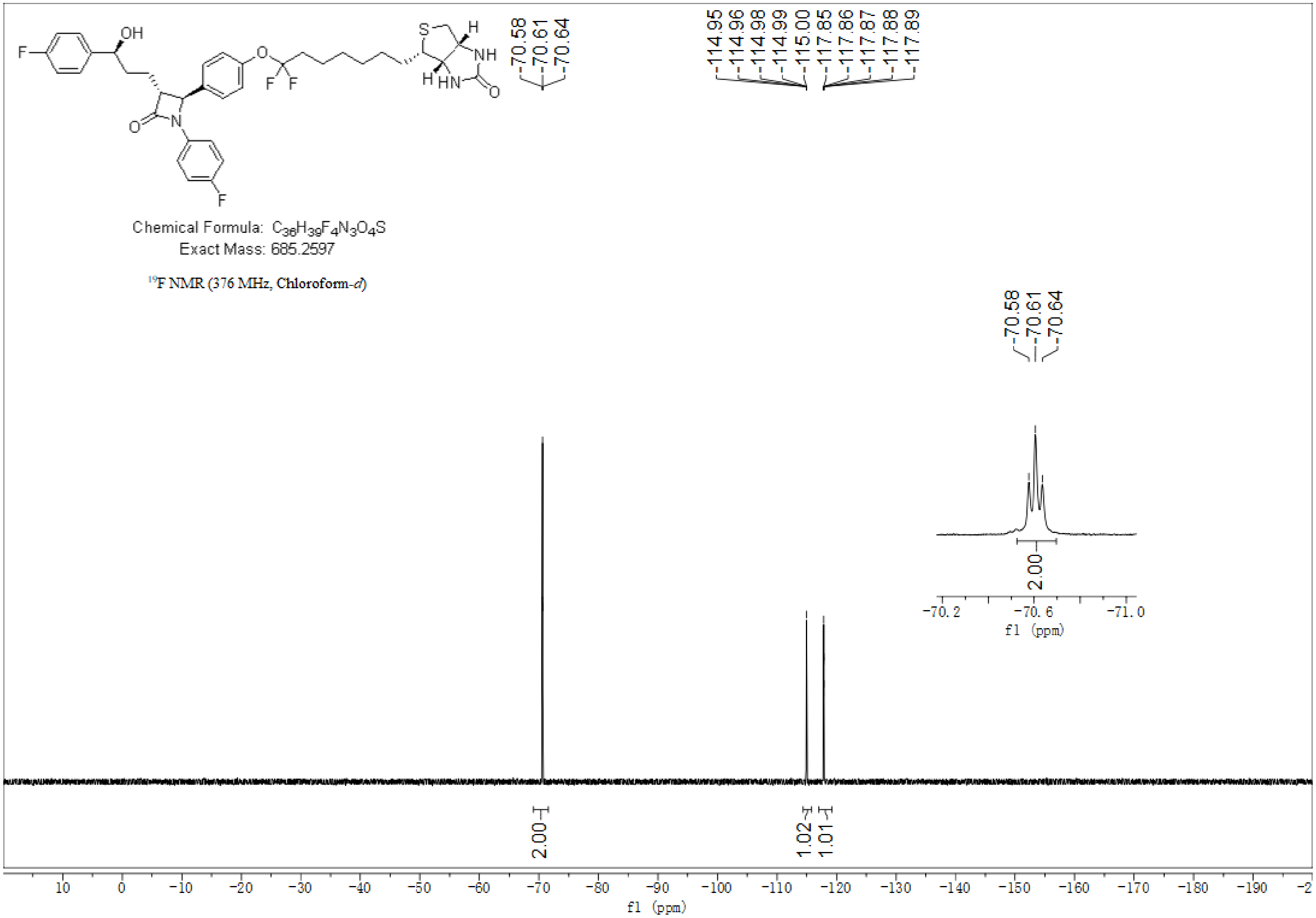

^{13}C NMR spectrum of **11c** (126 MHz, CD_3OD)

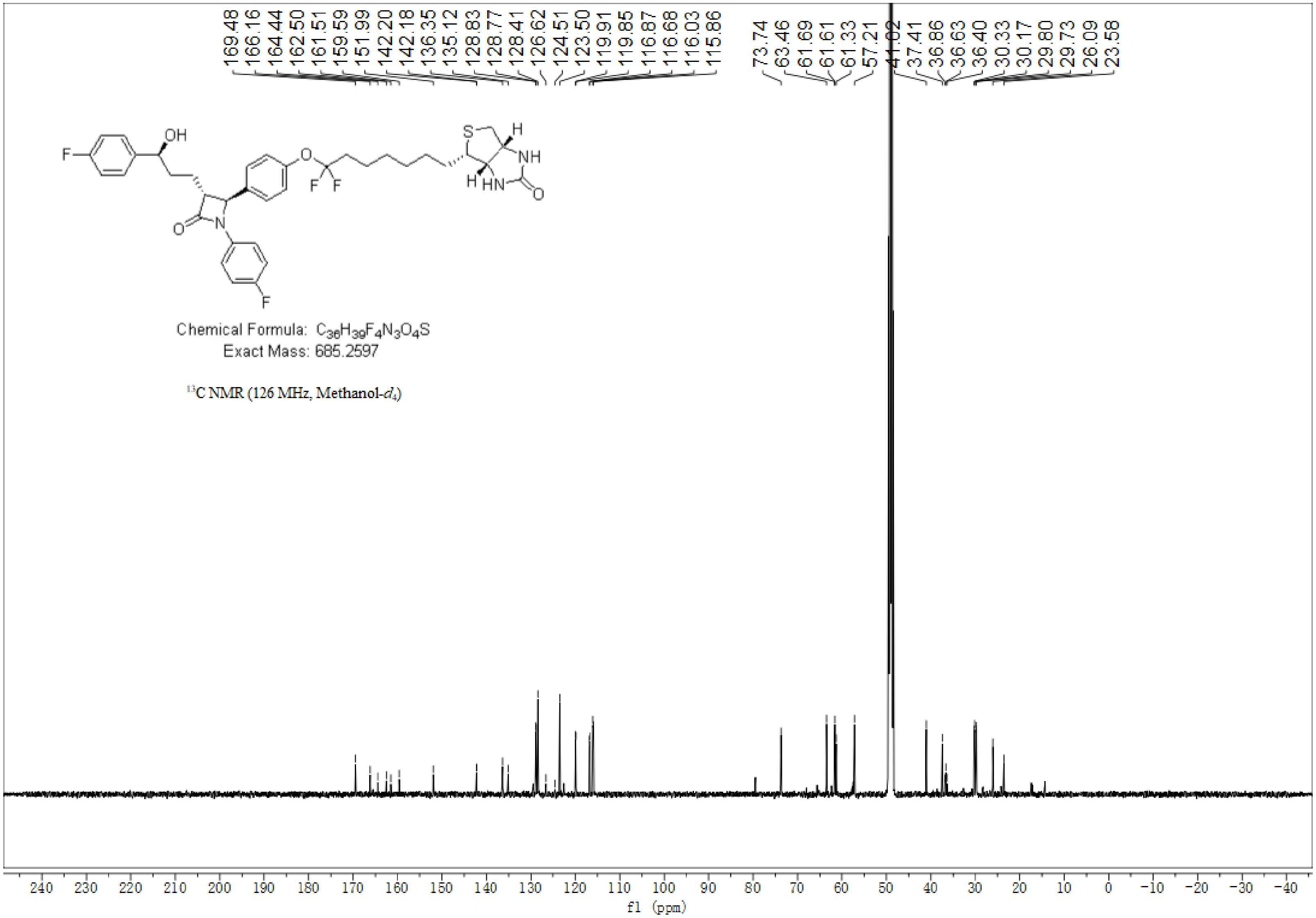

^{19}F NMR spectrum of **11d** (376 MHz, DMSO-d6)

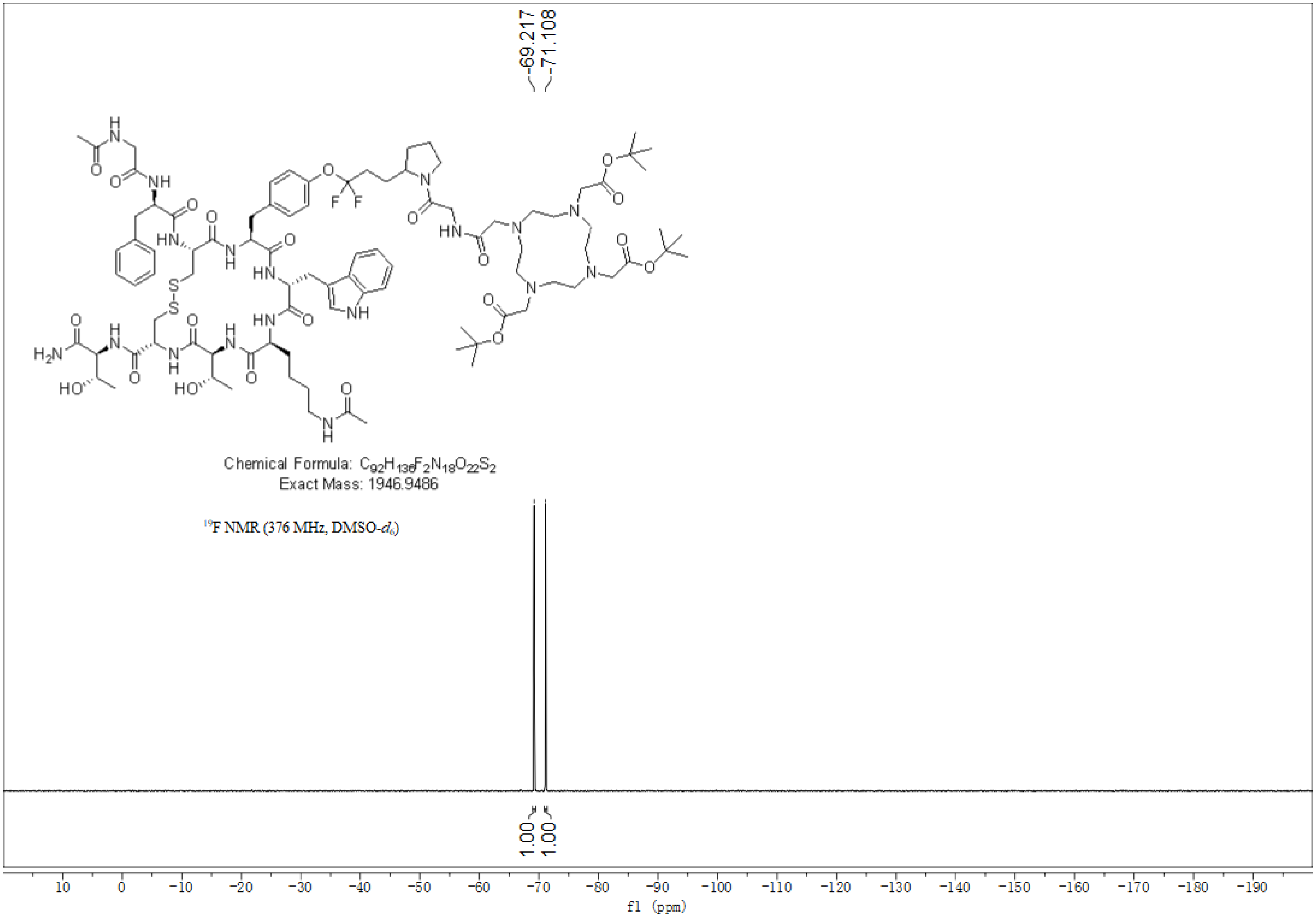

LRMS of **11d** (ESI)

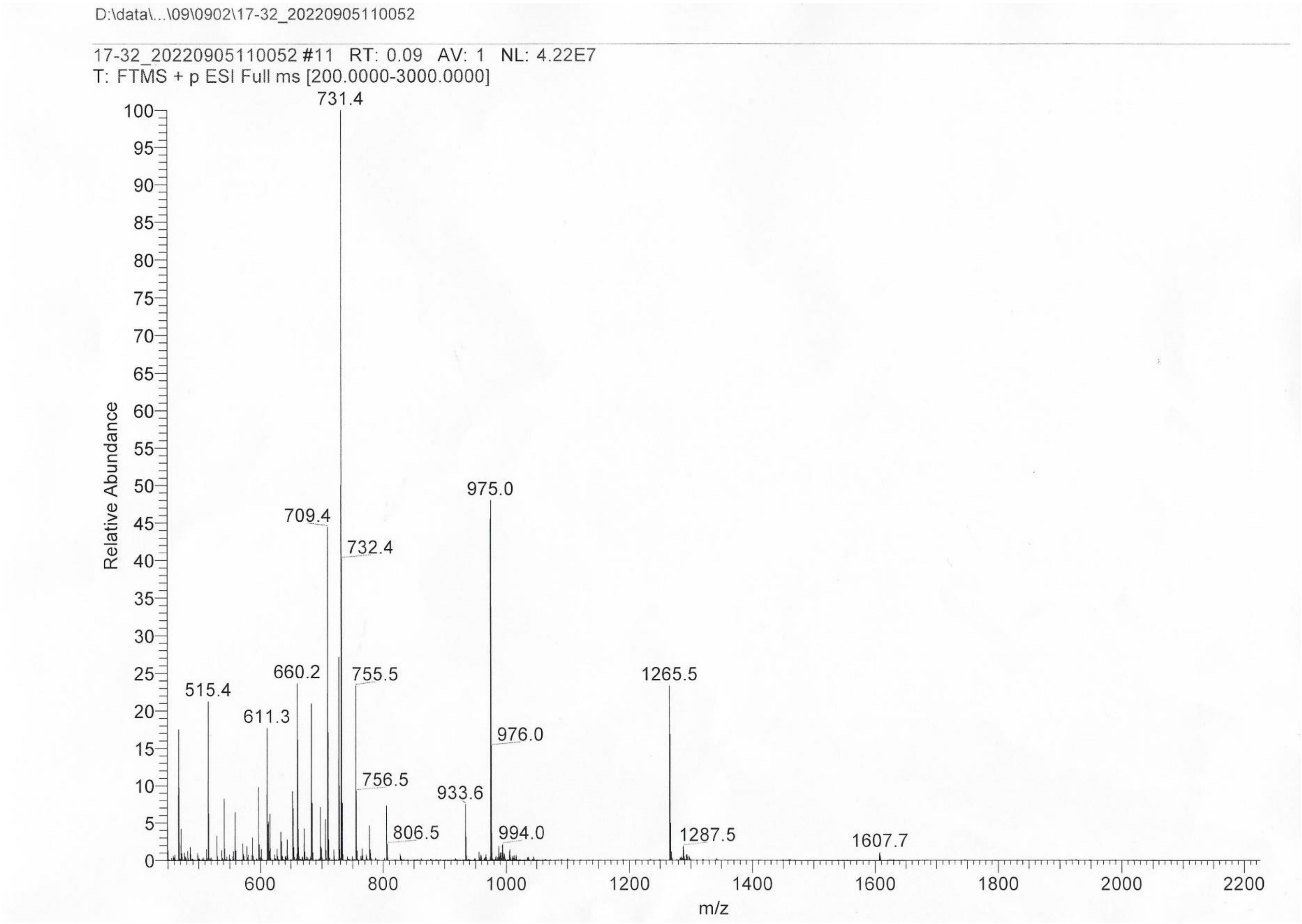

LRMS of **11d** (ESI)

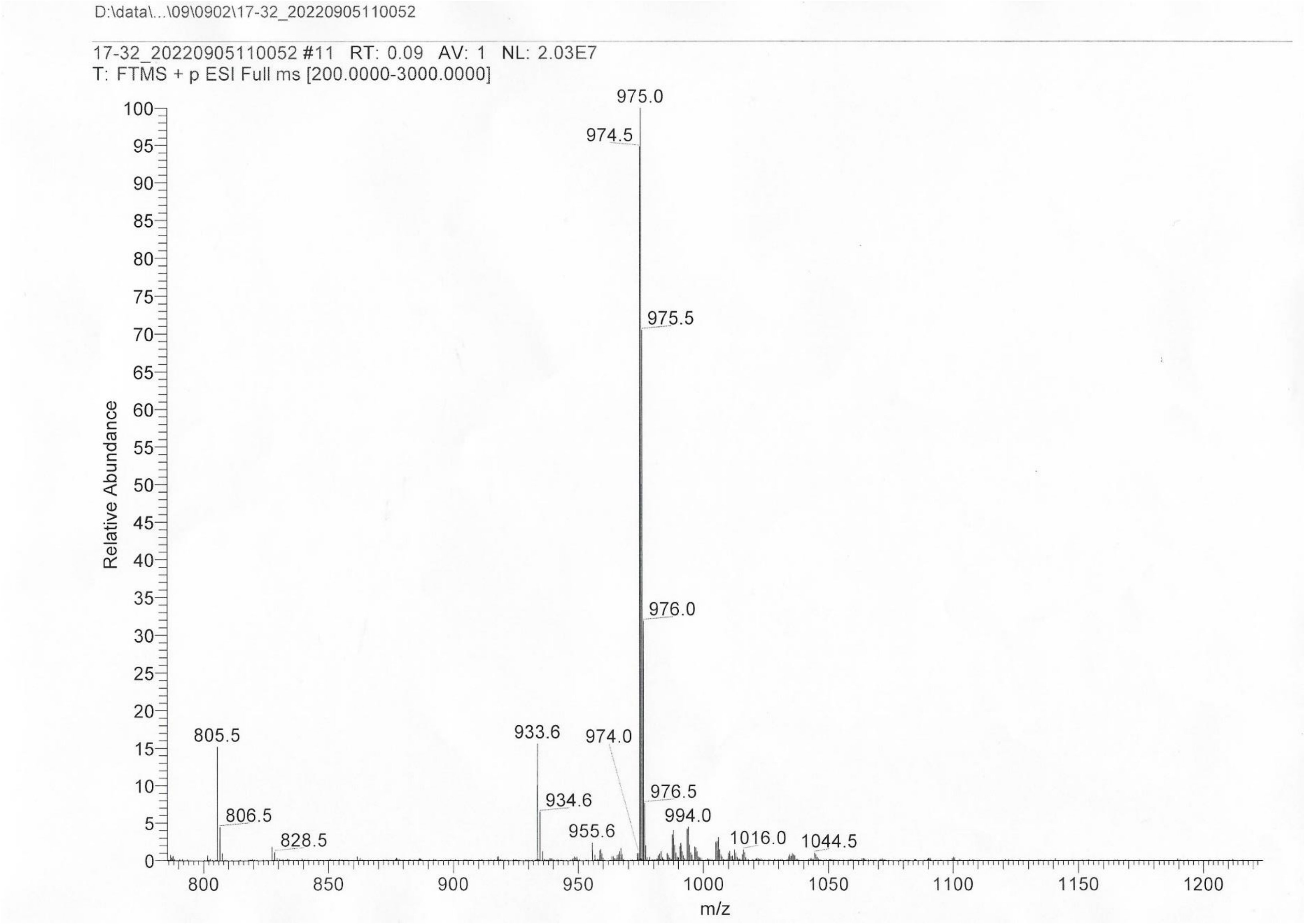

HRMS of **11d** (ESI)

National Center for Organic Mass Spectrometry in Shanghai
Shanghai Institute of Organic Chemistry
Chinese Academic of Sciences
High Resolution ESI-MS REPORT

Instrument: Thermo Scientific Q Exactive HF Orbitrap-FTMS

Card Serial Number: E221597

Sample Serial Number: 17-32

Operator: Songw Date: 2022/09/02

Operation Mode: ESI Positive Ion Mode

Charge: z= +2

Elemental composition search on mass 974.4809

m/z= 969.4809-979.4809

m/z	Theo. Mass	Delta (ppm)	RDB equiv.	Composition
974.4809	974.4816	-0.70	32.0	$C_{92} H_{138} O_{22} N_{18} F_2 S_2$

^{1}H NMR spectrum of **11e** (500 MHz, $CDCl_3$)

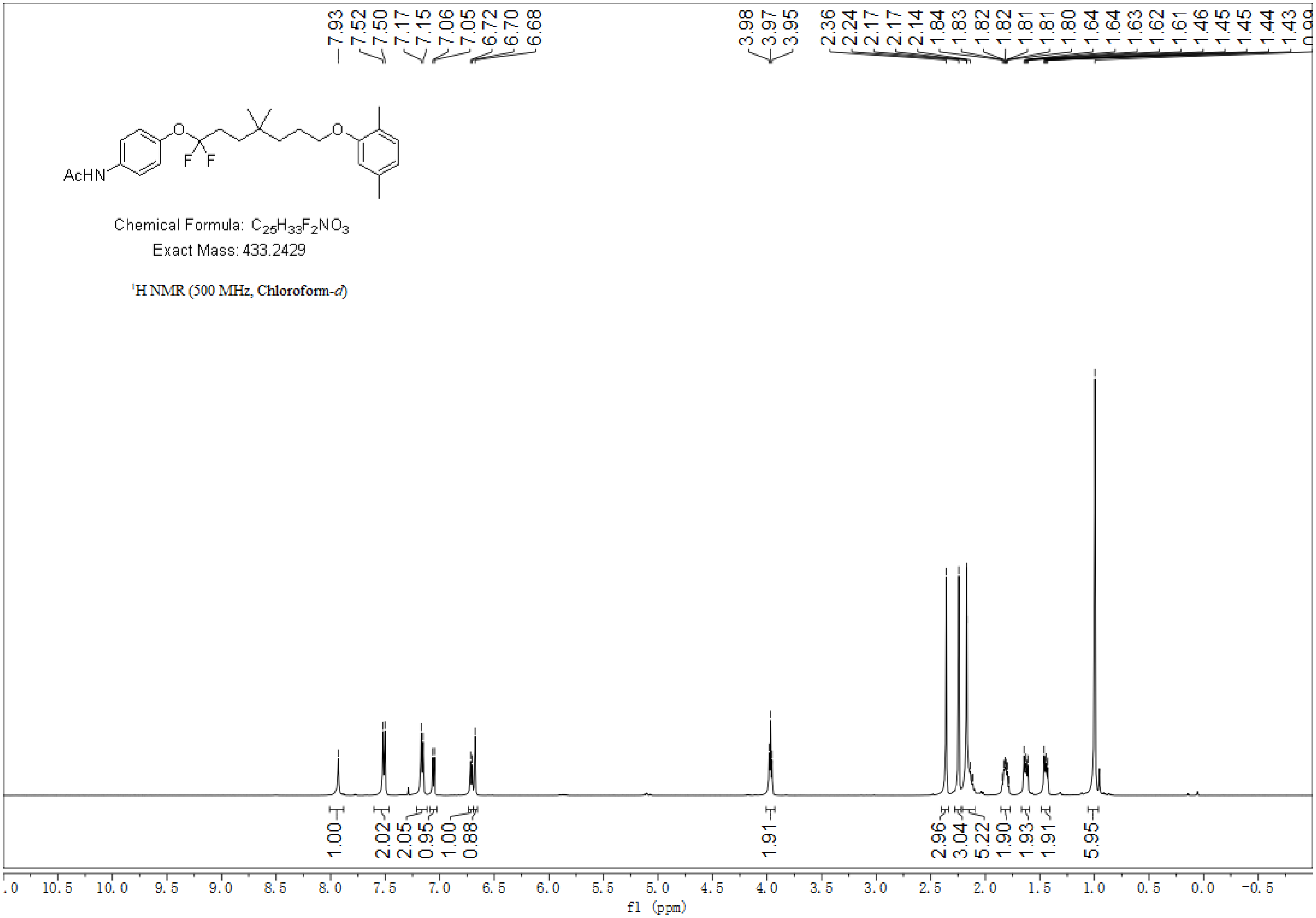

^{19}F NMR spectrum of **11e** (376 MHz, $CDCl_3$)

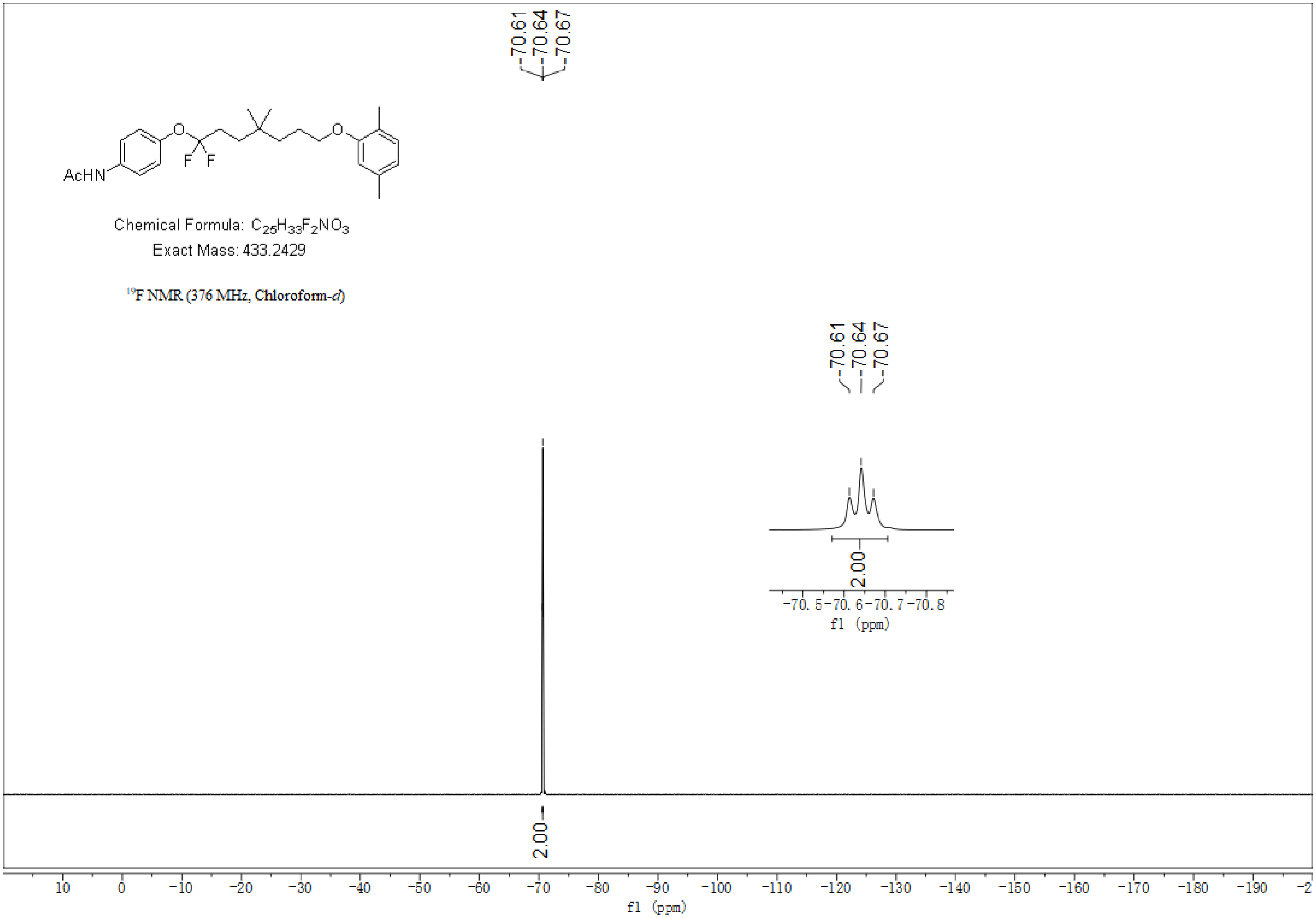

^{13}C NMR spectrum of **11e** (126 MHz, $CDCl_3$)

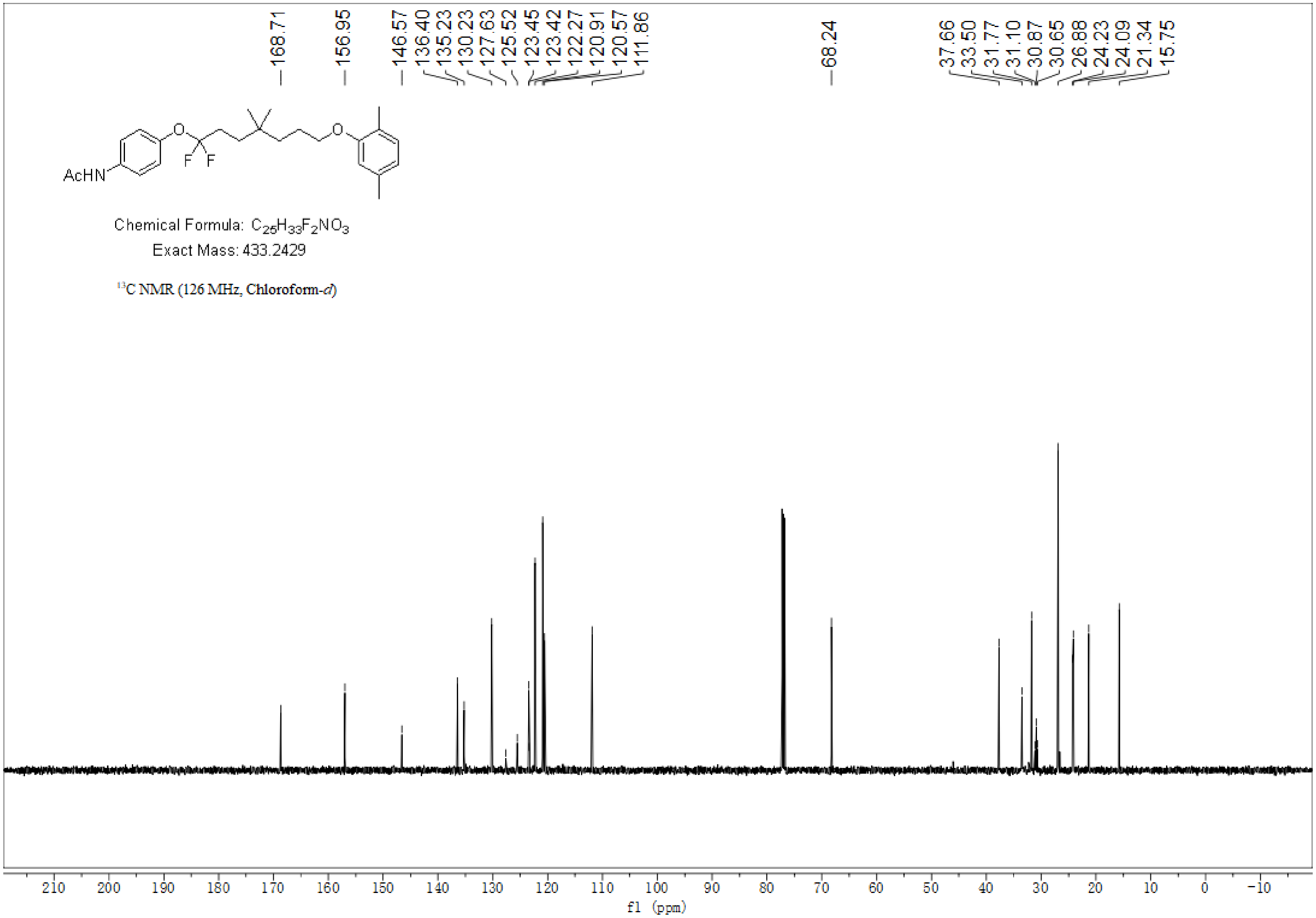

^{1}H NMR spectrum of **11e'** (400 MHz, $CDCl_3$)

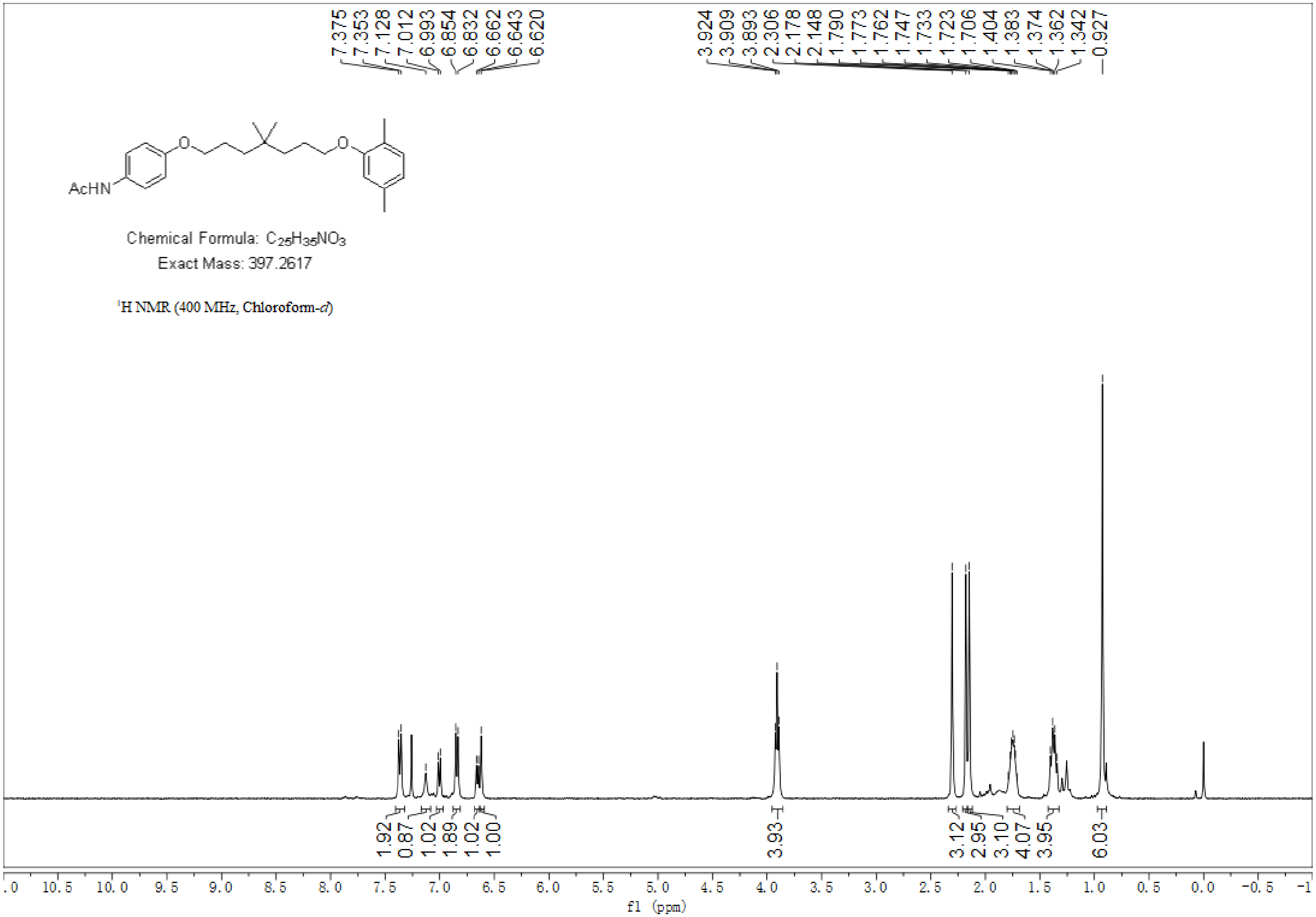

^{13}C NMR spectrum of **11e'** (126 MHz, $CDCl_3$)

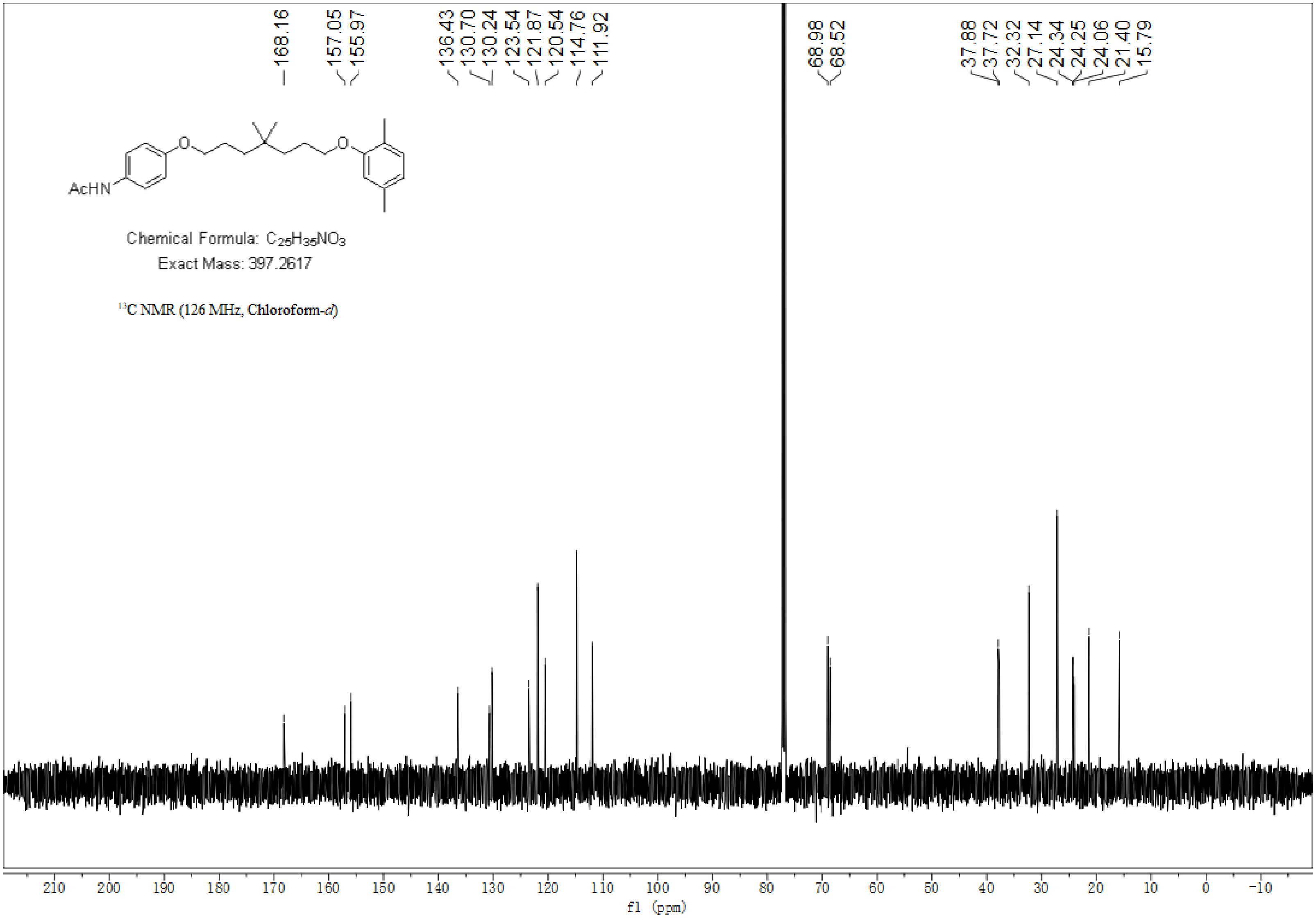

^{1}H NMR spectrum of **12** (400 MHz, DMSO-d6)

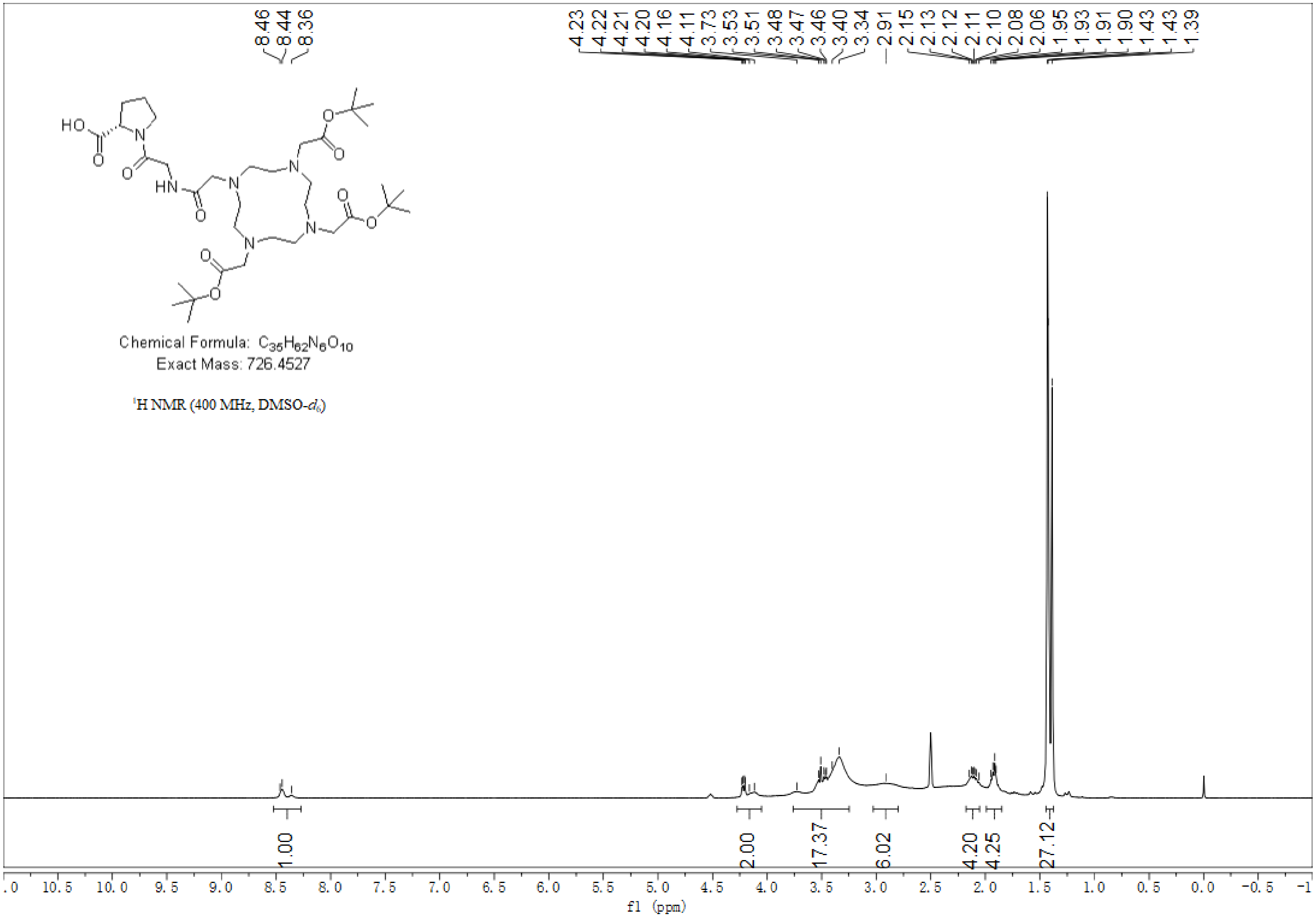

^{13}C NMR spectrum of **12** (151 MHz, DMSO-d6, 80 °C)

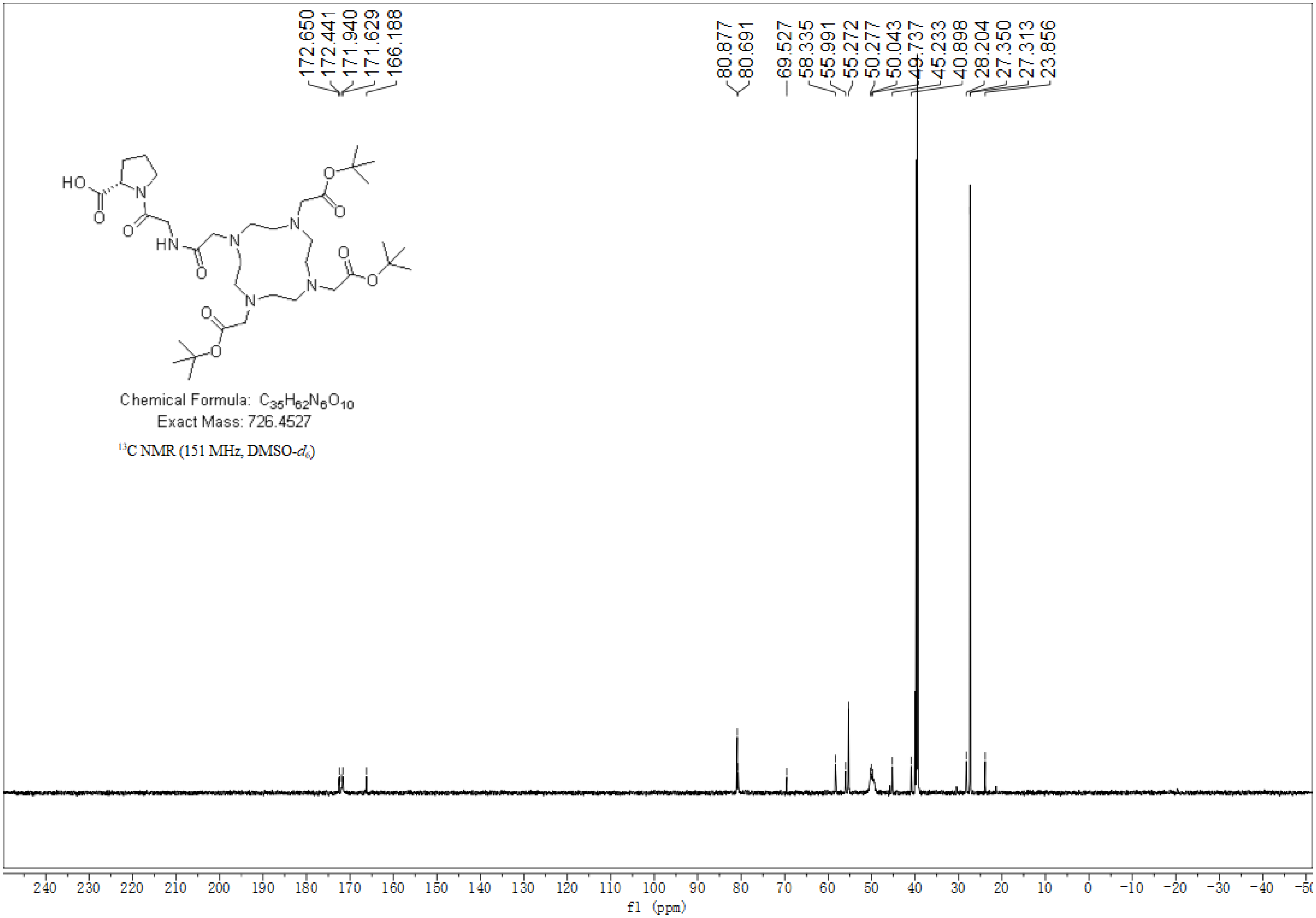

Supplement: SC-015-D3SC06302J-s001 [file SC-015-D3SC06302J-s001.pdf]
